# Cancer Hallmarks Expression in Oral Leukoplakia: Systematic Review and Meta‐Analysis

**DOI:** 10.1111/odi.70106

**Published:** 2025-09-30

**Authors:** I. González‐Ruiz, P. Ramos‐García, H. Boujemaoui‐Boulaghmoudi, N. Mjouel‐Boutaleb, M. A. González‐Moles

**Affiliations:** ^1^ Pius de Valls Hospital Tarragona España; ^2^ School of Dentistry University of Granada Granada Spain; ^3^ Instituto de Investigación Biosanitaria Ibs.GRANADA Granada Spain

**Keywords:** hallmarks of cancer, meta‐analysis, Oral cancer, Oral leukoplakia, Oral potentially malignant disorders, systematic review

## Abstract

**Objectives:**

To assess the available evidence on the expression of hallmarks of cancer and oral leukoplakia (OL) malignant transformation probability, with the goal of identifying the earliest oncogenic molecular events participating in oral cancer carcinogenesis.

**Methods:**

Embase, MEDLINE/PubMed, Scopus, and Web of Science were searched for primary‐level studies published prior to Sept 24, strictly designed as longitudinal cohorts.

**Results:**

A total of 60 studies (9758 OLs) fulfilled the eligibility criteria, and the expression of 68 different biomarkers was evaluated using the immunohistochemical technique. Sustaining proliferation hallmark was frequently harbored by OLs (PP = 56.30%, 95% CI = 43.10–69.09), significantly associated with malignant transformation (RR = 1.92, 95% CI = 1.45–2.55, *p* < 0.001), and markedly more frequent than in normal oral mucosa (OR = 7.70, 95% CI = 2.22–26.65, *p* = 0.001). Also related, genome instability markers were considerably overexpressed and associated with oral cancer development (*p* < 0.05), although resulting from a smaller sample size. Another remarkable finding is related to the activation of proinvasive mechanisms in OLs, representing the epithelial–mesenchymal transition (EMT) phenomenon, which was frequent (PP = 37.30%, 95% CI = 28.21–46.86) and significantly associated with oral cancer (RR = 3.43, 95% CI = 2.67–4.40, *p* < 0.001). Finally, avoiding immune destruction markers were also overexpressed (PP = 35.77%, 95% CI = 24.66–47.69) and significantly higher in leukoplakias progressing to oral cancer (RR = 3.65, 95% CI = 1.87–7.13, *p* < 0.001).

**Conclusions:**

Malignant transformation of OL is significantly increased in hyperproliferative lesions, which develop the EMT phenomenon and avoid immune destruction through oncogenic mechanisms.

## 1 Introduction

Oral leukoplakia (OL) is an oral potentially malignant disorder (OPMD) (Warnakulasuriya et al. 2021) with a prevalence between 1.36% and 2.60% of the general population according to the highest evidence published to date (Mello et al. 2018; Petti 2003; C. Zhang et al. 2023). OL is defined as a predominantly white plaque of questionable risk having excluded (other) known diseases or disorders that carry no increased risk for cancer (Warnakulasuriya et al. 2021). A recent meta‐analytical study conducted on 55 primary‐level studies and 41,231 OL patients indicates that the average malignancy rate of this OPMD is 6.64%, with the main malignant transformation risk factors being the presence of nonhomogeneous lesions, tongue locations, large size, smoking habits, and oral epithelial dysplasia (Pimenta‐Barros et al. 2025). Despite the importance of this OPMD, there is no evidence‐based information to date on which molecular alterations constitute relevant events in its malignization process; in other words, it is unknown which distinctive cellular characteristics—the hallmarks of cancer in the concept of Hanahan and Weinberg (Hanahan and Weinberg 2000; Hanahan and Weinberg 2011)—are expressed in oral epithelial cells affected by leukoplakia and which would presumably may serve as predictive biological markers that help identify lesions with a higher likelihood of progressing toward malignant transformation.

In order to synthesize and critically analyze the available evidence on the topic, we designed and carried out a systematic review and meta‐analysis on 60 primary‐level studies, which followed up patients over time and focused on the analysis of the expression of the distinctive signals of neoplastic cells in 9758 biopsy samples derived from areas of oral epithelium diagnosed with OL. The aim was to determine, based on available evidence, the earliest oncogenic molecular mechanisms potentially involved in the malignant transformation of this OPMD, behaving as risk factors, which could perhaps favor the establishment of preventive and therapeutic interventions in these OLs.

The objective of this meta‐analytical study was to determine the earliest oncogenic molecular mechanisms that may contribute to the malignant transformation of this OPMD. These mechanisms could act as risk factors, and their identification could support the development of early preventive strategies and targeted therapeutic interventions for OLs in the future.

## 2 Materials and Methods

In preparing and reporting this systematic review and meta‐analysis, careful consideration was given to the recommendations established by both the MOOSE (Meta‐Analysis of Observational Studies in Epidemiology) and PRISMA (Preferred Reporting Items for Systematic Reviews and Meta‐Analyses) guidelines, in order to ensure methodological transparency and comprehensive reporting (Page et al. 2021; Stroup et al. 2000). Furthermore, Cochrane Collaboration (J. P. Higgins and Green 2008) and Cochrane Prognosis Method Group (Riley et al. 2007) methodological criteria were followed to comply with an appropriate study design.

### 2.1 Protocol

A standardized protocol was established and presented to PROSPERO (ID1067519), a renowned global database that collects data from prospectively documented secondary‐level systematic reviews. In addition, for the design of the protocol, PRISMA‐P guidelines were used as a basis, which ensured strict adherence to them (Shamseer et al. 2015).

### 2.2 Search Strategy

Searches were carried out using Embase, MEDLINE (via PubMed), Scopus, and Web of Science database platforms, including studies with publication dates before the cutoff date (September 2024) with no language or date restrictions. The search method was developed through a combination of the thesaurus of the previously mentioned databases, such as MeSH and EMTREE, as well as free terms, using the keywords “oral leukoplakia,” “malignant transformation,” and synonyms. The full syntax has been adapted for each database consulted, with the aim of maximizing sensitivity (Table S1). We preferred this broad approach, as it enables the inclusion of a large number of studies investigating OL, instead of trying to devise a more precise search strategy (e.g., use of specific terms like “biomarkers” or “hallmarks of cancer”), due to the fact that the titles, keywords, and abstracts of several papers do not include biomarkers. Additionally, a manual search was performed to identify any relevant new primary‐level studies by examining the reference lists of previously included records. This process was complemented by a targeted search using Google Scholar to capture additional eligible publications that may not have been retrieved through the primary database search. Each reference was managed through the software Mendeley v.1.19.8 for the removal of duplicate records.

### 2.3 Eligibility Criteria

Inclusion criteria: (1) Studies at the primary level, not restricted to date or language of publication; (2) longitudinal studies; (3) studies in which any protein's relative differential expression is analyzed and then evaluated by immunohistochemistry, in samples obtained from patients affected by OL, with or without a comparator healthy control group, i.e., normal oral mucosa; (4) analysis of malignant transformation, comprising progression and nonprogression data for OSCC; and (5) inclusion of patients of all ages, genders, and geographical areas.

Exclusion criteria: (1) Studies not involving the expression of proteins, evaluated by the immunohistochemical technique, in samples of OL; (2) investigation of other OPMDs; (3) cross‐sectional observational studies or studies with an interventional design; (4) lacking essential statistical data for meta‐analysis; and (5) the following types of publications: abstract, animal, or in vitro basic study, secondary‐/tertiary‐level study (e.g., scoping or systematic reviews, etc.), case report, conference proceedings, editorial, book chapter, correspondence, or commentaries.

### 2.4 Process of Selection of Studies

Two teams of blinded reviewers separately applied the inclusion criteria (I.G.‐R., H.B.‐B., N.M.‐B.); disagreements with a supervising author were then resolved by consensus (P.R.G.). The following two steps were taken to select the studies: an initial phase involved a careful examination of the titles and abstracts of all retrieved records to identify studies that potentially matched the eligibility criteria. Following this preliminary filtering, the second phase consisted of a comprehensive review of the full texts of those studies deemed relevant in order to verify their suitability for inclusion based on the predefined selection standards. Both teams were trained and calibrated by the supervising author by running serial screening rounds of fifty papers at a time. An optimum inter‐rater agreement of 98.40% has been achieved. An almost perfect agreement, in terms of reliability, was obtained through the implementation of Cohen's kappa statistic (*κ* = 0.92).

### 2.5 Data Extraction

Upon a thorough examination of the full texts, an Excel standard form for gathering information (v. 16/2018, Redmond, Microsoft, WA) was used by the entire review team to extract data from the selected articles. The following information was gathered: authors, publication year, sample sizes, language and publication dates, countries, continents, anatomic sites, clinical types, age, sex, alcohol, tobacco, follow‐up, histopathology, study design, immunohistochemistry, cutoff for positivity and cellular type, and total number of positive cases for OL in the different epithelial layers and corium tissues, as well as positive cases for malignant transformation cases and healthy controls; regarding biomarkers, their respective biological and oncogenic roles, making it possible to assign a hallmark of cancer by consulting the databases HGNC (HUGO [Human Genome Organization] Gene Nomenclature Committee), NCBI (National Center for Biotechnology Information) Gene Database, and target scientific articles focused on their oncogenic roles in cancer and in OLs.

### 2.6 Evaluation of Quality and Risk of Bias of Individual Studies

The methodological quality and risk of potential bias were critically appraised by the review team, employing a specific tool developed by the Cochrane Prognosis Methods group (i.e., Quality in Prognosis Studies QUIPS tool) (Hayden et al. 2006; Hayden et al. 2013). Six potential bias areas were examined—domain 1 (D1): study participation; domain 2 (D2): study attrition; domain 3 (D3): prognostic factor measurement; domain 4 (D4): outcome measurement; domain 5 (D5): study confounding; and domain 6 (D6): statistical analysis and reporting. Each domain was rated as low, moderate, or high potential risk of bias.

### 2.7 Statistical Analysis

Relative risks (RRs) with their corresponding confidence intervals (95% CIs) were computed in order to analyze the malignant transformation probability of OLs in patients showing positive expression of cancer hallmarks. Furthermore, pooled proportions (PPs) and their 95% CIs were applied to assess variations in the expression levels of specific biomarkers observed in OL tissue samples. In order to calculate these proportions, the initial step involved extracting the raw data from each study, specifically the numerators (number of cases that showed positive biomarker expression) and the denominators (total number of OL samples analyzed). Accordingly, 95% CIs were calculated for each primary‐level study using the Wilson score method (Agresti and Coull 1998). The Freeman–Tukey double arcsine transformation was applied to stabilize the variance of the specific proportions in each study and to reduce the influence of studies with extreme values (i.e., values of 0, 100, or near these limits) (Freeman and Tuckey 1950). Then, the transformed proportions entered into meta‐analysis and were sequentially backtransformed to finally show PPs, expressed as a percentage (Miller 1978). Furthermore, a comparison was also made between the OLs and healthy oral mucosa groups to explore the magnitude of association between the expression of the hallmarks of cancer by computing and pooling odds ratios (ORs) with 95% CIs. Random‐effects models, weighted by inverse variance (DerSimonian and Laird 1986), were applied to all meta‐analyses to address potential differences among study subpopulations, for example, variability arising from different biomarkers, scoring methods, or laboratory procedures (Borenstein et al. 2010). Forest plots were constructed to graphically represent the overall meta‐analytical results. Heterogeneity was also assessed between studies through Cochrane's *Q* test; due to its low statistical power, a heterogeneity *p*‐value < 0.10 was considered significant (Higgins and Thompson 2002; Higgins et al. 2003). In addition, secondary analyses were performed to explore the presence of small‐study effects, including potential publication bias. To this end, funnel plots were generated to visually assess asymmetry, and the Egger's regression test was applied as a statistical method for detecting statistical asymmetry, with a *p*‐value threshold of < 0.10 considered indicative of significance (Egger et al. 1997). All statistical procedures were conducted using Stata software, version 16.1.

## 3 Results

### 3.1 Results of the Literature Search

**FIGURE 1:**
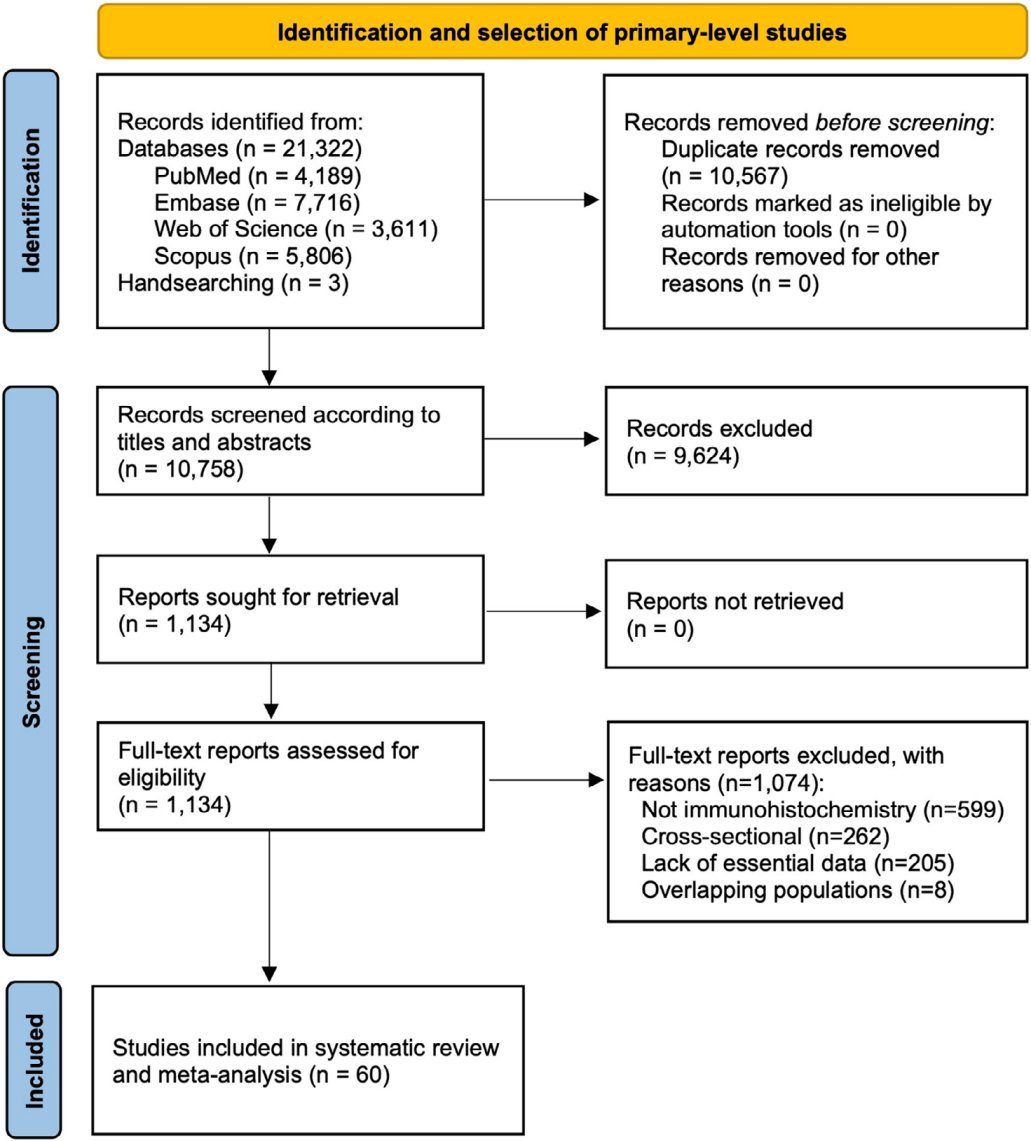
Flow diagram of the process of identification and selection of primary‐level studies offering scientific information on the hallmarks of cancer in oral leukoplakias.

The flow diagram depicts the study selection process in Figure 1. A total of 21,325 records were retrieved: 7716 of which were from Embase, 5806 from Scopus, 4189 from PubMed, 3611 from Web of Science, and 3 through handsearching methods. After duplicate deletion, 10,758 studies were considered for screening according to titles and abstracts. Following this, 1134 studies were assessed in full text, resulting in 1074 studies that did not meet the eligibility criteria and 60 included studies (Benchekroun et al. 2010; Cao et al. 2011; Chen et al. 2023; Cruz et al. 1998; D'Souza et al. 2018; de Vicente, Del Molino, et al. 2019; de Vicente, Rodríguez‐Santamarta, et al. 2019; De Vicente et al. 2013; de Villalaín et al. 2023; Ding et al. 2018; Fernández‐Valle, Rodrigo, García‐Pedrero, et al. 2016; Fernández‐Valle, Rodrigo, Rodríguez–Santamarta, et al. 2016; Gissi et al. 2015; Graveland et al. 2013; Habiba et al. 2017; Kanekawa et al. 1995; Kaur et al. 2014; Kawaguchi et al. 2008; Kikegawa 2001; Kreppel et al. 2012; Lima et al. 2016; Liu, Wu, et al. 2012; Liu, Feng, et al. 2012; Lv et al. 2020; Mao et al. 2020; Mariz et al. 2023; Matsubara et al. 2011; Matthias et al. 2008; Mondal et al. 2020; Monteiro et al. 2022; Monteiro, Silva, et al. 2021; Nayak et al. 2015; Nguyen et al. 2017; Ögmundsdóttir et al. 2009; Oliver et al. 2000; Papadimitrakopoulou et al. 1997; Rich et al. 1999; Ries et al. 2012, 2013; Saintigny et al. 2009, 2018; Sakata et al. 2020, 2017; Santos García et al. 2005; Shigeoka et al. 2020; Soni et al. 2005; Sulkowska et al. 2001; Sundberg et al. 2019; Tanda et al. 2000; Tarle et al. 2022; Weber et al. 2020; Wils et al. 2023; Wu et al. 2019; Wu et al. 2022; Xia et al. 2013; Xu et al. 2023; Yagyuu et al. 2021; Zhang, Kim, Zheng, Bazarsad, and Kim 2017; Zhang, Kim, Zheng, Kim, et al. 2017; Zhu et al. 2018) (the included studies as well as the full‐text reports excluded with reasons can also be found, respectively, in the Appendices S1, S2).

### 3.2 Study Characteristics

The general characteristics of the study sample are summarized in Table 1 whereas Table S2 describes them in detail. Publication dates ranged between 1995 and 2023; all studies were designed as longitudinal cohorts, with 52 being retrospective and 8 prospective nature. The 60 included primary‐level studies investigated a total of 9758 samples of oral epithelium affected by OL (range: 13–200), in which the expression of 68 different biomarkers was investigated using the immunohistochemical technique. These biomarkers were categorized as follows: sustaining proliferative signaling (AgNOR, c‐jun, cyclin D1, EGFR, EZH2, FGF‐2, FGFR‐1, FGFR‐2, FGFR‐3, KCNC4, KCNH2, ki‐67, Notch1, PCNA, PTMA); evading growth suppressors (14‐3‐3σ, DPC4, p16, p21, p27, p53, p63, p73, pRb); resisting cell death (14–3‐3ζ, Bcl‐2, DcR2, MDM2, ΔNp63); enabling replicative immortality (BMI‐1); inducing angiogenesis (c‐met); activating invasion and metastasis (ABCG2, ALDH1, Axin2, BSG, NANOG, podoplanin, PROM1, Snail, SOX2); avoiding immune destruction (CD163, Foxp3, PD‐1, PD‐L1, TIPE2); deregulating cellular energetics (CA9); genome instability (ATM, BUB3, BubR1, HNRNPK, Mad2, MAGE‐A, SPINDLY, γH2AFX); tumor‐promoting inflammation (CD11c, CD3, CD68, CD8, COX2); and an additional group of unspecified biomarkers (CD44v6, CK13, CK17, CK8, Dec1, LAMC2, PTHrP, S100A7, β‐catenin). These biomarkers were then classified by roles (Table S4) and subsequently by hallmarks of cancer. With regard to geographical location, 28 studies were from Asia (4 countries), 24 from Europe (10 countries), 6 from North America (2 countries), 2 from South America (1 country), and only one from Oceania (1 country). Most of them were published in English (*n* = 57), while 1 was in Chinese, 1 in Japanese, and 1 in Spanish.

**TABLE 1:** Summarized characteristics of the study sample.

Total	60 studies
Year of publication	1995–2023
Total cases (range)	9758^a^ (13–200)
Study design	
Retrospective cohort	52
Prospective cohort	8
Experimental methods	
Immunohistochemistry	60 studies (68 biomarkers)
Publication languages	English (57 studies)
	Chinese (1 study)
	Japanese (1 study)
	Spanish (1 study)
Geographical region	
Europe	23 studies (10 countries)
Asia	28 studies (4 countries)
North America	6 studies (2 countries)
South America	2 studies (1 countries)
Oceania	1 study (1 country)
Total	5 continents, 18 countries

^a^ More than one biomarker was analyzed per study.

### 3.3 Qualitative Evaluation

**FIGURE 2:**
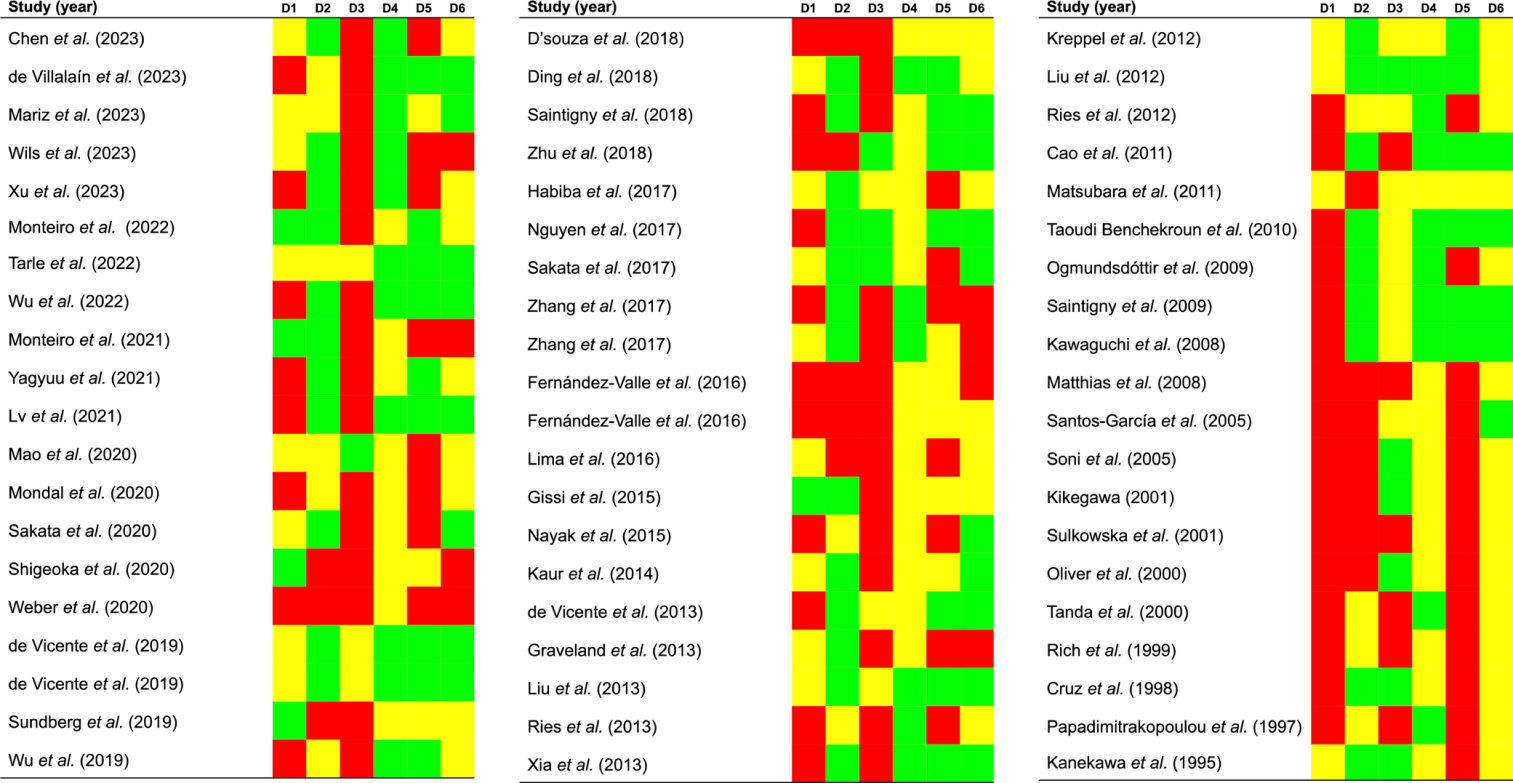
Quality plot graphically representing the risk of bias across the primary‐level studies included in this systematic review, qualitatively assessed applying the QUIPS tool. The most relevant sources of risk of bias were critically judged through six specific domains: (1) Study participation, (2) Study attrition, (3) Prognostic factor measurement, (4) Outcome measurement, (5) Study confounding, and (6) Statistical analysis and reporting. Red color represents a high risk of potential bias, yellow color moderate risk, and green color low risk.

The assessment of the methodological rigor and risk of bias was done using the QUIPS tool, identifying the potential sources of bias in six different domains:

*D1*. Risk of potential bias was high in 58.33% of the studies, moderate in 33.33%, and low in 8.33% (Figure 2). A number of studies fail to report pertinent data on specific subpopulations. This data includes the recruitment period and/or location, sex, lesion sites, and age, among other information.

*D2*. Twenty‐five percent of studies showed a high risk of bias, 20% moderate, and 55% low (Figure 2). The loss of subjects during the follow‐up process is frequently not reported. Furthermore, shortcomings in the reporting of essential patient follow‐up periods (i.e., total months and average periods) were identified.

*D3*. Risk of potential bias was high in 60% of the studies, moderate in 23.33%, and low in 16.67% (Figure 2). It is important to note that many of the studies do not clearly report the immunohistochemical study methodology (e.g., the antibodies and dilutions used) as well as the ways in which positivity or the cutoff points are determined.

*D4*. Risk of potential bias was moderate in 56.67%, and low in 43.33% (Figure 2). In this systematic review, the eligibility criteria meant that all studies included cases with malignant transformation confirmed by biopsy or histopathological examination. The studies deemed to be of moderate risk are those that report a short follow‐up period, as time is a crucial factor in the development of oral cancer in OL.

*D5*. Risk of potential bias was high in 46.67% of the studies, moderate in 16.67%, and low in 36.67% (Figure 2). Studies that did not properly control for potentially confounding factors (e.g., sex, age, alcohol, or tobacco) or were not adjusted by a multivariate model were penalized.

*D6*. Risk of potential bias was high in 13.33% of the studies, moderate in 50%, and low in 36.67% (Figure 2). Some of the studies failed to report ratio metrics of malignant transformation and their confidence intervals.

### 3.4 Quantitative Evaluation (Meta‐Analysis)

The meta‐analytical results have been documented in Table 2, graphically represented in forest plots (Figures S1–S27), as well as in a forest top plot (Figure 3).

**TABLE 2:** Meta‐analysis on the expression of hallmarks of cancer in oral leukoplakia.

Meta‐analyses	No. of studies	No. of cases^a^	Stat. Model	Wt	Pooled data		Heterogeneity	
					ES (95% CI)	*p*	P_het_	*I* ^2^ (%)
*Hallmark 1: Sustaining proliferative signaling*								
Expression of hallmarks of cancer and OL malignant transformation probability								
Oncogenic (pro‐proliferative)	27	1949	REM	D‐L	RR = 1.92 (1.45–2.55)	< 0.001	0.06	31.9
Differential expression in OL								
Oncogenic (pro‐proliferative)	26	1919	REM	D‐L	PP = 56.30% (43.10–69.09)	—	< 0.001	97.0
Comparison between expression in OL vs. healthy controls								
Oncogenic (pro‐proliferative)	5	529	REM	D‐L	OR = 7.70 (2.22–26.65)	0.001	0.62	0.0
*Hallmark 2: Evading growth suppressors*								
Expression of hallmarks of cancer and OL malignant transformation probability								
Protector (growth suppressor)	28	1932	REM	D‐L	RR = 1.21 (0.84–1.75)	0.31	< 0.001	60.9
Differential expression in OL								
Protector (growth suppressor)	27	1842	REM	D‐L	PP = 51.27% (41.63–60.87)	—	< 0.001	94.0
Comparison between expression in OL vs. healthy controls								
Protector (growth suppressor)	10	702	REM	D‐L	OR = 5.58 (2.47–12.58)	< 0.001	0.21	25.5
*Hallmark 3: Resisting cell death*								
Expression of hallmarks of cancer and OL malignant transformation probability								
Oncogenic (antiapopotic)	8	625	REM	D‐L	RR = 0.94 (0.43–2.03)	0.87	< 0.001	81.1
Differential expression in OL								
Oncogenic (antiapopotic)	8	625	REM	D‐L	PP = 69.11% (39.36–92.37)	—	< 0.001	98.1
Comparison between expression in OL vs. healthy controls								
Oncogenic (antiapopotic)	2	44	REM	D‐L	OR = 6.17 (0.85–44.84)	0.07	0.48	0.0
*Hallmark 4: Enabling replicative immortality*								
Expression of hallmarks of cancer and OL malignant transformation probability								
Oncogenic (immortalization)	2	244	REM	D‐L	RR = 3.74 (1.94–7.21)	< 0.001	0.83	0.0
Differential expression in OL								
Oncogenic (immortalization)	2	244	REM	D‐L	PP = 41.26% (24.56–59.04)	—	0.01	87.4
Comparison between expression in OL vs. healthy controls								
Oncogenic (immortalization)	0	0	REM	D‐L	—	—	—	—
*Hallmark 5: Inducing angiogenesis*								
Expression of hallmarks of cancer and OL malignant transformation probability								
Oncogenic (proangiogenic)	2	280	REM	D‐L	RR = 3.74 (1.79–7.82)	< 0.001	0.91	0.0
Differential expression in OL								
Oncogenic (proangiogenic)	2	280	REM	D‐L	PP = 43.80% (32.41–55.52)	—	0.05	74.4
Comparison between expression in OL vs. healthy controls								
Oncogenic (proangiogenic)	0	0	REM	D‐L	—	—	—	—
*Hallmark 6: Activating invasion and metastasis*								
Expression of hallmarks of cancer and OL malignant transformation probability								
Oncogenic (proinvasive)	18	1602	REM	D‐L	RR = 3.43 (2.67–4.40)	< 0.001	0.92	0.0
Differential expression in OL								
Oncogenic (proinvasive)	18	1602	REM	D‐L	PP = 37.30% (28.21–46.86)	—	< 0.001	93.2
Comparison between expression in OL vs. healthy controls								
Oncogenic (proinvasive)	6	674	REM	D‐L	OR = 2.20 (0.39–12.42)	0.37	< 0.001	84.5
*Hallmark 7: Avoiding immune destruction*								
Expression of hallmarks of cancer and OL malignant transformation probability								
Oncogenic (Antitumor arrest)	7	810	REM	D‐L	RR = 3.65 (1.87–7.13)	< 0.001	0.1	43.9
Differential expression in OL								
Oncogenic (Antitumor arrest)	7	810	REM	D‐L	PP = 35.77% (24.66–47.69)	—	< 0.001	91.5
Comparison between expression in OL vs. healthy controls								
Oncogenic (Antitumor arrest)	0	0	REM	D‐L	—	—	—	—
*Hallmark 8: Deregulating cellular energetics*								
Expression of hallmarks of cancer and OL malignant transformation probability								
Oncogenic (tumor acidosis)	1	160	REM	D‐L	RR = 4.57 (0.99–21.03)	0.051	—	0.0
Differential expression in OL								
Oncogenic (tumor acidosis)	1	160	REM	D‐L	PP = 42.50% (34.92–50.21)	—	—	0.0
Comparison between expression in OL vs. healthy controls								
Oncogenic (tumor acidosis)	1	178	REM	D‐L	OR = 27.40 (1.62–462.61)	0.02	—	0.0
*Hallmark 9: Genome instability and mutation*								
Expression of hallmarks of cancer and OL malignant transformation probability								
Oncogenic (DNA instability)	1	98	REM	D‐L	RR = 8.14 (4.07–16.29)	0.009	—	0.0
Protector (DNA damage repair)	7	506	REM	D‐L	RR = 4.31 (1.44–12.84)	< 0.001	< 0.001	83.0
Differential expression in OL								
Oncogenic (DNA instability)	1	98	REM	D‐L	PP = 41.84% (32.22–51.77)	—	—	0.0
Protector (DNA damage repair)	7	506	REM	D‐L	PP = 54.15% (25.19–81.69)	—	< 0.001	97.8
Comparison between expression in OL vs. healthy controls								
Oncogenic (DNA instability)	1	123	REM	D‐L	OR = 36.81 (2.18–621.98)	0.01	—	0.0
Protector (DNA damage repair)	4	246	REM	D‐L	OR = 3.24 (1.38–7.60)	0.007	0.96	0.0
*Hallmark 10: Tumor promoting inflammation*								
Expression of hallmarks of cancer and OL malignant transformation probability								
Oncogenic (proinflammatory)	5	523	REM	D‐L	RR = 2.32 (1.63–3.30)	< 0.001	0.92	0.0
Differential expression in OL								
Oncogenic (proinflammatory)	5	523	REM	D‐L	PP = 42.50% (27.17–58.59)	—	< 0.001	92.7
Comparison between expression in OL vs. healthy controls								
Oncogenic (proinflammatory)	1	178	REM	D‐L	OR = 11.23 (0.66–190.89)	0.09	—	0.0

Abbreviations: CI, confidence intervals; D‐L, DerSimonian and Laird method; OL, leukoplakia; OR, odds ratio; PP, pooled proportion; RR, relative risk; Stat., statistical; REM, random‐effects model; Wt, method of weighting.

^a^ Note that more than one analysis unit was analyzed per study and patient.

**FIGURE 3:**
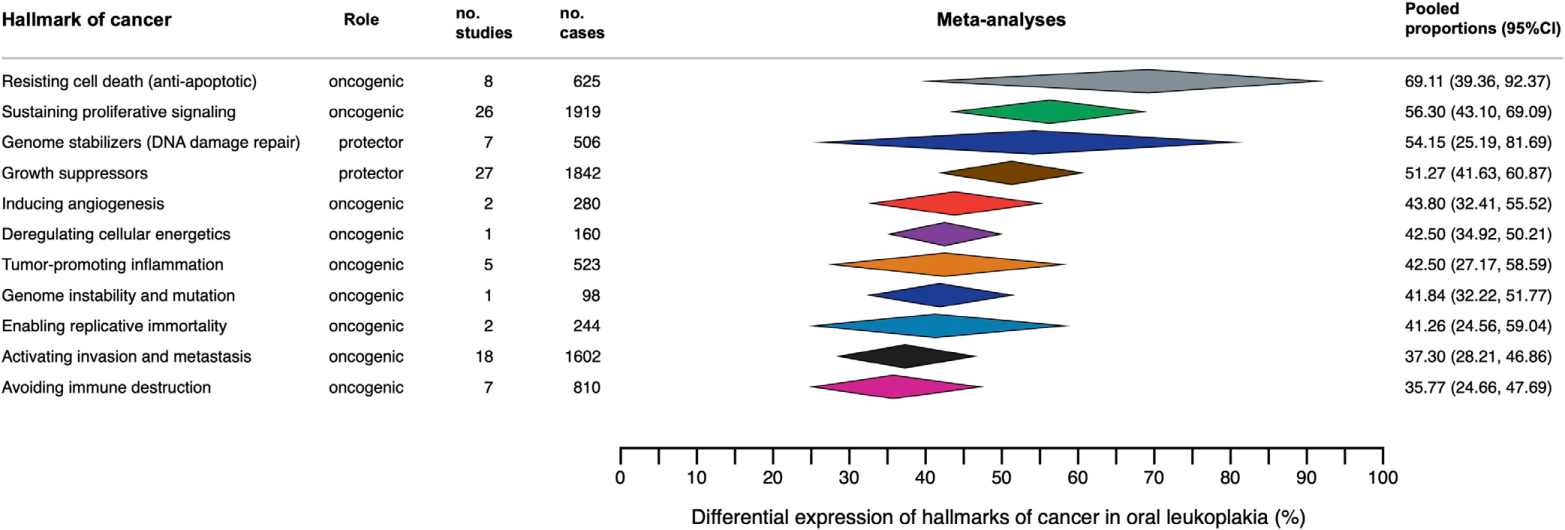
Summary Forest Plot (aka *forest top plot*) graphically representing pooled proportions—expressed as percentages—with their corresponding 95% confidence intervals, obtained through the meta‐analyses on the hallmarks of cancer in oral leukoplakias (OL). This plot exhibits the results of all meta‐analyses carried out row by row, the meta‐analyses findings were depicted as diamonds‐ according to the different hallmarks of cancer expressed in OL (*n* = 11 different meta‐analyses of proportions performed in this study).

#### 3.4.1 Hallmark of Cancer No. 1

##### 3.4.1.1 Expression of Sustaining Proliferative Signaling Hallmark and OL Malignant Transformation Probability

The relative risk (RR) associated with the expression of pro‐proliferative biomarkers was 1.92 (95% CI = 1.45–2.55, *p* < 0.001), estimated from a large sample size (27 primary‐level studies/1949 cases).

##### 3.4.1.2 Differential Expression in OL

The estimated PP for sustaining proliferative signaling was 56.30% (95% CI = 43.10–69.09), from a meta‐analysis of 26 studies and 1919 cases.

##### 3.4.1.3 Comparison Between Expression in OL vs. Healthy Controls

OL cases showed a significantly higher frequency for pro‐proliferative biomarkers (OR = 7.70, 95% CI = 2.22–26.65, *p* = 0.001) (5 studies/529 cases).

#### 3.4.2 Hallmark of Cancer No. 2

##### 3.4.2.1 Expression of Evading Growth Suppressors Hallmark and OL Malignant Transformation Probability

The magnitude of association between the expression of tumor growth suppressor biomarkers and malignant transformation showed nonsignificant results (RR = 1.21, 95% CI = 0.84–1.75, *p* = 0.31) (28 studies/1932 cases).

##### 3.4.2.2 Differential Expression in OL

The estimated PP for tumor evading growth suppressors was 51.27% (95% CI = 41.63–60.87), once again from a large sample size (27 studies/1842 cases).

##### 3.4.2.3 Comparison Between Expression in OL vs. Healthy Controls

OL cases showed a significantly higher frequency for tumor growth suppressor biomarkers (OR = 5.58, 95% CI = 2.47–12.58, *p* < 0.001) (10 studies/702 cases).

#### 3.4.3 Hallmark of Cancer No. 3

##### 3.4.3.1 Expression of Resisting Cell Death Hallmark and OL Malignant Transformation Probability

The risk ratio associated with the expression of antiapoptotic biomarkers was 0.94 (95% CI = 0.43–2.03, *p* = 0.87) (8 studies/625 cases).

##### 3.4.3.2 Differential Expression in OL

The estimated PP for antiapoptotic biomarkers was 69.11% (95% CI = 39.36–92.37) (8 studies/625 cases).

##### 3.4.3.3 Comparison Between Expression in OL vs. Healthy Controls

OL cases showed a higher frequency for antiapoptotic biomarkers, although significant differences were not reached (OR = 6.17, 95% CI = 0.85–44.84, *p* = 0.07) (2 studies/44 cases).

#### 3.4.4 Hallmark of Cancer No. 4

##### 3.4.4.1 Expression of Enabling Replicative Immortality Hallmark of Cancer and OL Malignant Transformation Probability

The RR associated with the expression of prosurvival/immortalization biomarkers was 3.74 (95% CI = 1.94–7.21, *p* < 0.001), although only two studies (244 cases) were published on this topic.

##### 3.4.4.2 Differential Expression in OL

The estimated PP for prosurvival/immortalization biomarkers was 41.26% (95% CI = 24.56–59.04) (2 studies/244 cases).

##### 3.4.4.3 Comparison Between Expression in OL vs. Healthy Controls

Primary‐level studies were not identified on this cancer hallmark, so meta‐analysis could not be performed.

#### 3.4.5 Hallmark of Cancer No. 5

##### 3.4.5.1 Expression of Inducing Angiogenesis Hallmark and OL Malignant Transformation Probability

The RR associated with the expression of proangiogenic biomarkers was 3.74 (95% CI = 1.79–7.82, *p* < 0.001) (2 studies/280 cases).

##### 3.4.5.2 Differential Expression in OL

The estimated PP for proangiogenic biomarkers was 43.80% (95% CI = 32.41–55.52) (2 studies/280 cases).

##### 3.4.5.3 Comparison Between Expression in OL vs. Healthy Controls

Primary‐level studies were not identified on this cancer hallmark, so meta‐analysis could not be performed.

#### 3.4.6 Hallmark of Cancer No. 6

##### 3.4.6.1 Expression of Activating Invasion and Metastasis Hallmark and OL Malignant Transformation Probability

There was a significant association between proinvasive biomarkers and OL malignant transformation (RR = 3.43, 95% CI = 2.67–4.40, *p* < 0.001), from a large sample size (18 studies/1602 cases).

##### 3.4.6.2 Differential Expression in OL

The estimated PP for proinvasive biomarkers was 37.30% (95% CI = 28.21–46.86) (18 studies/1602 cases).

##### 3.4.6.3 Comparison Between Expression in OL vs. Healthy Controls

OL cases showed a higher frequency for pro‐invasive biomarkers, but it did not reach statistical significance (OR = 2.20, 95% CI = 0.39–12.42, *p* = 0.37) (6 studies/674 cases).

#### 3.4.7 Hallmark of Cancer No.7

##### 3.4.7.1 Expression of Avoiding Immune Destruction Hallmark and OL Malignant Transformation Probability

The magnitude of association between the expression of antitumor arrest biomarkers and malignant transformation showed significant differences (RR = 3.65, 95% CI = 1.87–7.13, *p* < 0.001) (7 studies/810 cases).

##### 3.4.7.2 Differential Expression in OL

The estimated PP for antitumor arrest biomarkers was 35.77% (95% CI = 24.66–47.69) (7 studies/810 cases).

##### 3.4.7.3 Comparison Between Expression in OL vs. Healthy Controls

Primary‐level studies were not identified on this cancer hallmark, so meta‐analysis could not be performed.

#### 3.4.8 Hallmark of Cancer No. 8

##### 3.4.8.1 Expression of Deregulating Cellular Energetics Hallmark and OL Malignant Transformation Probability

The risk ratio associated with the expression of enhancing tumor acidosis biomarkers was 4.57 (95% CI = 0.99–21.03, *p* = 0.051), although only a single study was analyzed (160 cases).

##### 3.4.8.2 Differential Expression in OL

The estimated PP for enhancing tumor acidosis biomarkers was 42.50% (95% CI = 34.92–50.21) (1 study/160 cases).

##### 3.4.8.3 Comparison Between Expression in OL vs. Healthy Controls

OL cases showed a significantly higher frequency for enhancing tumor acidosis biomarkers (OR = 27.40, 95% CI = 1.62–462.61, *p* = 0.02) (1 study/178 cases).

#### 3.4.9 Hallmark of Cancer No. 9

##### 3.4.9.1 Expression of Genome Instability Hallmark and OL Malignant Transformation Probability

The RR associated with the expression of DNA instability biomarkers was 8.14 (95% CI = 4.07–16.29, *p* = 0.009) (1 study/98 cases). In contrast, the expression of DNA damage repair biomarkers resulted in a RR of 4.31 (95% CI = 1.44–12.84, *p* < 0.001) (7 studies/506 cases).

##### 3.4.9.2 Differential Expression in OL

The estimated PP for DNA instability biomarkers was 41.84% (95% CI = 32.22–51.77) (1 study/98 cases), while for DNA damage repair biomarkers, the PP was 54.15% (95% CI = 25.19–81.69) (7 studies/506 cases).

##### 3.4.9.3 Comparison Between Expression in OL vs. Healthy Controls

For DNA instability biomarkers, OL cases showed a significantly higher frequency (OR = 36.81, 95% CI = 2.18–621.98, *p* = 0.01) (1 study/123 cases). For DNA damage repair biomarkers, OL cases also showed a higher frequency of expression (OR = 3.24, 95% CI = 1.38–7.60, *p* = 0.007) (4 studies/246 cases).

#### 3.4.10 Hallmark of Cancer No. 10

##### 3.4.10.1 Expression of Tumor‐Promoting Inflammation Hallmark and OL Malignant Transformation Probability

There was a significant association between proinflammatory biomarkers and OL malignant transformation (RR = 2.32, 95% CI = 1.63–3.30, *p* < 0.001) (5 studies/523 cases).

##### 3.4.10.2 Differential Expression in OL

The estimated PP for tumor‐promoting inflammation was 42.50% (95% CI = 27.17–58.59) (5 studies/523 cases).

##### 3.4.10.3 Comparison Between Expression in OL vs. Healthy Controls

OL cases showed a significantly higher frequency for proinflammatory biomarkers (OR = 11.23, 95% CI = 0.66–190.89, *p* = 0.09) (1 study/178 cases).

#### 3.4.11 Meta‐Analysis of Subgroups Stratified by Geographical Area

When stratifying the meta‐analyses by geographic area, inter‐subgroup *p*‐values were generally nonsignificant across most hallmarks of cancer. Only a few analyses showed *p*‐values < 0.05 (i.e., hallmark no. 3: resisting cell death, and hallmark no. 7: avoiding immune destruction), but these corresponded to small subgroups with high heterogeneity, suggesting that such differences are likely due to random variation rather than true geographic effects. In contrast, the largest and most robust datasets (including hallmark no. 1: sustaining proliferative signaling, hallmark no. 2: evading growth suppressors, and hallmark no. 6: activating invasion and metastasis) displayed consistent relative risks, PPs, and ORs between regions. This suggests that, although the expression of individual biomarkers may vary by region, the global predictive value of the cancer hallmarks framework appears to be stable worldwide. These results are summarized in Table S3 and detailed in Figures S31–S57.

#### 3.4.12 Unspecified

Finally, it was determined that a number of biomarkers should be classified as “unspecified” and thus were not assigned to any of the hallmarks previously mentioned (Table S4) as a consequence of their marked pleiotropism or their oncogenic‐protective activity depending on their upregulation or downregulation. Consequently, these biomarkers were not meta‐analyzed within any specific hallmark of cancer in samples of oral mucosa from patients with OL.

### 3.5 Analysis of Small‐Study Effects

In order to analyze the reliability and robustness of the meta‐analyses, we carried out small‐study effects analyses via the inspection of funnel plots (Figures S28–S30) and through statistical tests. The presence of biases—e.g., publication bias—could be potentially ruled out (hallmark no. 1 [pEgger = 0.22]; hallmark no. 2 [pEgger = 0.75]; hallmark no. 6 [pEgger = 0.31]). The remaining hallmarks were not considered in this statistical analysis, as a sample size lower than 10 primary‐level studies was included in their corresponding meta‐analyses.

## 4 Discussion

The most consistent evidence derived from our meta‐analysis on the expression of hallmarks of cancer in malignant vs. nonmalignant OL (60 primary‐level studies, 9758 leukoplakias) indicates that the probability of developing oral cancer in this OPMD is significantly increased in those leukoplakias that express markers denoting an acquired capacity to maintain a sustained proliferative state (hallmark 1), capacity to activate mechanisms of invasion and metastasis (hallmark 6), and capacity to evade immune‐mediated destruction (hallmark 7). As will be seen, other hallmarks are also significantly overexpressed in malignant OL, although evidence is less robust, essentially as a consequence of the small number of primary‐level studies published for these hallmarks.

The ability of OLs to proliferate in a sustained manner (hallmark 1: sustaining proliferative signaling) strongly indicates its probability of malignant transformation (Hanahan and Weinberg 2000, 2011; Pimenta‐Barros et al. 2025). Our results show that 56.30% of OLs overexpress proliferative markers, while this only occurs in 15.49% of healthy controls (results in Table 2 and Table S2); furthermore, our meta‐analysis demonstrates that OLs present a significantly higher probability of proliferation than healthy oral mucosa (OR = 7.70, 95% CI = 2.22–26.65, *p* = 0.001). Hyperproliferative leukoplakias have almost twice the increased malignant transformation *probability* to lower proliferation rates (RR = 1.92, *p* < 0.001). The above findings indicate, in our opinion, that the hyperproliferative state behaves as a driver for malignant transformation in OL, presumably due to the fact that a higher frequency of cell divisions is associated with a higher risk of genetic aberrations and the acquisition of oncogenic advantages (Hanahan and Weinberg 2000, 2011). This concept is specifically gathered in hallmark 9 (genome instability and mutation), which makes reference to the genome of hyperproliferative cells becoming unstable and prone to developing oncogenic aberrations (Hanahan and Weinberg 2000, 2011). The information available in our meta‐analysis regarding hallmark 9 derives only from one study on 98 patients analyzing the malignization of OLs expressing genomic instability markers. Although the evidence is very limited and research should continue, this study indicates that the probability of malignancy of these leukoplakias is significant and 8 times higher than for leukoplakias that do not express genomic instability (RR = 8.14, 95% CI = 4.07–16.29, *p* = 0.009). Related to the concept of genomic instability is the expression of genes and proteins involved in DNA damage repair: genomic instability and the damage it causes would lead to a reparative response. In this regard, the available evidence is more robust: 7 cohort studies report that 54.15% out of the total number of leukoplakias express DNA repair proteins; however, our results also indicate that DNA repair mechanisms are insufficient to prevent malignant transformation and behave as risk markers for the progression to cancer of OL (RR = 4.31, 95% CI = 1.44–12.84, *p* < 0.001). Thus, OLs that activate DNA repair mechanisms “paradoxically” become more malignant, probably as a consequence of the fact that these leukoplakias carry severe oncogenic alterations with a high probability of evolving into oral cancer (Hanahan and Weinberg 2000, 2011).

Another remarkable result of our meta‐analysis concerns the ability of epithelial cells in OL to activate mechanisms of invasion and metastasis (hallmark 6) (Hanahan and Weinberg 2000, 2011); 37.30% of OLs express invasion and metastasis markers (95% CI = 28.21–46.86), and these leukoplakias become malignant 3.43 times more than those that do not express these markers. These results are robust and derived from a considerable number of primary‐level studies (18 studies, 1602 cases) (RR = 3.43, 95% CI = 2.67–4.40, *p* < 0.001). All proteins included in this hallmark (Table S4) activate the epithelial–mesenchymal transition (EMT) phenomenon (Nieto et al. 2016), a regulatory program by which epithelial cells acquire a spindle‐shaped mesenchymal morphology. These cells suppress epithelial traits, particularly the expression of E‐cadherin, and instead begin expressing mesenchymal markers like vimentin, alongside developing apoptosis resistance and increased migratory capacity. EMT is therefore considered to be responsible for the development of the final cancer‐defining cellular event, invasion, the driving force for the neoplasm's spread (Bakir et al. 2020). Our meta‐analysis demonstrates that OLs at increased probability of cancer development activate early EMT mechanisms. Podoplanin is one of the most studied markers in this regard, with primary‐level studies reporting that a high percentage of OL (46.91%; 7 cohort studies, 558 patients) overexpress this cell motility activating protein. E‐cadherin (Lorenzo‐Pouso et al. 2023; Peinado et al. 2004) and β‐catenin (González‐Moles et al. 2014; Ramos‐García and González‐Moles 2022) are also recognized as adhesion molecules whose loss of expression is a requirement for the development of the EMT phenomenon, although, unfortunately, the primary‐level studies performed on them in OL are heterogeneous and do not offer precise information that would allow their inclusion in the meta‐analysis.

OLs activate molecular mechanisms to evade immune destruction (hallmark 7) (Hanahan and Weinberg 2000, 2011). Our meta‐analysis indicates with high evidence (7 cohort studies, 810 cases) that 35.77% of OLs (95% CI = 24.66–47.69) overexpress proteins involved in mechanisms of evasion of the antitumor immune response, and these leukoplakias present a probability of malignization 3.65 times higher than leukoplakias that do not develop this type of molecular mechanism (RR = 3.65, 95% CI = 1.87–7.13, *p* < 0.001). Among the best studied proteins in this hallmark are PD‐L1 and its receptor PD‐1 (Lenouvel et al. 2020; Strati et al. 2025). Tumor cells and malignant cells, through PD‐L1/PD‐1 expression, should be able to avoid destruction mediated by the antitumor immune response. Overexpression of PD‐L1 proteins in premalignant and malignant epithelial cells activates T‐lymphocyte apoptosis after binding to their PD‐1 receptors expressed on the lymphocyte membrane (Doroshow et al. 2021). Primary level studies on PD‐L1/PD‐1 overexpression in OL (3 studies, 364 patients) indicate that 23.73% of cases overexpress these proteins, indicating that PD‐L1 upregulation in OL is a protective mechanism of oral epithelial cells against immune aggression and a marker of poor prognosis in this OPMD.

Our study presents some limitations that should be discussed. First, several of the meta‐analyses showed a degree of heterogeneity, which is common, particularly in meta‐analyses of proportions. This was expected and anticipated in the study protocol, and a random‐effects model was applied in all analyses to account for this variability. Second, there was a lack of available evidence for some canonical and emerging hallmarks of cancer, such as resistance to cell death and deregulated cellular energetics. However, we consider this limitation to be inherent to the existing body of primary research, rather than a limitation intrinsic to our meta‐analysis. Third, OL is a complex and heterogeneous pathology whose malignant transformation does not always occur and may follow diverse pathogenetic pathways in different cases. The results obtained in this study confirm once again the existence of a complex carcinomatous process in the malignancy of OL, in which some alterations appear in a significant proportion of cases, but not always and in all of them. Recognizing these gaps may help guide and refine future research directions. Finally, we would like to emphasize some strengths of this study: firstly, its originality, as this is the first meta‐analysis to date investigating the association between the expression of hallmarks of cancer and OL malignant transformation; and secondly, its methodological quality, supported by the strict inclusion of longitudinal studies with follow‐up, which provide stronger and more reliable evidence. This is particularly relevant since most published primary studies harbor a cross‐sectional nature. Therefore, our meta‐analysis allows for a better assessment of causality and offers conclusions that are more robust and, therefore, closer to reality. Previous relevant systematic reviews, including those from the WHO Collaborating Center for Oral Cancer (Luis Monteiro, Mello, and Warnakulasuriya 2021) and the World Workshop on Oral Medicine group (Villa et al. 2019), have also identified significant biomarkers for OLs' malignant transformation, such as promising emerging proteins, like podoplanin, along with classical tumor suppressors like p53 and p27, and genomic alterations such as aneuploidy and loss of heterozygosity (Monteiro, Silva, et al. 2021; Villa et al. 2019). Our meta‐analysis reinforces the predictive value of these well‐recognized biomarkers and adds quantitative evidence that several hallmarks of cancer are consistently overexpressed during the malignant transformation of OL. These findings support that molecular pathways driving malignant transformation may be broader and more interconnected than previously recognized.

In conclusion, our meta‐analysis indicates on the basis of evidence that OL malignant transformation is significantly increased in hyperproliferative lesions, which develop mesenchymal epithelial transition phenomenon and molecular mechanisms to evade immune response. Consequently, an immunohistochemical analysis of proliferation markers (i.e., Ki‐67), markers of mesenchymal epithelial transition (i.e., podoplanin), and markers of antitumor immune response evasion (i.e., PD‐L1) could, jointly with other clinical (nonhomogeneous leukoplakias, of larger size, localized on the tongue, in smoking patients) and pathological (presence and severity of epithelial dysplasia) markers (Pimenta‐Barros et al. 2025), help to more precisely assess the malignant transformation of OL and to establish specific prevention and treatment strategies.

## Author Contributions

**I. González‐Ruiz:** conceptualization, investigation, writing – original draft, methodology, validation, visualization, writing – review and editing, software, formal analysis, data curation, supervision. **P. Ramos‐García:** conceptualization, investigation, writing – original draft, methodology, validation, visualization, writing – review and editing, software, formal analysis, data curation, supervision. **H. Boujemaoui‐Boulaghmoudi:** conceptualization, investigation, writing – original draft, methodology, validation, visualization, writing – review and editing, software, formal analysis, data curation, supervision. **N. Mjouel‐Boutaleb:** conceptualization, investigation, writing – original draft, methodology, validation, visualization, writing – review and editing, software, formal analysis, data curation, supervision. **M. A. González‐Moles:** conceptualization, investigation, writing – original draft, methodology, validation, visualization, writing – review and editing, software, formal analysis, data curation, supervision.

## Conflicts of Interest

The authors declare no conflicts of interest.

## Supporting information

odi70106‐sup‐0001‐DataS1.docx

## Data Availability

Data is contained within the article or S1.
