# Supplementary material for: Cancer Hallmarks Expression in Oral Leukoplakia: Systematic Review and Meta‐Analysis
Source: Oral Dis. 2025 Sep 30;32(2):322–37. doi: 10.1111/odi.70106 (PMC13077026; doi:10.1111/odi.70106)
Supplement: Supplementary file 1 — odi70106‐sup‐0001‐DataS1.docx [file ODI-32-322-s001.docx]

**Supplementary information to the manuscript**

**Cancer hallmarks expression in oral leukoplakia: systematic review and meta-analysis**

**Table of contents**

1. Search strategy. 3

2. Descriptive characteristics of the study sample (extended version) 4

3. Meta-analysis on the expression of hallmarks of cancer in oral leukoplakia 25

4. Analysis of small-study effects. 52

5. Meta-analysis on the expression of hallmarks of cancer in oral leukoplakia stratified by geographical area 55

6. Biomarkers roles in the context of hallmarks of cancer 86

7. List of included studies 102

8. List of excluded studies (with reasons). 108

**1. Search strategy**

| **Database** | **Query/Search Strategy** | **Results/ Items found** | **Search time limits** |
| --- | --- | --- | --- |
| MEDLINE | ("Leukoplakia, Oral”[Mesh] OR ("leukoplakia"[All Fields] AND ("Mouth"[Mesh] OR “oral”[All Fields]))) AND (malign* OR premalign* OR precancer* OR “potentially malignant disorder” OR “cancer"[All Fields] OR "Carcinoma, Squamous Cell"[MeSH] OR "squamous cell carcinoma"[All Fields] OR "oscc"[All Fields] OR “transformation” [All Fields] OR "risk"[All Fields] OR "progression"[All Fields]) | 4,189 | September,  2024 |
| Embase | (('leukoplakia'/exp OR 'leukoplakia') AND ('mouth'/exp OR 'oral')) AND (‘malign*’ OR ‘premalign*’ OR 'potentially malignant disorder' OR 'precancer'/exp OR 'precancer' OR 'cancer'/exp OR 'cancer' OR 'squamous cell carcinoma'/exp OR 'squamous cell carcinoma' OR 'oscc' OR 'transformation'/exp OR 'transformation' OR 'risk'/exp OR 'risk' OR 'progression') | 7,716 | September,  2024 |
| Web of Science | TS=(leukoplakia AND (oral OR mouth)) AND TS=(malign* or premalign* or potentially malignant disorder or precancer or cancer or squamous cell carcinoma or oscc or transformation or risk or progression) | 3,611 | September,  2024 |
| Scopus | TITLE-ABS-KEY ((“leukoplakia” AND (“oral” OR “mouth”)) AND ("malign*" OR "premalign*" OR "potentially malignant disorder" OR "precancer" OR "cancer" OR "squamous cell carcinoma" OR "oscc" OR "transformation" OR "risk" OR "progression")) | 5,806 | September,  2024 |
| Total | 21,322 | | |

**Table S1.** Search strategy for each database, number of results, and execution date.

**2. Descriptive characteristics of the study sample (extended version)**

**Table S2.** Descriptive characteristics of the study sample

| **Study**  **(year)** | **Country**  **(language)** | **Design** | **Recruitment period** | **Follow up, m,**  **mean±SD**  **(range)** | **Patients with oral leukoplakia and progression to cancer** | | | | | | | | | **Analysis of biomarkers expression** | | | | |
| --- | --- | --- | --- | --- | --- | --- | --- | --- | --- | --- | --- | --- | --- | --- | --- | --- | --- | --- |
|  |  |  |  |  | **Sample**  **Size,**  **n** | **Sex, M/F,**  **n(%);**  **Age, y (mean±SD,**  **range)** | **Tobacco,**  **n (%)** | **Alcohol,**  **n (%)** | **Affected**  **oral**  **subsites** | **Clinical subclassification,**  **n** | **Epithelial dysplasia, n** | **Cancer development, n** | **Healthy controls,**  **n** | **Methods** | **Antibody (dilution, incubation time, temperature)** | **IHC pattern** | **IHQ cutoff point (%)** | **Biomarker**  **positivity:**  **n(%)** |
| Chen *et al.* (2023) | South Korea  (English) | R | 1996 -2011 | 125.7±NR | 103 | M=59 (57.3)  F=44 (42.7)  Median: 56  (NR, 24-84) | NR | NR | Bm:28  Tongue:30  Gingiva:45 | NR | No ED:49  ED:54 | 21 | NR | IHC | TIPE2:  clone ab133616  (1:1000, NR,NR) | nuclear-  cytoplasmic | NR | TIPE2: 40 (38.83) |
| de Villalaín *et al.* (2023) | Spain  (English) | R | 2000 -2005 | NR  (60-NR) | 60 | M=30 (50)  F=30 (50)  (60.17±15.3, 18-87) | Former smokers:  13 (21.7) | Former  drinkers:  10 (16.7) | NR | NR | No ED:47  ED: 13  mild:5  moderate: 3  severe:5 | 15 | NR | IHC | Ki67:  clone MIB-1  (NR, RT,NR)  p16:  clone E6H4  (NR, RT, NR)  DcR2:  NR (1:1000,RT,NR)  DEC1:  NR  (1:200,RT,NR ) | Ki-67:  nuclear  p16:  nuclear  DcR2:  nuclear  Dec1:  nuclear-  cytoplasmic | Ki-67: NR  p16:  10  DcR2: 50  Dec 1:  10 | Ki67:  38 (63)  p16:  8 (13)  DcR2:  9 (15)  Dec1 (nuclear):  50 (87.72)  Dec1 (cytoplasmic): 27 (47.37) |
| Mariz *et al.* (2023) | Brazil  (English) | R | NR | 40.1±NR | 30 | M=18 (60)  F=12 (40)  (52.7± NR, 24–74) | Yes: 9  No: 4  Former smoker: 8  missing: 9 | Yes: 6  No: 10  Former drinker: 2  missing: 12 | Tongue: 16  Others: 14 | Non-  homogeneous: 16  Homogeneous: 14 | No ED: 0  ED: 30  mild: 14  moderate:  8  severe: 8 | 6 | NR | IHC | FGFR1:  NR  (1:50, 2h, RT) | nuclear | NR | NR |
| Wils *et al.*  (2023) | The Netherlands  (English) | R | 1997- 2022 | 63±NR  (12-300) | 176  missing: 69 | M= 60 (34.09)  F= 116 (65.91)  Median: 62  (NR, 29-97) | NR | NR | Bm: 15  Tongue: 60  Gingiva: 32  Lip: 1  Palate: 15  Fom: 30  Others: 23 | NR | No ED:117  ED: 59  mild :35  moderate:  11  severe:13 | 33 | NR | IHC | CK13:  clone KS-1A3 (1:50, 24min, 100ºC)  CK17:  clone SP95  ( 1:50, 32min, 95ºC) | NR | NR | CK13:  24 (28.56)  CK17:  86 (57.72) |
| Xu *et al.* (2023) | China  (English) | R | 2008 - 2018 | 148±88  (36 - 410) | 82 | M=43 (52.44)  F=39 (47.56)  NR | NR | NR | Bm: 22  Tongue:45  Others: 15 | NR | No ED: NR  ED: NR  Low grade: 57  High grade: 25 | 2 | NR | IHC | PD-1:  NR  (1:200, overnight, 4ºC)  PD-L1:  NR  (1:200, overnight, 4ºC)  CD3:  NR  (1:200, overnight, 4ºC)  CD8:  NR  (1:200, overnight, 4ºC)  Foxp3:  NR  (1:200, overnight, 4ºC) | PD-1:  NR  PD-L1:  cytoplasmic-membranous  CD3:  NR  CD8:  NR  Foxp3:  NR | PD-1: >17 positive cells  PD-L1:1  CD3:  >60 positive cells  CD8: >40 positive cells  Foxp3: >42 positive cells | PD-1:  25 (30.49)  PD-L1:  10 (12.20)  CD3:  34 (41.46)  CD8:  31 (37.80)  Foxp3:  29 (35.37) |
| Monteiro *et al.*  (2022) | Portugal  (English) | R | 1995-2006 | 32.4±29  (2 - 120) | 52 | M=38 (73.08)  F=14 (26.92)  (57.55±16.53)  (20-88) | Current:  12 (23.08)  Former or never: 22 (42.31)  missing: 17 | Current: 11 (21.15)  Former or never: 24 (46.15)  missing: 17 | Bm: 12  Tongue: 23  Gingiva: 8  Lip: 4  Palate: 1  Rmt: 2  Fom: 2 | Non-  homogeneous:  10  Homogeneous: 17  missing: 25 | No ED: NR  ED: NR  Low-grade: 41  High-grade: 11 | 6 | 12 | IHC | CD44v6:  clone VFF-7 (1:120, NR, NR)  BSG:  clone AB1843  (1:30, NR, NR)  EGFR:  clone EGFR.25  (1:100, NR, NR)  podoplanin:  clone D2-40  (1:150, NR, NR)  p53:  clone NCLp53 DO7  (1:20, NR, NR)  p63:  clone 7JUL  (1:25, NR, NR)  p73:  clone 24  (1:25, NR, NR)  p16:  OA315 clone  (NR, NR, NR) | CD44v6: membranous  BSG:  membranou  EGFR:  membranou  podoplanin:  membranous  p53:  nuclear  p63:  nuclear  p73:  nuclear  p16:  nuclear-cytoplasmic | Labelling index (intensity x cell count) | CD44v6:  45 (95.75)  BSG:  24 (54.55)  EGFR:  43 (95.56)  podoplanin:  5 (12.82)  p53:  25 (64.10)  p63:  42 (97.67)  p73:  30 (73.17)  p16:  8 (20) |
| Tarle *et al.*  (2022) | Croatia  (English) | R | 2010 - 2014 | NR | 31 | M=12 (38.71)  F=19 (61.29)  (61.77±15.70)  (17-96) | 22  (70.97) | 10  (32.26) | Bm:3  Tongue:15  Gingiva:3  Palate:2  Rmt: 1  Fom:6  Other:1 | NR | No ED: 15  Mild:13  Moderate:3  Severe:0 | 9 | NR | IHC | EGFR:  EGFR-1 clone (1:25, 90 min, NR)  ABCG2:  B-1 clone (1:25, 90 min, NR) | EGFR: nuclear  ABCG2:  undefined | EGFR:1  ABCG2: 1 | EGFR:  17 (54.84)  ABCG2:  17 (54.84) |
| Wu *et al.*  (2022) | China  (Chinese) | R | 1996 - 2018 | 114  (25 - 285) | 109 | M=51 (46.79)  F= 58 (53.21)  (NR)  (18 - 74) | 29 (26.61) | 15  (13.76) | Low-risk areas (bm, back of tongue, lips, gingiva, palate): 68  high-risk areas (fom, lateral and ventral tongue): 41 | NR | No ED: 0  ED: 109  mild: 84  moderate: 0  severe: 25 | 25 | NR | IHC | BMI-1:  NR  (1:200, overnight, 4ºC) | Nuclear | Labelling index (Intensity) | BMI-1:  55 (50.46) |
| Monteiro *et al.*  (2021) | Portugal  (English) | R | 1995 -2006 | 32.4±29  (2-120) | 52 | M= 38  (73.08)  F= 14  (26.92)  (57.55±16.53, 20-88) | Current:  12 (23.08)  Former or never: 22 (42.31)  missing: 32 | Current: 11 (21.15)  Former or never: 24 (46.15)  missing: 29 | Bm: 12  Tongue: 23  Gingiva: 8  Lip: 4  Palate: 1  Rmt: 2  Fom: 2 | Non-  homogeneous:  10  Homogeneous: 17  missing: 25 | No ED: NR  ED: NR  low grade: 41  high grade:  11 | 6 | 12 | IHC | BubR1:  clone 9  (1:150, 1h, NR)  Mad2:  clone 48  (1:75, 1h, NR)  SPINDLY:  clone HPA044700 (1:500, 1h,NR)  ki-67:  clone MIB-1 (1:100,1h,NR)  BUB3:  clone EPR5319  (1:500, 1h,NR) | BubR1 and Mad2:  nuclear-cytoplasmic  BUB3 and ki-67: nuclear  SPINDLY: cytoplasmic | All Labelling index (intensity x cell count) | BubR1:  5 (9.62)  Mad2:  19 (.39.58)  SPINDLY:  24 (48.00)  ki-67:  19 (37.25)  BUB3:  40 (83.33) |
| Yagyuu *et al.*  (2021) | Japan  (English) | R | 1995 - 2015 | 55.4±58.6  (0.02-219.4) | 200 | M=82 (41)  F=118 (59)  (65.07±12.61)  (26.15 - 89.44) | 59  (29.5) | Current/ former:79  (39.5)  Never: 121 | Tongue: 67  Others: 133 | NR | No ED: 0  ED:200  Low-grade (mild): 132  High-grade (moderate and severe): 68 | 15 | NR | IHC | PD-L1:  NR  (1:50, 60min, rt) | Membranous | Labelling index (intensity x cell count) | PD-L1:  57 (28.5) |
| Lv *et al.*  (2020) | China  (English) | R | 2010 - 2018 | NR  (5 - 75) | 79 | NR | NR | NR | NR | NR | NR | 30 | NR | IHC | PTHrP:  NR  (1:150, overnight, 4°C) | Undefined | Labelling index (intensity x cell count) | PTHrP:  54 (68.35) |
| Mao *et al.* (2020) | China  (English) | R | 2014- 2018 | NR | 59 | M=55(93.22)  F=4(6.78)  (49.28) | NR | NR | Bm: 13  Tongue: 46 | NR | No ED: 12  ED:  mild: 21  moderate and severe:  14 | 12 | NR | IHC | Dec1:  clone ab70723  (1:1000;overnight, 4°C) | nuclear and cytoplasmic | 5 | Dec1:  47 (79.66) |
| Mondal *et al.* (2020) | India (English) | P | 2013- 2016 | (X-48) | 140 | NR | NR | NR | NR | NR | No ED: 0  ED: 126  mild: 64  moderate: 43  severe:19 | 14 | 20 | IHC | NR | nuclear | Labelling index (intensity x cell count)  5 | ki-67:  103 (73.57) |
| Sakata *et al.* (2020) | Japan  (English) | R | 2000 - 2013 | 37.26 ± NR  (0.4 - 202.39) | 165 | M=89 (53.94)  F=76 (46.06)  M63.6  (19-87) | NR | NR | Bm:21  Tongue:62  Gingiva:68  Others: 14 | NR | No ED: 116  ED:49  mild: 36  moderate and severe: 13 | 25 | NR | IHC | Foxp3:  259D clone  (1:100, overnight, 4°C) | NR | ≥ 15 positive cells | Foxp3:  72 (43.64) |
| Shigeoka *et al.* (2020) | Japan  (English) | R | 2009 - 2016 | NR | 26 | M=14 (53.8)  F= 12 (46.2)  (62.5±NR)  M66.0  (28-89) | Never: 15  Past and present: 11 (42.31) | Never: 15  Past and present: 11 (42.31) | Tongue: 26 | Non-homogeneous: 14  Homogeneous: 12 | No ED: NR  ED: NR  Low degree (normal, mild):8  High degree (moderate, severe, CIS):18 | 9 | NR | IHC | Ki-67:  clone MIB-1  (1:100, NR, NR)  CK13:  clone KS-1A3  (1:50, NR, NR)  CK17:  clone E3  (1:40, NR, NR) | NR | ki-67: NR  CK13: Labelling index (intensity)  CK17:Labelling index (intensity) | ki-67:  19 (100)  CK13:  2 (22.22)  CK17:  10 (66.67) |
| Weber *et al.* (2020) | Germany  (English) | P | 1994- 2014 | NR | 103 | M= 60 (58.25)  F= 43 (41.75)  (57.05±12.72, 23-92) | NR | NR | NR | NR | No ED: 69  ED:  mild: 22  moderate: 8 severe: 4 | 49 | 49 | IHC | CD68:  clone KP1  (1:3000, NR, NR)  CD163:  clone EP1347y (1:100, NR,NR)  CD11c:  clone NCL-CD163 (1:100, NR.NR) | cytoplasmic | Labelling index (intensity x cell count) | CD68:  70 (67.96)  CD163:  63 (65.63)  CD11c:  42 (43.75) |
| de Vicente *et al.* (2019) | Spain  (English) | R | 2000-2005 | 85.47±44.41  (4-252)  Median:  75 | 55 | M= 26 (47.27)  F= 29 (52.73)  (62.61±12.56, 39-83) | Smoker: 10  Non-  smoker: 21  missing: 24 | Drinker: 4  Non-  drinker: 27  missing:  24 | Bm: 13  Tongue: 20  Gingiva: 14  Palate: 2  Fom: 2  Others: 4 | NR | No ED: 0  ED: 55  low grade: 42  high grade: 13 | 12 | NR | IHC | SOX2:  clone AB5603 (1:1000, NR,RT) | Nuclear | 1 | SOX2:  16 (29.09) |
| de Vicente *et al.* (2019) | Spain  (English) | R | 2000-2005 | 85.47±44.41  (4-252)  Median: 75 | 55 | M= 26 (47.27)  F= 29 (52.73)  (62.61±12.56, 39-83) | smoker: 10  Non-  smoker: 21  missing: 24 | drinker: 4  Non-  drinker: 27  missing:  24 | Bm: 13  Tongue: 20  Gingiva: 14  Palate: 2  Fom: 2  Others: 4 | NR | No ED: 0  ED: 55  low grade: 42  high grade: 13 | 12 | NR | IHC | NANOG:  clone D73G4  (1:200, NR, RT) | Nuclear | 1 | NANOG  (nuclear):  2 (3.64)  NANOG (cytoplasmatic):  9 (16.36) |
| Sundberg *et al.* (2019) | Sweden  (English) | P | 2011 -2017 | NR | 81 | M= 39 (48.15)  F= 42 (51.85)  (NR) | Yes: 25  No: 55  missing: 1 | Several times per week: 12 (14.81)  Once a week: 29 (35.80)  Never/rarely: 34 (41.98)  missing: 6 | Bm: 17  Tongue: 25  Gingiva: 27  Lip: 3  Palate: 4  Fom: 5 | Non-homogeneous: 52  Homogeneous: 22 | No ED: 63  ED: 14  mild: 13  moderate: 3  severe: 1 | 4 | NR | IHC | p16: clone E6H4 (NR,NR,NR) | NR | 10 | p16: 13 (17.57)  missing: 7 |
| Wu *et al.* (2019) | China  (English) | R | 2000 - 2015 | 71.74 ±NR  (6 - NR) | 76 | M=25 (32.89)  F= 51 (67.11)  (NR) | Smoker; 18 (23.68)  Non-smoker: 58 (76.63) | Drinker: 10  (13.16)  Non-drinker: 66 (86.84) | Bm:14  Tongue:59  Gingiva:3 | NR | No ED: 0  ED: 76  mild: 26  moderate: 39  severe: 11 | 41 | NR | IHC | p16:  NR  (1:!50, overnight, 4°C) | nuclear-cytoplasmic | 70 | p16:  29 (38.16) |
| D’souza *et al.* (2018) | India  (English) | NR | NR | NR | 30 | M= 22 (73.33)  F= 8 (26.67)  (49.86)  (18 - 70) | Smoking:6 (20)  Tobacco chewing: 21 (70) | 1 (3.33) | Bm: 27  Tongue:2  Gingiva:1 | Non-homogeneous: 15  Homogeneous: 15 | No ED: 9  ED: 20  mild: 9  moderate: 6  severe: 5 | 1 | 15 | IHC | podoplanin:  D2-40 clone  (NR, NR, NR) | cytoplasmic | 1 | podoplanin:  23 (76.67) |
| Ding *et al.* (2018) | China  (English) | R | 1996-2010 | 74.18±NR | 78 | M= 33 (42.31)  F= 45 (57.69)  (56±NR)  (27-85) | Never: 57 (73.08)  Past and present: 15 (19.23)  missing: 6 | Never: 58 (74.36)  Past and present: 15 (19.23)  missing: 5 | Tongue: 29  Others: 49 | NR | No ED: 0  ED: 78  mild-moderate:59  severe: 19 | 19 | NR | IHC | Notch1:  clone D1E11 (1:500, NR, NR) | membranous | Labelling index (intensity) | Notch1:  24 (30.77) |
| Saintigny *et al.* (2018) | (English) | P | NR | NR | 120 | M= 61 (50.83)  F= 59 (49.17)  (NR) | Current: 42  Former:  45  Never: 33 | Current: 70  Former: 10  Never: 40 | NR | NR | No ED: 76  ED: 44 | 31 | NR | IHC | Met:  clone sc-20 (1:1000, 60min, RT) | membranous-cytoplasmic | Labelling index (intensity) | Met:  60 (50.00) |
| Zhu *et al.* (2018) | China  (English) | R | 2005 -2014 | NR | 99 | M= 52 (51.48)  F= 47 (46.53)  (56.22±11.30,  31-71) | Never: 61 (61.62)  Past-present: 38 (38.38) | Never: 39 (39.40)  Past-present: 60 (60.61) | Bm: 35  Tongue: 57  Gingiva: 3  Lip: 3  Palate: 1 | NR | NR | 18 | NR | IHC | ATM:  clone ab78  (1:1000, 1h, RT)  γH2AFX:  clone ab22551  (1:200, 1h, RT) | ATM: nuclear-  cytoplasmic  γH2AFX: nuclear | 5 | ATM:  54 (53.46)  γH2AFX:  29 (28.71) |
| Habiba *et al.* (2017) | Japan  (English) | R | 2002-2012 | 42.1±34.1  (6-125)  Median: 25 | 79 | M= 25 (31.64)  F= 54 (68.35)  (70±12)  Median:72 | NR | NR | Bm: 21  Tongue: 28  Gingiva: 18  Fom: 5  Other: 7 | NR | No ED: 0  ED: 79  Low grade: 27  High grade: 52 | 37 | NR | IHC | podoplanin:  clone D2-40  (1:100, overnight, 4ºC)  ALDH1:  clone 44 (1:100, overnight, 4ºC) | cytoplasmic-membranous | NR | podoplanin:  53 (67.09)  ALDH1:  48 (60.76) |
| Nguyen *et al.* (2017) | Japan  (English) | R | NR | 20.3±NR  (0.5-158.4) | 93 | NR | NR | NR | Tongue: 93 | NR | No ED: 0  ED: 93  mild: 11  moderate: 78  severe: 4 | NR | NR | IHC | LAMC2:  clone B-2  (1: 500 overnight, 4ºC) | cytoplasmic | 1 | LAMC2:  61 (65.59) |
| Sakata *et al.* (2017) | Japan  (English) | R | 2000-2011 | 34.0±NR  (0-131) | 150 | M= 76 (50.70)  F= 74 (49.30)  Median: 62.3  (NR, 19-87) | Never: 16 (10.67)  Past and present:  15 (10.0)  missing: 119 | Never: 13 (8.67)  Past and present:  14 (9.33)  missing: 123 | Bm: 21  Tongue: 52  Gingiva: 64  Other: 13 | NR | No ED: 101  ED: 49  mild: 37  moderate-  severe: 12 | 23 | NR | IHC | DPC4:  clone H-552  (NR, overnight, 4ºC) | nuclear | 4 | DPC4:  84 (56.00) |
| Zhang *et al.* (2017) | South Korea  (English) | R | 1995 - 2010 | Median: 130  (1.5- 222) | 154 | M= 96 (62.34)  F= 58 (37.66)  (Median: 55)  (13 - 89) | NR | NR | Bm: 44  Tongue: 42  Gingiva: 68 | NR | NR | 22 | 18 | IHC | Snail:  NR  (1:500, 2h, rt)  Axin2:  NR  (1:200, 2h, rt) | Snail:  nuclear  Axin2:  cytoplasmic | 1 | Snail:  58 (37.7)  Axin2:  19 (12.3) |
| Zhang *et al.* (2017) | South Korea  (English) | R | 1994 - 200 9 | M 135 ± NR  (55.2 - 278.4) | 160 | M= 100 (62.5)  F= 60 (37.5)  (51.9, M 54) (13-89) | NR | NR | Bm: 44  Tongue: 44  Gingiva: 72 | NR | No ED: 82  ED: 78  Low grade: 54  High grade: 24 | 22 | 18 | IHC | COX2:  NR  (1:50, NR, NR)  c-Met:  NR  (1:100, NR, NR)  β-catenin:  NR  (1:100, NR, NR)  CA9:  NR  (1:100, NR, NR)  ki-67:  NR  (1:150, NR, NR)  p16:  NR  (1:100, NR, NR)  p53:  NR  (1:150, NR, NR)  podoplanin:  NR  (1:100, NR, NR)  c-jun:  NR  (1:100, NR, NR) | COX2, c-Met, β-catenin, CA9, podoplanin:  membranous-cytoplasmic  ki-67, p53, c-Jun:  nuclear  p16:nuclear-cytoplasmic | 1 | COX2:  37(23.13)  c-Met:  61(38.13)  β-catenin:  25(15.63)  CA9:  68(42.5)  ki-67:  31(19.38)  p16:  51(31.88)  p53:  29(18.13)  podoplanin:  37(23.13)  c-jun:  17(10.63) |
| Fernández-Valle *et al.* (2016) | Spain  (English) | P | 1995 - 2006 | NR | 62  (missing 3) | M= 35 (56.45)  F=27 (43.55)  (61.08±12.44)  (30-85) | No: 34 (54.84)  Yes: 21 (33.87)  Ex-smoker: 7 (11.29) | No: 48 (77.42)  Yes: 10 (16.13)  Ex-drinker: 4 (6.45) | NR | NR | No ED: 43  ED: 19  mild: 8  moderate: 7  severe/CIS: 4 | 27 | NR | IHC | KCNC4:  NR  (1:150, NR, RT) | NR | Labelling index (intensity) | KCNC4:  15 (25.42) |
| Fernández-Valle *et al.* (2016) | Spain  (English) | P | 1995 - 2006 | NR | 62  (missing 1) | M= 35 (56.45)  F=27 (43.55)  (61.08±12.44)  (30-85) | No: 34 (54.84)  Yes: 21 (33.87)  Ex-smoker: 7 (11.29) | No: 48 (77.42)  Yes: 10 (16.13)  Ex-drinker: 4 (6.45) | NR | NR | No ED: 43  ED: 19  mild: 8  moderate: 7  severe/CIS: 4 | 27 | NR | IHC | KCNH2:  NR  (1:200, NR, RT) | NR | Labelling index (intensity) | KCNH2:  22 (36.07) |
| Lima *et al.* (2016) | Brazil  (English) | R | 2005 -2011 | NR | 73 | M= 36 (49.32)  F= 36 (49.32)  (56.97±NR, 28-89)  missing: 1 | smokers:  39 (53.42)  Never-  smokers: 34 (46.58) | NR | Bm: 18  Tongue: 22  Gingiva:14  Palate: 5  Fom: 11  missing: 4 | NR | No ED: 29  ED: 44  low grade:  19  high grade:  25 | 6 | NR | IHC | c-Jun:  NR  (1:200,NR,NR)  pc-Jun:  NR  (1:100,NR,NR)  p27:  NR  (1:100,NR,NR) | nuclear | 20 | c-Jun:  46 (63.01)  pc-Jun:  22 (30.14)  p27:  27 (36.99) |
| Gissi *et al.* (2015) | Italy  (English) | R | 2006- 2013 | 42± NR  (12-90) | 77 | M= 34 (44.16)  F= 43 (55.84)  (61.6 ± 13.8, 26-95) | smokers:  35 (45.45)  no-smokers: 42 (54.45) | NR | Bm: 8  Tongue: 5  Gingiva:19  Lip:1  Palate: 3 | Non-homogeneous: 17  Homogeneous: 60 | No ED: 0  ED: 77 | 7 | NR | IHC | p53:  clone p53  (1:50, NR, NR) | nuclear | 20 | p53:  19 (24.68) |
| Nayak *et al.* (2015) | India  (English) | R | 2007-2012 | (NR-60) | 172  (missing: 129) | M= NR  F= NR  (NR) | NR | NR | NR | NR | No ED: 10  ED: 33  mild: 3  moderate: 10  severe: 20 | 7 | 52 | IHC | FGF-2:  NR  (NR, overnight, 4ºC)  FGFR-2:  NR  (NR, overnight, 4ºC)  FGFR-3:  NR  (NR, overnight, 4ºC) | cytoplasmic | Labelling index (intensity x cell count) | FGF-2:  14 (32.56)  FGFR-2:  17 (39.53)  FGFR-3:  18 (41.86) |
| Kaur *et al.* (2014) | Canada  (English) | R | 2000-2010 | 43±NR  (0-150)  Median: 36.5 | 110 | M= 59 (53.64)  F= 51 (46.36) (59±NR, 30–88) | Yes: 46  No: 42  missing: 22 | NR | Bm: 18  Tongue: 79  Lip:1  Fom:12 | NR | No ED: 0  ED: 110  mild: 58  moderate: 39  severe: 13 | 39 | NR | IHC | S100A7:  clone sc-52948 (1:500,NR,NR)  PTMA:  clone LS-B2322 (1:3500.NR.NR)  14-3-3σ:  clone ab14116-50 (1:2500,NR,NR)  14-3-3ζ:  clone IMG-6664A (1:100,NR,NR)  hnRNP:  clone ab23644 (1:5000,NR,NR)  p16:  clone sc-1661 (1:100,NR,NR) | nuclear-  cytoplasmic | Labelling index (intensity x cell count) | S100A7 (nuclear):  84 (76.36)  S100A7 (cytoplasmatic):  70 (79.09)  PTMA (nuclear):  99 (90.00)  PTMA (cytoplasmatic):  61 (55.45)  14-3-3ζ (nuclear):  59 (53.64)  14-3-3ζ (cytoplasmatic):  93 (84.55)  14-3-3σ (nuclear):  64 (58.18)  14-3-3σ (cytoplasmatic):82 (74.55)  hnRNP (nuclear):  109 (99.09)  p16 (nuclear):  85 (77.27) |
| de Vicente *et al.* (2013) | Spain (English) | R | 2000 -2005 | NR±NR  (NR-250) | 58 | M= 27 (46.55)  F= 31 (53.45)  (63.94±13.20, 39-87) | Yes: 35 (60.34)  No: 23 (39.66) | Yes: 28 (48.28)  No: 30 (51.72) | NR | NR | No ED: 0  ED: 58  mild: 43  moderate: 7  severe: 8 | 13 | NR | IHC | Podoplanin:  clone D2-40 (1:100, overnight, 4ºC) | membranous and cytoplasmic | 1 | Podoplanin:  22 (37.93) |
| Graveland *et al.* (2013) | The Netherlands  (English) | R | 2004 -2009 | NR±NR  (0-84.50) | 43 | M= 17 (39.53)  F= 26 (60.47) (61±NR, 31–90) | NR | NR | Bm: 8  Tongue: 13  Gingiva: 6  Palate: 6  Fom: 10 | NR | No ED: 31  ED: 12  mild: 4  moderate: 4  severe: 4 | 6 | NR | IHC | p53:  clone DO7  (NR, NR, NR) | nuclear | 50 | p53:  15 (34.88)  missing: 8 |
| Liu *et al.* (2013) | China  (English) | R | 1985 - 2010 | 65.63±25.90  (12 - 240) | 141 | M= 68 (48.23)  F= 73 (51.77)  (53.18±10.99, 21-79) | Never: 99 (70.21)  Past and present: 34 (24.11)  missing: 8 | Never: 118 (83.69)  Past and present: 14 (9.93)  missing: 9 | Bm: 39  Tongue: 76  Gingiva:13  Palate:8  Fom:5 | NR | No ED: 0  ED: 141  low-grade: 109  high-grade: 32 | 37 | NR | IHC | ALDH1A1:  ab52492 clone (1:250, NR, NR)  PROM1:  AC133 clone  (1:50, NR, NR) | cytoplasmic-membranous | 5 | ALDH1A1:  54 (38.30)  PROM1:  32 (22.70) |
| Ries *et al.* (2013) | Germany  (English) | R | 1997 - 2011 | 60(all) | 98 | M= 59 (60.20)  F= 39 (39.80)  (55.8±NR) | NR | NR | NR | NR | No ED: 37  ED: 60  mild: 32  moderate: 17  severe: 11 | 53 | 30 | IHC | EGFR:  M3563 clone  (1:300, NR, NR) | cytoplasmic-membranous | 44.96 | EGFR:  38 (38.78) |
| Xia *et al.* (2013) | China  (English) | R | 1996 - 2010 | 76.18±NR  (8-191) | 88 | M= 37 (42.05)  F= 51 (57.95)  (56±NR)  (27-85) | Never:64 (72.73)  Past and present: 16 (18.18)  missing: 8 (9.09) | Never:64(72.73)  Past and present: 17 (19.32)  missing:7 (7.95) | Tongue:57  Non-tongue: 31 | NR | No ED: NR  ED: NR  low-moderate: 66  high: 22 | 22 | NR | IHC | DPC4:  NR  (1:250, overnight, 4ºC) | nuclear | Labelling index (intensity x cell count) | DPC4:  43 (48.86) |
| Kreppel *et al.* (2012) | Germany  (English) | R | 2005 - 2007 | 48.0±15.8  median 48.5  (18.6 - 93.7) | 60 | M= 32 (53.33)  F= 28 (46.67)  (58.6±16.7)  median 60.8 | Yes: 28 (46.67)  Former smoker: 11 (18.33)  Never: 21 (0.35) | Never: 29 (48.33)  Occasionally: 14 (23.33)  More than once a week: 8 (13.33)  Daily: 9 (0.15) | Bm:16  Tongue:6  Gingiva:12  Palate:17  Fom:9 | NR | No ED: 31  ED:29  SIN I: 8  SIN II:12  SIN III: 9 | 12 | NR | IHC | podoplanin:  D2-40 clone  (1:100, overnight, 4ºC) | membranous | 1 | podoplanin: 41 (68.33) |
| Liu *et al.* (2012) | China  (English) | R | 1985 - 2008 | 65.78±  44.03  (12-240) | 135 | M= 64 (47.41)  F= 71 (52.59)  (53.21±11.12)  (21-79) | Never: 97 (76.38)  Past and present: 30 (23.62) | Never: 115 (91.27)  Past and present: 11 (8.73) | Bm:21  Tongue:73  Gingiva:6  Palate:4  Fom:3  NR: 28 | NR | No ED: 0  ED: 135  low-grade: 103  high-grade: 32 | 32 | NR | IHC | ABCG2:  clone sc-58222  (1:100, overnight, 4ºC)  BMI-1:  clone ab14389  (1:150, overnight, 4ºC) | ABCG2: membranous-cytoplasmic  BMI-1:  nuclear | ABCG2: 1  BMI-1: 1 | ABCG2:  58 (42.96)  BMI-1:  44 (32.59) |
| Ries *et al.* (2012) | Germany  (English) | R | 1997 - 2004 | 60 (all) | 98 | M= 57 (58.16)  F= 41 (41.84)  (53.7±NR) | NR | NR | NR | NR | No ED: 41  ED: 57  mild: 32  moderate: 18  severe: 7 | 48 | 25 | IHC | MAGE-A:  6C1/57B clones  (1:20/1:50, overnight, 4ºC) | nuclear-cytoplasmic | 1 | MAGE-A: 41 (41.84) |
| Cao *et al.* (2011) | China  (English) | R | 1993-2006 | median:  80.4  (0-275.51) | 76 | M= 42 (55.26)  F= 34 (44.74)  (55.1±13.6, 25-82)  median: 53.5 | Yes: 14 (18.42)  No: 51 (67.11)  missing: 11 (14.47) | Yes: 17 (22.37)  No: 48 (63.16)  missing: 11 (14.47) | NR | NR | No ED: 19  ED: 57 | 37 | NR | IHC | EZH2:  clone 11  (1:200, overnight, 4ºC) | nuclear | Labelling index (intensity x cell count) | EZH2:  60 (78.95) |
| Matsubara *et al.*  (2011) | Japan  (english) | R | 2004 -2008 | NR | 112 | M=72 (64.29)  F=40 (35.71)  (61.9±13.6)  (12-91) | NR | NR | Bm: 10  Tongue: 41  Gingiva: 48  Lip: 1  Palate: 12 | NR | No ED: 76  ED: 36  mild: 22  moderate: 8  severe: 6 | 6 | 10 | IHC | ΔNp63:  clone 4A4  (1:200, 3h, rt)  ki-67:  clone MIB-1  (1:100, 3h, rt) | ΔNp63: nuclear  ki-67:  nuclear | ΔNp63: 1  ki-67:1 | ΔNp63:  112 (100)  ki-67:  112 (100) |
| Taoudi Benchekroun *et al.* (2010) | USA  (English) | P | 1992- 2001 | 90±NR  Median:  89.64  (2.28-183.72) | 145  (missing: 18) | M= 77 (53.10)  F= 68 (46.90)  (NR) | Never: 36 (24.83)  Former: 58 (40.00)  Current: 51 (35.17) | Never: 46 (31.72)  Former: 19 (13.10)  Current: 80 (55.17) | NR | NR | No ED:97  ED: 48  low-grade: 37  high-grade:11 | 35 | NR | IHC | EGFR:  clone 31G7  (1:100, 90 min, RT) | membranous-cytoplasmic | 10 | EGFR:  103 (71.03) |
| Ogmundsdóttir *et al.* (2009) | Iceland  (English) | R | 1990- 1995 | 174±NR  (144–204) | 42 | NR | NR | NR | NR | NR | No ED: 22  ED: 23  low grade: 20  high grade: 3 | 1 | NR | IHC | p53:  clone DO-7  (1:50, 30min, RT) | nuclear-cytoplasmic | Labelling index (intensity) | p53:  6 (14.29) |
| Saintigny *et al.* (2009) | USA  (English) | P | 1994 - 2001 | 90±NR  median:  89.64  (2.28-183.72) | 162  (missing: 10) | M= 85 (52.45)  F= 77 (47.53)  (NR±NR, median 56, 23-90) | Never: 41 (25.31)  Former: 65 (40.12)  Current: 56 (34.57) | Never: 50 (30.86)  Former: 19 (11.73)  Current: 93 (57.41) | NR | NR | No ED: 109  ED: 53  low-grade: 40  high-grade: 13 | 36 | NR | IHC | ΔNp63:  4A4 clone  (1:400, 1h, RT) | nuclear | Labelling index (intensity) | ΔNp63: 41 (26.97) |
| Kawaguchi *et al.* (2008) | USA  (English) | P | 1994- 2001 | 90±NR  median:  89.64  (2.28-183.72) | 150  (missing: 18) | M= 80 (53.33)  F= 70 (46.67)  (56.03±13.58,23-90) | Never: 38 (25.33)  Former: 60 (40.00)  Current: 52 (34.67) | Never: 46 (30.67)  Former: 18 (12.00)  Current: 85 (56.67) | NR | NR | No ED: 101  ED: 49  mild: 37  moderate: 10  severe: 2 | 35 | NR | IHC | podoplanin:  clone D2-40  (1:100, overnight, 4°C) | membranous | 1 | podoplanin:  56 (37.33) |
| Matthias *et al.* (2008) | Germany (English) | R | NR | NR | 19 | NR | NR | NR | NR | NR | No ED: 9  ED: 6 | 4 | NR | IHC | CK8:  clone 35βH11  (1:100, NR, NR) | NR | Labelling index (intensity) | CK8:  10 (52.63) |
| Santos-García *et al.* (2005) | Spain  (Spanish) | R | 1990-2000 | NR | 42 | NR | NR | NR | NR | NR | No ED: 11  ED: 30  Low grade: 2  High grade: 28 | 12 | 11 | IHC | p53:  clone DO7 (1:200, 30min, NR)  ki-67:  clone MIB-1  (1:100, 30min, NR) | Nuclear | 20 | p53:  22 (52.38)  ki-67:  26 (61.90) |
| Soni *et al.* (2005) | India  (English) | P | NR | NR | 90 | M= 72 (80.00)  F= 18 (20.00)  (NR, NR) | NR | NR | Bm: 64  Tongue: 14  Gingiva: 3  Lip: 5  Other: 4 | NR | No ED: 52  ED: 38 | NR | 81 | IHC | cyclin D1:  R-124  (1:100, NR, NR)  pRb:  IF8  (1:100, NR, NR)  p53:  D0-7 (1:100,NR,NR) | nuclear | 10 | cyclin D1:  41 (45.56)  pRb:  60 (66.67)  p53:  NR |
| Kikegawa.  (2001) | Japan  (Japanese) | R | NR | NR | 43 | NR  (37.6±16.6, 20-70) | NR | NR | NR | NR | No ED/Mild:25  Moderate/Severe: 23  Analysis Units: lesions | 11 | NR | IHC | p53:  clone BP 53-12  (1:100, 24h, 4ºC) | nuclear | 10 | p53:  22 (51.16) |
| Sulkowska *et al.* (2001) | Poland  (English) | R | NR | NR | 55 | M=31 (56.36)  F=4 (7.27)  missing=20  (NR,NR) | NR | NR | Tongue:15 Lip:10 Fom:10 missing=20 | NR | No ED: 7  ED: 48  mild:23 moderate:  16  severe:9 | 35 | NR | IHC | p53:  clone D0-7  (NR,NR,NR)  Bcl-2:  clone M0887  (NR,NR,NR) | nuclear-cytoplasmic | 10 | p53:  28 (50.91)  Bcl-2:  24 (43.64) |
| Oliver *et al.* (2000) | United Kingdom  (English) | R | NR | NR | 14 | M= 5 (35.71)  F= 9 (64.29)  (62.23±15.17) | NR | NR | Tongue: 5  Fom: 8 | NR | No ED:0  ED: 14  minimal: 2  mild: 3  moderate: 6  severe: 3 | 2 | NR | IHC | BrdU:  clone Bu20a  (1:50, overnight, 4ºC)  ki-67:  clone MM1  (1:100, 2h, rt) | nuclear | BrdU:  10.87  ki-67:  30.68 | BrdU:  4 (28.57)  ki-67:  3 (21.43) |
| Tanda *et al.* (2000) | Japan  (English) | R | NR | 84±NR  (48-96) | 13 | M= 7 (53.85)  F= 6 (46.15)  (56±NR) | NR | NR | NR | NR | No ED:2  ED: 11  mild: 5  moderate: 5  severe:1 | 1 | 9 | IHC | Bcl-2:  clone 124  (NR, NR, NR)  wt-p53:  clone Ab-5  (NR, NR, NR)  p21:  clone 187  (NR, NR, NR)  MDM2:  clone IF2  (NR, NR, NR) | Bcl-2: nuclear  wt-p53: nuclear  p21:  nuclear  MDM2:  nuclear | Bcl-2:1  wt-p53:1  p21:1  MDM2:1 | Bcl-2:  12 (92.31)  wt-p53:  8 (61.54)  p21:  11 (84.62)  MDM2:  13 (100) |
| Rich *et al.* (1999) | Australia  (English) | R | NR | NR±NR  (10-106) | 41  (missing: 11) | M= NR (NR)  F= NR (NR)  (NR±NR) | NR | NR | NR | NR | No ED: 0  ED: 41  mild: 16  moderate: 4  severe: 10  missing: 11 | 3 | 8 | IHC | p53:  clone DO-7  (1:25, 1h, RT) | p53:  nuclear | p53: 50 | p53:  26 (86.67) |
| Cruz *et al.* (1998) | The Netherlands(English) | R | NR | 54.72±NR  (12-180) | 32 | M= 10 (31.25)  F= 22 (68.75)  (62.53±NR, 28-86) | Yes: 24  No: 8 | NR | Tongue: 15  Fom: 5  Others: 12 | NR | No ED: NR  ED: NR  mild: NR  moderate/severe: 12 | 10 | 11 | IHC | p53:  clone DO-7  (1:500, overnight, 4ºC) | nuclear | 1 | p53:  25 (78.13) |
| Papadimitrakopoulou *et al.* (1997) | USA  (English) | R | NR | median: 63 | 36 | NR | NR | NR | NR | NR | NR | 8 | NR | IHC | p16:  NR  (1:400, overnight, 4ºC) | nuclear-cytoplasmic | 5 | p16:  19 (52.78) |
| Kanekawa *et al.* (1995) | Japan  (English) | R | 1993 | (36-NR) | 28 | M= 16 (57.14)  F= 12  (42.86)  (59±NR, 42-89) | NR | NR | Bm: 1  Tongue: 23  Gingiva: 1  Palate: 3 | NR | No ED: 21  ED: 7 | 5 | NR | IHC | PCNA:  clone PC-10  (1:20, 60min, RT) | nuclear | 10 | PCNA:  9 (32.14) |
| Abbreviations: n, number; m, months; y, years; SD, standard deviation; NR, not reported; ED, epithelial dysplasia; IHC, immunohistochemistry; M, male; F, female; bm, buccal mucosa; rmt, retromolar trigone; fom, floor of mouth; h, hour; min, minutes; RT, room temperature;…. | | | | | | | | | | | | | | | | | | |

**3. Meta-analysis on the expression of hallmarks of cancer in oral leukoplakia**

**3.1 Hallmark Sustaining proliferative signaling**

**Figure S1.** Forest plot graphically representing the meta-analysis on the magnitude of association -using RR as effect size metric- between hallmark of cancer expression and OLs malignant transformation risk. RR, relative risk; CI, confidence intervals, DerSimonian and Laird, DL. Random-effects model, inverse-variance weighting based on the DL method. A RR > 1 suggests a higher malignant transformation risk. Diamonds indicate the pooled RR with their corresponding 95% CIs.


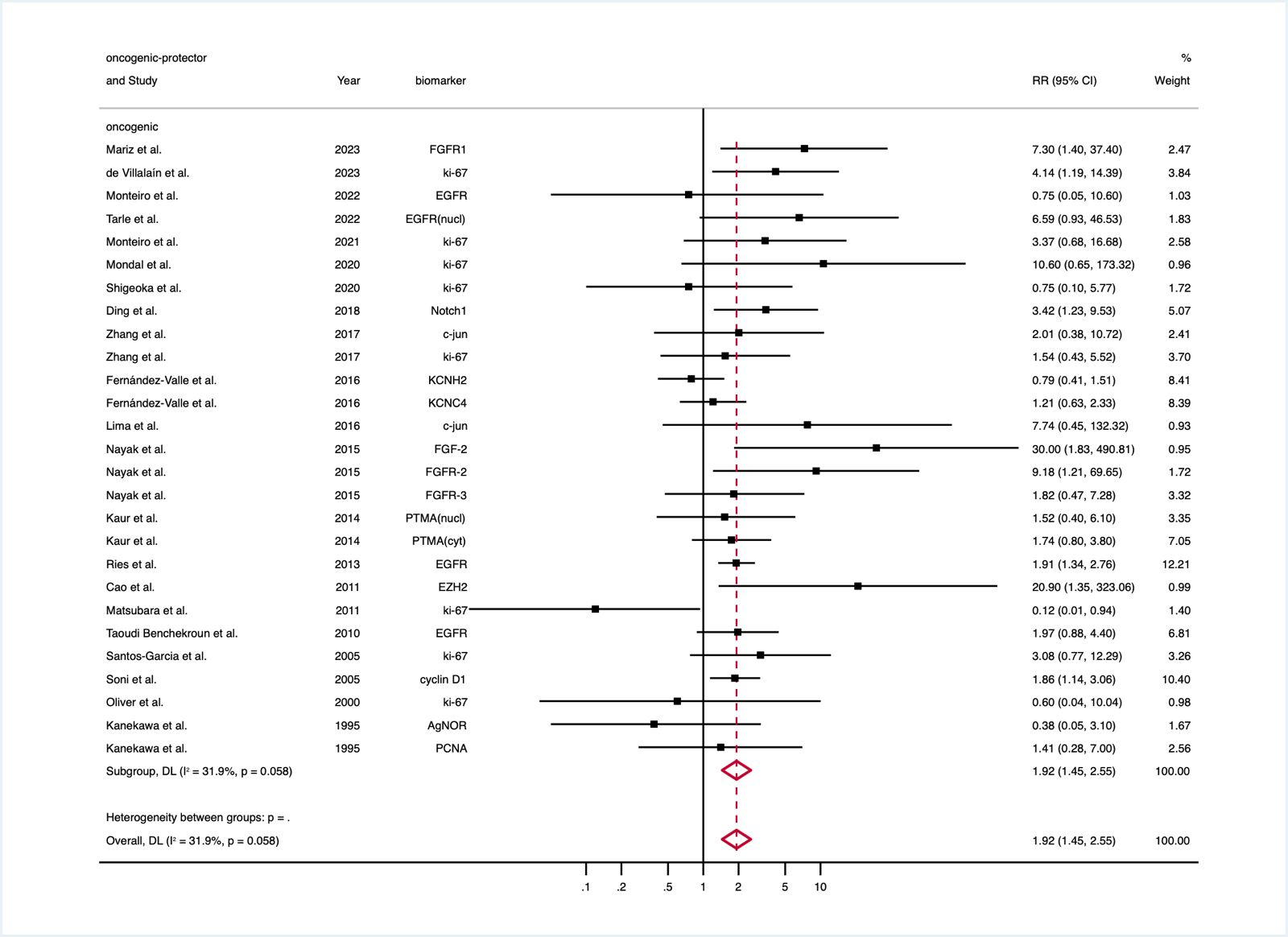


**Figure S2.** Forest plot graphically representing the differential expression of biomarkers on the hallmark sustaining proliferative signaling -using pooled proportions as ES metric, expressed as percentage- among OL patients. ES, effect size; CI, confidence interval; Random-effects model.


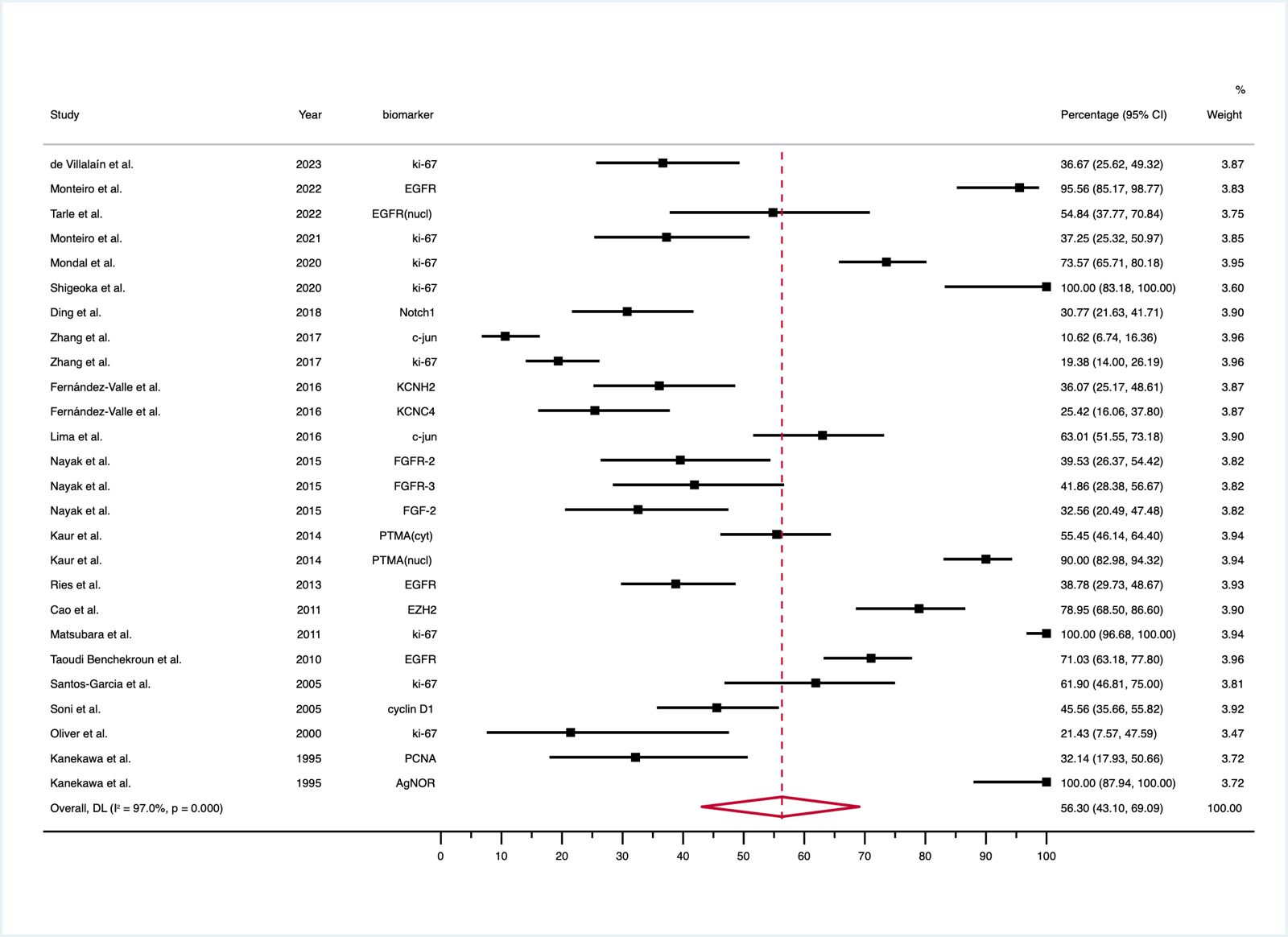


**Figure S3.** Forest plot graphically representing the meta-analysis of the magnitude of association -using OR as effect size metric- in order to compare the differential expression of biomarkers on the hallmark sustaining proliferative signaling between OL and healthy controls. OR, odds ratio; CI, confidence interval; Random-effects model, inverse-variance weighting based on the DL method. A OR> 1 suggests a higher expression in OL in comparison to healthy oral mucosa. Diamonds indicate the pooled OR with their corresponding 95% CIs.


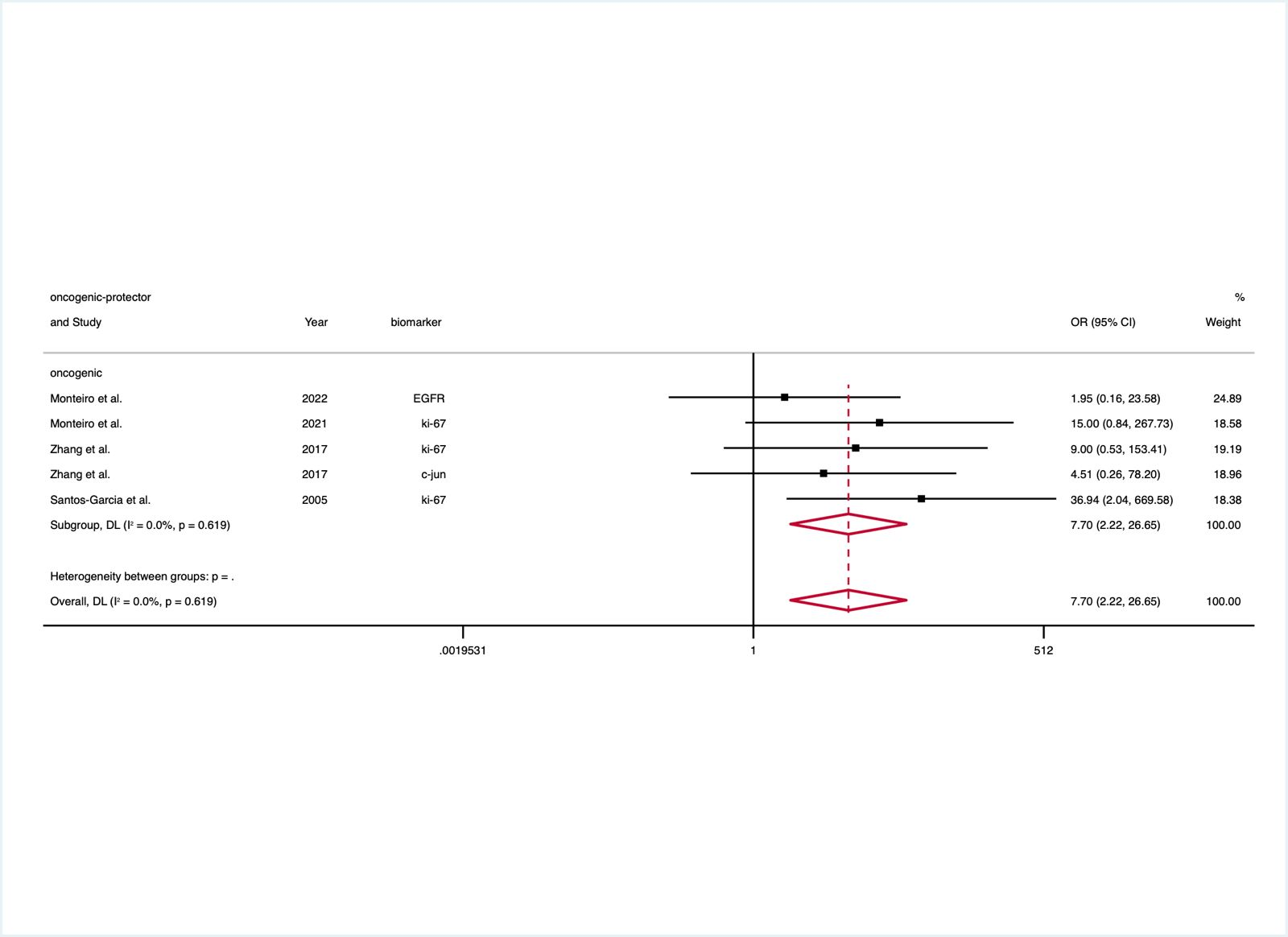


**3.2 Hallmark Evading growth suppressors**

**Figure S4.** Forest plot graphically representing the meta-analysis on the magnitude of association -using RR as effect size metric- between hallmark of cancer expression and OLs malignant transformation risk. RR, relative risk; CI, confidence intervals, DerSimonian and Laird, DL. Random-effects model, inverse-variance weighting based on the DL method. A RR > 1 suggests a higher malignant transformation risk. Diamonds indicate the pooled RR with their corresponding 95% CIs.


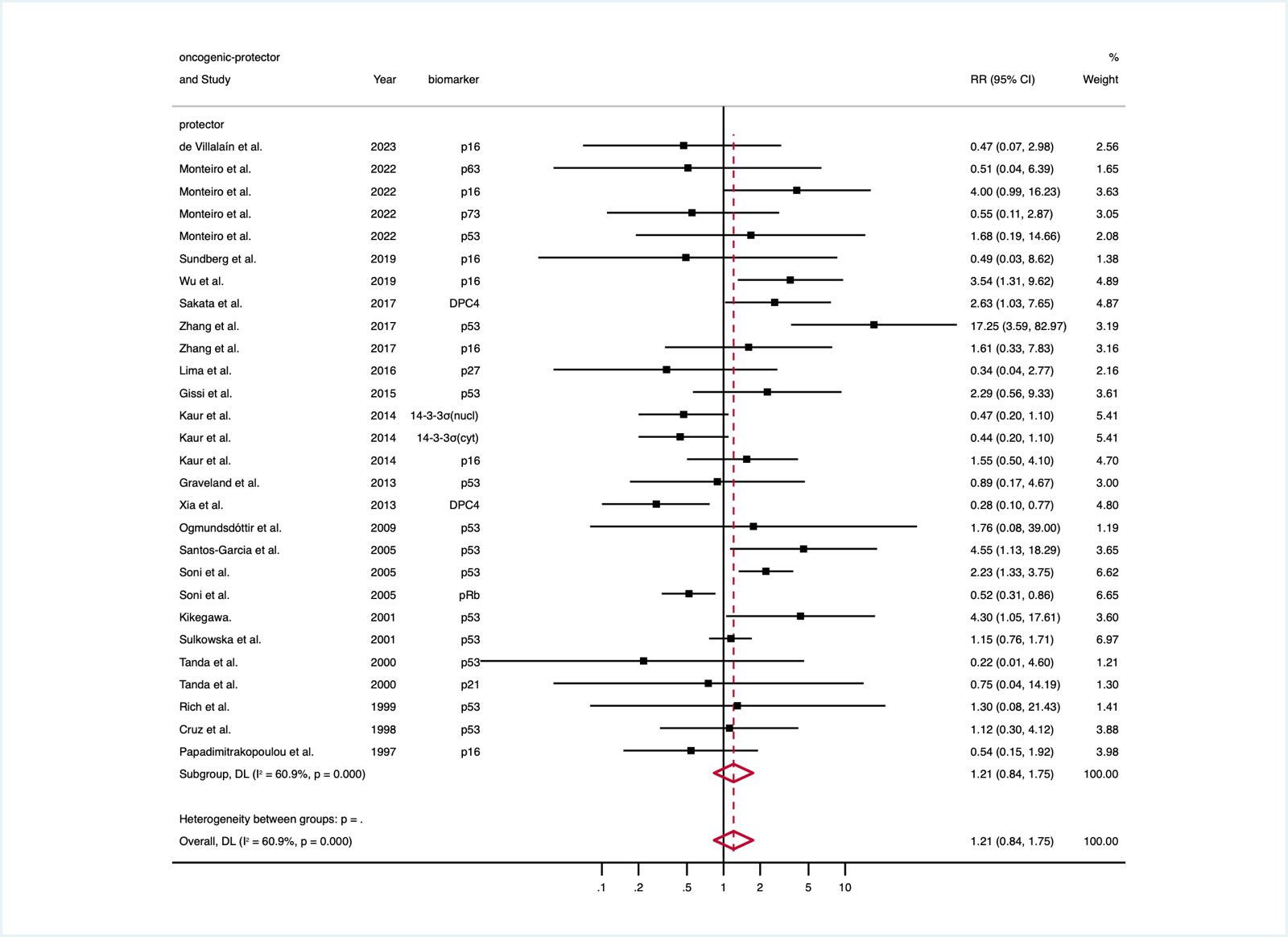


**Figure S5.** Forest plot graphically representing the differential expression of biomarkers on the hallmark evading growth suppressors -using pooled proportions as ES metric, expressed as percentage- among OL patients. ES, effect size; CI, confidence interval; Random-effects model.


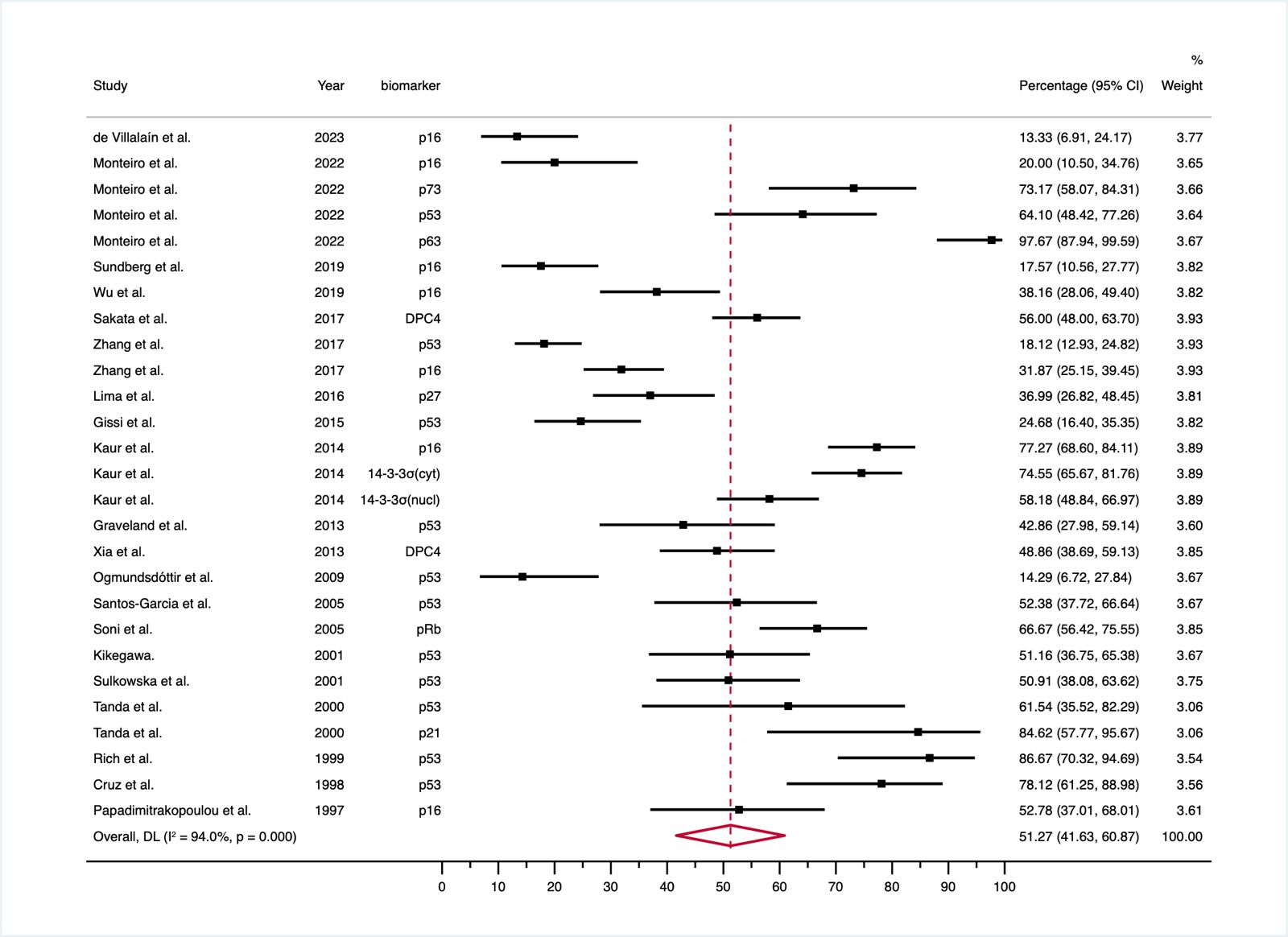


**Figure S6.** Forest plot graphically representing the meta-analysis of the magnitude of association -using OR as effect size metric- in order to compare the differential expression of biomarkers on the hallmark evading growth suppressors between OL and healthy controls. OR, odds ratio; CI, confidence interval; Random-effects model, inverse-variance weighting based on the DL method. A OR> 1 suggests a higher expression in OL in comparison to healthy oral mucosa. Diamonds indicate the pooled OR with their corresponding 95% CIs.


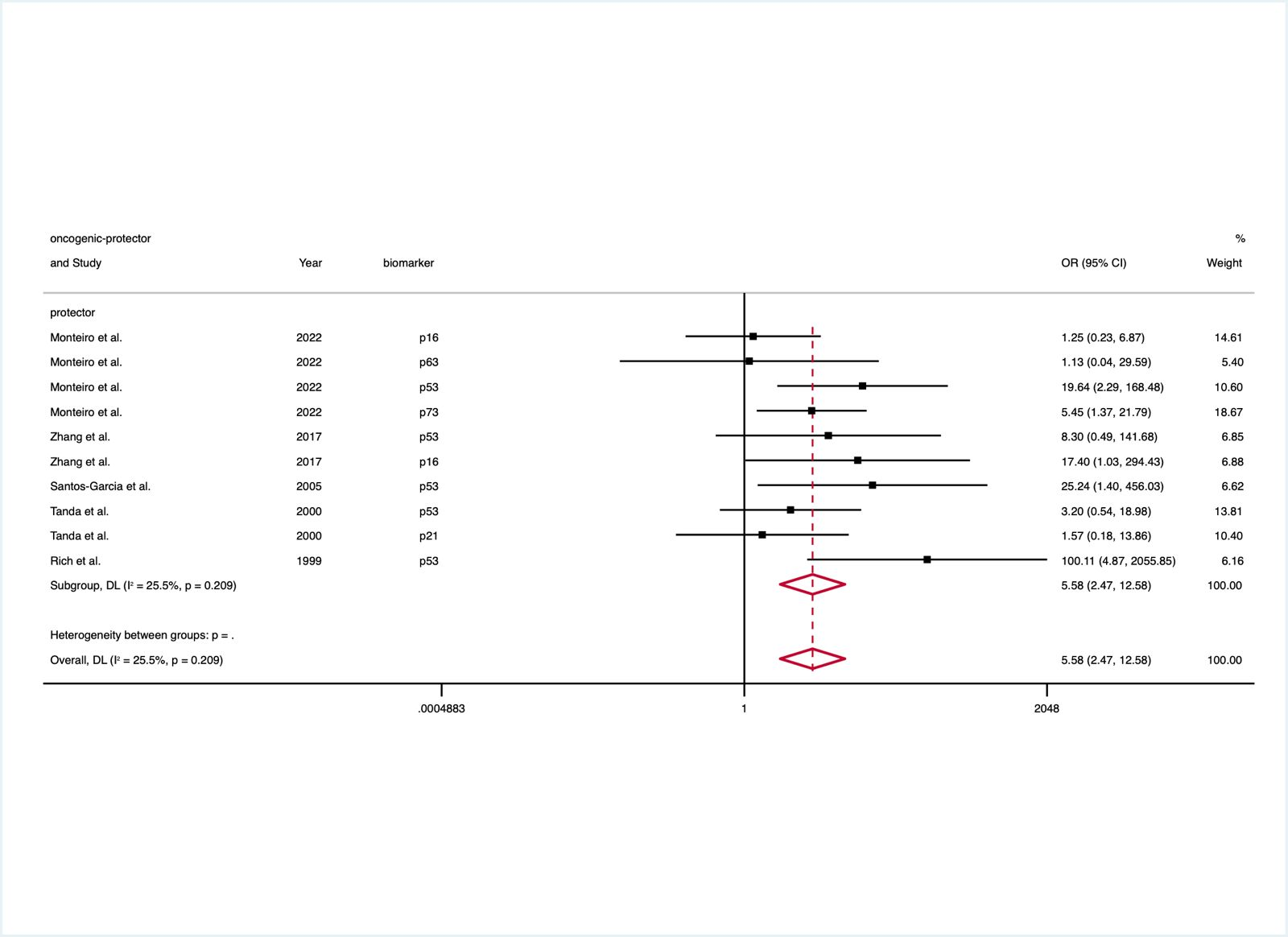


**3.3 Hallmark Resisting cell death**

**Figure S7.** Forest plot graphically representing the meta-analysis on the magnitude of association -using RR as effect size metric- between hallmark of cancer expression and OLs malignant transformation risk. RR, relative risk; CI, confidence intervals, DerSimonian and Laird, DL. Random-effects model, inverse-variance weighting based on the DL method. A RR > 1 suggests a higher malignant transformation risk. Diamonds indicate the pooled RR with their corresponding 95% CIs.


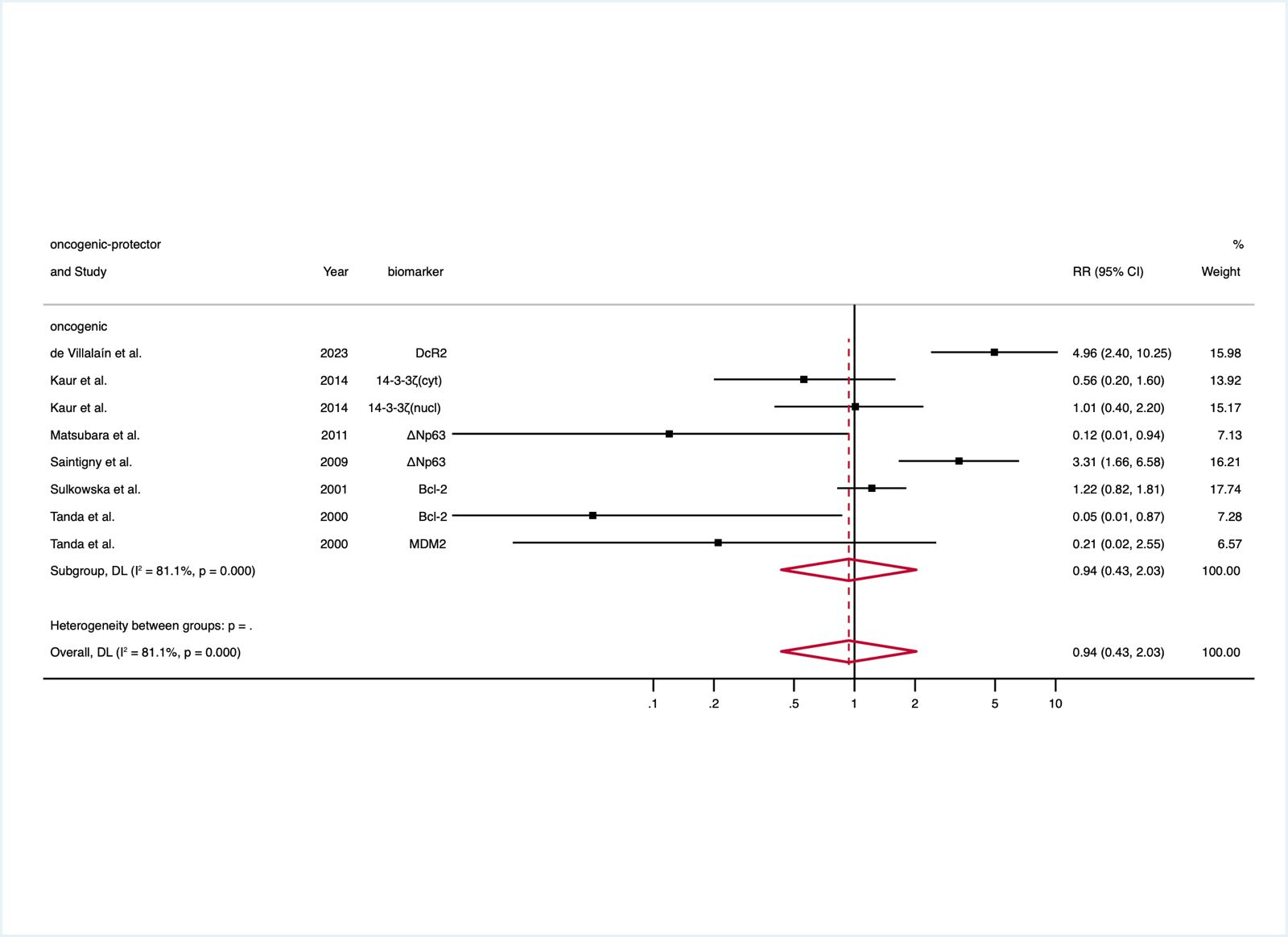


**Figure S8.** Forest plot graphically representing the differential expression of biomarkers on the hallmark resisting cell death -using pooled proportions as ES metric, expressed as percentage- among OL patients. ES, effect size; CI, confidence interval; Random-effects model.


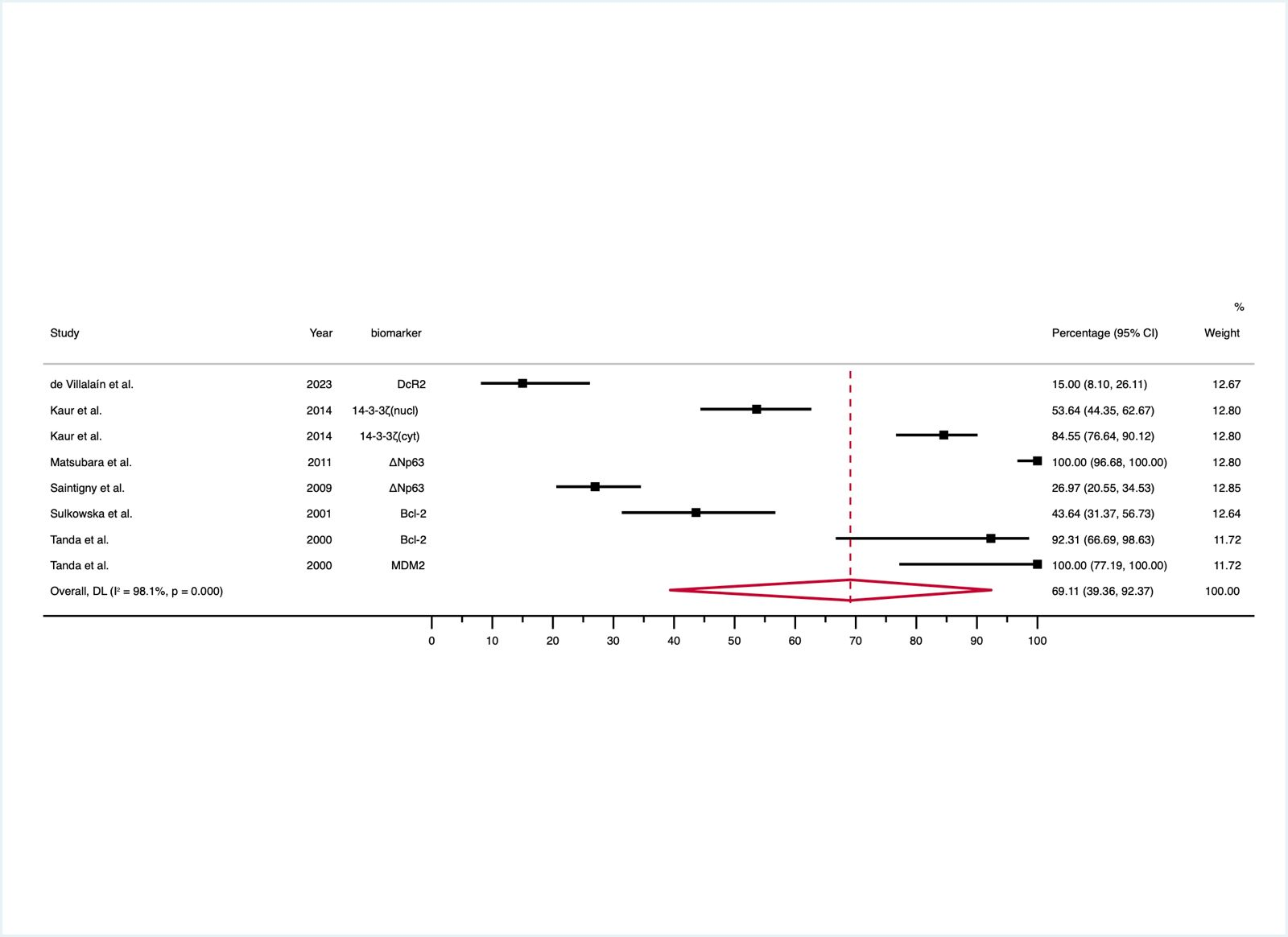


**Figure S9.** Forest plot graphically representing the meta-analysis of the magnitude of association -using OR as effect size metric- in order to compare the differential expression of biomarkers on the hallmark resisting cell death between OL and healthy controls. OR, odds ratio; CI, confidence interval; Random-effects model, inverse-variance weighting based on the DL method. A OR> 1 suggests a higher expression in OL in comparison to healthy oral mucosa. Diamonds indicate the pooled OR with their corresponding 95% CIs.


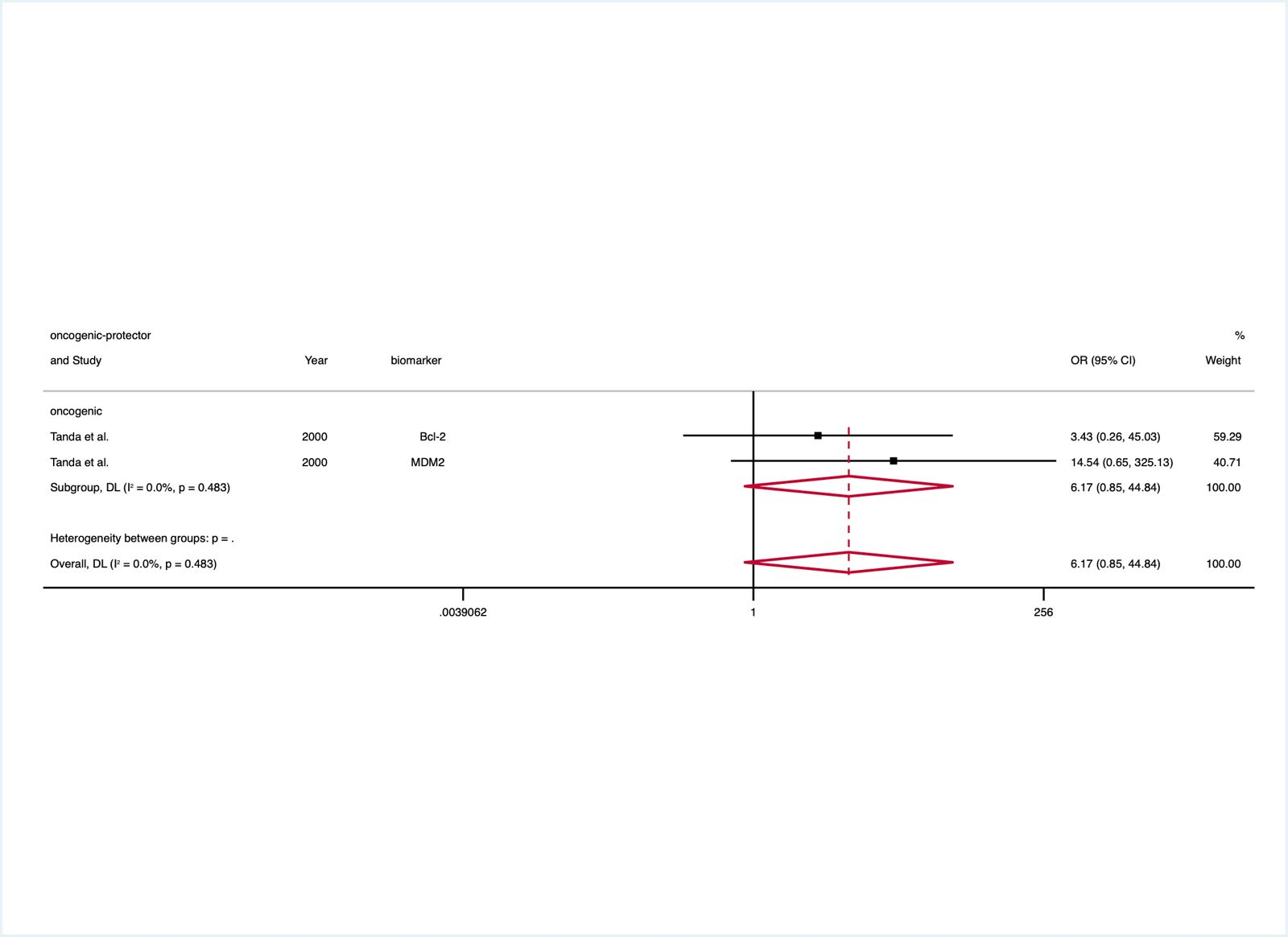


**3.4 Hallmark Enabling replicative immortality**

**Figure S10.** Forest plot graphically representing the meta-analysis on the magnitude of association -using RR as effect size metric- between hallmark of cancer expression and OLs malignant transformation risk. RR, relative risk; CI, confidence intervals, DerSimonian and Laird, DL. Random-effects model, inverse-variance weighting based on the DL method. A RR > 1 suggests a higher malignant transformation risk. Diamonds indicate the pooled RR with their corresponding 95% CIs.


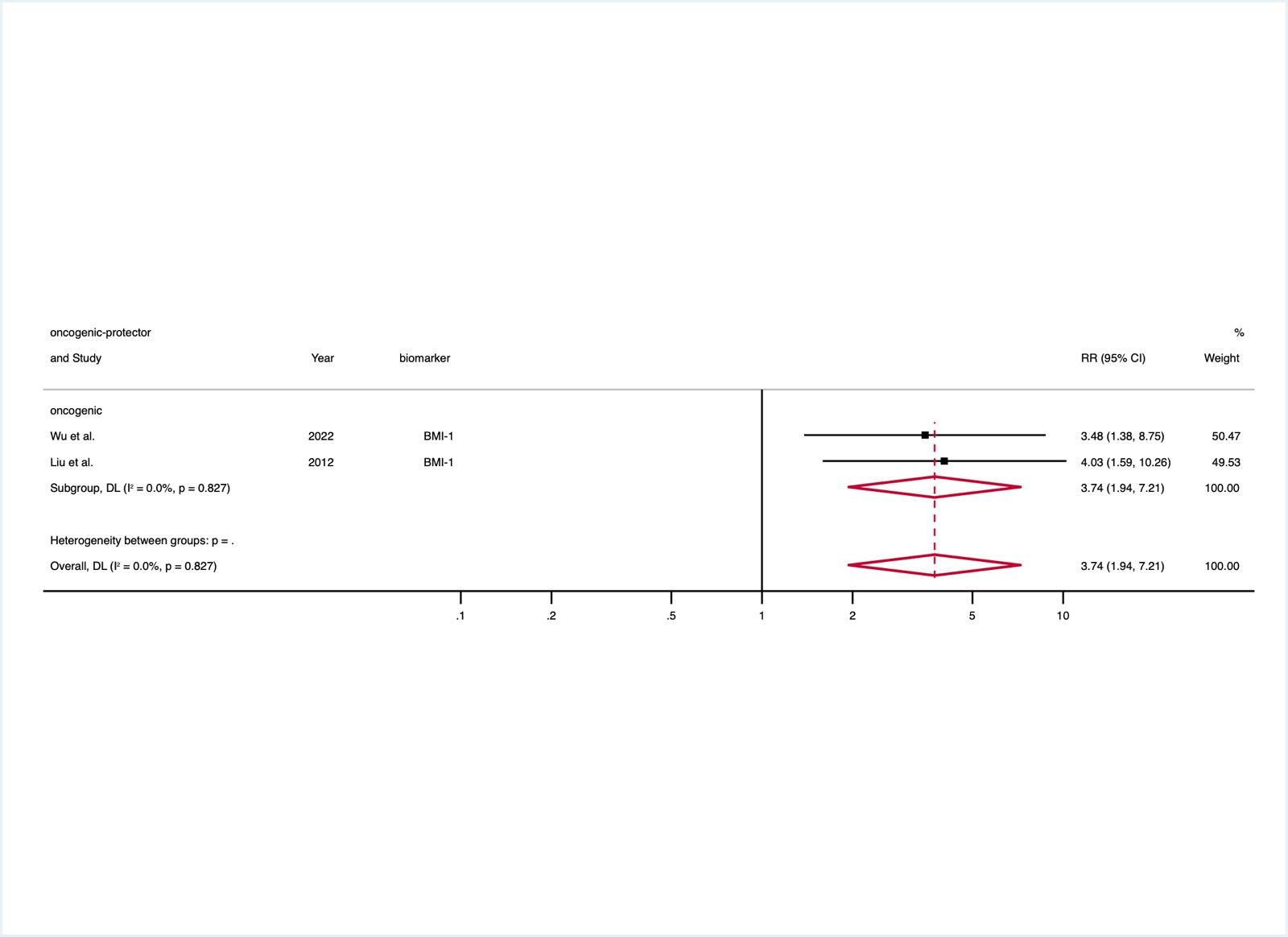


**Figure S11.** Forest plot graphically representing the differential expression of biomarkers on the hallmark enabling replicative immortality -using pooled proportions as ES metric, expressed as percentage- among OL patients. ES, effect size; CI, confidence interval; Random-effects model.


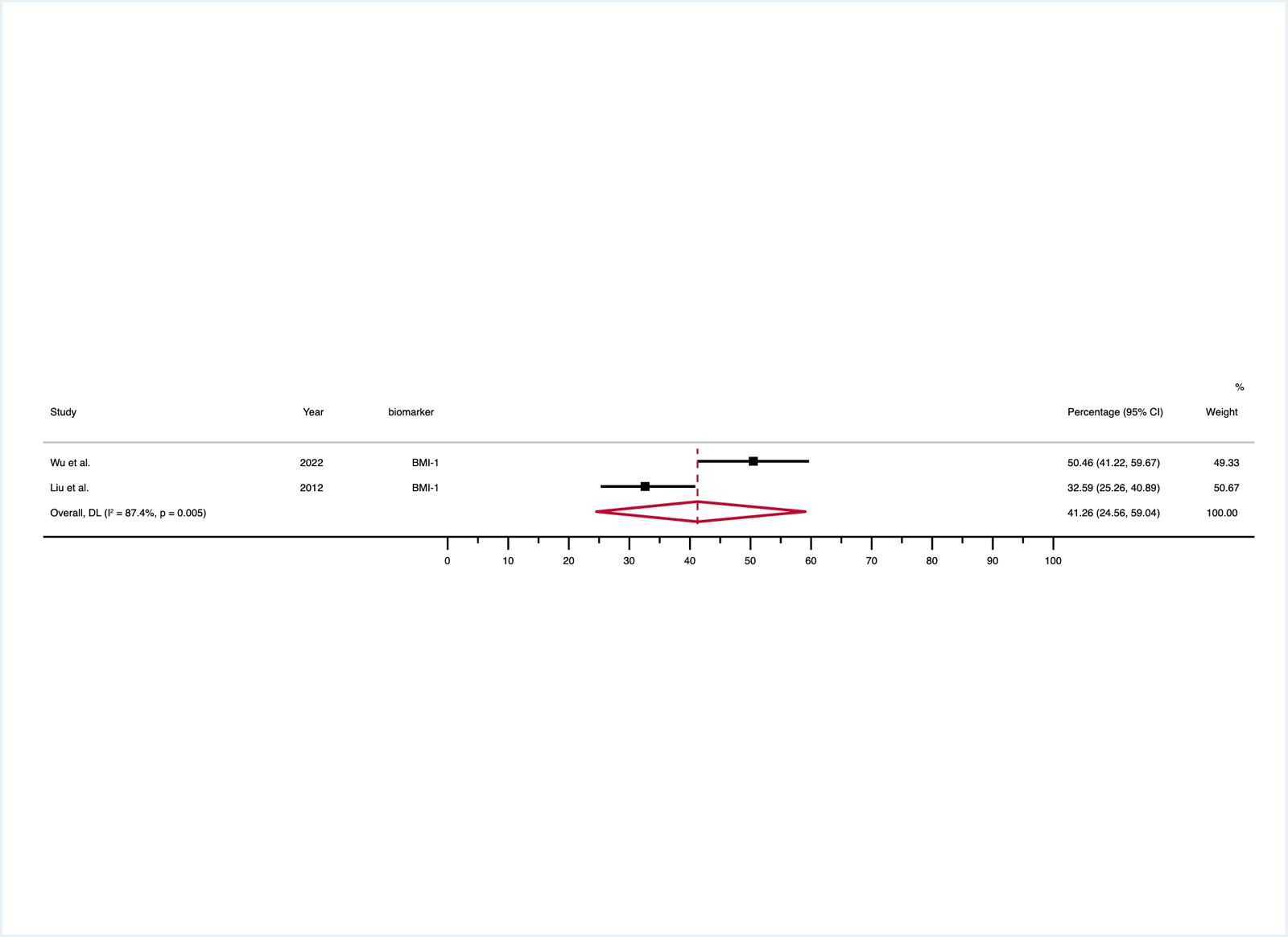


**3.5 Hallmark Inducing angiogenesis**

**Figure S12.** Forest plot graphically representing the meta-analysis on the magnitude of association -using RR as effect size metric- between hallmark of cancer expression and OLs malignant transformation risk. RR, relative risk; CI, confidence intervals, DerSimonian and Laird, DL. Random-effects model, inverse-variance weighting based on the DL method. A RR > 1 suggests a higher malignant transformation risk. Diamonds indicate the pooled RR with their corresponding 95% CIs.


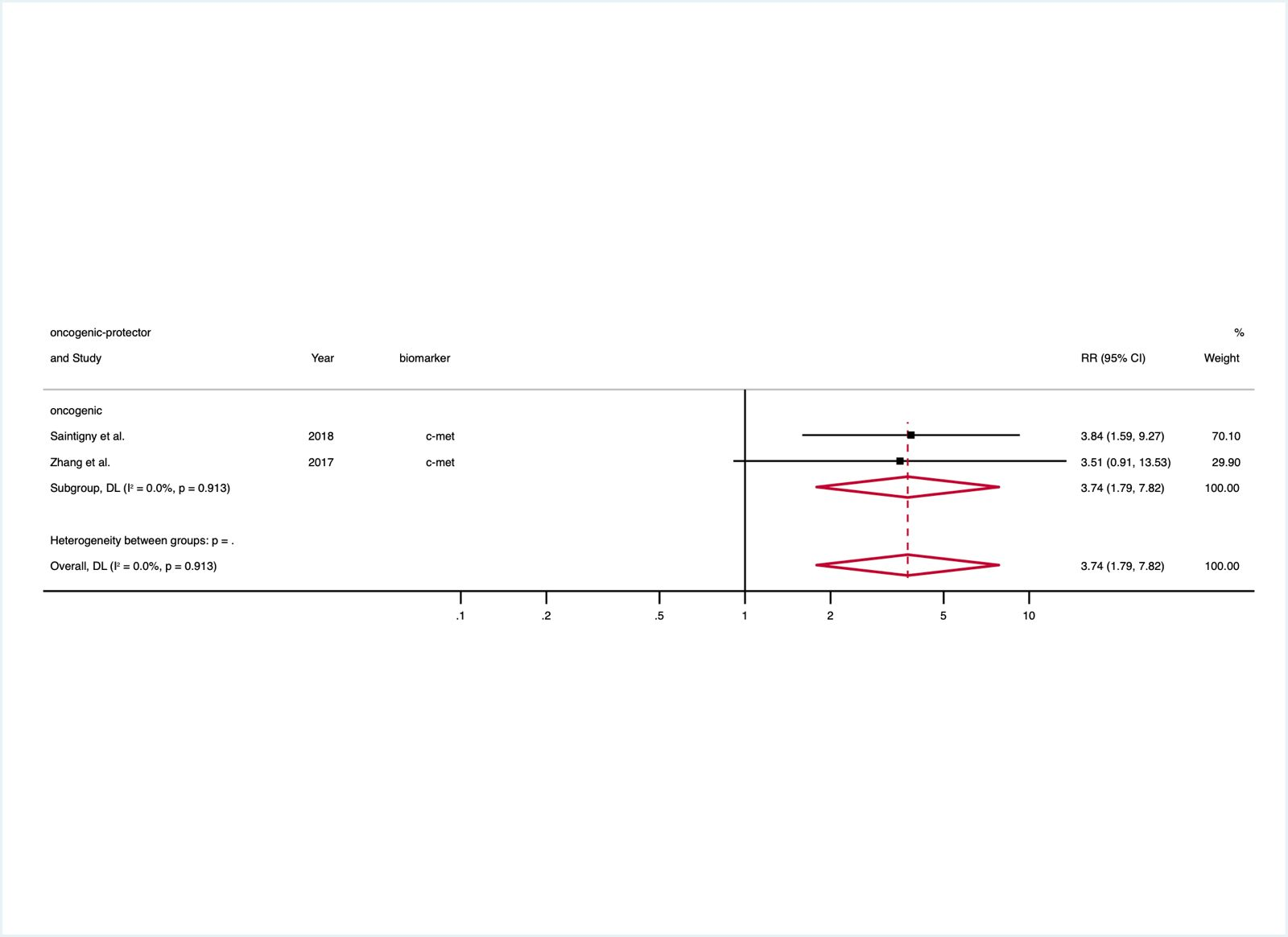


**Figure S13.** Forest plot graphically representing the differential expression of biomarkers on the hallmark inducing angiogenesis -using pooled proportions as ES metric, expressed as percentage- among OL patients. ES, effect size; CI, confidence interval; Random-effects model.


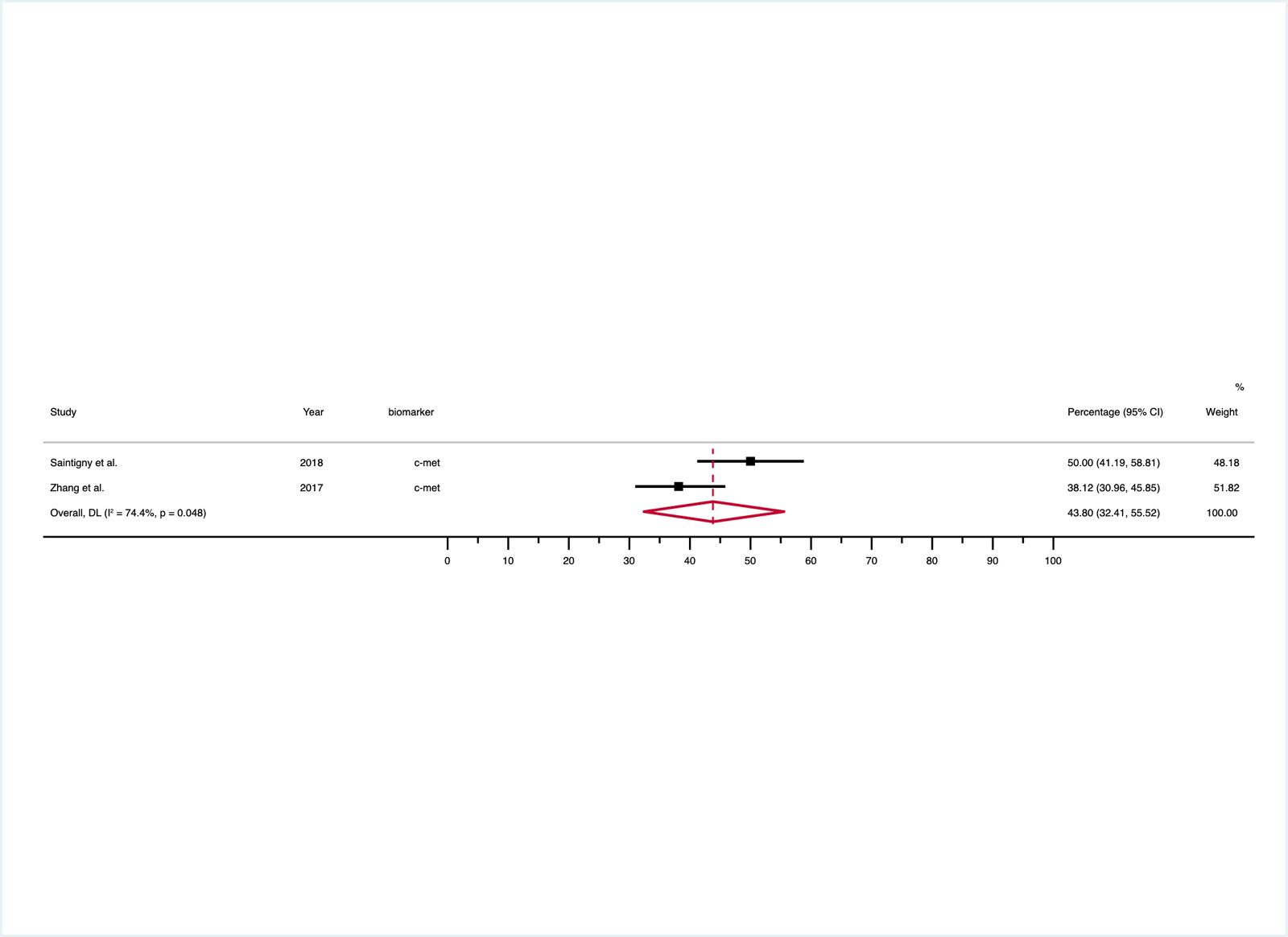


**3.6 Hallmark Activating invasion and metastasis**

**Figure S14.** Forest plot graphically representing the meta-analysis on the magnitude of association -using RR as effect size metric- between hallmark of cancer expression and OLs malignant transformation risk. RR, relative risk; CI, confidence intervals, DerSimonian and Laird, DL. Random-effects model, inverse-variance weighting based on the DL method. A RR > 1 suggests a higher malignant transformation risk. Diamonds indicate the pooled RR with their corresponding 95% CIs.


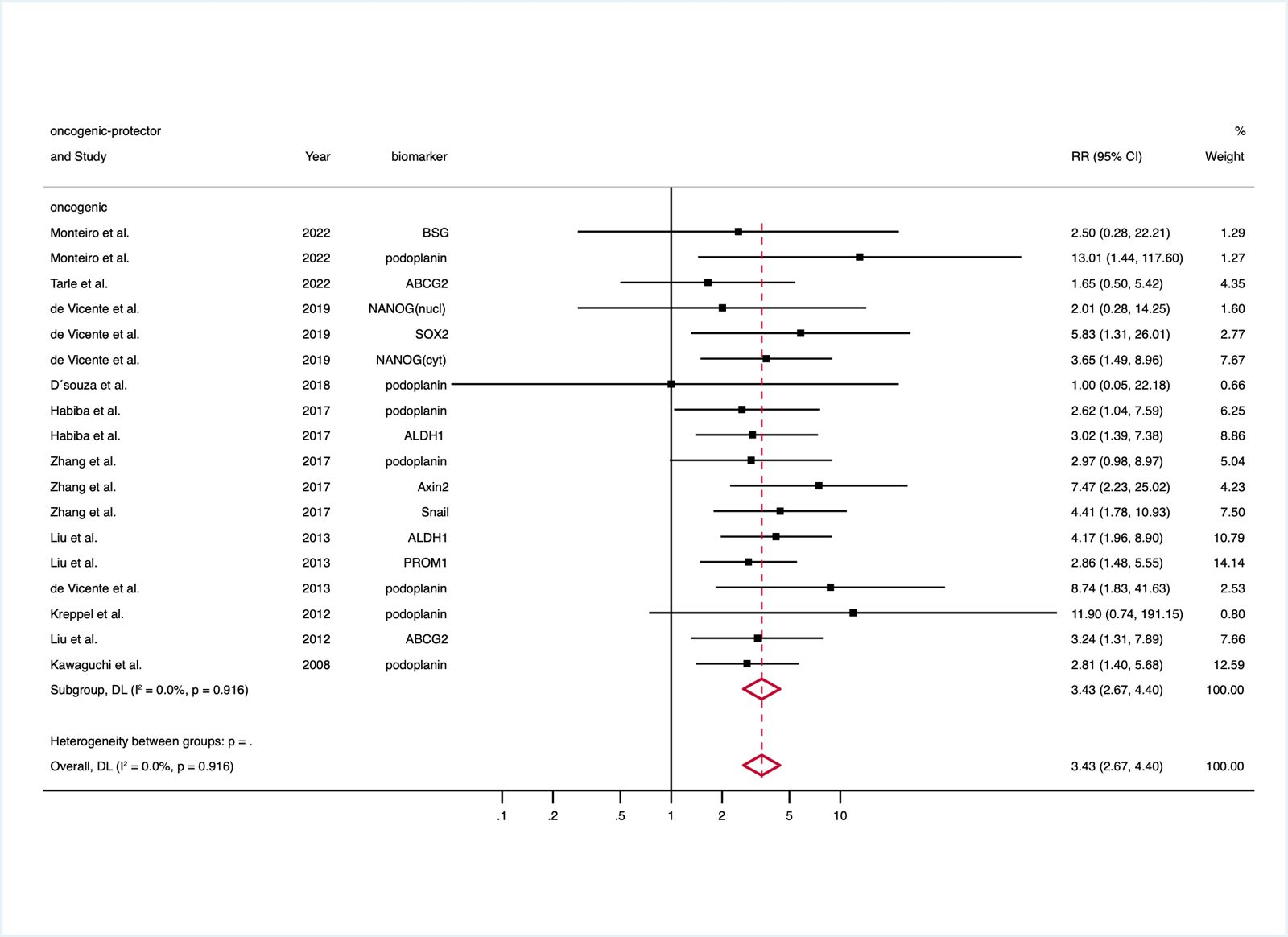


**Figure S15.** Forest plot graphically representing the differential expression of biomarkers on the hallmark activating invasion and metastasis -using pooled proportions as ES metric, expressed as percentage- among OL patients. ES, effect size; CI, confidence interval; Random-effects model.


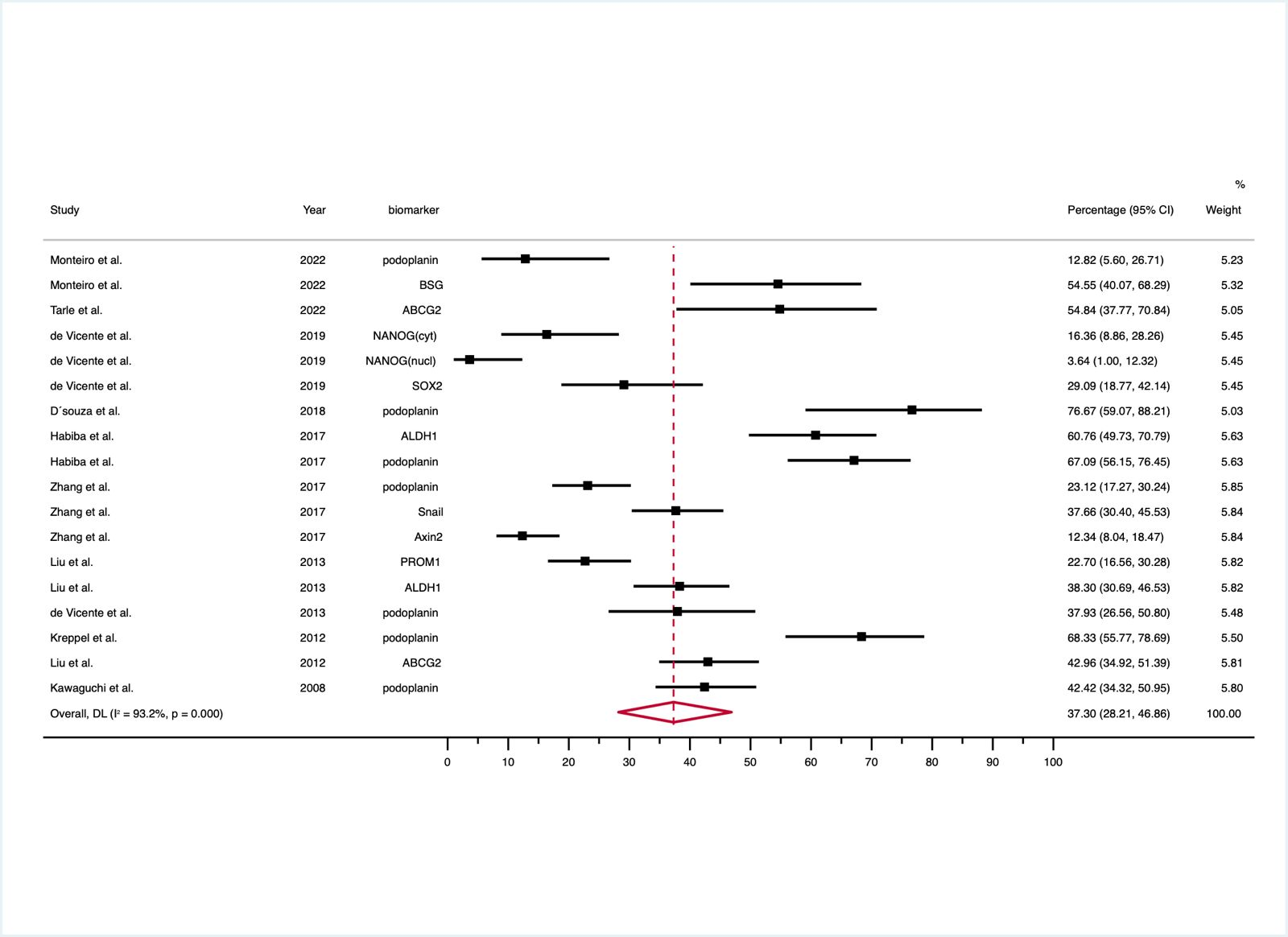


**Figure S16.** Forest plot graphically representing the meta-analysis of the magnitude of association -using OR as effect size metric- in order to compare the differential expression of biomarkers on the hallmark activating invasion and metastasis between OL and healthy controls. OR, odds ratio; CI, confidence interval; Random-effects model, inverse-variance weighting based on the DL method. A OR> 1 suggests a higher expression in OL in comparison to healthy oral mucosa. Diamonds indicate the pooled OR with their corresponding 95% CIs.

**
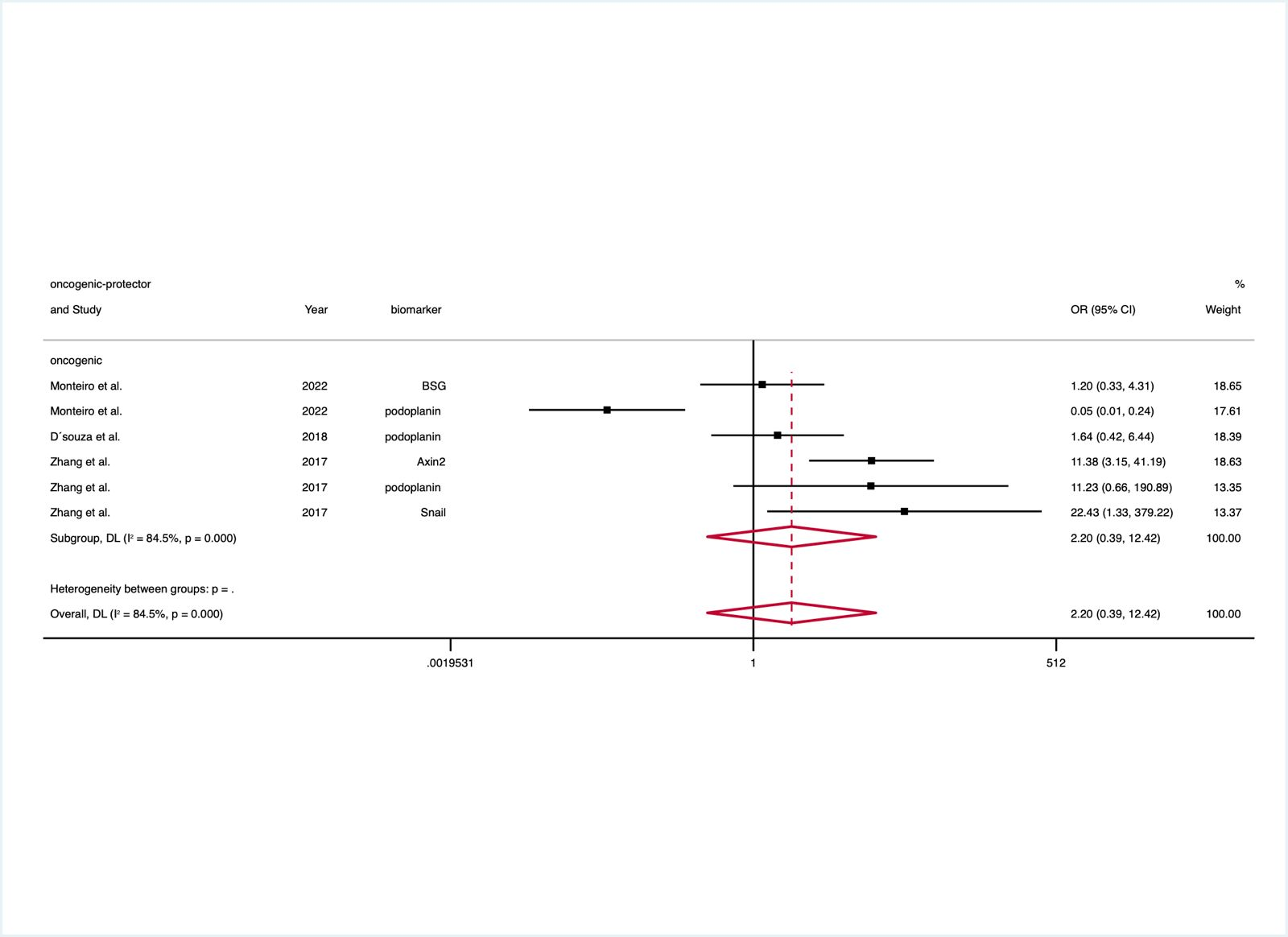
**

**3.7 Hallmark Avoiding immune destruction**

**Figure S17.** Forest plot graphically representing the meta-analysis on the magnitude of association -using RR as effect size metric- between hallmark of cancer expression and OLs malignant transformation risk. RR, relative risk; CI, confidence intervals, DerSimonian and Laird, DL. Random-effects model, inverse-variance weighting based on the DL method. A RR > 1 suggests a higher malignant transformation risk. Diamonds indicate the pooled RR with their corresponding 95% CIs.


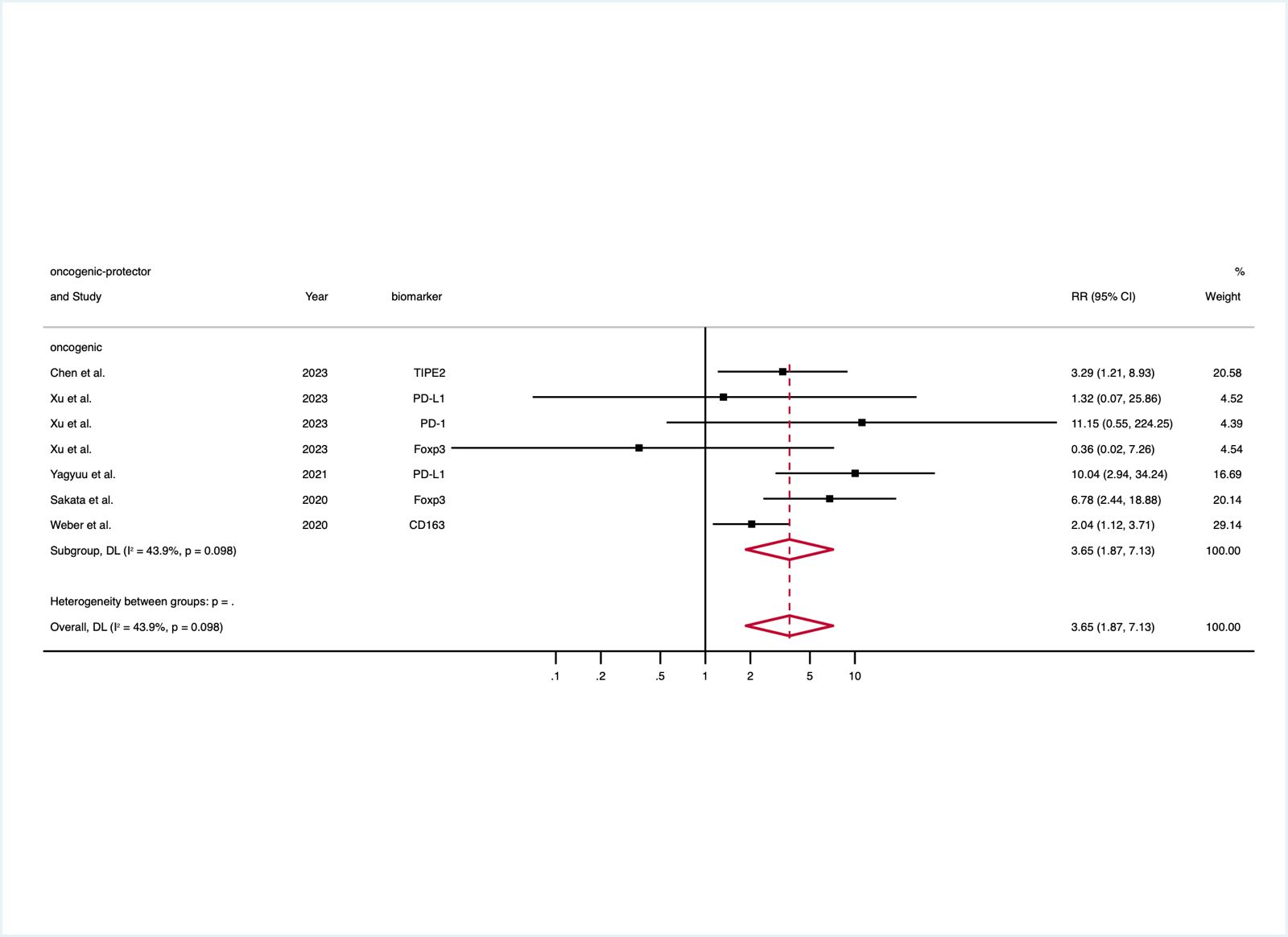


**Figure S18.** Forest plot graphically representing the differential expression of biomarkers on the hallmark avoiding immune destruction -using pooled proportions as ES metric, expressed as percentage- among OL patients. ES, effect size; CI, confidence interval; Random-effects model.


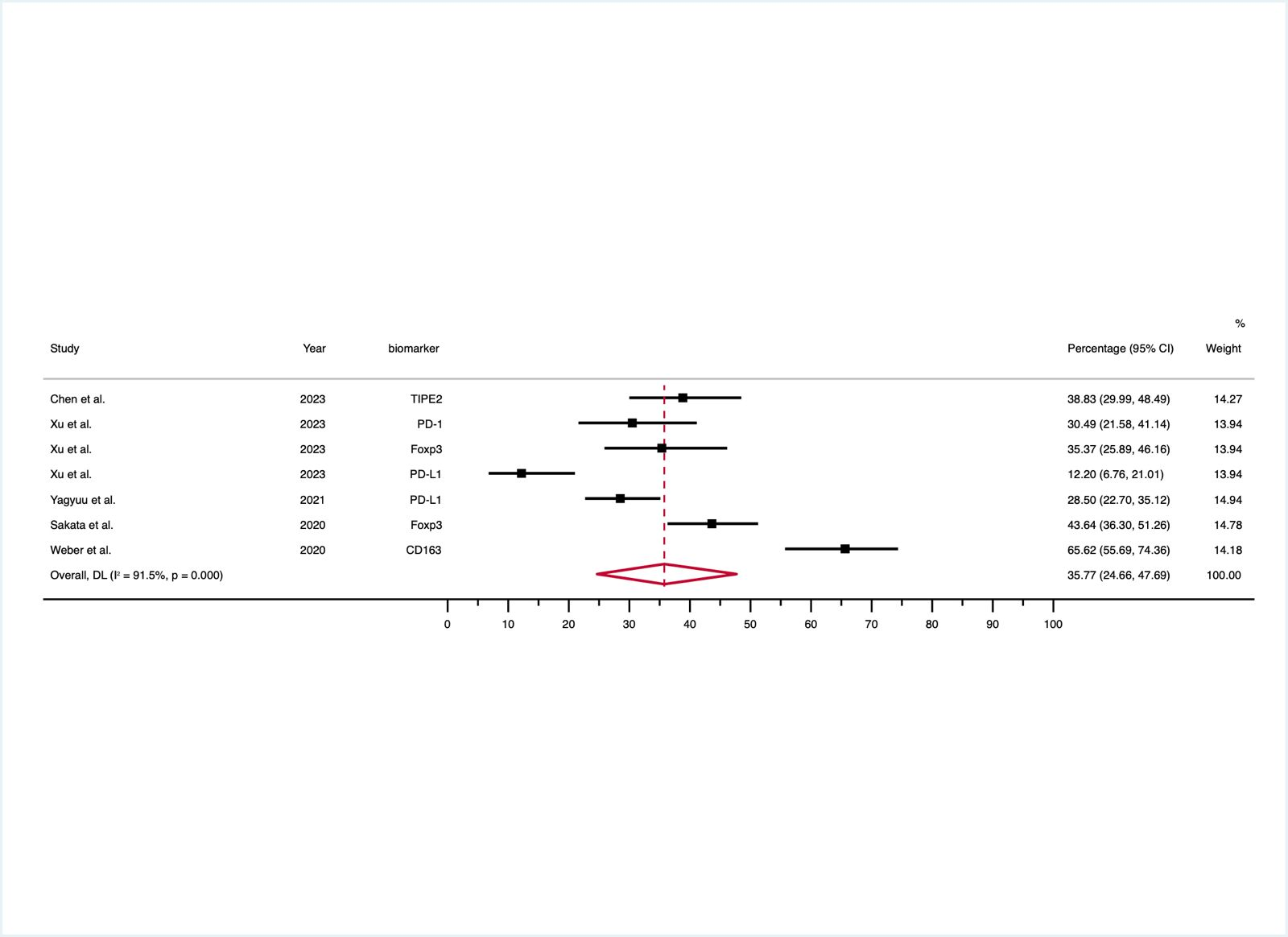


**3.8 Hallmark Deregulating cellular energetics**

**Figure S19.** Forest plot graphically representing the meta-analysis on the magnitude of association -using RR as effect size metric- between hallmark of cancer expression and OLs malignant transformation risk. RR, relative risk; CI, confidence intervals, DerSimonian and Laird, DL. Random-effects model, inverse-variance weighting based on the DL method. A RR > 1 suggests a higher malignant transformation risk. Diamonds indicate the pooled RR with their corresponding 95% CIs.


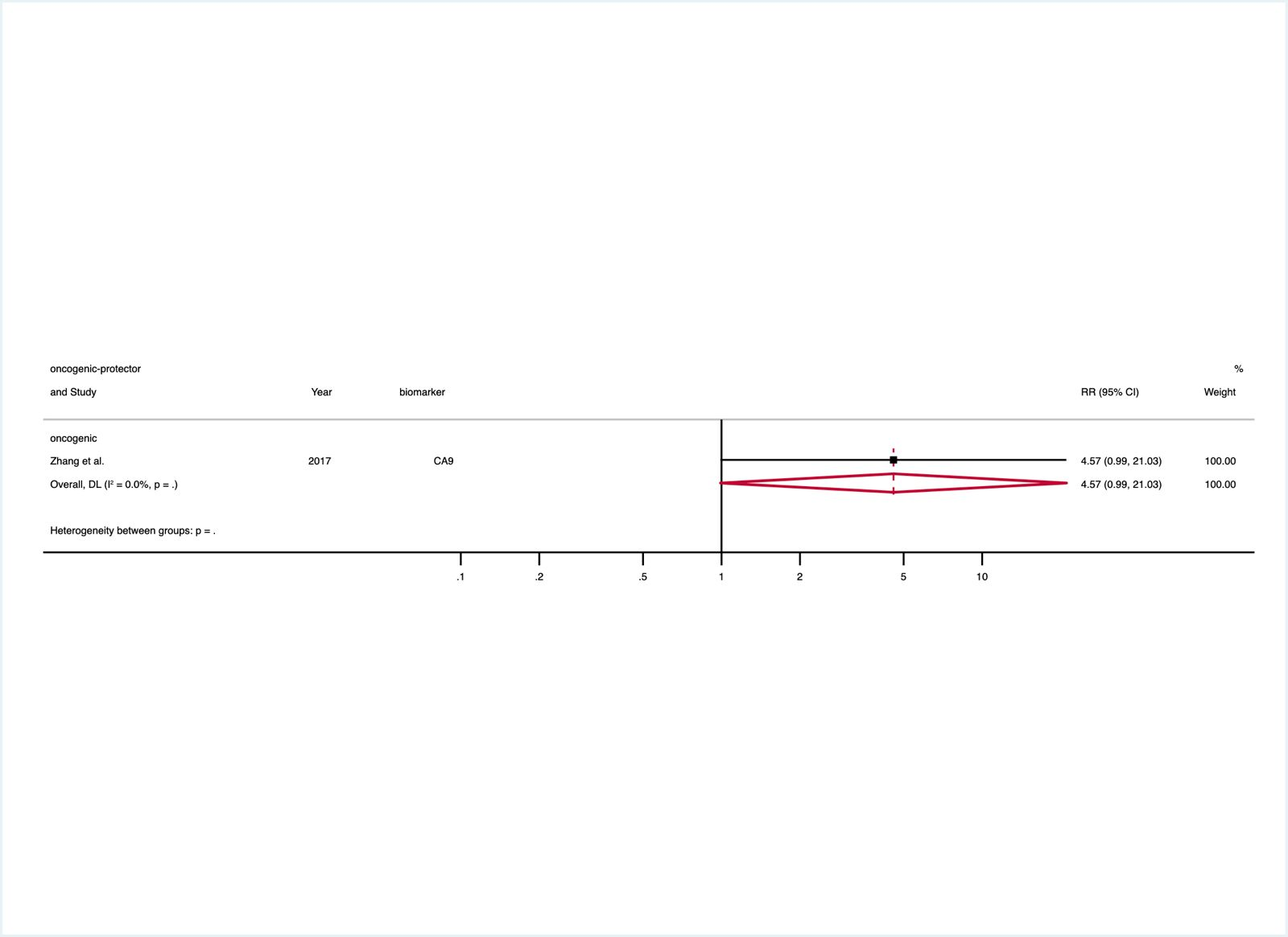


**Figure S20.** Forest plot graphically representing the differential expression of biomarkers on the hallmark deregulating cellular energetics -using pooled proportions as ES metric, expressed as percentage- among OL patients. ES, effect size; CI, confidence interval; Random-effects model.


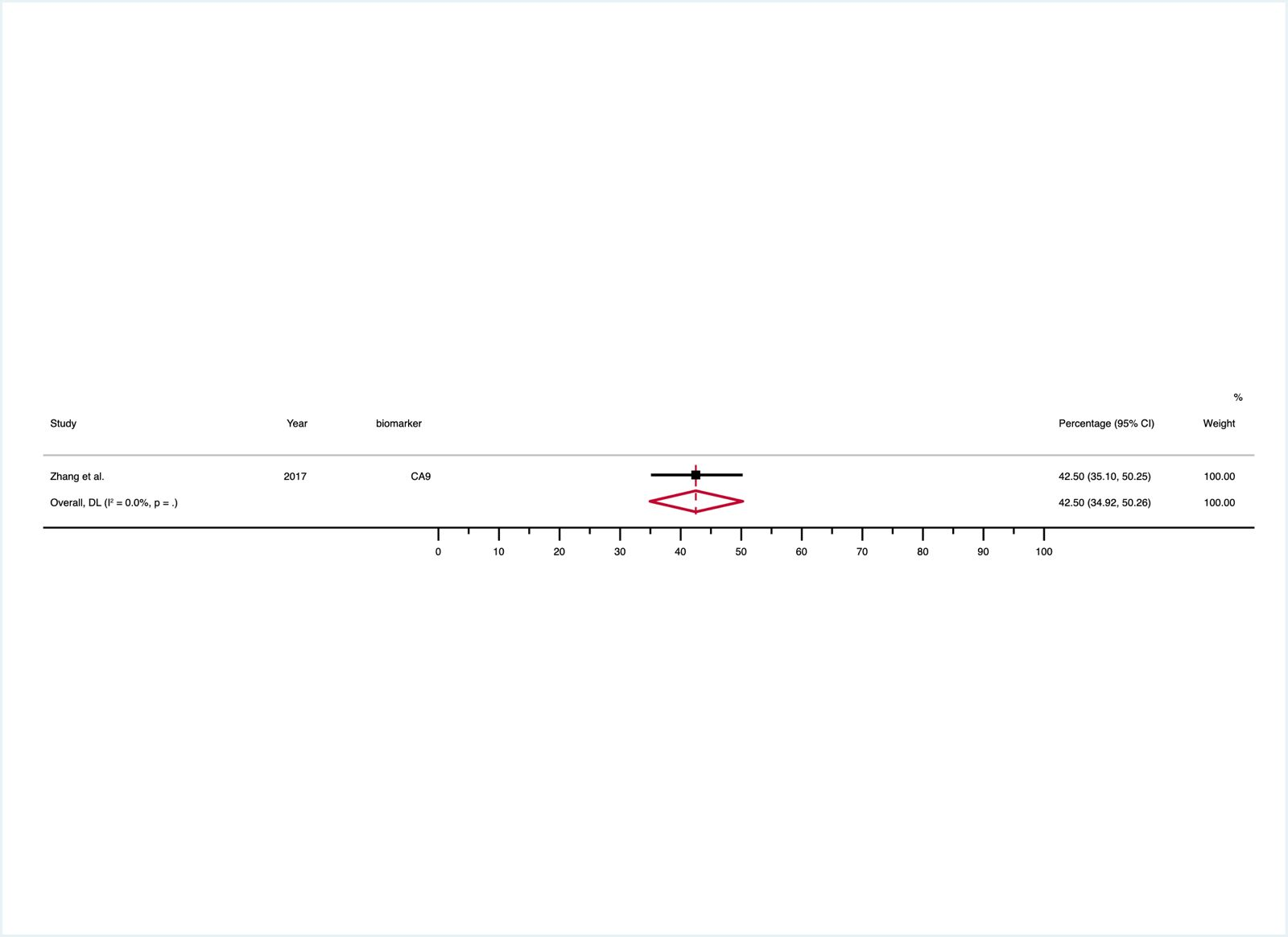


**Figure S21.** Forest plot graphically representing the meta-analysis of the magnitude of association -using OR as effect size metric- in order to compare the differential expression of biomarkers on the hallmark deregulating cellular energetics between OL and healthy controls. OR, odds ratio; CI, confidence interval; Random-effects model, inverse-variance weighting based on the DL method. A OR> 1 suggests a higher expression in OL in comparison to healthy oral mucosa. Diamonds indicate the pooled OR with their corresponding 95% CIs.


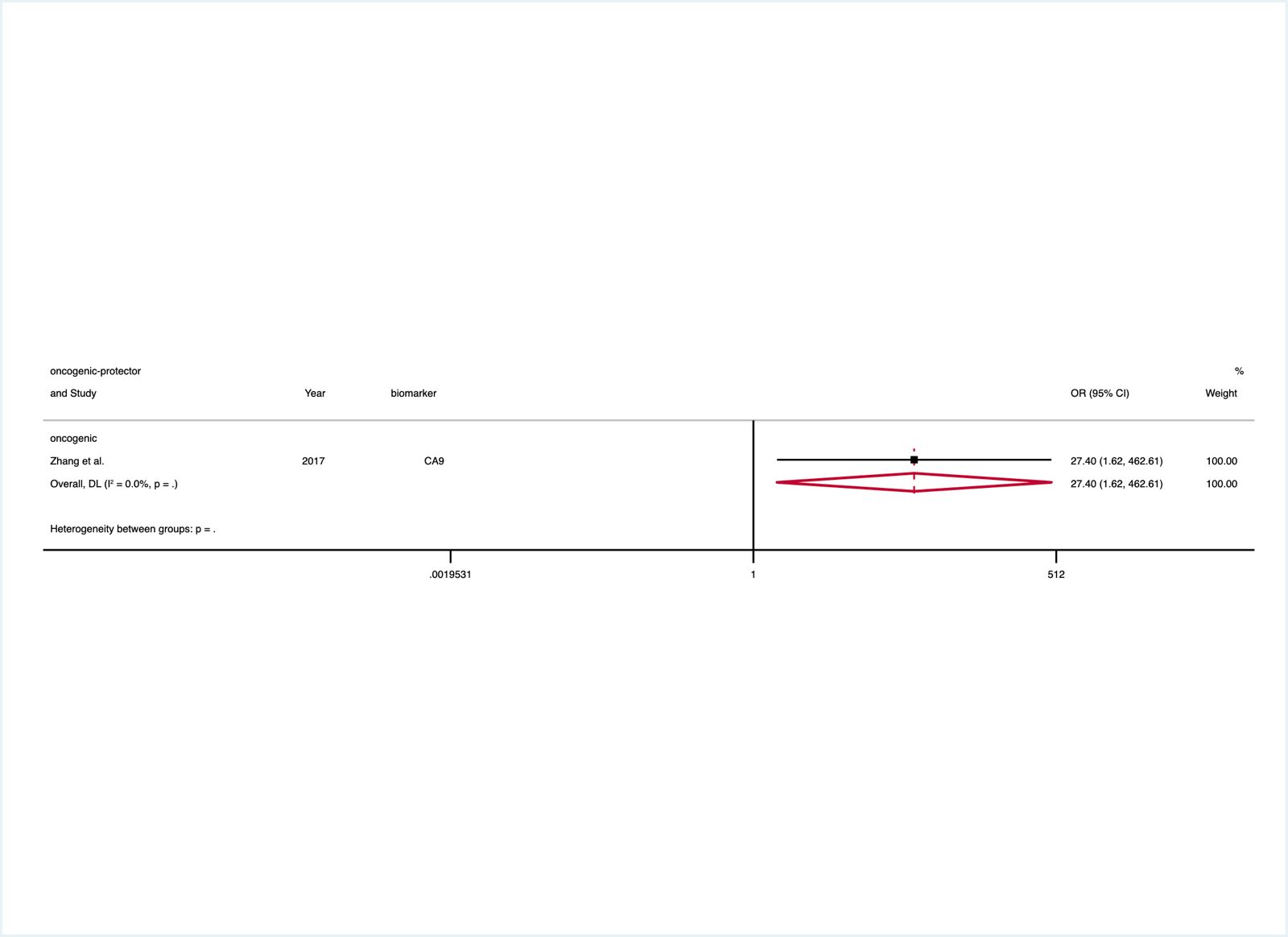


**3.9 Hallmark Genome instability and mutation**

**Figure S22.** Forest plot graphically representing the meta-analysis on the magnitude of association -using RR as effect size metric- between hallmark of cancer expression and OLs malignant transformation risk. RR, relative risk; CI, confidence intervals, DerSimonian and Laird, DL. Random-effects model, inverse-variance weighting based on the DL method. A RR > 1 suggests a higher malignant transformation risk. Diamonds indicate the pooled RR with their corresponding 95% CIs.


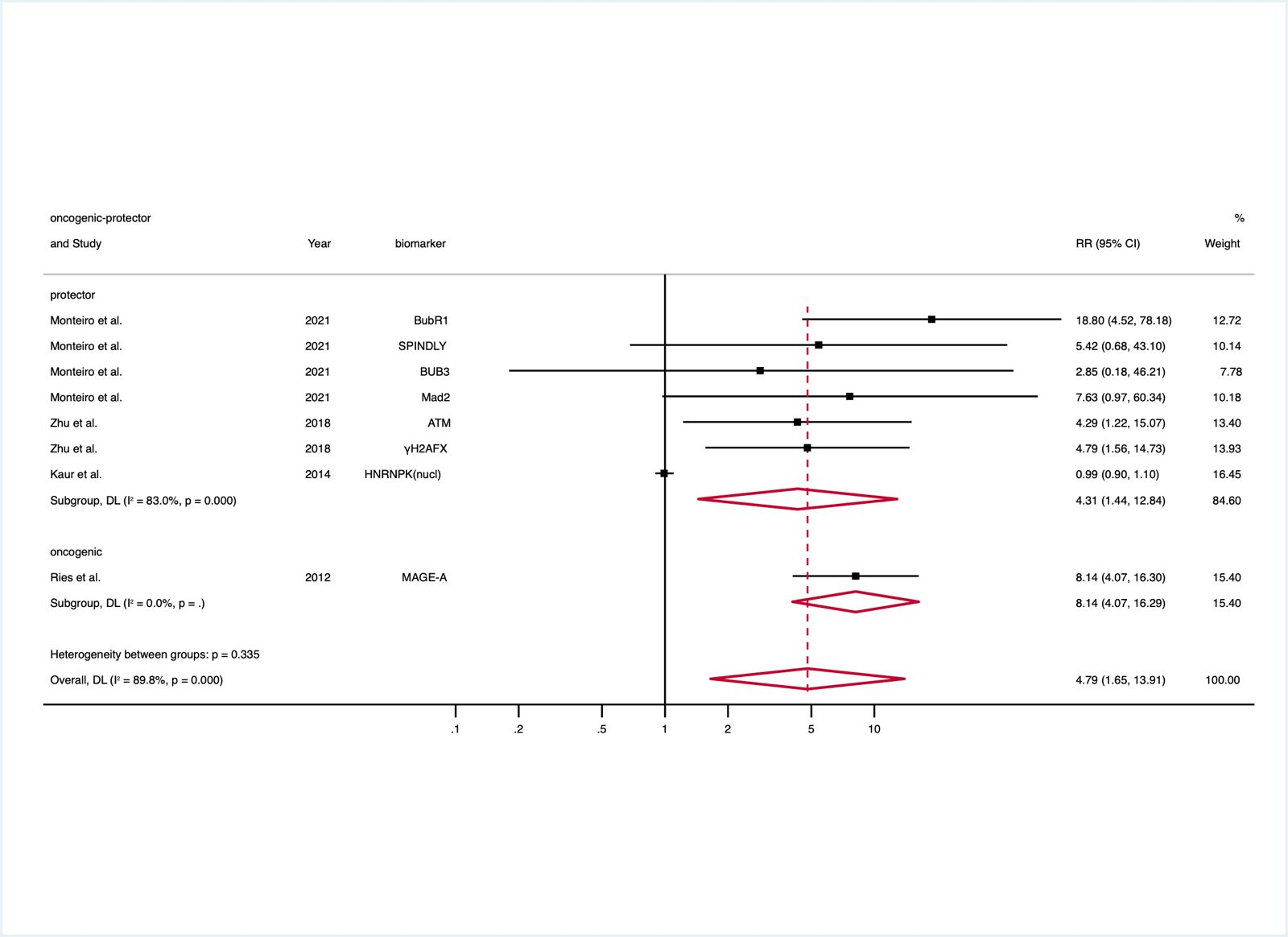


**Figure S23.** Forest plot graphically representing the differential expression of biomarkers on the hallmark genome instability and mutation -using pooled proportions as ES metric, expressed as percentage- among OL patients. ES, effect size; CI, confidence interval; Random-effects model.


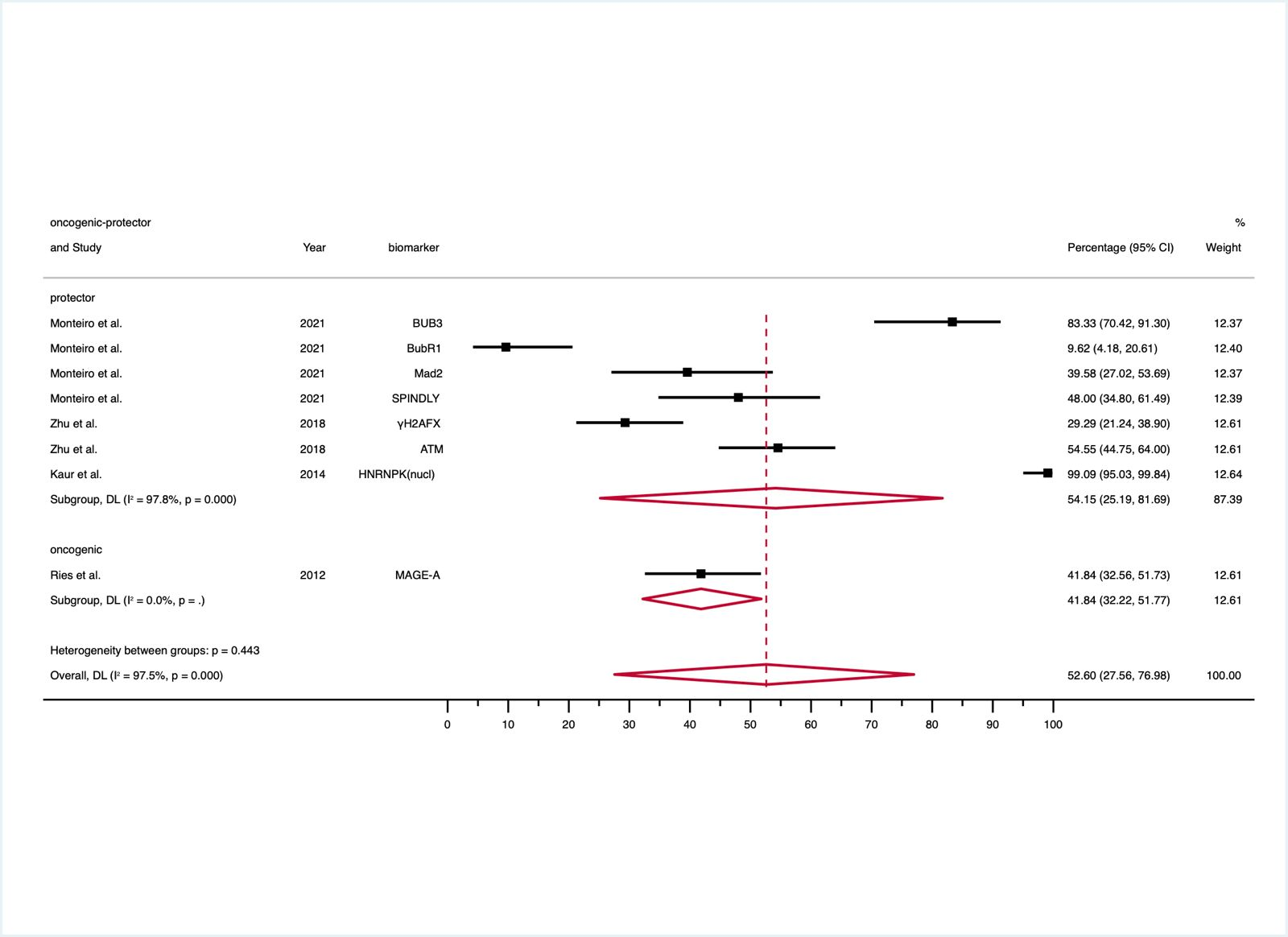


**Figure S24.** Forest plot graphically representing the meta-analysis of the magnitude of association -using OR as effect size metric- in order to compare the differential expression of biomarkers on the hallmark genome instability and mutation between OL and healthy controls. OR, odds ratio; CI, confidence interval; Random-effects model, inverse-variance weighting based on the DL method. A OR> 1 suggests a higher expression in OL in comparison to healthy oral mucosa. Diamonds indicate the pooled OR with their corresponding 95% CIs.


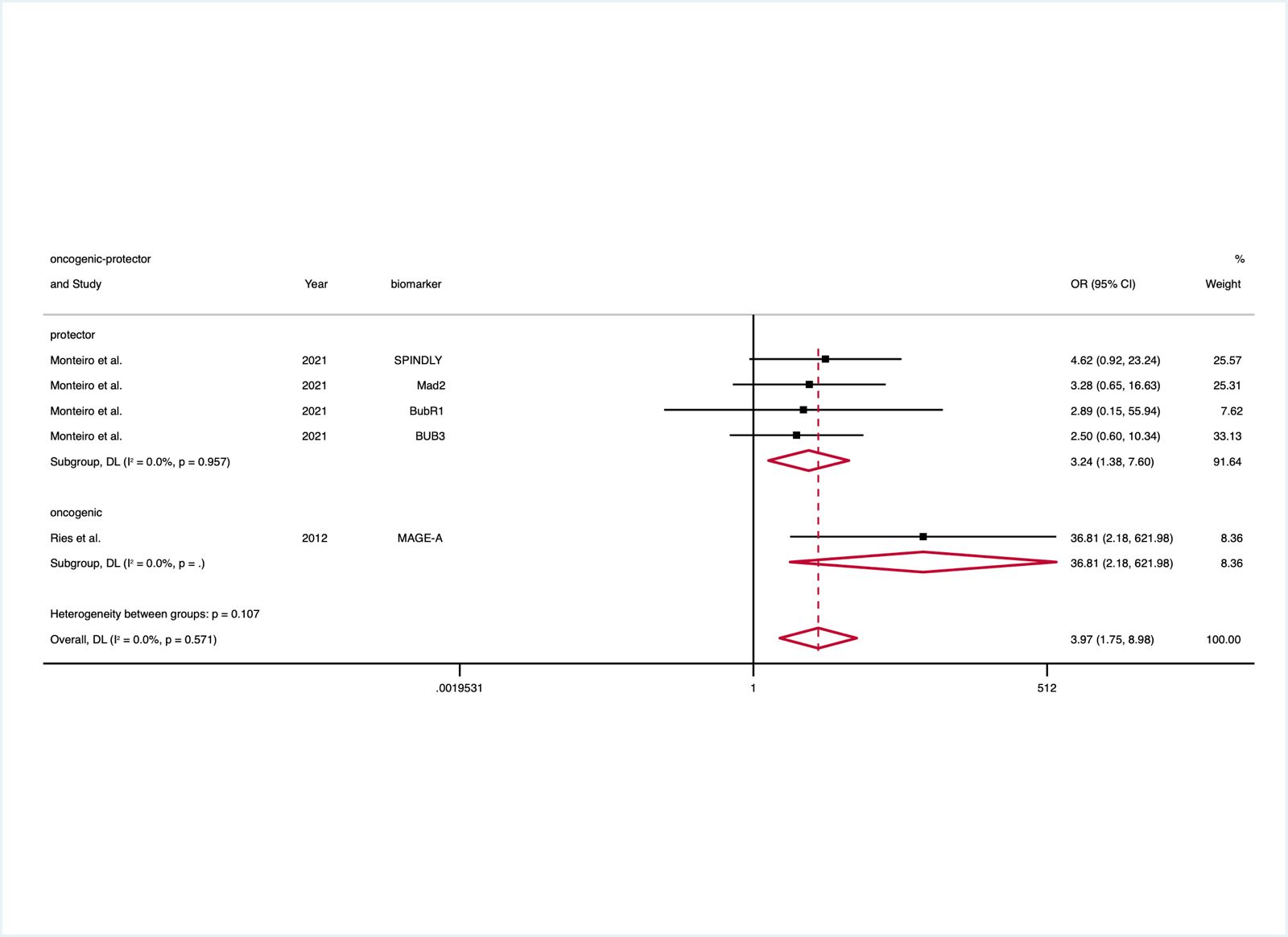


**3.10 Hallmark Tumor promoting inflammation**

**Figure S25.** Forest plot graphically representing the meta-analysis on the magnitude of association -using RR as effect size metric- between hallmark of cancer expression and OLs malignant transformation risk. RR, relative risk; CI, confidence intervals, DerSimonian and Laird, DL. Random-effects model, inverse-variance weighting based on the DL method. A RR > 1 suggests a higher malignant transformation risk. Diamonds indicate the pooled RR with their corresponding 95% CIs.


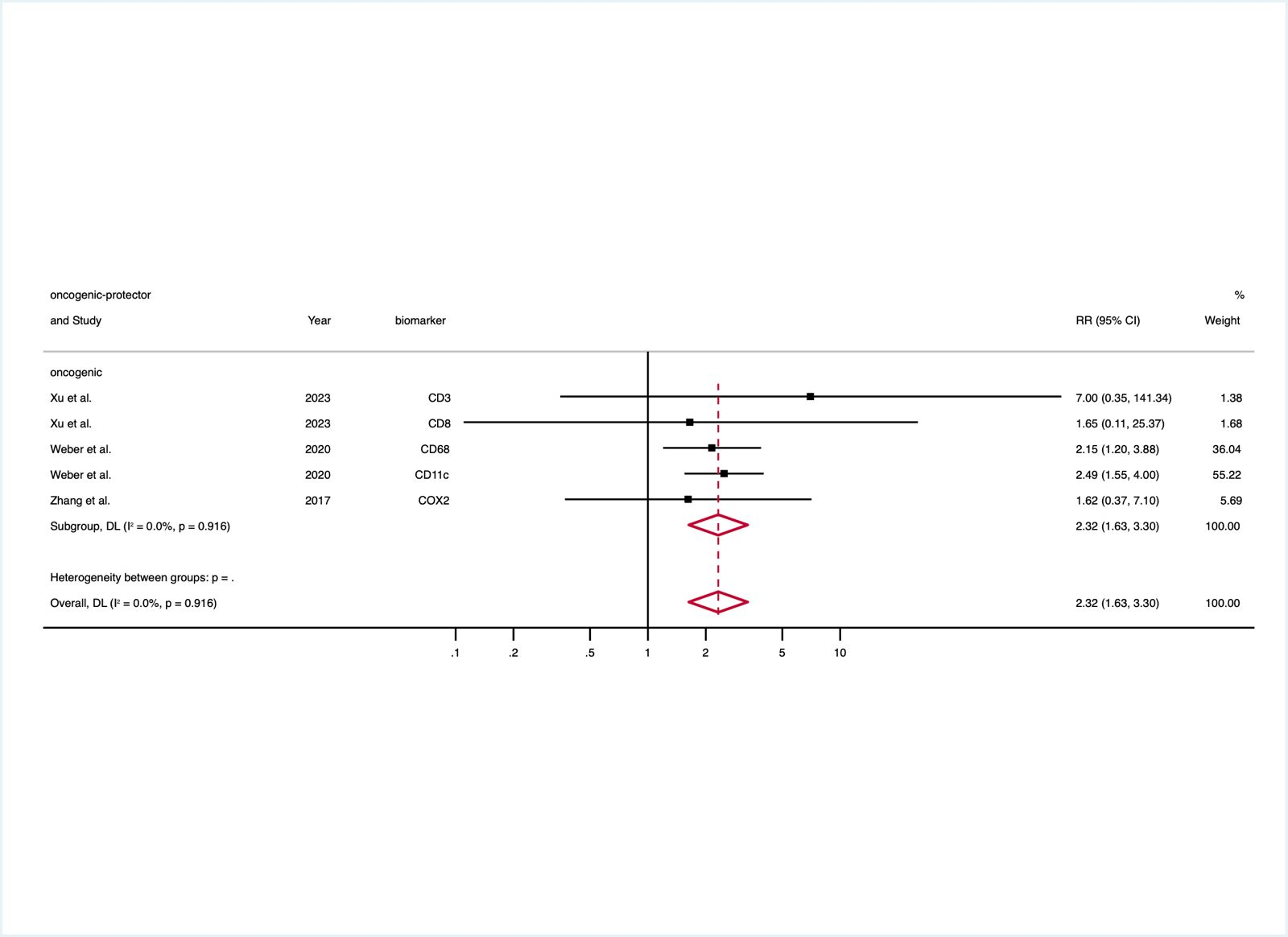


**Figure S26.** Forest plot graphically representing the differential expression of biomarkers on the hallmark tumor promoting inflammation -using pooled proportions as ES metric, expressed as percentage- among OL patients. ES, effect size; CI, confidence interval; Random-effects model.


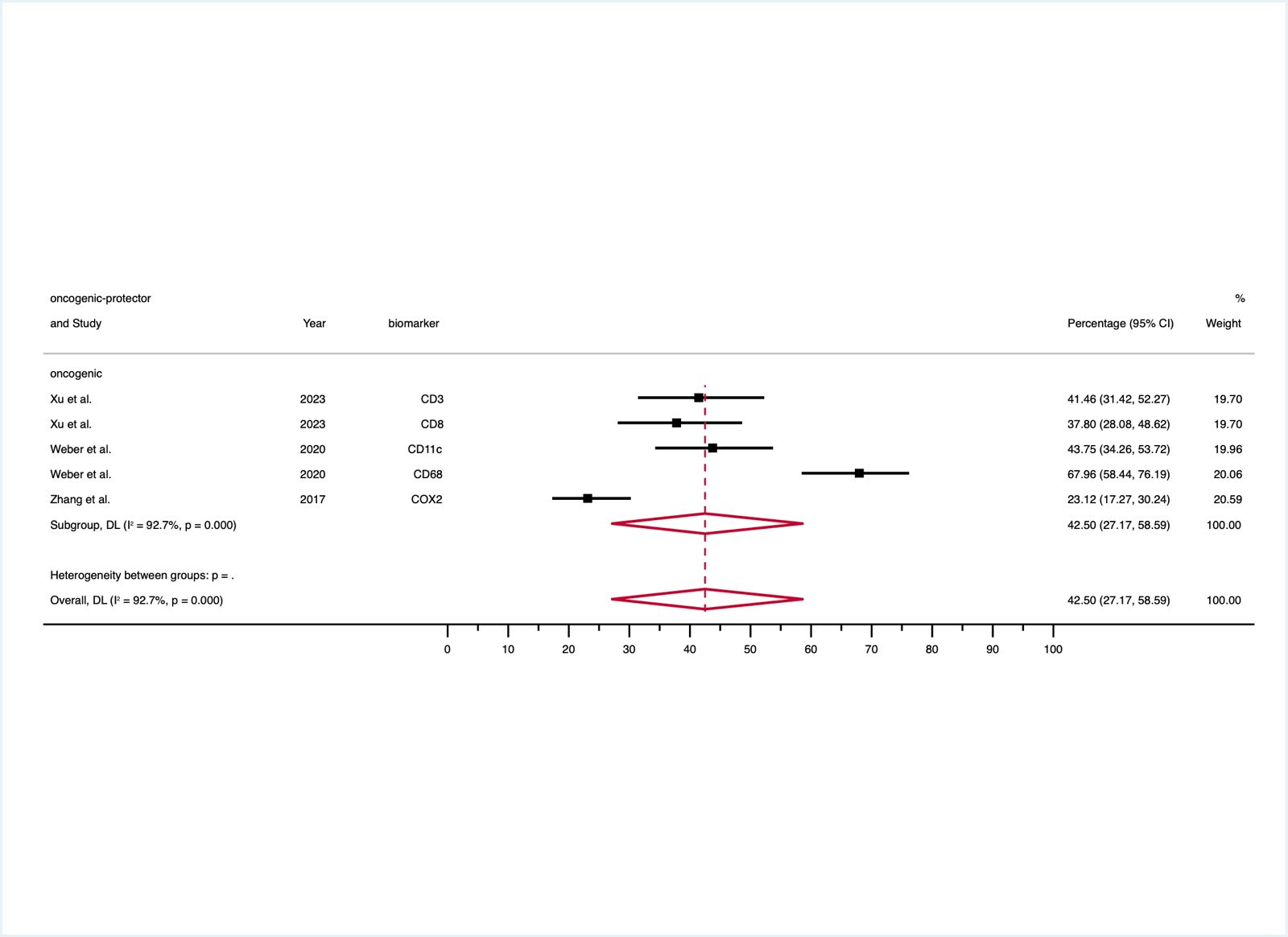


**Figure S27.** Forest plot graphically representing the meta-analysis of the magnitude of association -using OR as effect size metric- in order to compare the differential expression of biomarkers on the hallmark tumor promoting inflammation between OL and healthy controls. OR, odds ratio; CI, confidence interval; Random-effects model, inverse-variance weighting based on the DL method. A OR> 1 suggests a higher expression in OL in comparison to healthy oral mucosa. Diamonds indicate the pooled OR with their corresponding 95% CIs.


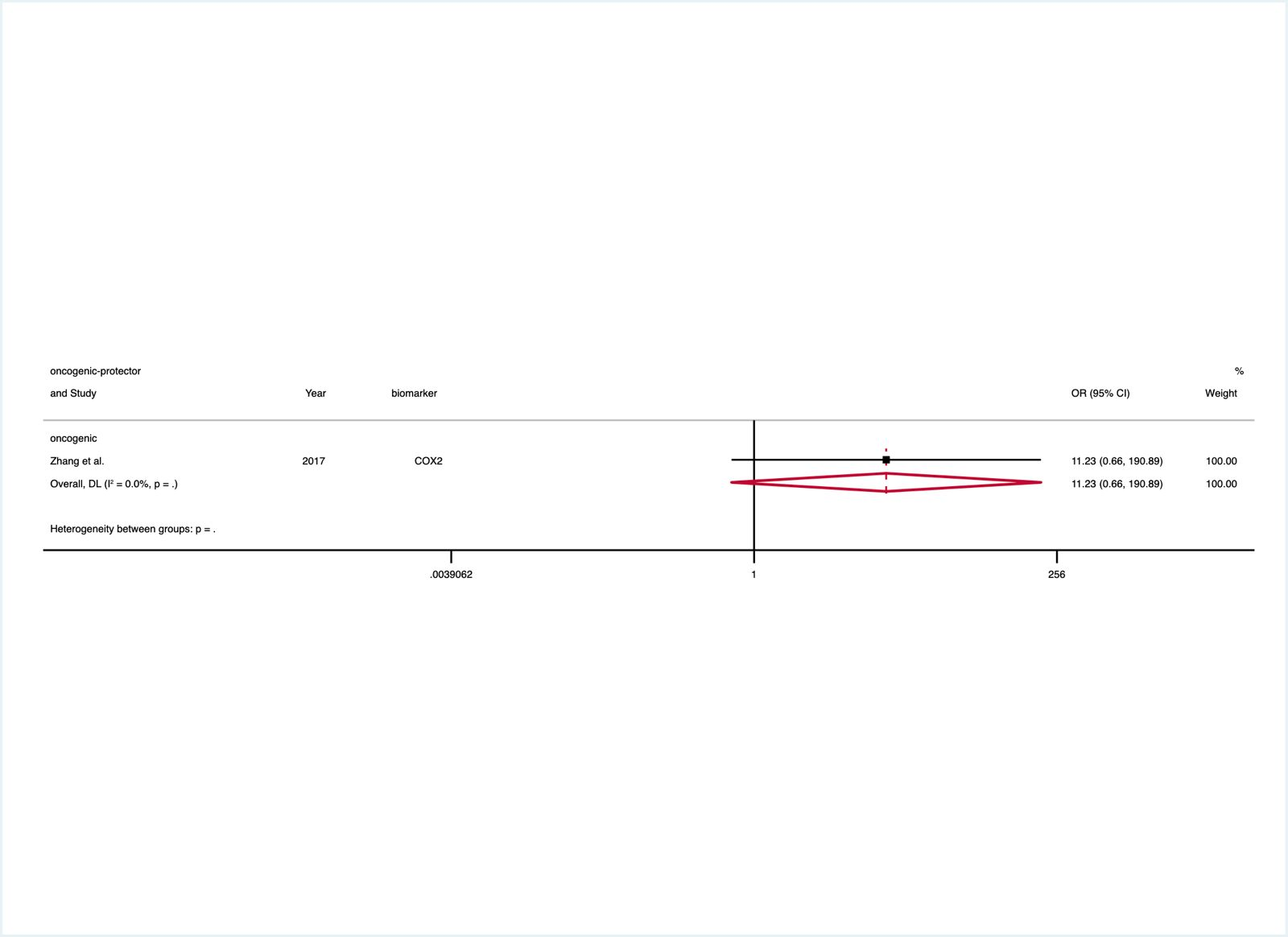


**4. Analysis of small‐study effects**

**Figure S28.** A funnel plot of estimated logRRs against their SEs, graphically representing the analysis of small-study effects on the association between the expression of the hallmark sustaining proliferative signaling and OL malignant transformation risk. SE, standard error; RR, relative risk; log, natural logarithm (i.e., log base e); OL, oral leukoplakia. The black vertical line corresponds to the pooled effect size estimated in the meta-analysis. The two diagonal intermittent lines represent the pseudo-95% confidence interval. The blue circles represent the estimates from primary-level studies.


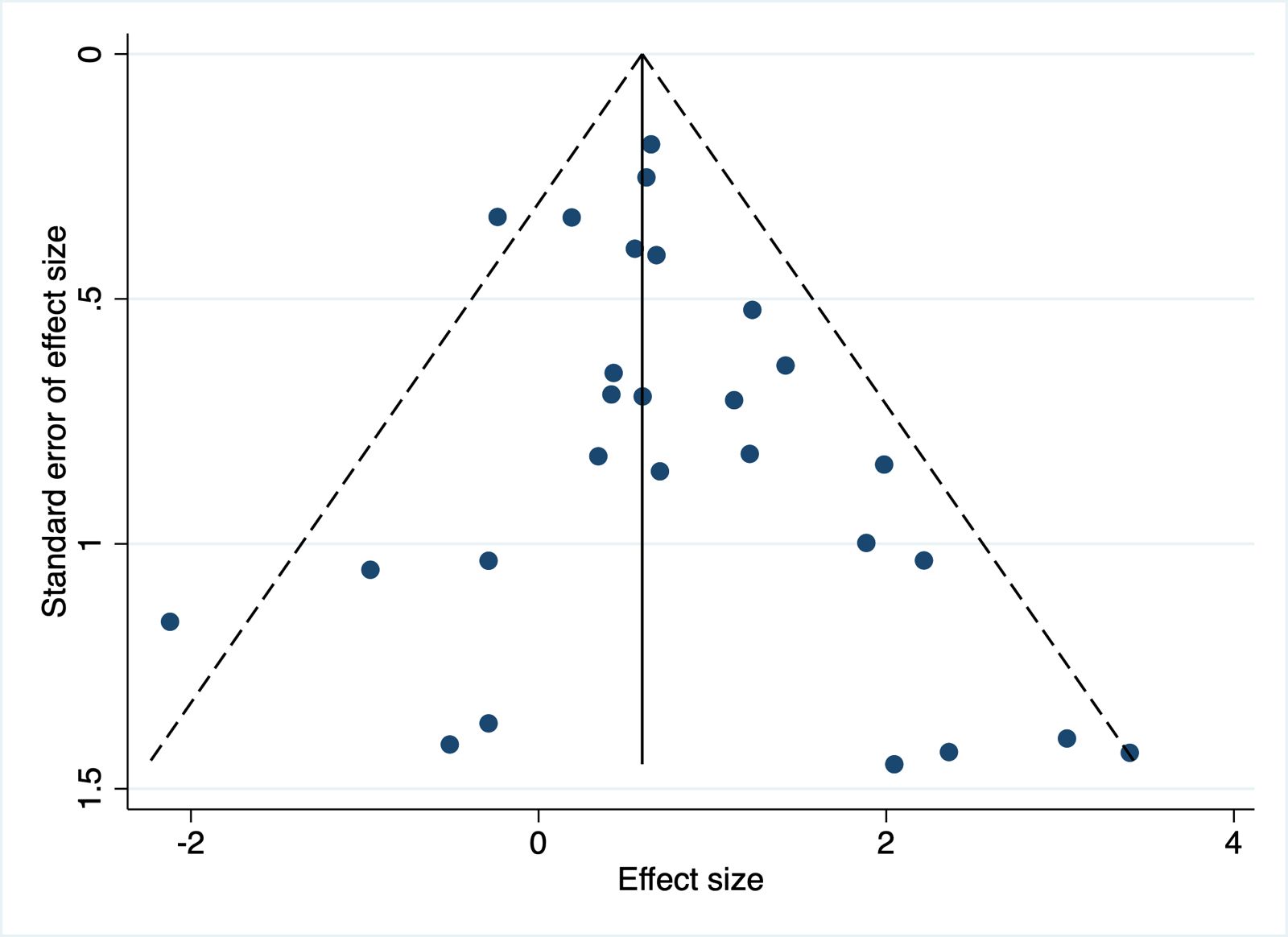


**Figure S29.** A funnel plot of estimated logRRs against their SEs, graphically representing the analysis of small-study effects on the association between the expression of the hallmark evading growth suppressors and OL malignant transformation risk. SE, standard error; RR, relative risk; log, natural logarithm (i.e., log base e); OL, oral leukoplakia. The black vertical line corresponds to the pooled effect size estimated in the meta-analysis. The two diagonal intermittent lines represent the pseudo-95% confidence interval. The blue circles represent the estimates from primary-level studies.

**
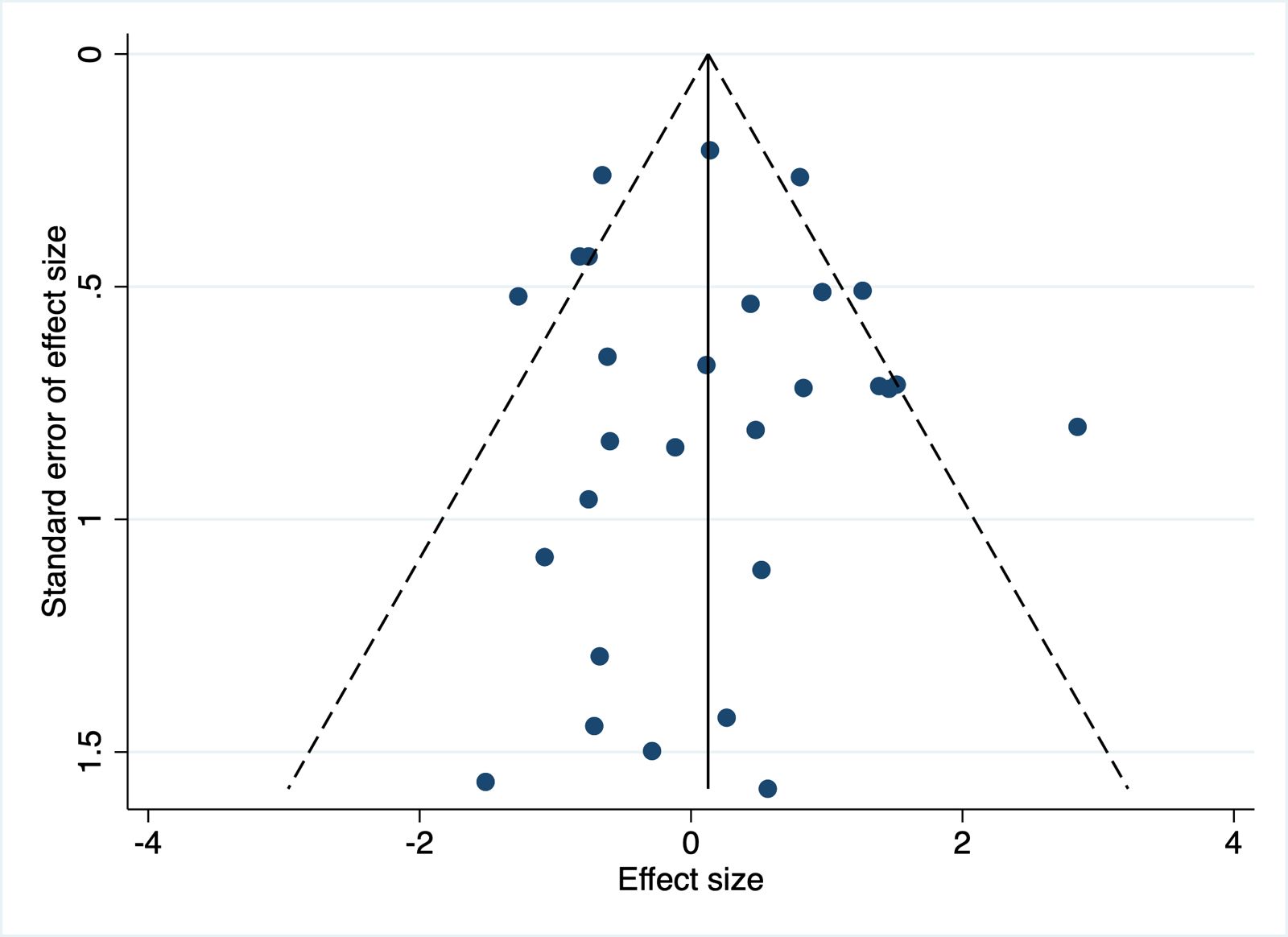
**

**Figure S30.** A funnel plot of estimated logRRs against their SEs, graphically representing the analysis of small-study effects on the association between the expression of the hallmark activating invasion and metastasis and OL malignant transformation risk. SE, standard error; RR, relative risk; log, natural logarithm (i.e., log base e); OL, oral leukoplakia. The black vertical line corresponds to the pooled effect size estimated in the meta-analysis. The two diagonal intermittent lines represent the pseudo-95% confidence interval. The blue circles represent the estimates from primary-level studies.


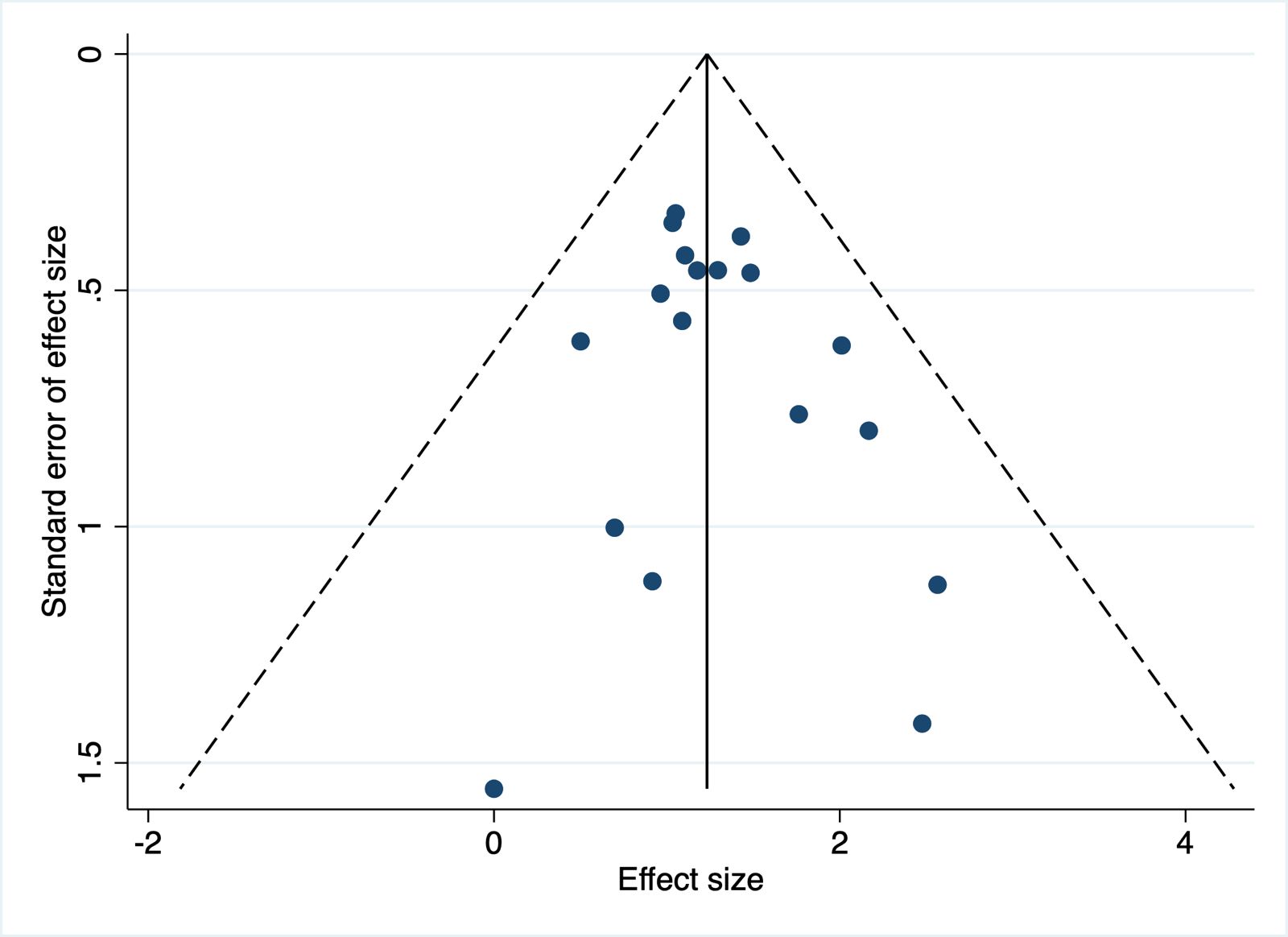


**5. Meta-analysis on the expression of hallmarks of cancer in oral leukoplakia stratified by geographical area**

| **Table-S3. Meta-analysis on the expression of hallmarks of cancer in oral leukoplakia stratified by geographical area** | | | | | | | | | | | |
| --- | --- | --- | --- | --- | --- | --- | --- | --- | --- | --- | --- |
|  |  |  |  |  | **Pooled data** | |  | **Heterogeneity** | | | |
| **Meta-analyses** | **No. of studies**^*^ | **No. of**  **cases**^*^ | **Stat. Model** | **Wt** | **ES (95% CI)** | ***P-value*** |  | ***P_het_*** | | ***I^2^***  **(%)** | |
| **Hallmark 1: Sustaining proliferative signaling** | | | | | | | | | | | |
| Expression of hallmarks of cancer and OL malignant transformation probability | | | | | | | | | | | |
| Oncogenic (pro-proliferative) | 27 | 1,949 | REM | D-L | RR= 1.92 (1.45 - 2.55) | <0.001 |  | | 0.06 | | 31.9 |
| Subgroup meta-analysis by geographical area | | | | | | 0.45 |  | |  | |  |
| Asian subgroup | 12 | 992 | REM | D-L | RR=2.26 (1.29 - 3.99) | 0.005 |  | | 0.08 | | 39.6 |
| Non-Asian subgroup | 15 | 957 | REM | D-L | RR=1.76 (1.27 - 2.42) | 0.001 |  | | 0.16 | | 31.9 |
| Differential expression in OL | | | | | | | | | | | |
| Oncogenic (pro-proliferative) | 26 | 1,919 | REM | D-L | PP= 56.30% (43.10-69.09) | — |  | | <0.001 | | 97.0 |
| Subgroup meta-analysis by geographical area | | | | | | 0.68 |  | |  | |  |
| Asian subgroup | 12 | 992 | REM | D-L | PP=53.27% (30.67-75.20) | — |  | | <0.001 | | 98.1 |
| Non-Asian subgroup | 14 | 927 | REM | D-L | PP=59.04% (44.84-72.54) | — |  | | <0.001 | | 94.5 |
| Comparison between expression in OL vs healthy controls | | | | | | | | | | | |
| Oncogenic (pro-proliferative) | 5 | 529 | REM | D-L | OR= 7.70 (2.22-26.65) | 0.001 |  | | 0.62 | | 0.0 |
| Subgroup meta-analysis by geographical area | | | | | | 0.81 |  | |  | |  |
| Asian subgroup | 2 | 356 | REM | D-L | OR= 6.39 (0.85-47.71) | 0.07 |  | | 0.0 | | 0.74 |
| Non-Asian subgroup | 3 | 173 | REM | D-L | OR= 8.95 (1.54-52.12) | 0.02 |  | | 19.2 | | 0.29 |
| **Hallmark 2: Evading growth suppressors** | | | | | | | | | | | |
| Expression of hallmarks of cancer and OL malignant transformation probability | | | | | | | | | | | |
| Protector (growth suppressor) | 28 | 1,932 | REM | D-L | RR= 1.21 (0.84-1.75) | 0.31 |  | | <0.001 | | 60.9 |
| Subgroup meta-analysis by geographical area | | | | | | 0.22 |  | |  | |  |
| Asian subgroup | 10 | 883 | REM | D-L | RR= 1.65 (0.77-3.53) | 0.20 |  | | <0.001 | | 80.3 |
| Non-Asian subgroup | 18 | 1049 | REM | D-L | RR= 0.98 (0.69-1.38) | 0.91 |  | | 0.21 | | 20.3 |
| Differential expression in OL | | | | | | | | | | | |
| Protector (growth suppressor) | 27 | 1,842 | REM | D-L | PP= 51.27% (41.63-60.87) | — |  | | <0.001 | | 94.0 |
| Subgroup meta-analysis by geographical area | | | | | | 0.71 |  | |  | |  |
| Asian subgroup | 9 | 793 | REM | D-L | PP= 48.66% (35.49-61.92) | — |  | | <0.001 | | 92.1 |
| Non-Asian subgroup | 18 | 1049 | REM | D-L | PP= 52.30% (39.02. 65.42) | — |  | | <0.001 | | 94.6 |
| Comparison between expression in OL vs healthy controls | | | | | | | | | | | |
| Protector (growth suppressor) | 10 | 702 | REM | D-L | OR=5.58 (2.47-12.58) | <0.001 |  | | 0.21 | | 25.5 |
| Subgroup meta-analysis by geographical area | | | | | | 0.51 |  | |  | |  |
| Asian subgroup | 4 | 400 | REM | D-L | OR=4.04 (1.30-12.56) | 0.01 |  | | 0.56 | | 0.0 |
| Non-Asian subgroup | 6 | 302 | REM | D-L | OR=7.17 (2.02-25.45) | 0.002 |  | | 0.08 | | 48.6 |
| **Hallmark 3: Resisting cell death** | | | | | | | | | | | |
| Expression of hallmarks of cancer and OL malignant transformation probability | | | | | | | | | | | |
| Oncogenic (anti-apopotic) | 8 | 625 | REM | D-L | RR=0.94 (0.43-2.03) | 0.87 |  | | <0.001 | | 81.1 |
| Subgroup meta-analysis by geographical area | | | | | | <0.001 |  | |  | |  |
| Asian subgroup | 3 | 138 | REM | D-L | RR=0.10 (0.03, 0.39) | 0.001 |  | | 0.69 | | 0.0 |
| Non-Asian subgroup | 5 | 487 | REM | D-L | RR=1.69 (0.84. 3.41) | 0.14 |  | | <0.001 | | 80.4 |
| Differential expression in OL | | | | | | | | | | | |
| Oncogenic (anti-apopotic) | 8 | 625 | REM | D-L | PP=69.11% (39.36-92.37) | — |  | | <0.001 | | 98.1 |
| Subgroup meta-analysis by geographical area | | | | | | <0.001 |  | |  | |  |
| Asian subgroup | 3 | 138 | REM | D-L | PP=99.86% (93.50-100.00) | — |  | | 0.14 | | 50.0 |
| Non-Asian subgroup | 5 | 487 | REM | D-L | PP=44.73% (20.54-70.30) | — |  | | <0.001 | | 97.0 |
| Comparison between expression in OL vs healthy controls | | | | | | | | | | | |
| Oncogenic (anti-apopotic) | 2 | 44 | REM | D-L | OR= 6.17 (0.85-44.84) | 0.07 |  | | 0.48 | | 0.0 |
| Subgroup meta-analysis by geographical area | | | | | | — |  | |  | |  |
| Asian subgroup | 2 | 44 | REM | D-L | OR= 6.17 (0.85-44.84) | 0.07 |  | | 0.48 | | 0.0 |
| Non-Asian subgroup | 0 | 0 | REM | D-L | — | — |  | | — | | — |
| **Hallmark 4: Enabling replicative immortality** | | | | | | | | | | | |
| Expression of hallmarks of cancer and OL malignant transformation probability | | | | | | | | | | | |
| Oncogenic (immortalization) | 2 | 244 | REM | D-L | RR= 3.74 (1.94-7.21) | <0.001 |  | | 0.83 | | 0.0 |
| Subgroup meta-analysis by geographical area | | | | | | — |  | |  | |  |
| Asian subgroup | 2 | 244 | REM | D-L | RR= 3.74 (1.94-7.21) | <0.001 |  | | 0.83 | | 0.0 |
| Non-Asian subgroup | 0 | 0 | REM | D-L | — | — |  | | — | | — |
| Differential expression in OL | | | | | | | | | | | |
| Oncogenic (immortalization) | 2 | 244 | REM | D-L | PP= 41.26% (24.56-59.04) | — |  | | 0.01 | | 87.4 |
| Subgroup meta-analysis by geographical area | | | | | | — |  | |  | |  |
| Asian subgroup | 2 | 244 | REM | D-L | PP= 41.26% (24.56-59.04) | — |  | | 0.01 | | 87.4 |
| Non-Asian subgroup | 0 | 0 | REM | D-L | — | — |  | | — | | — |
| Comparison between expression in OL vs healthy controls | | | | | | | | | | | |
| Oncogenic (immortalization) | 0 | 0 | REM | D-L | — | — |  | | — | | — |
| Subgroup meta-analysis by geographical area | | | | | |  |  | |  | |  |
| Asian subgroup | 0 | 0 | REM | D-L | — | — |  | | — | | — |
| Non-Asian subgroup | 0 | 0 | REM | D-L | — | — |  | | — | | — |
| **Hallmark 5: Inducing angiogenesis** | | | | | | | | | | | |
| Expression of hallmarks of cancer and OL malignant transformation probability | | | | | | | | | | | |
| Oncogenic (pro-angiogenic) | 2 | 280 | REM | D-L | RR=3.74 (1.79-7.82) | <0.001 |  | | 0.91 | | 0.0 |
| Subgroup meta-analysis by geographical area | | | | | | 0.91 |  | |  | |  |
| Asian subgroup | 1 | 160 | REM | D-L | RR=3.51 (0.91-13.53) | 0.07 |  | | — | | 0.0 |
| Non-Asian subgroup | 1 | 120 | REM | D-L | RR=3.84 (1.59-9.27) | 0.003 |  | | — | | 0.0 |
| Differential expression in OL | | | | | | | | | | | |
| Oncogenic (pro-angiogenic) | 2 | 280 | REM | D-L | PP= 43.80% (32.41-55.52) | — |  | | 0.05 | | 74.4 |
| Subgroup meta-analysis by geographical area | | | | | | 0.05 |  | |  | |  |
| Asian subgroup | 1 | 160 | REM | D-L | PP=38.12% (30.73-45.80) | — |  | | — | | 0.0 |
| Non-Asian subgroup | 1 | 120 | REM | D-L | PP=50.00% (41.05-58.95) | — |  | | — | | 0.0 |
| Comparison between expression in OL vs healthy controls | | | | | | | | | | | |
| Oncogenic (pro-angiogenic) | 0 | 0 | REM | D-L | — | — |  | | — | | — |
| Subgroup meta-analysis by geographical area | | | | | | — |  | |  | |  |
| Asian subgroup | 0 | 0 | REM | D-L | — | — |  | | — | | — |
| Non-Asian subgroup | 0 | 0 | REM | D-L | — | — |  | | — | | — |
| **Hallmark 6: Activating invasion and metastasis** | | | | | | | | | | | |
| Expression of hallmarks of cancer and OL malignant transformation probability | | | | | | | | | | | |
| Oncogenic (pro-invasive) | 18 | 1,602 | REM | D-L | RR=3.43 (2.67-4.40) | <0.001 |  | | 0.92 | | 0.0 |
| Subgroup meta-analysis by geographical area | | | | | | 0.99 |  | |  | |  |
| Asian subgroup | 9 | 1073 | REM | D-L | RR=3.43 (2.52-4.66) | <0.001 |  | | 0.90 | | 0.0 |
| Non-Asian subgroup | 9 | 529 | REM | D-L | RR=3.43 (2.26. 5.23) | <0.001 |  | | 0.63 | | 0.0 |
| Differential expression in OL | | | | | | | | | | | |
| Oncogenic (pro-invasive) | 18 | 1,602 | REM | D-L | PP=37.30% (28.21-46.86) | — |  | | <0.001 | | 93.2 |
| Subgroup meta-analysis by geographical area | | | | | | 0.48 |  | |  | |  |
| Asian subgroup | 9 | 1073 | REM | D-L | PP=40.82% (28.33-53.93) | — |  | | <0.001 | | 94.6 |
| Non-Asian subgroup | 9 | 529 | REM | D-L | PP=33.61% (19.69-49.09) | — |  | | <0.001 | | 93.2 |
| Comparison between expression in OL vs healthy controls | | | | | | | | | | | |
| Oncogenic (pro-invasive) | 6 | 674 | REM | D-L | OR= 2.20 (0.39-12.42) | 0.37 |  | | <0.001 | | 84.5 |
| Subgroup meta-analysis by geographical area | | | | | | 0.06 |  | |  | |  |
| Asian subgroup | 4 | 567 | REM | D-L | OR=6.44 (1.84-22.56) | 0.004 |  | | 0.14 | | 44.6 |
| Non-Asian subgroup | 2 | 107 | REM | D-L | OR=0.25 (0.01-5.78) | 0.39 |  | | 0.002 | | 89.3 |
| **Hallmark 7: Avoiding immune destruction** | | | | | | | | | | | |
| Expression of hallmarks of cancer and OL malignant transformation probability | | | | | | | | | | | |
| Oncogenic (Anti-tumour arrest) | 7 | 810 | REM | D-L | RR= 3.65 (1.87-7.13) | <0.001 |  | | 0.10 | | 43.9 |
| Subgroup meta-analysis by geographical area | | | | | | 0.07 |  | |  | |  |
| Asian subgroup | 6 | 714 | REM | D-L | RR=4.78 (2.35-9.70) | <0.001 |  | | 0.27 | | 21.5 |
| Non-Asian subgroup | 1 | 96 | REM | D-L | RR=2.04 (1.12-3.71) | 0.02 |  | | — | | 0.0 |
| Differential expression in OL | | | | | | | | | | | |
| Oncogenic (Anti-tumour arrest) | 7 | 810 | REM | D-L | PP=35.77% (24.66-47.69) | — |  | | <0.001 | | 91.5 |
| Subgroup meta-analysis by geographical area | | | | | | <0.001 |  | |  | |  |
| Asian subgroup | 6 | 714 | REM | D-L | PP=31.15% (22.67-40.30) | — |  | | <0.001 | | 84.4 |
| Non-Asian subgroup | 1 | 96 | REM | D-L | PP=65.62% (55.79-74.84) | — |  | | — | | 0.0 |
| Comparison between expression in OL vs healthy controls | | | | | | | | | | | |
| Oncogenic (Anti-tumour arrest) | 0 | 0 | REM | D-L | — | — |  | | — | | — |
| Subgroup meta-analysis by geographical area | | | | | | — |  | |  | |  |
| Asian subgroup | 0 | 0 | REM | D-L | — | — |  | | — | | — |
| Non-Asian subgroup | 0 | 0 | REM | D-L | — | — |  | | — | | — |
| **Hallmark 8: Deregulating cellular energetics** | | | | | | | | | | | |
| Expression of hallmarks of cancer and OL malignant transformation probability | | | | | | | | | | | |
| Oncogenic (tumour acidosis) | 1 | 160 | REM | D-L | RR= 4.57 (0.99-21.03) | 0.051 |  | | — | | 0.0 |
| Subgroup meta-analysis by geographical area | | | | | | — |  | |  | |  |
| Asian subgroup | 1 | 160 | REM | D-L | RR= 4.57 (0.99-21.03) | 0.051 |  | | — | | 0.0 |
| Non-Asian subgroup | 0 | 0 | REM | D-L | — | — |  | | — | | — |
| Differential expression in OL | | | | | | | | | | | |
| Oncogenic (tumour acidosis) | 1 | 160 | REM | D-L | PP= 42.50% (34.92-50.21) | — |  | | — | | 0.0 |
| Subgroup meta-analysis by geographical area | | | | | | — |  | |  | |  |
| Asian subgroup | 1 | 160 | REM | D-L | PP= 42.50% (34.92-50.21) | — |  | | — | | 0.0 |
| Non-Asian subgroup | 0 | 0 | REM | D-L | — | — |  | | — | | — |
| Comparison between expression in OL vs healthy controls | | | | | | | | | | | |
| Oncogenic (tumour acidosis) | 1 | 178 | REM | D-L | OR= 27.40 (1.62-462.61) | 0.02 |  | | — | | 0.0 |
| Subgroup meta-analysis by geographical area | | | | | | — |  | |  | |  |
| Asian subgroup | 1 | 178 | REM | D-L | OR= 27.40 (1.62-462.61) | 0.02 |  | | — | | 0.0 |
| Non-Asian subgroup | 0 | 0 | REM | D-L | — | — |  | | — | | — |
| **Hallmark 9: Genome instability and mutation** | | | | | | | | | | | |
| Expression of hallmarks of cancer and OL malignant transformation probability | | | | | | | | | | | |
| Oncogenic (DNA instability) | 1 | 98 | REM | D-L | RR= 8.14 (4.07-16.29) | 0.009 |  | | — | | 0.0 |
| Subgroup meta-analysis by geographical area | | | | | | — |  | |  | |  |
| Asian subgroup | 0 | 0 | REM | D-L | — | — |  | | — | | — |
| Non-Asian subgroup | 1 | 98 | REM | D-L | RR= 8.14 (4.07-16.29) | 0.009 |  | | — | | 0.0 |
| Protector (DNA damage repair) | 7 | 506 | REM | D-L | RR= 4.31 (1.44-12.84) | <0.001 |  | | <0.001 | | 83.0 |
| Subgroup meta-analysis by geographical area | | | | | | 0.96 |  | |  | |  |
| Asian subgroup | 2 | 198 | REM | D-L | RR=4.56 (1.97-10.54) | <0.001 |  | | 0.90 | | 0.0 |
| Non-Asian subgroup | 5 | 308 | REM | D-L | RR=4.36 (0.93-20.46) | 0.06 |  | | <0.001 | | 82.6 |
| Differential expression in OL | | | | | | | | | | | |
| Oncogenic (DNA instability) | 1 | 98 | REM | D-L | PP= 41.84% (32.22-51.77) | — |  | | — | | 0.0 |
| Subgroup meta-analysis by geographical area | | | | | | — |  | |  | |  |
| Asian subgroup | 0 | 0 | REM | D-L | — | — |  | | — | | — |
| Non-Asian subgroup | 1 | 98 | REM | D-L | PP= 41.84% (32.22-51.77) | — |  | | — | | 0.0 |
| Protector (DNA damage repair) | 7 | 506 | REM | D-L | PP= 54.15% (25.19-81.69) | — |  | | <0.001 | | 97.8 |
| Subgroup meta-analysis by geographical area | | | | | | 0.49 |  | |  | |  |
| Asian subgroup | 2 | 198 | REM | D-L | PP=41.65% (18.70-66.66) | — |  | | <0.001 | | 92.4 |
| Non-Asian subgroup | 5 | 308 | REM | D-L | PP=59.16% (18.71-93.47) | — |  | | <0.001 | | 98.2 |
| Comparison between expression in OL vs healthy controls | | | | | | | | | | | |
| Oncogenic (DNA instability) | 1 | 123 | REM | D-L | OR= 36.81 (2.18-621.98) | 0.01 |  | | — | | 0.0 |
| Subgroup meta-analysis by geographical area | | | | | | — |  | |  | |  |
| Asian subgroup | 0 | 0 | REM | D-L | — | — |  | | — | | — |
| Non-Asian subgroup | 1 | 123 | REM | D-L | OR= 36.81 (2.18-621.98) | 0.01 |  | | — | | 0.0 |
| Protector (DNA damage repair) | 4 | 246 | REM | D-L | OR= 3.24 (1.38-7.60) | 0.007 |  | | 0.96 | | 0.0 |
| Subgroup meta-analysis by geographical area | | | | | | — |  | |  | |  |
| Asian subgroup | 0 | 0 | REM | D-L | — | — |  | | — | | — |
| Non-Asian subgroup | 4 | 246 | REM | D-L | OR= 3.24 (1.38-7.60) | 0.007 |  | | 0.96 | | 0.0 |
| **Hallmark 10: Tumor promoting inflammation** | | | | | | | | | | | |
| Expression of hallmarks of cancer and OL malignant transformation probability | | | | | | | | | | | |
| Oncogenic (pro-inflammatory) | 5 | 523 | REM | D-L | RR= 2.32 (1.63-3.30) | <0.001 |  | | 0.92 | | 0.0 |
| Subgroup meta-analysis by geographical area | | | | | | 0.83 |  | |  | |  |
| Asian subgroup | 3 | 324 | REM | D-L | RR=2.05 (0.62-6.74) | 0.24 |  | | 0.68 | | 0.0 |
| Non-Asian subgroup | 2 | 199 | REM | D-L | RR=2.35 (1.63-3.40) | <0.001 |  | | 0.70 | | 0.0 |
| Differential expression in OL | | | | | | | | | | | |
| Oncogenic (pro-inflammatory) | 5 | 523 | REM | D-L | PP= 42.50 % (27.17-58.59) | — |  | | <0.001 | | 92.7 |
| Subgroup meta-analysis by geographical area | | | | | | 0.99 |  | |  | |  |
| Asian subgroup | 3 | 324 | REM | D-L | PP=33.41% (21.78-46.13) | — |  | | 0.005 | | 81.1 |
| Non-Asian subgroup | 2 | 199 | REM | D-L | PP=56.07% (32.33-78.46) | — |  | | 0.001 | | 91.6 |
| Comparison between expression in OL vs healthy controls | | | | | | | | | | | |
| Oncogenic (pro-inflammatory) | 1 | 178 | REM | D-L | OR= 11.23 (0.66-190.89) | 0.09 |  | | — | | 0.0 |
| Subgroup meta-analysis by geographical area | | | | | |  |  | |  | |  |
| Asian subgroup | 1 | 178 | REM | D-L | OR= 11.23 (0.66-190.89) | 0.09 |  | | — | | 0.0 |
| Non-Asian subgroup | 0 | 0 | REM | D-L | — | — |  | | — | | — |
| Abbreviations: Stat., statistical; Wt, method of weighting; PP, pooled proportion; RR, relative risk; OR, odds ratio; CI, confidence intervals; REM, random-effects model; D-L, DerSimonian and Laird method; OL, leukoplakia.  *- Note that more than one analysis unit was analyzed per study and patient. | | | | | | | | | | | |

**5.1 Hallmark Sustaining proliferative signaling stratified by geographical area**

**Figure S31.** Forest plot graphically representing the meta-analysis on the magnitude of association -using RR as effect size metric- between hallmark of cancer expression and OLs malignant transformation risk. RR, relative risk; CI, confidence intervals, DerSimonian and Laird, DL. Random-effects model, inverse-variance weighting based on the DL method. A RR > 1 suggests a higher malignant transformation risk. Diamonds indicate the pooled RR with their corresponding 95% CIs.


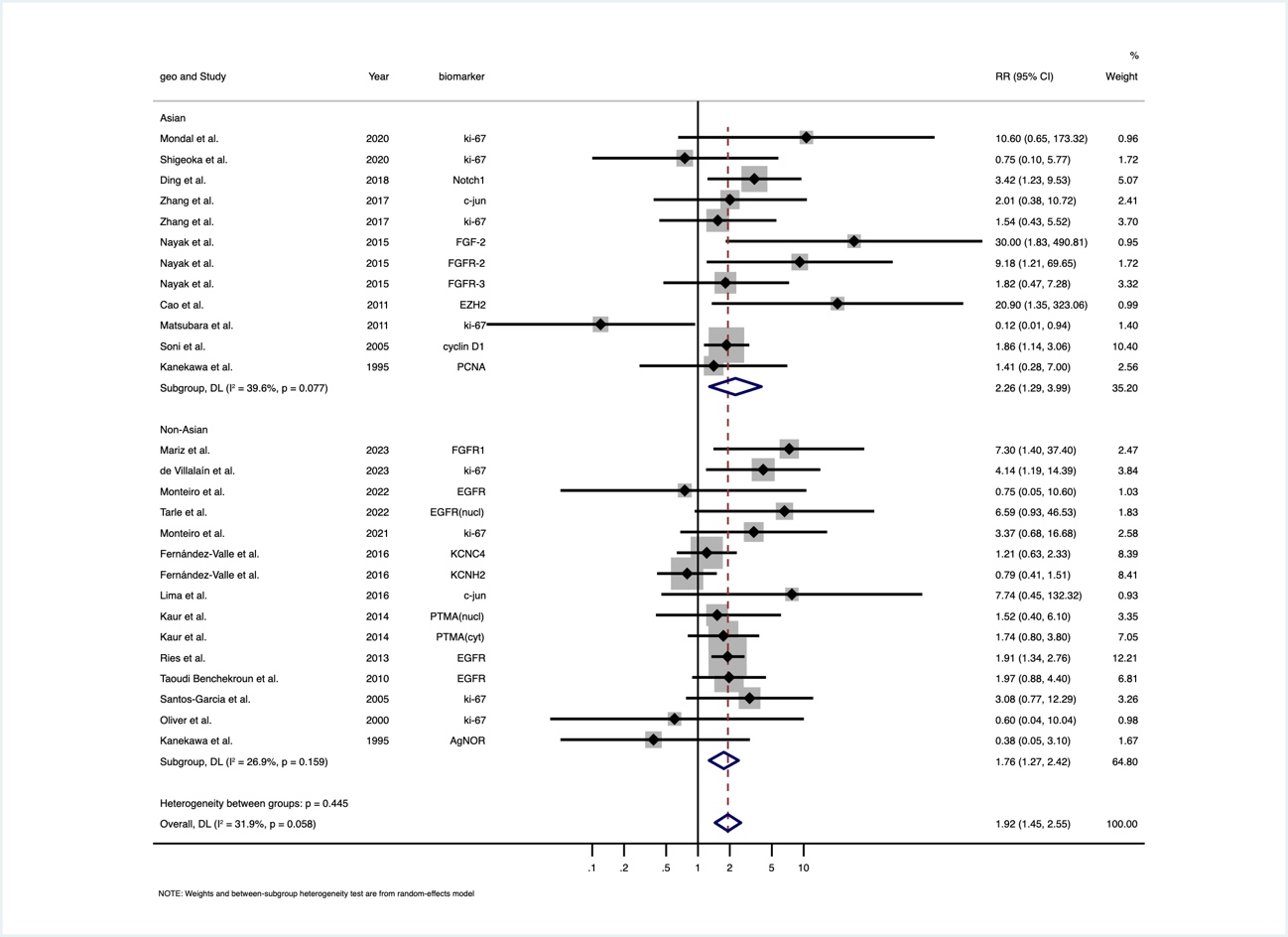


**Figure S32.** Forest plot graphically representing the differential expression of biomarkers on the hallmark sustaining proliferative signaling -using pooled proportions as ES metric, expressed as percentage- among OL patients. ES, effect size; CI, confidence interval; Random-effects model.


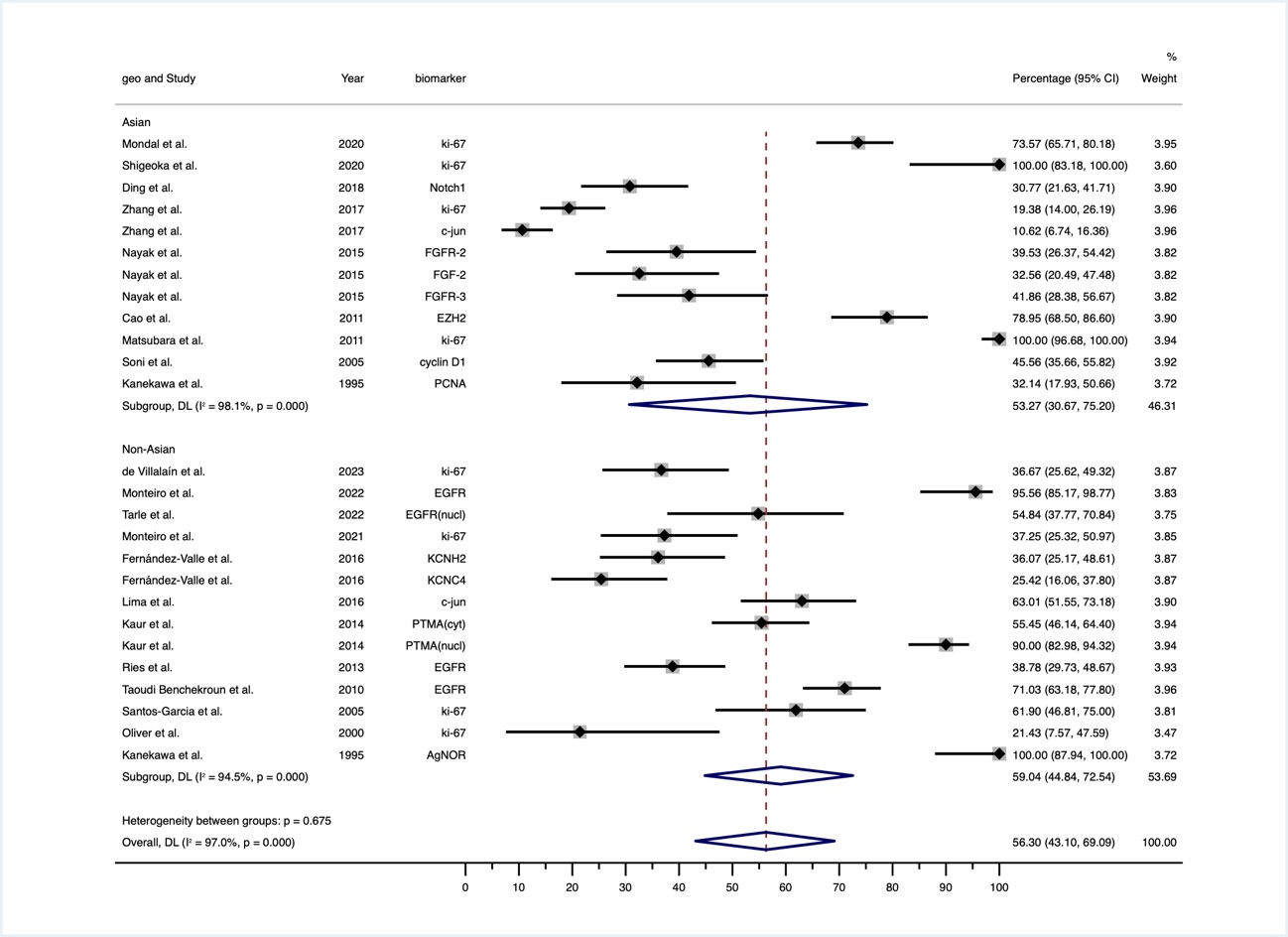


**Figure S33.** Forest plot graphically representing the meta-analysis of the magnitude of association -using OR as effect size metric- in order to compare the differential expression of biomarkers on the hallmark sustaining proliferative signaling between OL and healthy controls. OR, odds ratio; CI, confidence interval; Random-effects model, inverse-variance weighting based on the DL method. A OR> 1 suggests a higher expression in OL in comparison to healthy oral mucosa. Diamonds indicate the pooled OR with their corresponding 95% CIs.


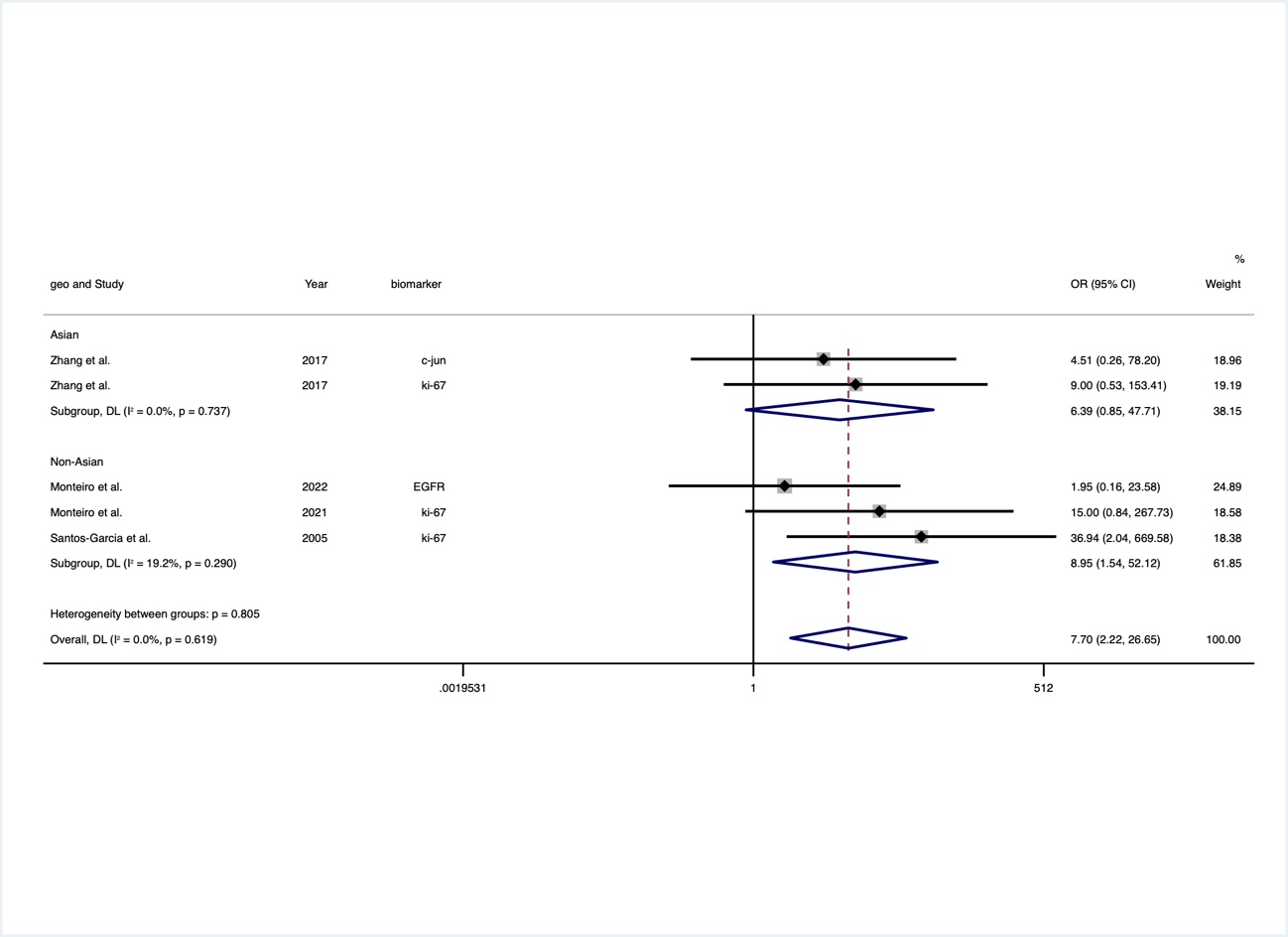


**5.2 Hallmark Evading growth suppressors stratified by geographical area**

**Figure S34.** Forest plot graphically representing the meta-analysis on the magnitude of association -using RR as effect size metric- between hallmark of cancer expression and OLs malignant transformation risk stratified by geographical area. RR, relative risk; CI, confidence intervals, DerSimonian and Laird, DL. Random-effects model, inverse-variance weighting based on the DL method. A RR > 1 suggests a higher malignant transformation risk. Diamonds indicate the pooled RR with their corresponding 95% CIs.


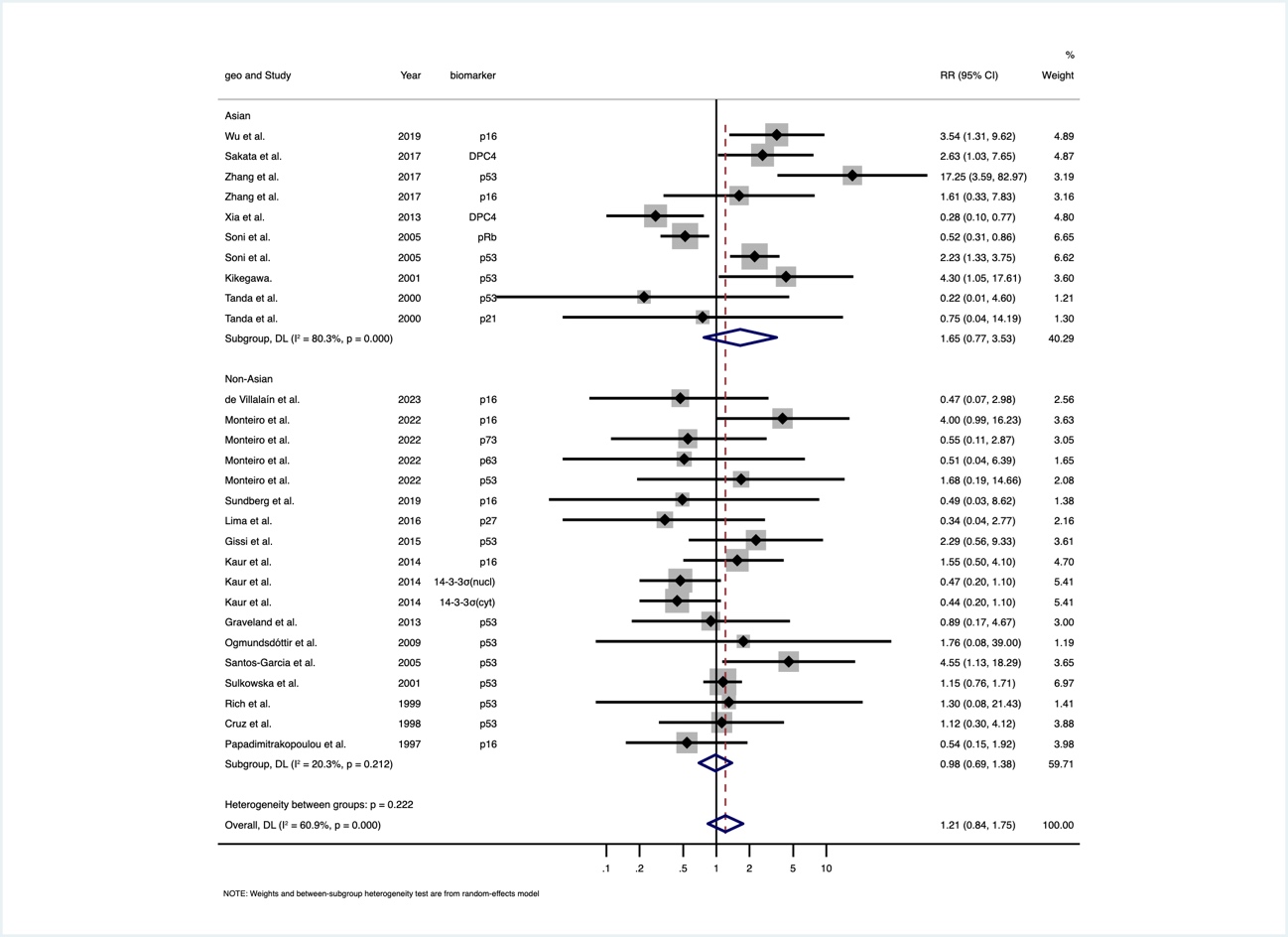


**Figure S35.** Forest plot graphically representing the differential expression of biomarkers on the hallmark evading growth suppressors -using pooled proportions as ES metric, expressed as percentage- among OL patients stratified by geographical area. ES, effect size; CI, confidence interval; Random-effects model.


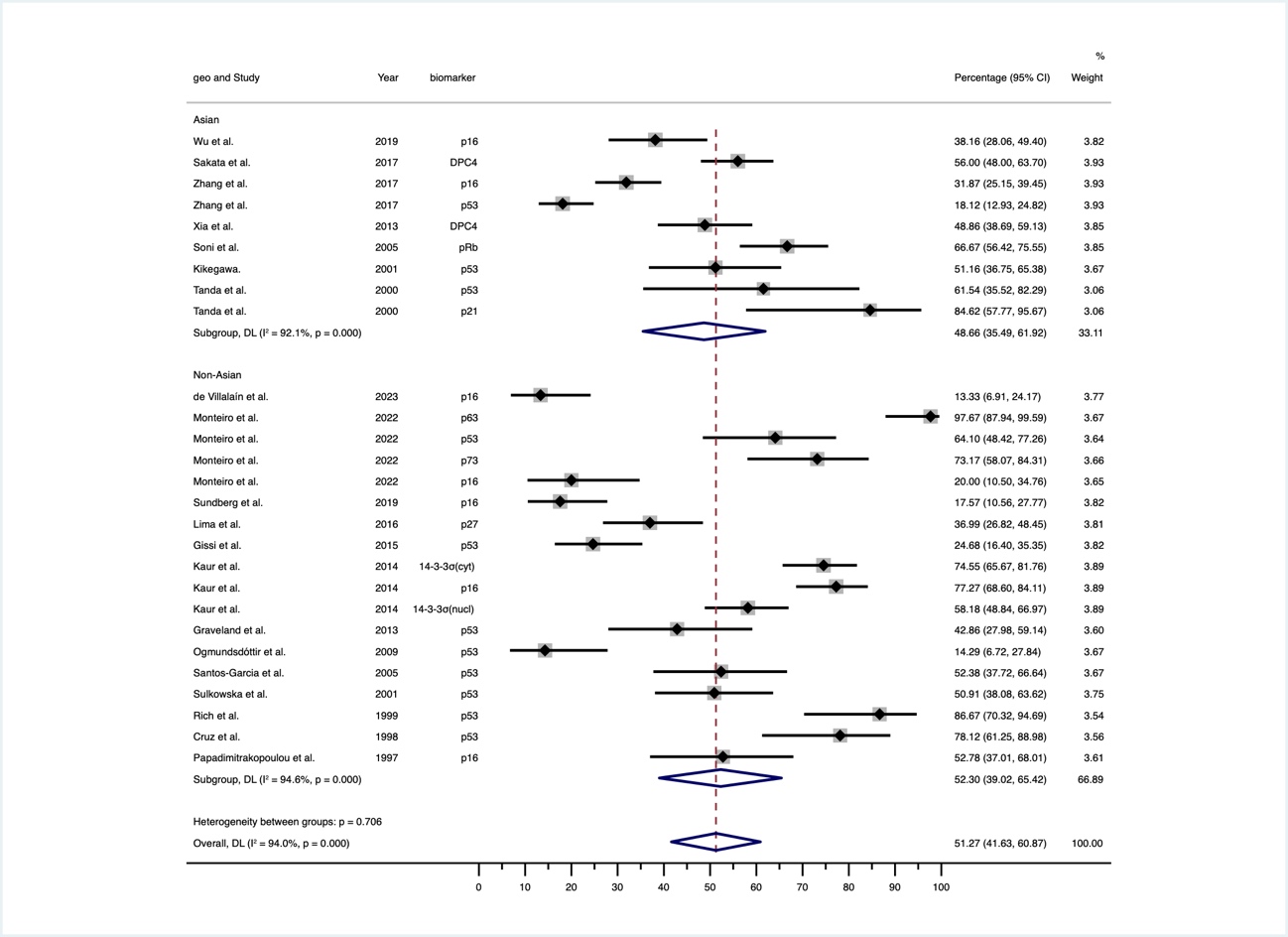


**Figure S36.** Forest plot graphically representing the meta-analysis of the magnitude of association -using OR as effect size metric- in order to compare the differential expression of biomarkers on the hallmark evading growth suppressors between OL and healthy controls stratified by geographical area. OR, odds ratio; CI, confidence interval; Random-effects model, inverse-variance weighting based on the DL method. A OR> 1 suggests a higher expression in OL in comparison to healthy oral mucosa. Diamonds indicate the pooled OR with their corresponding 95% CIs.


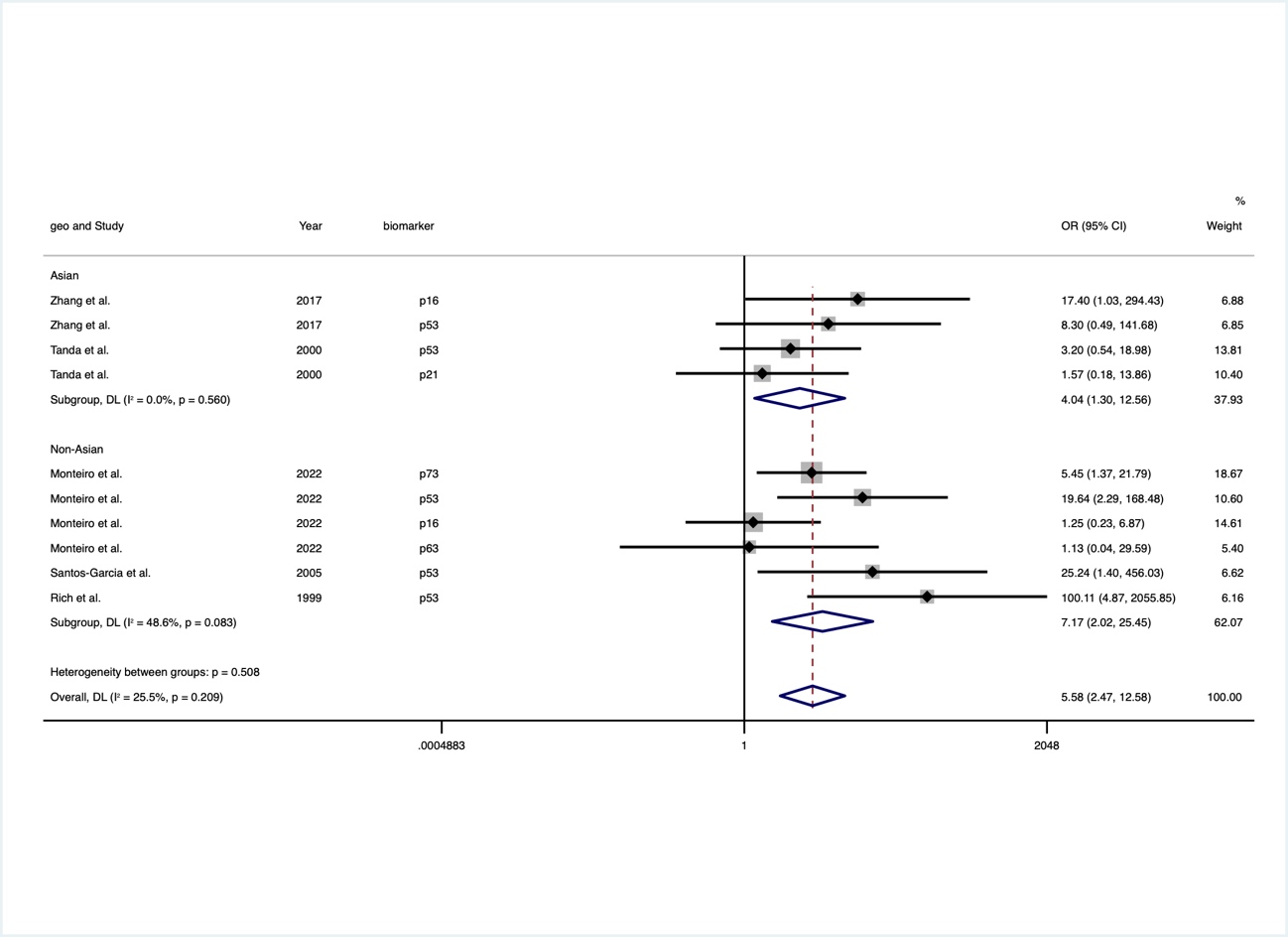


**5.3 Hallmark Resisting cell death**

**Figure S37.** Forest plot graphically representing the meta-analysis on the magnitude of association -using RR as effect size metric- between hallmark of cancer expression and OLs malignant transformation risk stratified by geographical area. RR, relative risk; CI, confidence intervals, DerSimonian and Laird, DL. Random-effects model, inverse-variance weighting based on the DL method. A RR > 1 suggests a higher malignant transformation risk. Diamonds indicate the pooled RR with their corresponding 95% CIs.


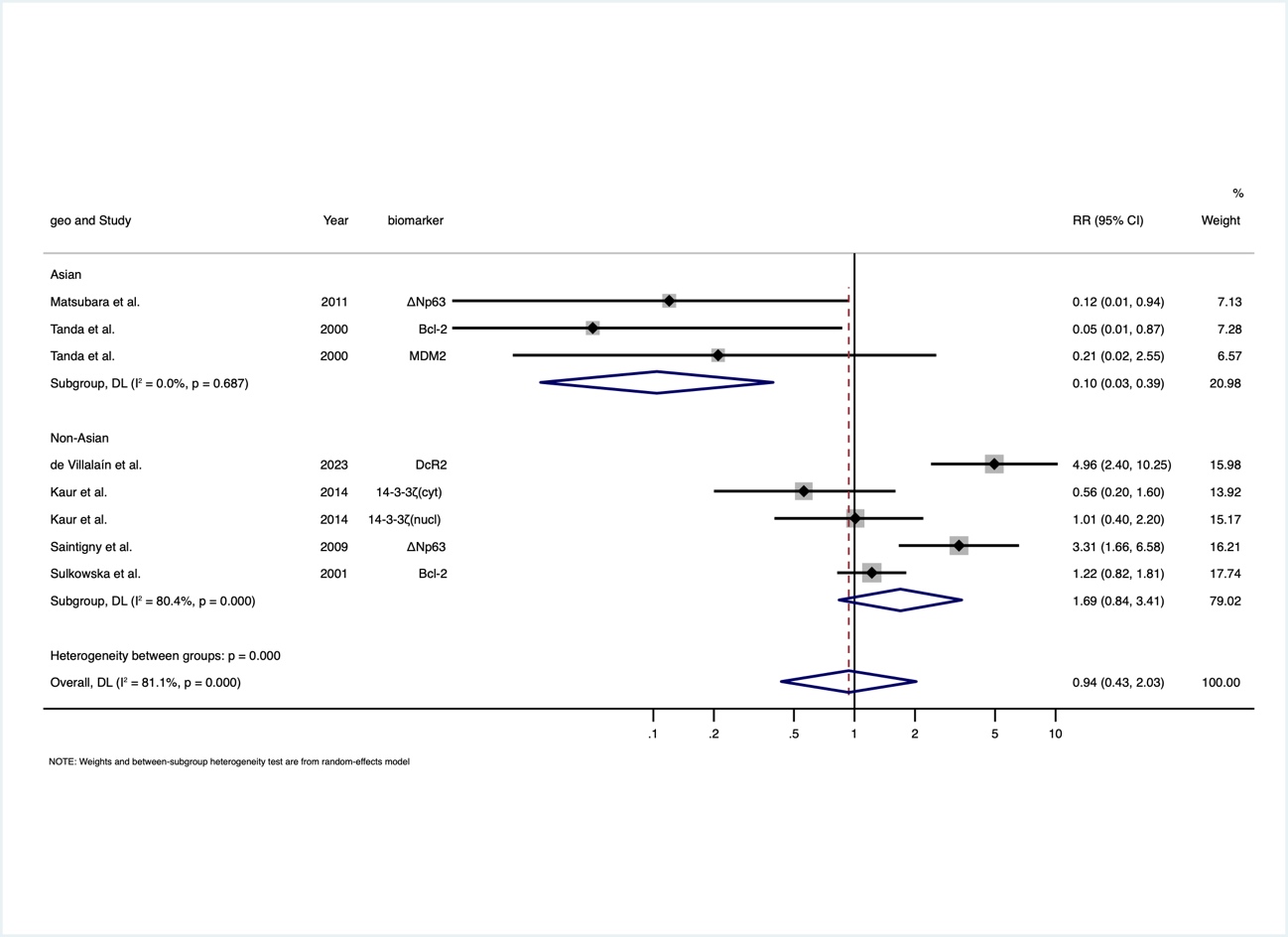


**Figure S38.** Forest plot graphically representing the differential expression of biomarkers on the hallmark resisting cell death -using pooled proportions as ES metric, expressed as percentage- among OL patients stratified by geographical area. ES, effect size; CI, confidence interval; Random-effects model.


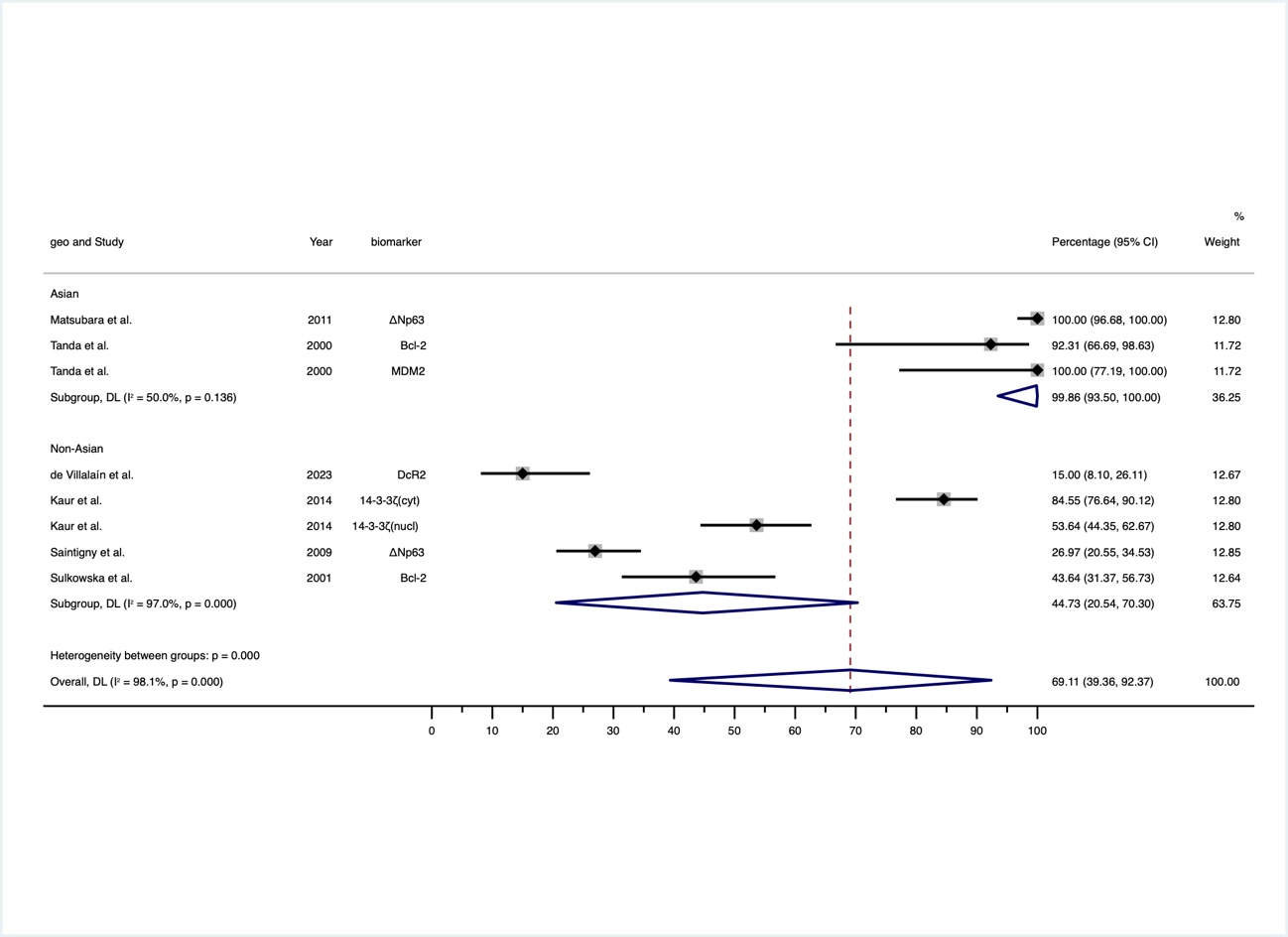


**Figure S39.** Forest plot graphically representing the meta-analysis of the magnitude of association -using OR as effect size metric- in order to compare the differential expression of biomarkers on the hallmark resisting cell death between OL and healthy controls stratified by geographical area. OR, odds ratio; CI, confidence interval; Random-effects model, inverse-variance weighting based on the DL method. A OR> 1 suggests a higher expression in OL in comparison to healthy oral mucosa. Diamonds indicate the pooled OR with their corresponding 95% CIs.


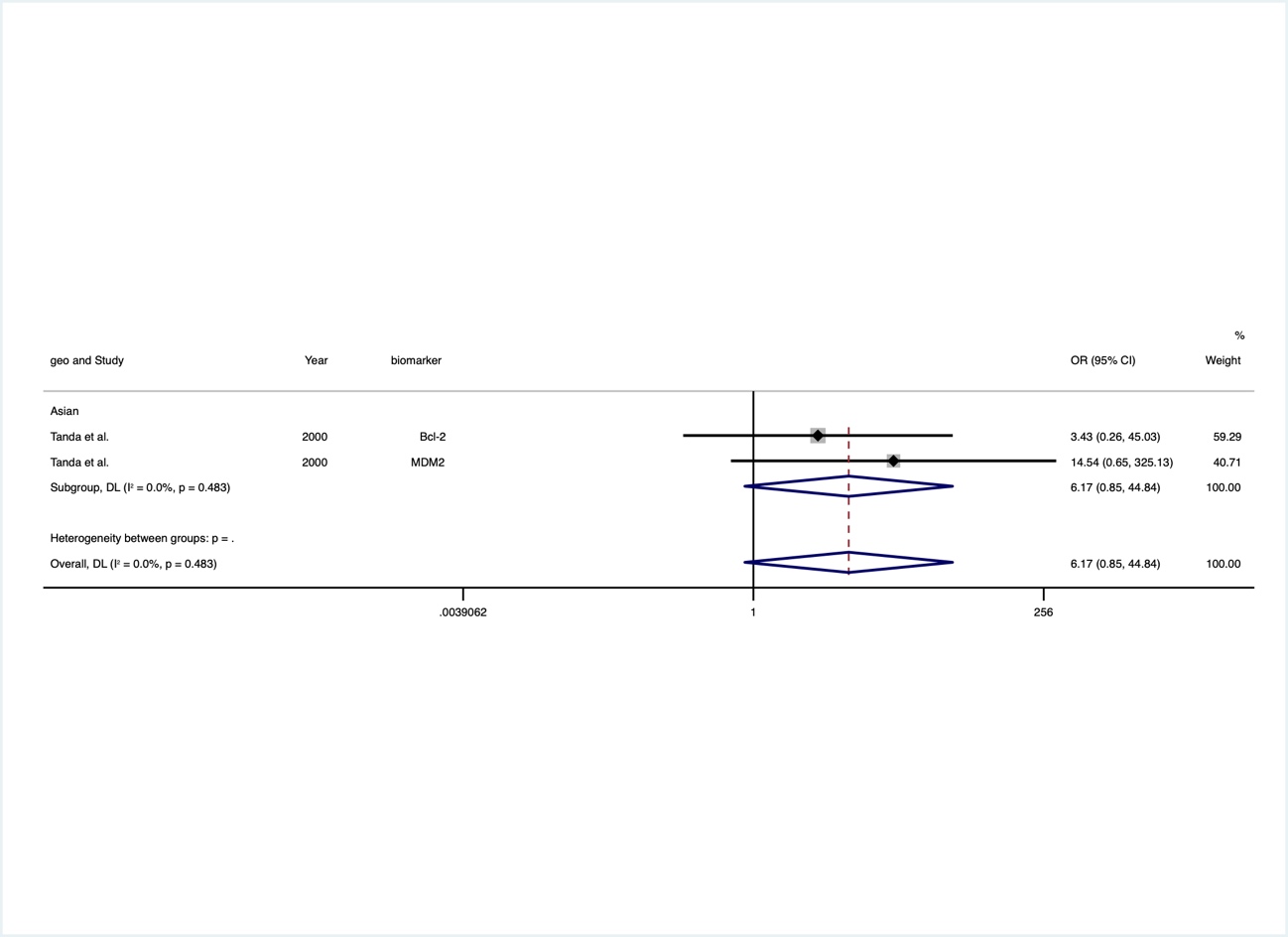


**5.4 Hallmark Enabling replicative immortality stratified by geographical area**

**Figure S40.** Forest plot graphically representing the meta-analysis on the magnitude of association -using RR as effect size metric- between hallmark of cancer expression and OLs malignant transformation risk stratified by geographical area. RR, relative risk; CI, confidence intervals, DerSimonian and Laird, DL. Random-effects model, inverse-variance weighting based on the DL method. A RR > 1 suggests a higher malignant transformation risk. Diamonds indicate the pooled RR with their corresponding 95% CIs.


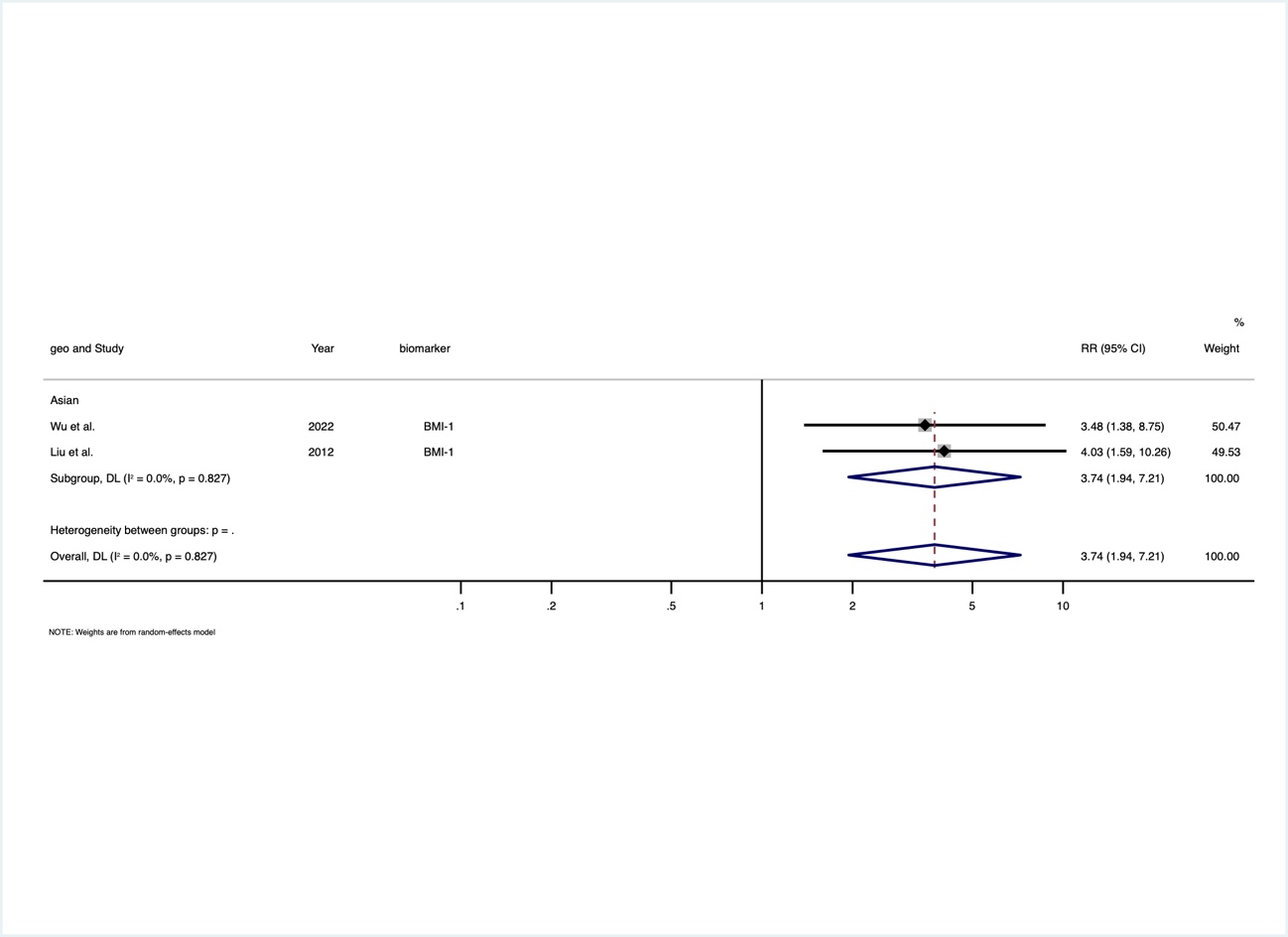


**Figure S41.** Forest plot graphically representing the differential expression of biomarkers on the hallmark enabling replicative immortality -using pooled proportions as ES metric, expressed as percentage- among OL patients stratified by geographical area. ES, effect size; CI, confidence interval; Random-effects model.


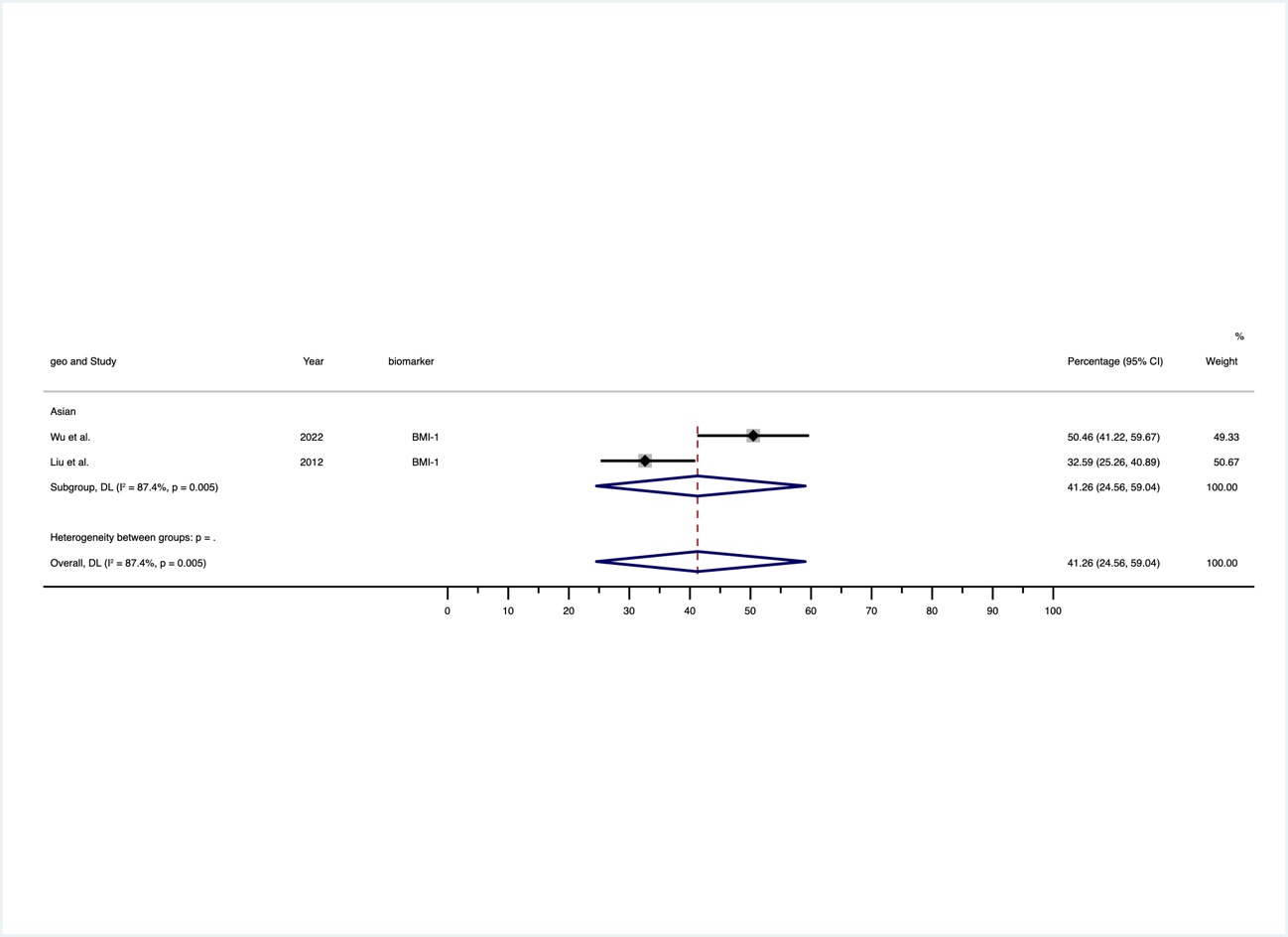


**5.5 Hallmark Inducing angiogenesis stratified by geographical area**

**Figure S42.** Forest plot graphically representing the meta-analysis on the magnitude of association -using RR as effect size metric- between hallmark of cancer expression and OLs malignant transformation risk stratified by geographical area. RR, relative risk; CI, confidence intervals, DerSimonian and Laird, DL. Random-effects model, inverse-variance weighting based on the DL method. A RR > 1 suggests a higher malignant transformation risk. Diamonds indicate the pooled RR with their corresponding 95% CIs.


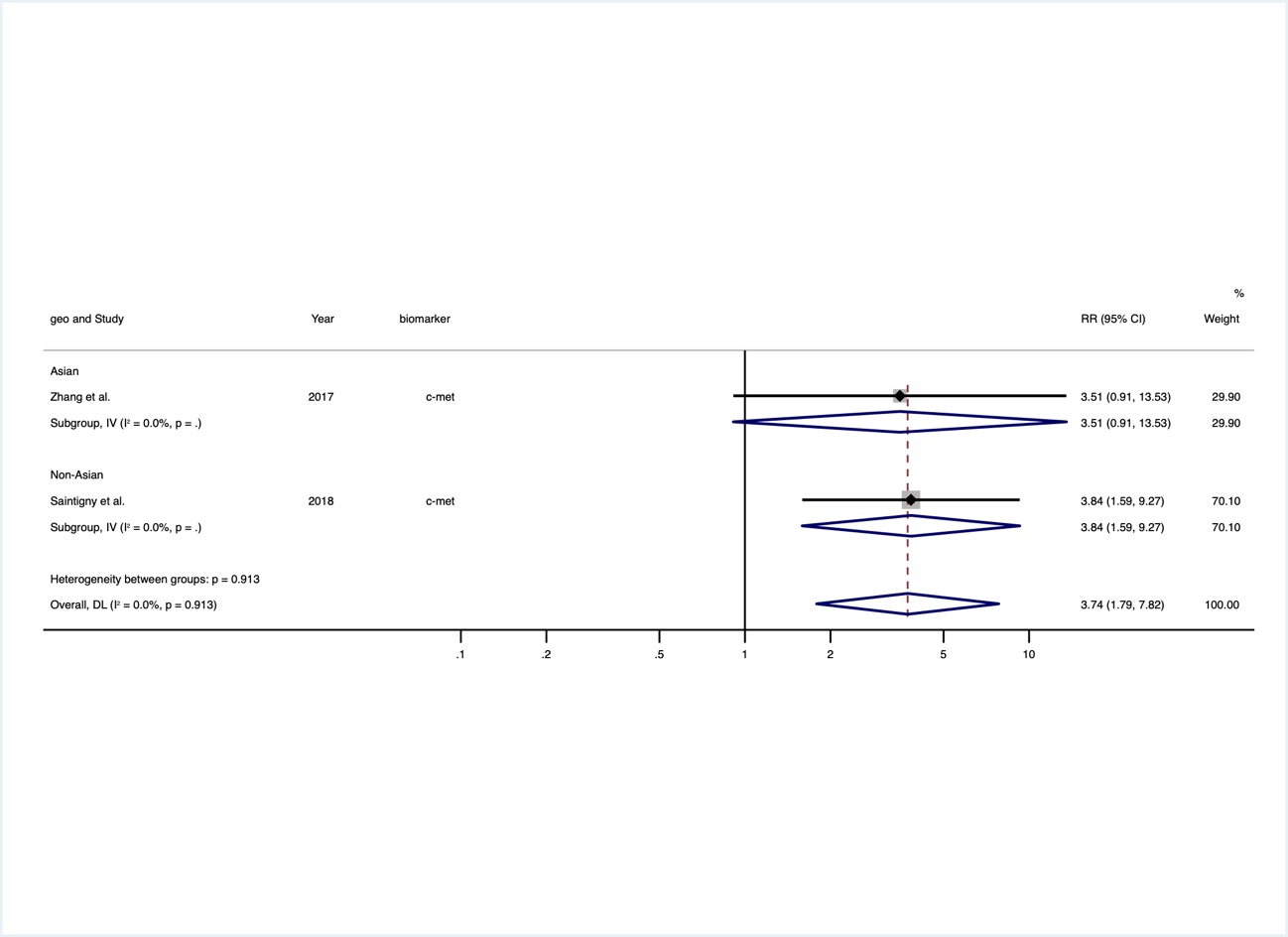


**Figure S43.** Forest plot graphically representing the differential expression of biomarkers on the hallmark inducing angiogenesis -using pooled proportions as ES metric, expressed as percentage- among OL patients stratified by geographical area. ES, effect size; CI, confidence interval; Random-effects model.


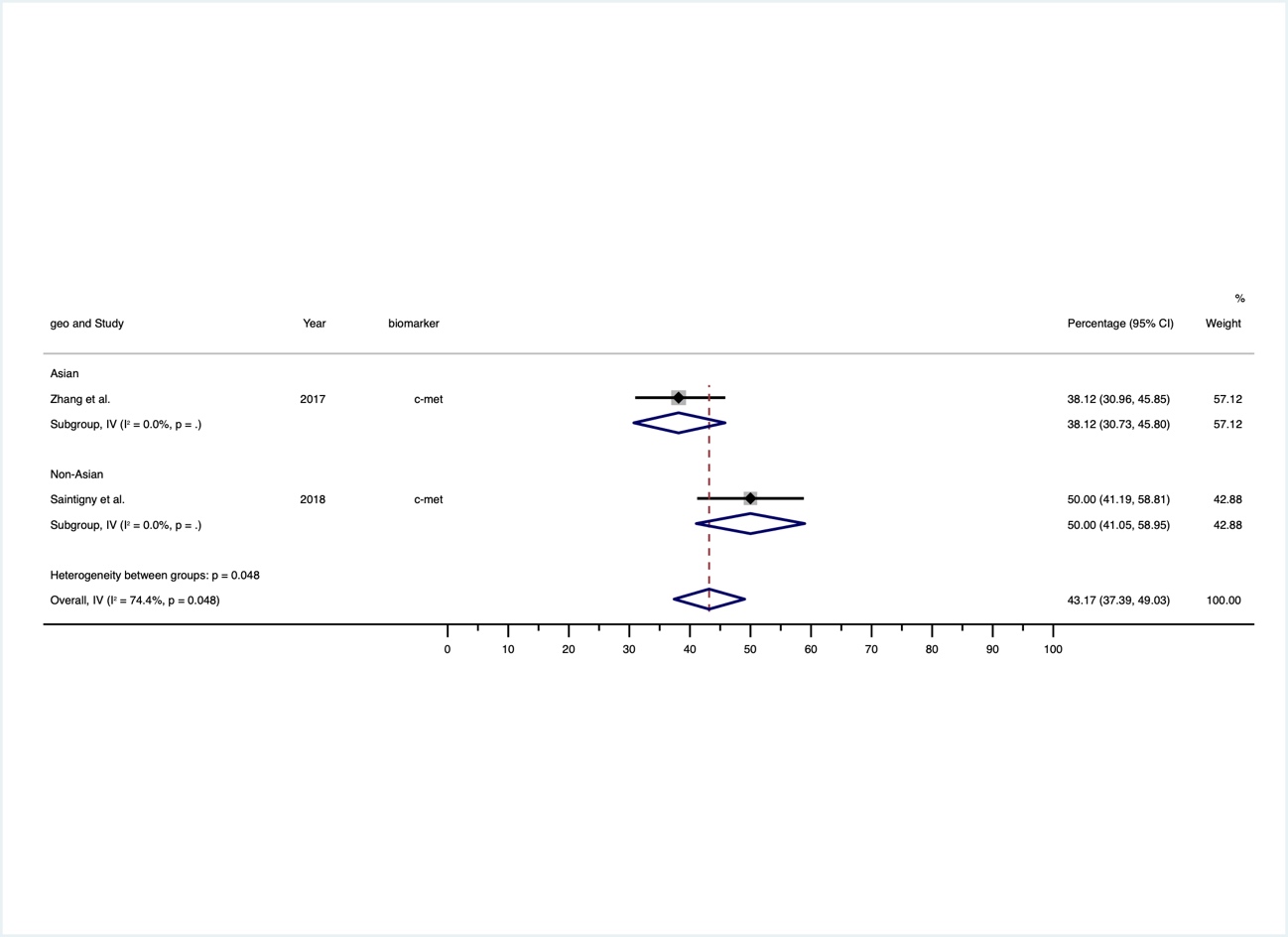


**5.6 Hallmark Activating invasion and metastasis stratified by geographical area**

**Figure S44.** Forest plot graphically representing the meta-analysis on the magnitude of association -using RR as effect size metric- between hallmark of cancer expression and OLs malignant transformation risk stratified by geographical area. RR, relative risk; CI, confidence intervals, DerSimonian and Laird, DL. Random-effects model, inverse-variance weighting based on the DL method. A RR > 1 suggests a higher malignant transformation risk. Diamonds indicate the pooled RR with their corresponding 95% CIs.


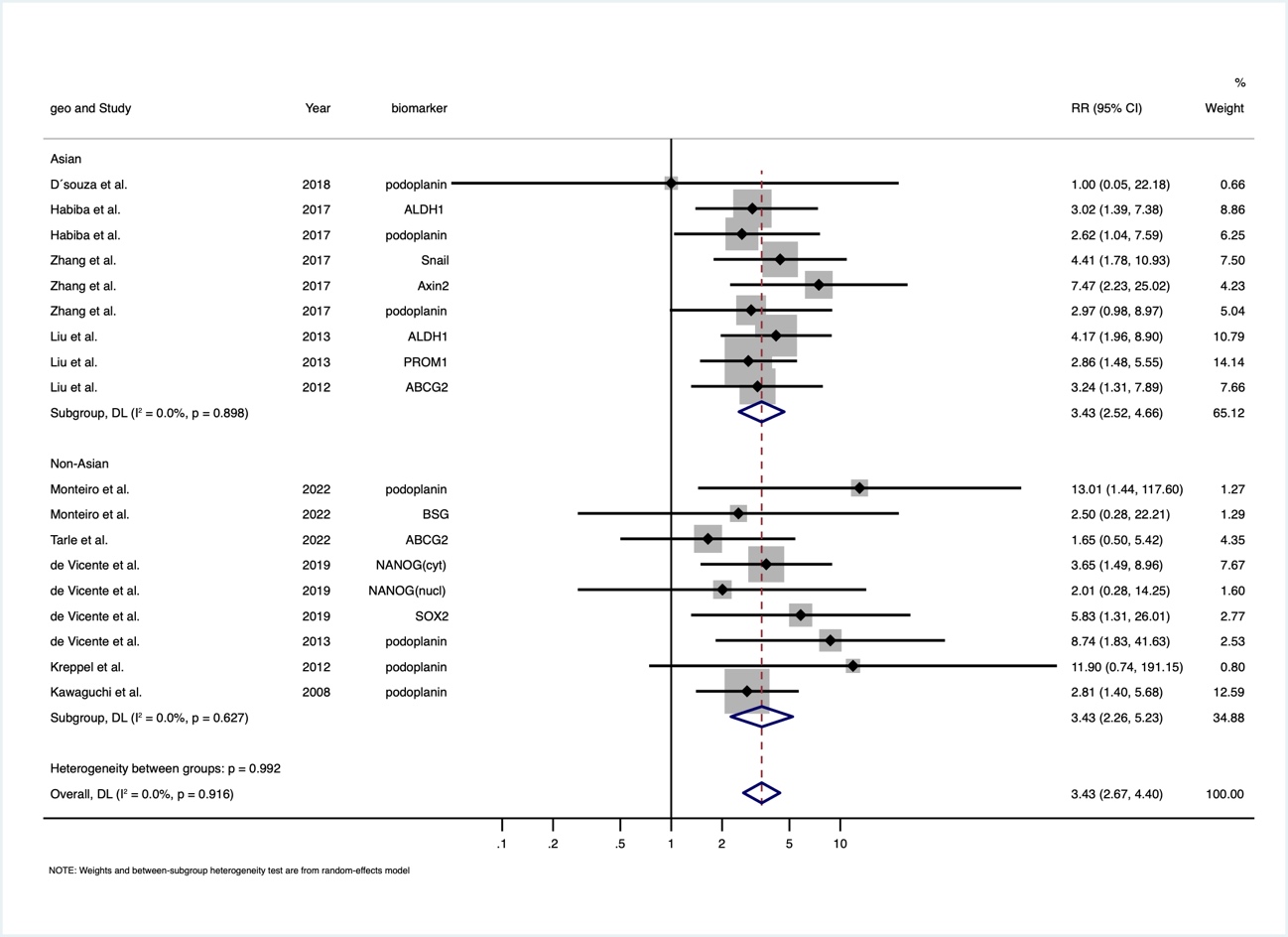


**Figure S45.** Forest plot graphically representing the differential expression of biomarkers on the hallmark activating invasion and metastasis -using pooled proportions as ES metric, expressed as percentage- among OL patients stratified by geographical area. ES, effect size; CI, confidence interval; Random-effects model.


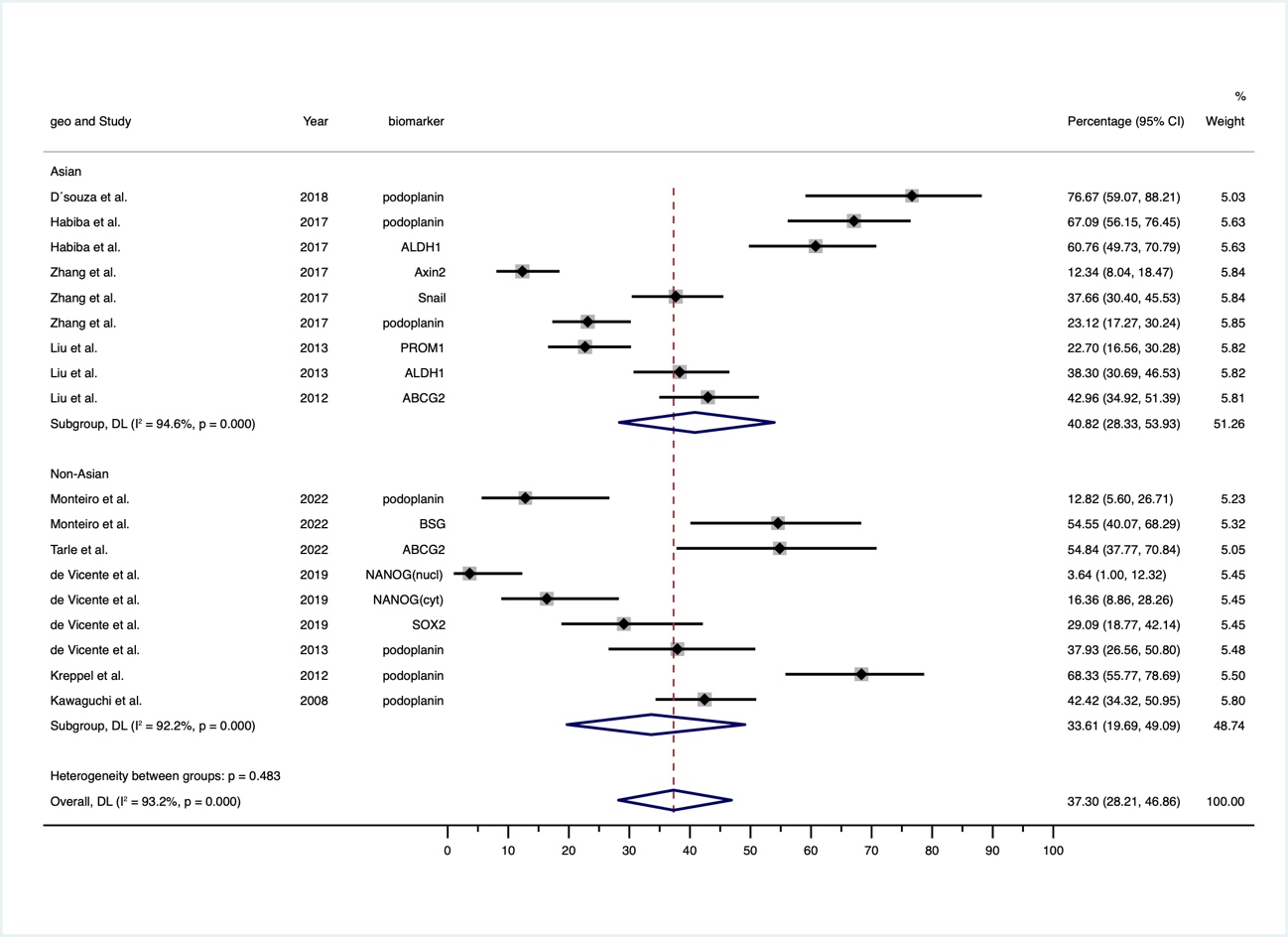


**Figure S46.** Forest plot graphically representing the meta-analysis of the magnitude of association -using OR as effect size metric- in order to compare the differential expression of biomarkers on the hallmark activating invasion and metastasis between OL and healthy controls stratified by geographical area. OR, odds ratio; CI, confidence interval; Random-effects model, inverse-variance weighting based on the DL method. A OR> 1 suggests a higher expression in OL in comparison to healthy oral mucosa. Diamonds indicate the pooled OR with their corresponding 95% CIs.


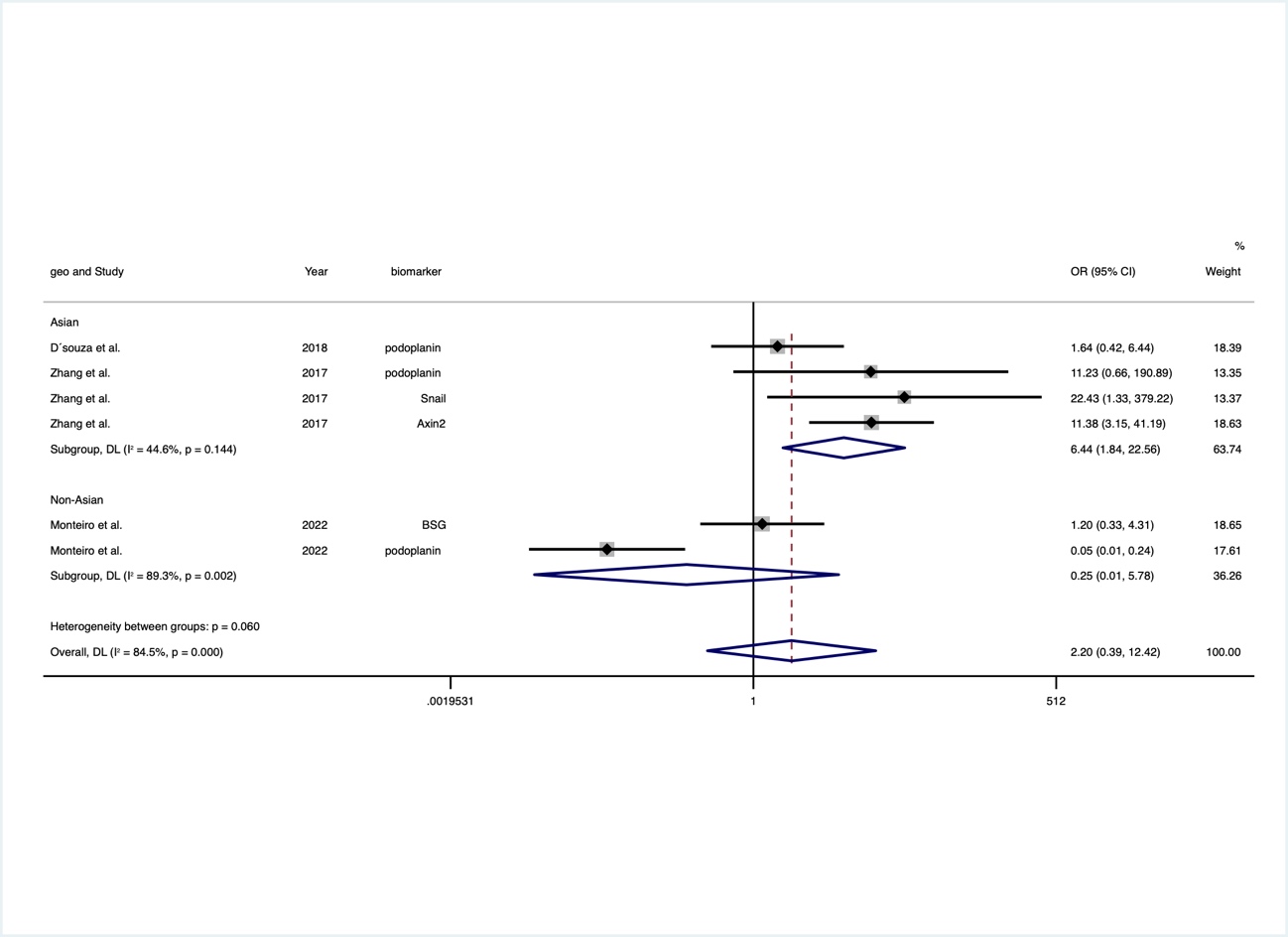


**5.7 Hallmark Avoiding immune destruction stratified by geographical area**

**Figure S47.** Forest plot graphically representing the meta-analysis on the magnitude of association -using RR as effect size metric- between hallmark of cancer expression and OLs malignant transformation risk stratified by geographical area. RR, relative risk; CI, confidence intervals, DerSimonian and Laird, DL. Random-effects model, inverse-variance weighting based on the DL method. A RR > 1 suggests a higher malignant transformation risk. Diamonds indicate the pooled RR with their corresponding 95% CIs.


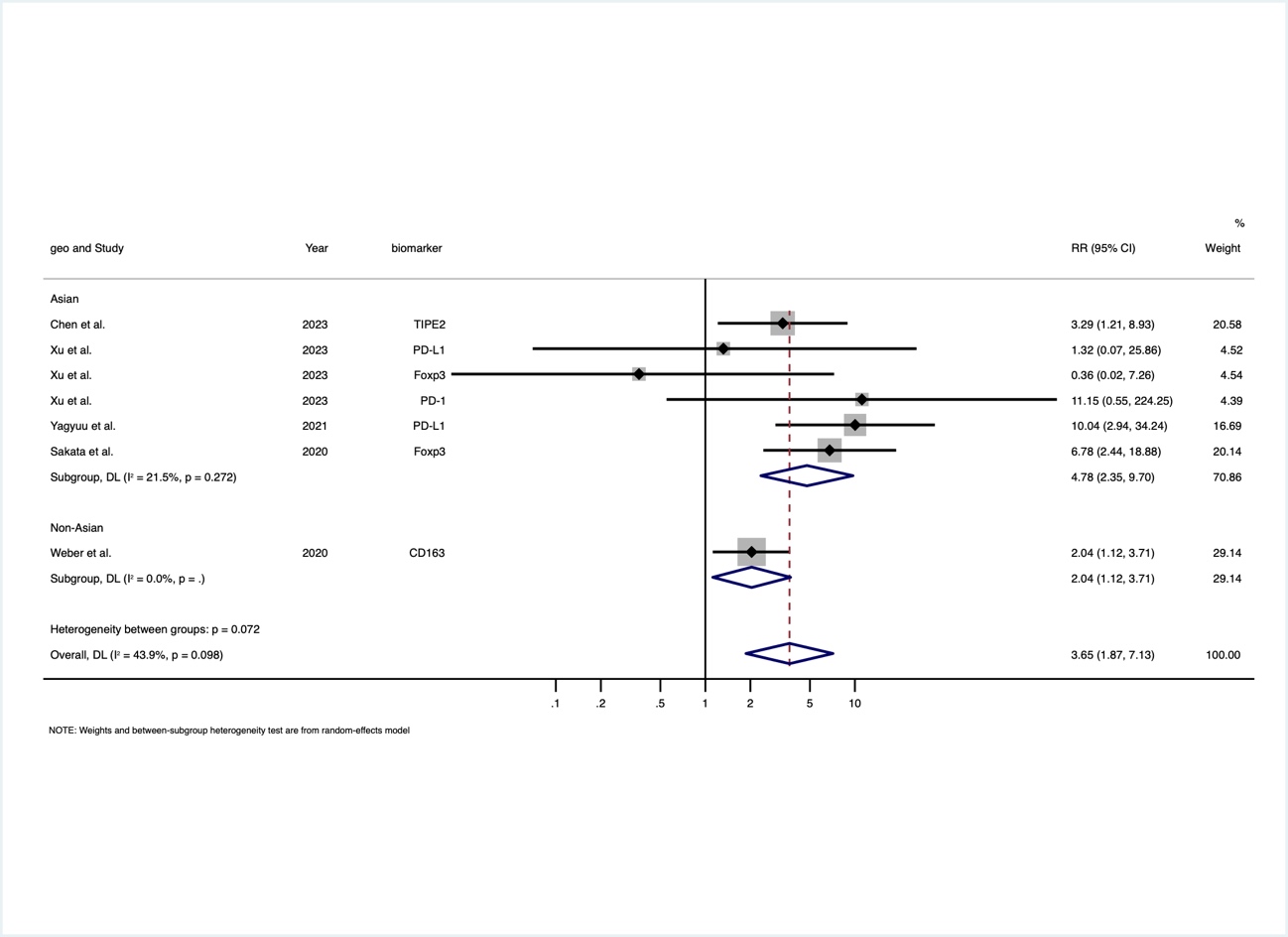


**Figure S48.** Forest plot graphically representing the differential expression of biomarkers on the hallmark avoiding immune destruction -using pooled proportions as ES metric, expressed as percentage- among OL patients stratified by geographical area. ES, effect size; CI, confidence interval; Random-effects model.


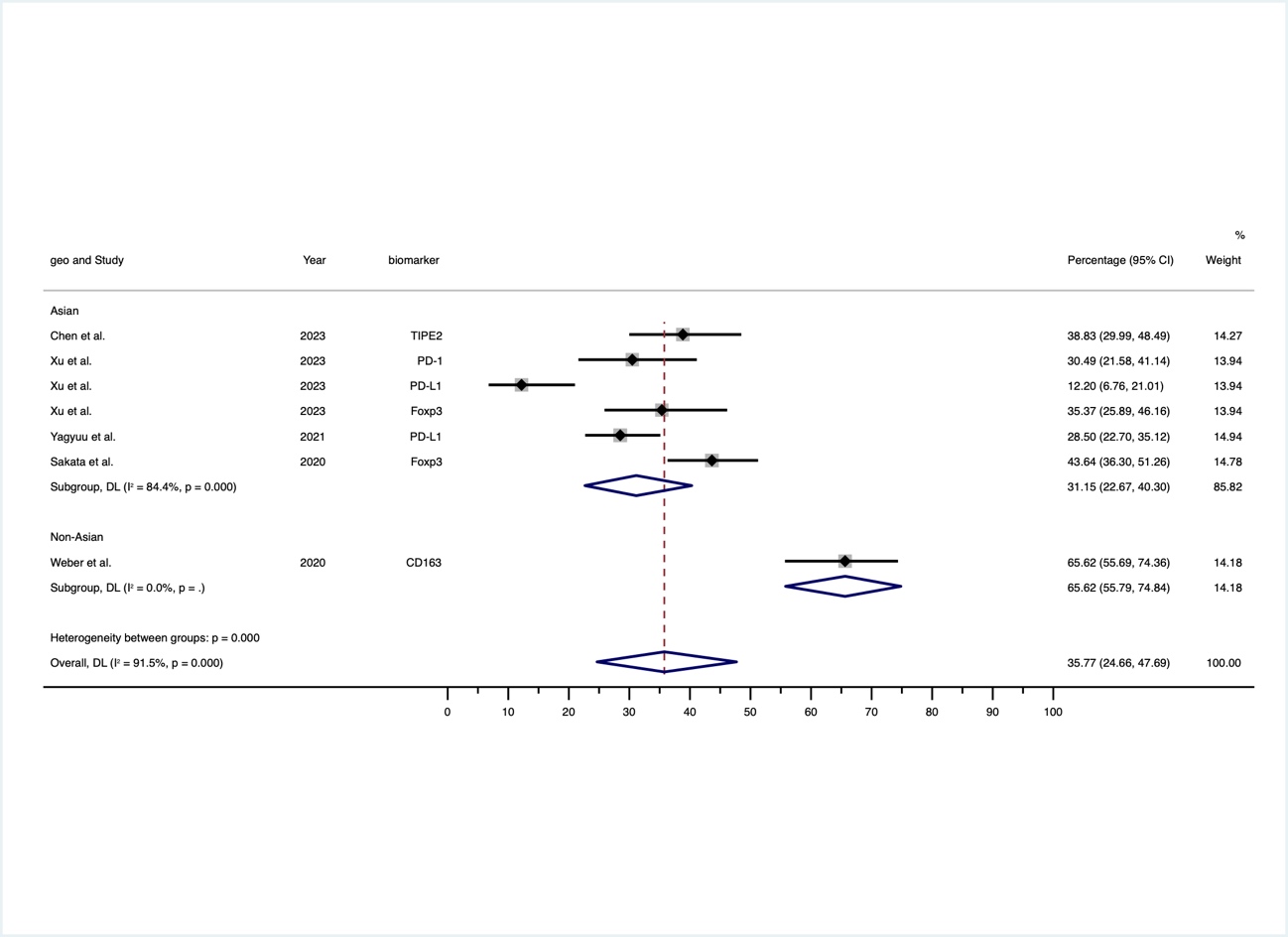


**5.8 Hallmark Deregulating cellular energetics stratified by geographical area**

**Figure S49.** Forest plot graphically representing the meta-analysis on the magnitude of association -using RR as effect size metric- between hallmark of cancer expression and OLs malignant transformation risk stratified by geographical area. RR, relative risk; CI, confidence intervals, DerSimonian and Laird, DL. Random-effects model, inverse-variance weighting based on the DL method. A RR > 1 suggests a higher malignant transformation risk. Diamonds indicate the pooled RR with their corresponding 95% CIs.


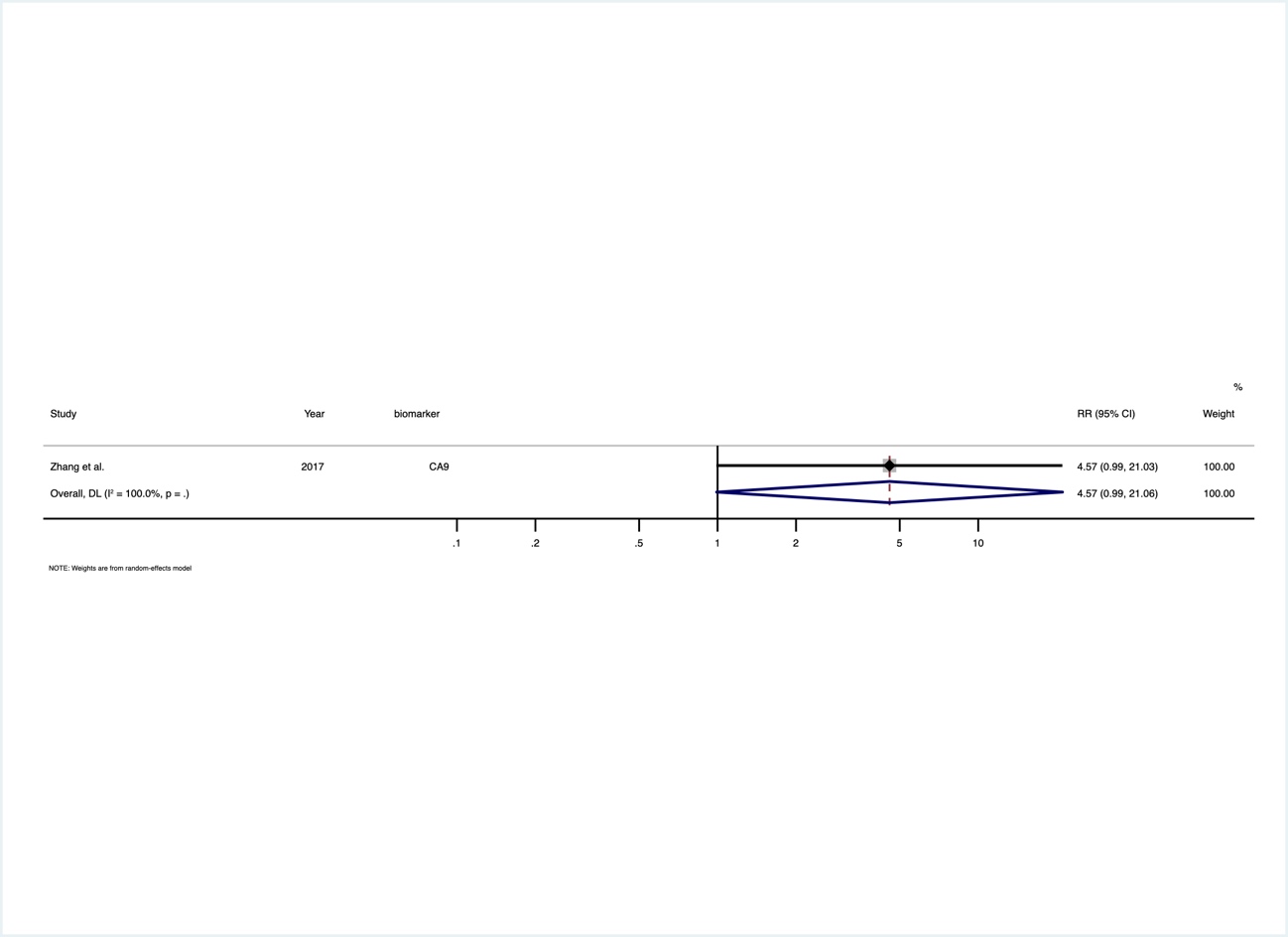


**Figure S50.** Forest plot graphically representing the differential expression of biomarkers on the hallmark deregulating cellular energetics -using pooled proportions as ES metric, expressed as percentage- among OL patients stratified by geographical area. ES, effect size; CI, confidence interval; Random-effects model.


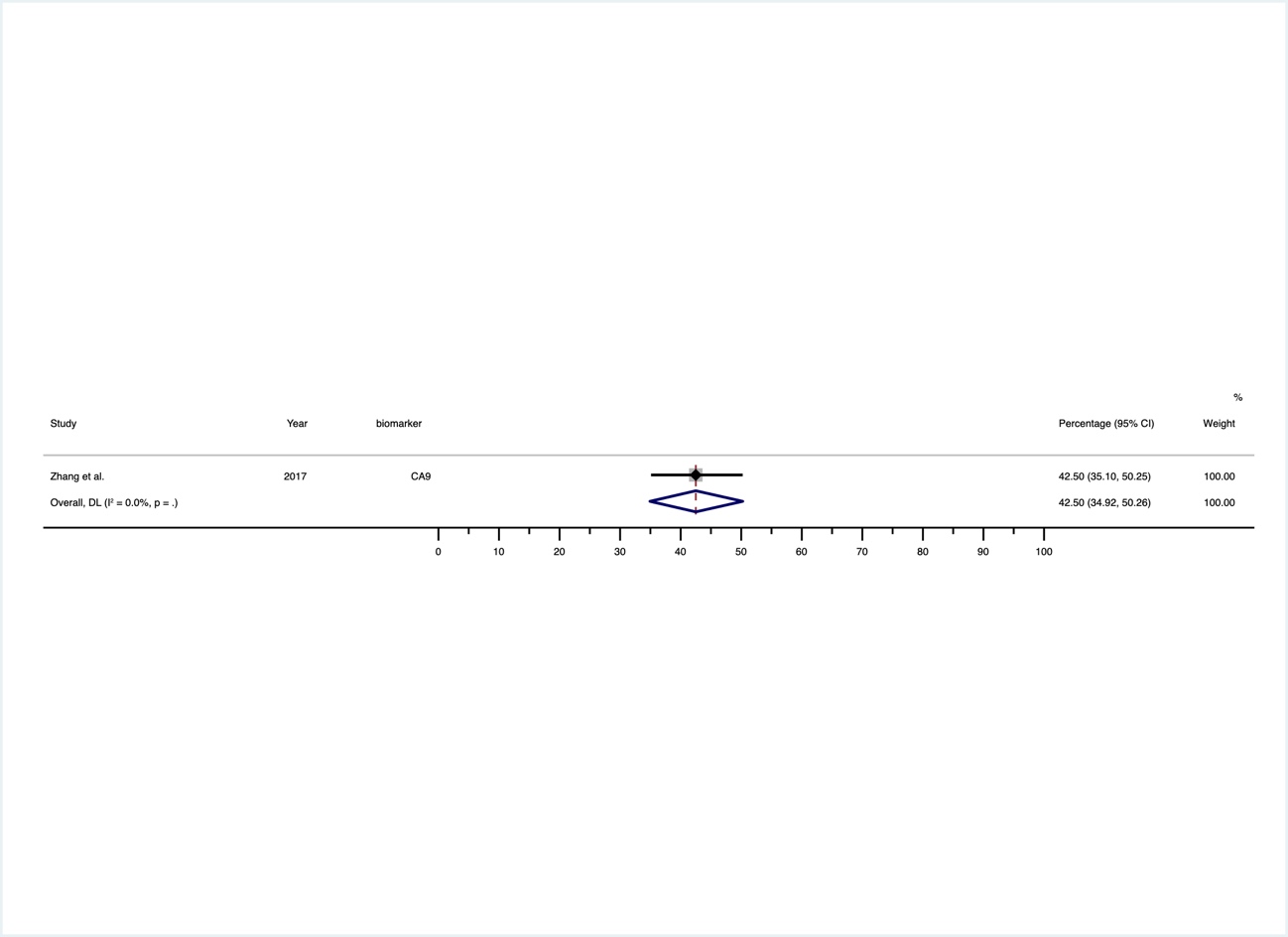


**Figure S51.** Forest plot graphically representing the meta-analysis of the magnitude of association -using OR as effect size metric- in order to compare the differential expression of biomarkers on the hallmark deregulating cellular energetics between OL and healthy controls stratified by geographical area. OR, odds ratio; CI, confidence interval; Random-effects model, inverse-variance weighting based on the DL method. A OR> 1 suggests a higher expression in OL in comparison to healthy oral mucosa. Diamonds indicate the pooled OR with their corresponding 95% CIs.


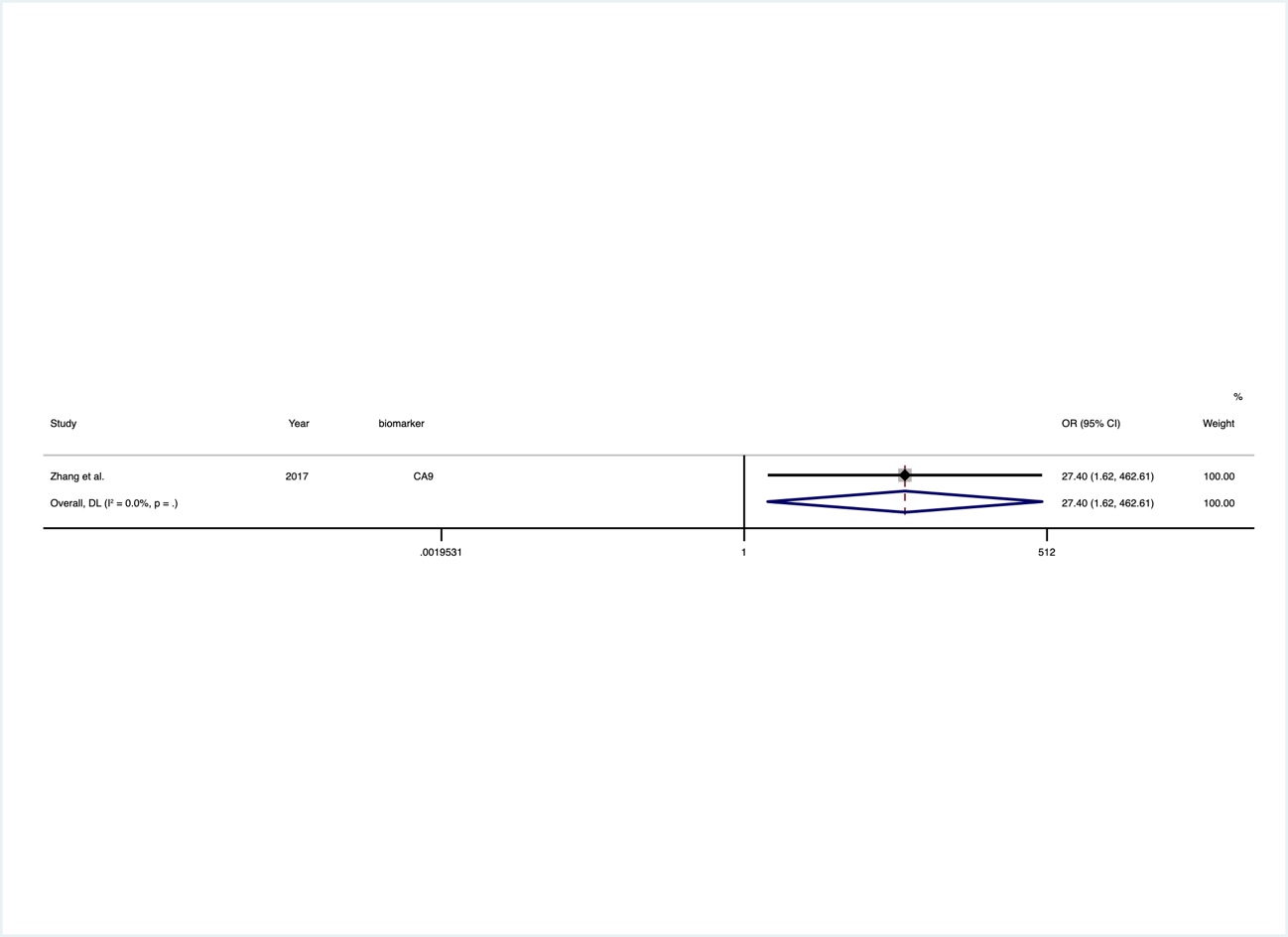


**5.9 Hallmark Genome instability and mutation stratified by geographical area**

**Figure S52.** Forest plot graphically representing the meta-analysis on the magnitude of association -using RR as effect size metric- between hallmark of cancer expression and OLs malignant transformation risk stratified by geographical area. RR, relative risk; CI, confidence intervals, DerSimonian and Laird, DL. Random-effects model, inverse-variance weighting based on the DL method. A RR > 1 suggests a higher malignant transformation risk. Diamonds indicate the pooled RR with their corresponding 95% CIs.


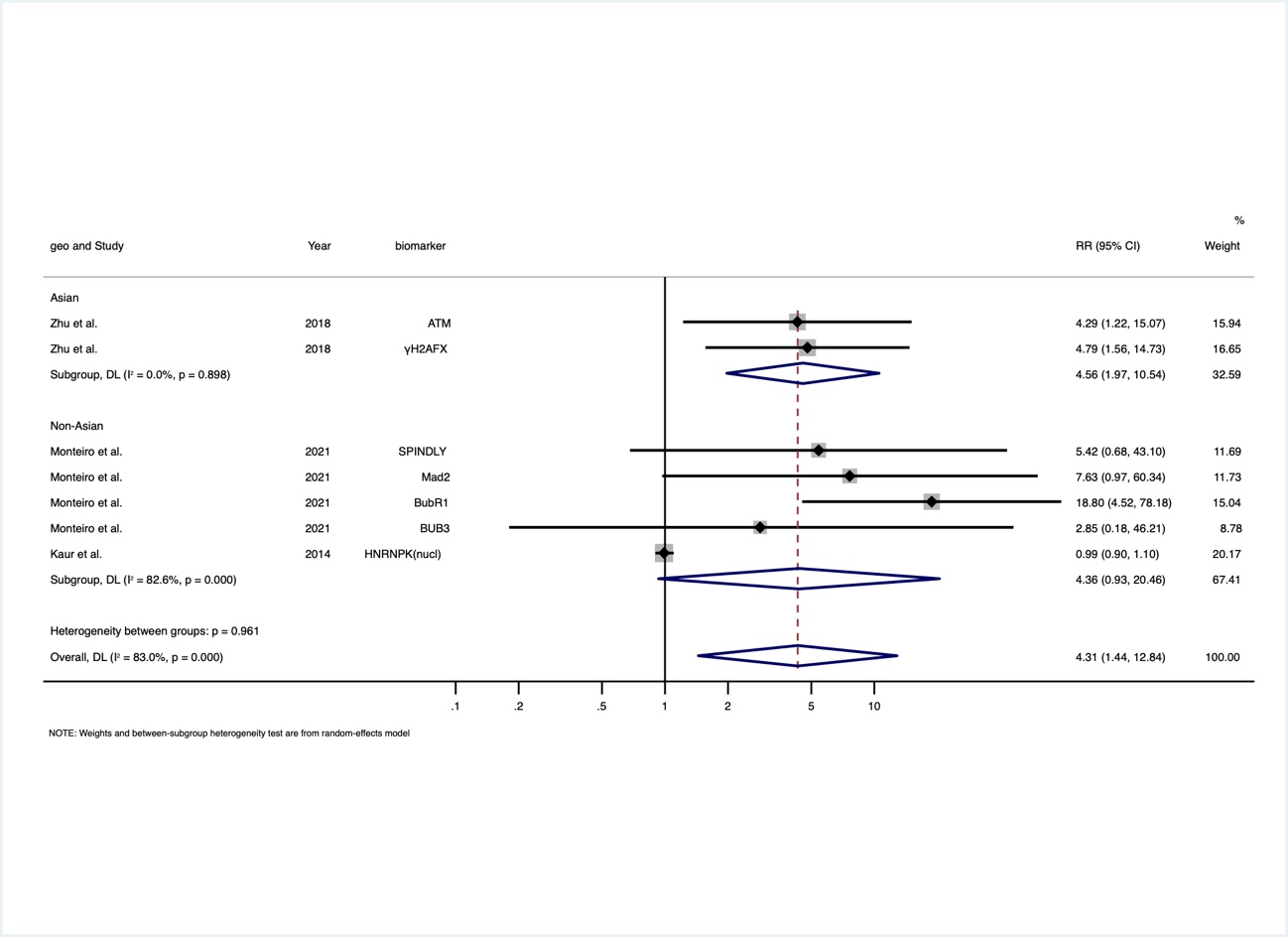


**Figure S53.** Forest plot graphically representing the differential expression of biomarkers on the hallmark genome instability and mutation -using pooled proportions as ES metric, expressed as percentage- among OL patients stratified by geographical area. ES, effect size; CI, confidence interval; Random-effects model.


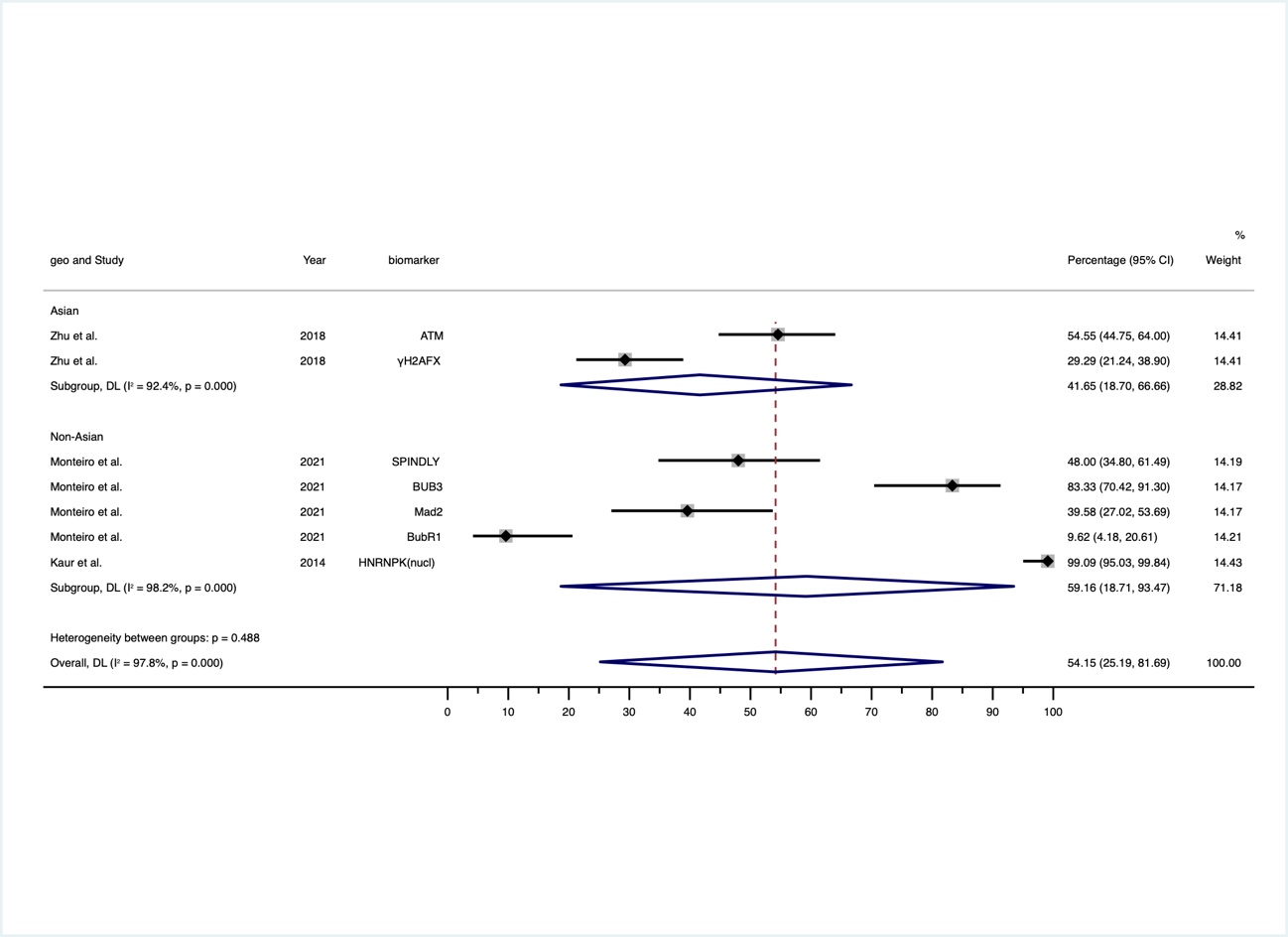


**Figure S54.** Forest plot graphically representing the meta-analysis of the magnitude of association -using OR as effect size metric- in order to compare the differential expression of biomarkers on the hallmark genome instability and mutation between OL and healthy controls stratified by geographical area. OR, odds ratio; CI, confidence interval; Random-effects model, inverse-variance weighting based on the DL method. A OR> 1 suggests a higher expression in OL in comparison to healthy oral mucosa. Diamonds indicate the pooled OR with their corresponding 95% CIs.


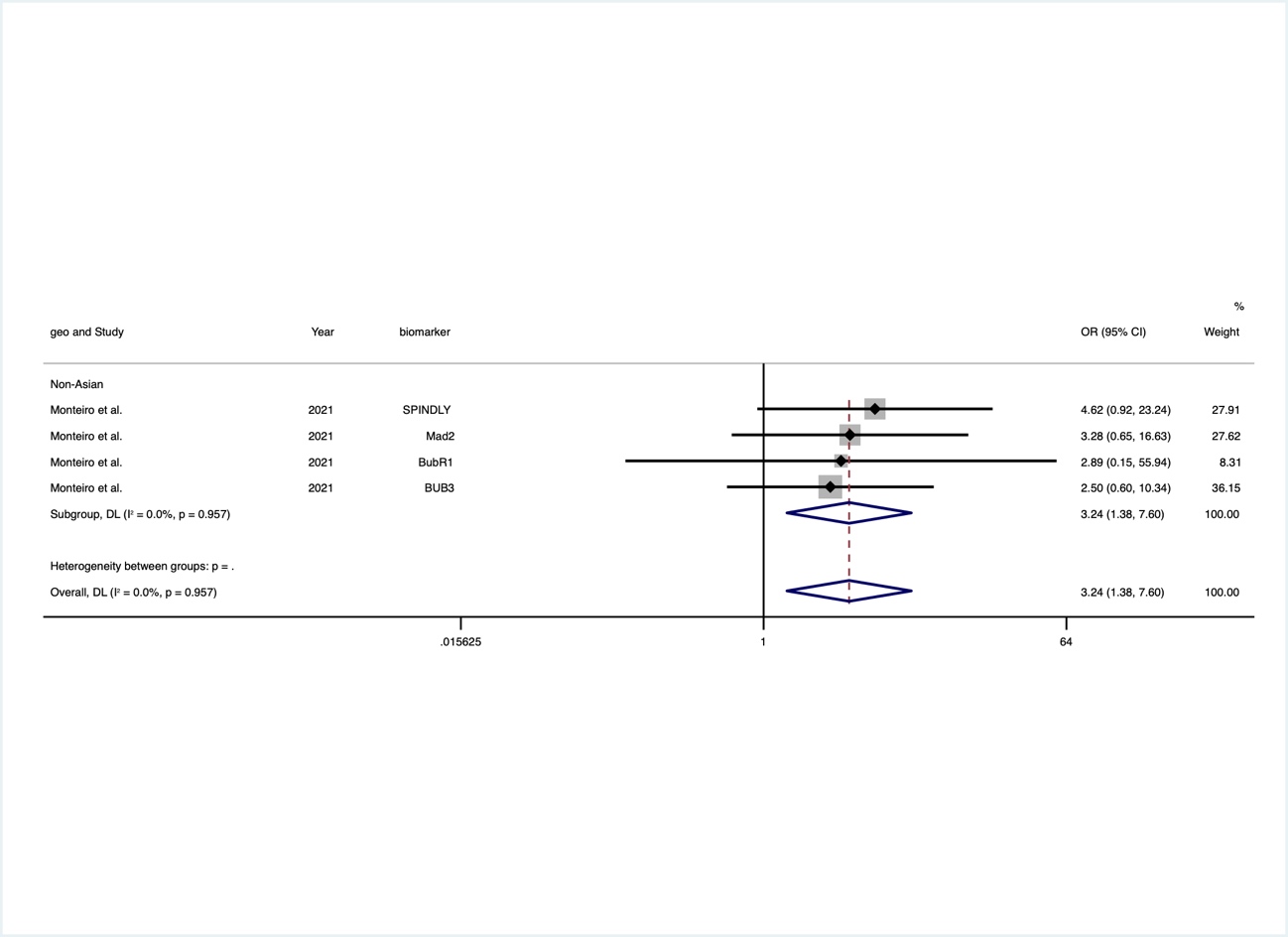


**5.10 Hallmark Tumor promoting inflammation stratified by geographical area**

**Figure S55.** Forest plot graphically representing the meta-analysis on the magnitude of association -using RR as effect size metric- between hallmark of cancer expression and OLs malignant transformation risk stratified by geographical area. RR, relative risk; CI, confidence intervals, DerSimonian and Laird, DL. Random-effects model, inverse-variance weighting based on the DL method. A RR > 1 suggests a higher malignant transformation risk. Diamonds indicate the pooled RR with their corresponding 95% CIs.


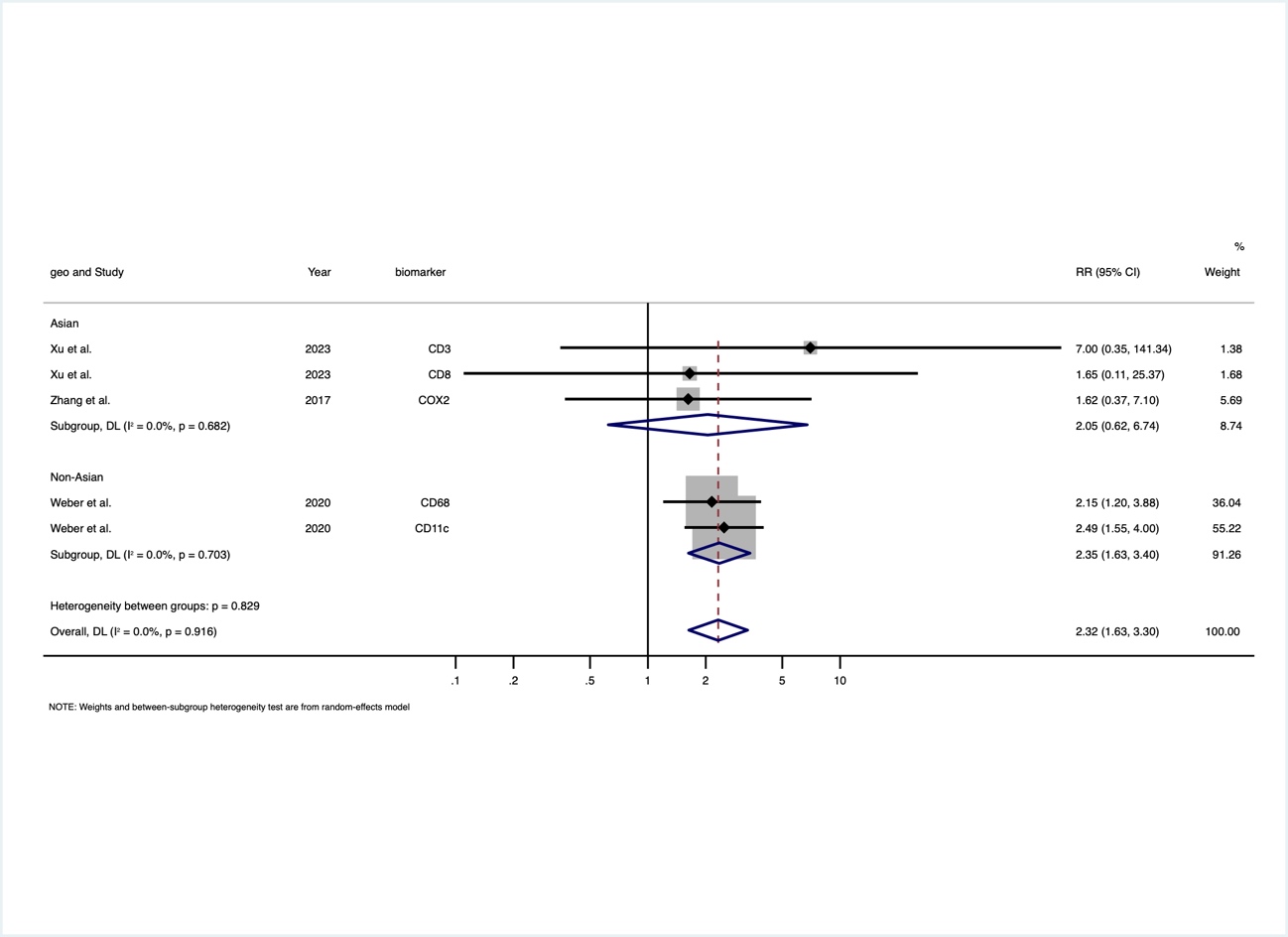


**Figure S56.** Forest plot graphically representing the differential expression of biomarkers on the hallmark tumor promoting inflammation -using pooled proportions as ES metric, expressed as percentage- among OL patients stratified by geographical area. ES, effect size; CI, confidence interval; Random-effects model.


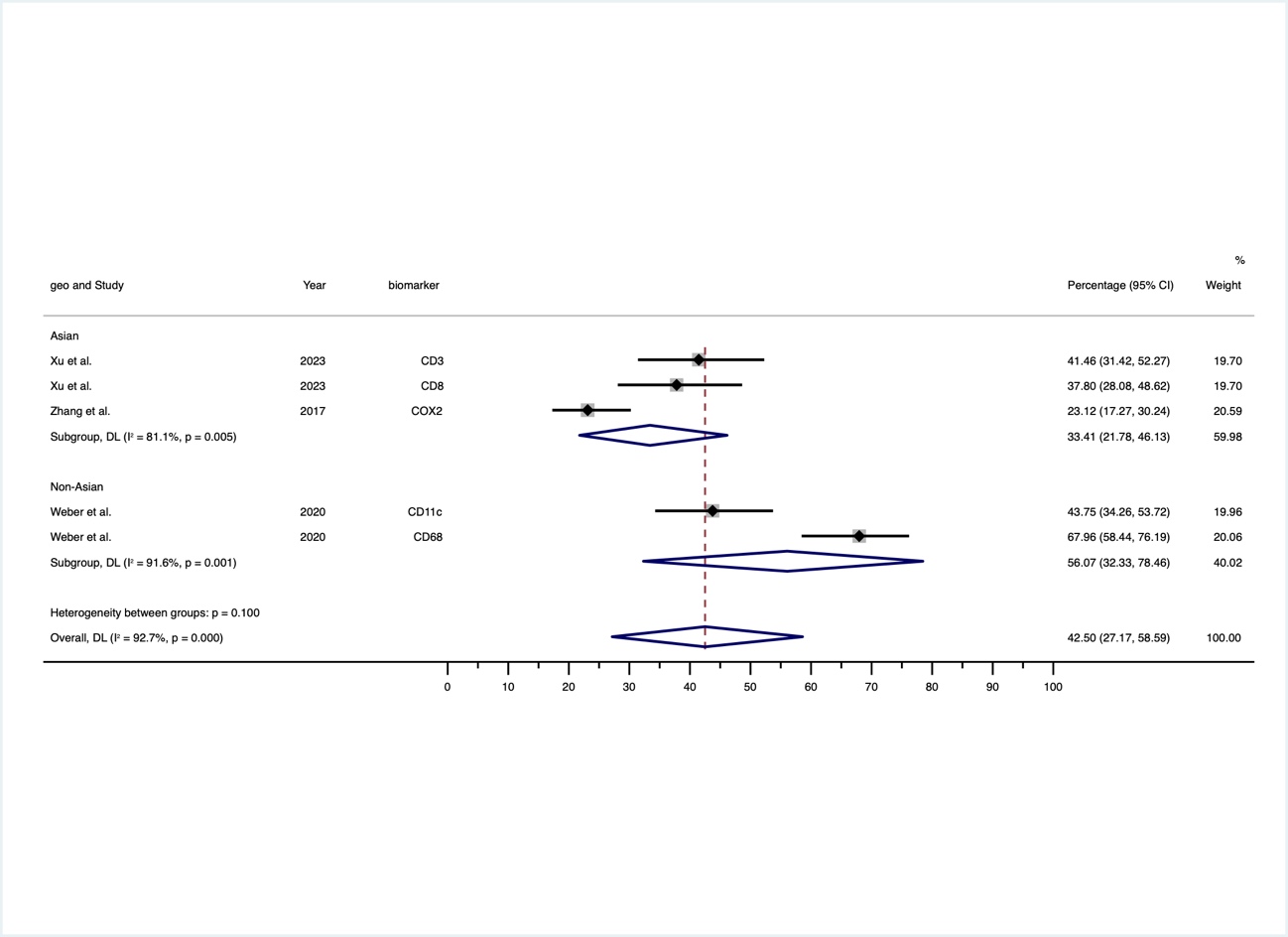


**Figure S57.** Forest plot graphically representing the meta-analysis of the magnitude of association -using OR as effect size metric- in order to compare the differential expression of biomarkers on the hallmark tumor promoting inflammation between OL and healthy controls stratified by geographical area. OR, odds ratio; CI, confidence interval; Random-effects model, inverse-variance weighting based on the DL method. A OR> 1 suggests a higher expression in OL in comparison to healthy oral mucosa. Diamonds indicate the pooled OR with their corresponding 95% CIs.


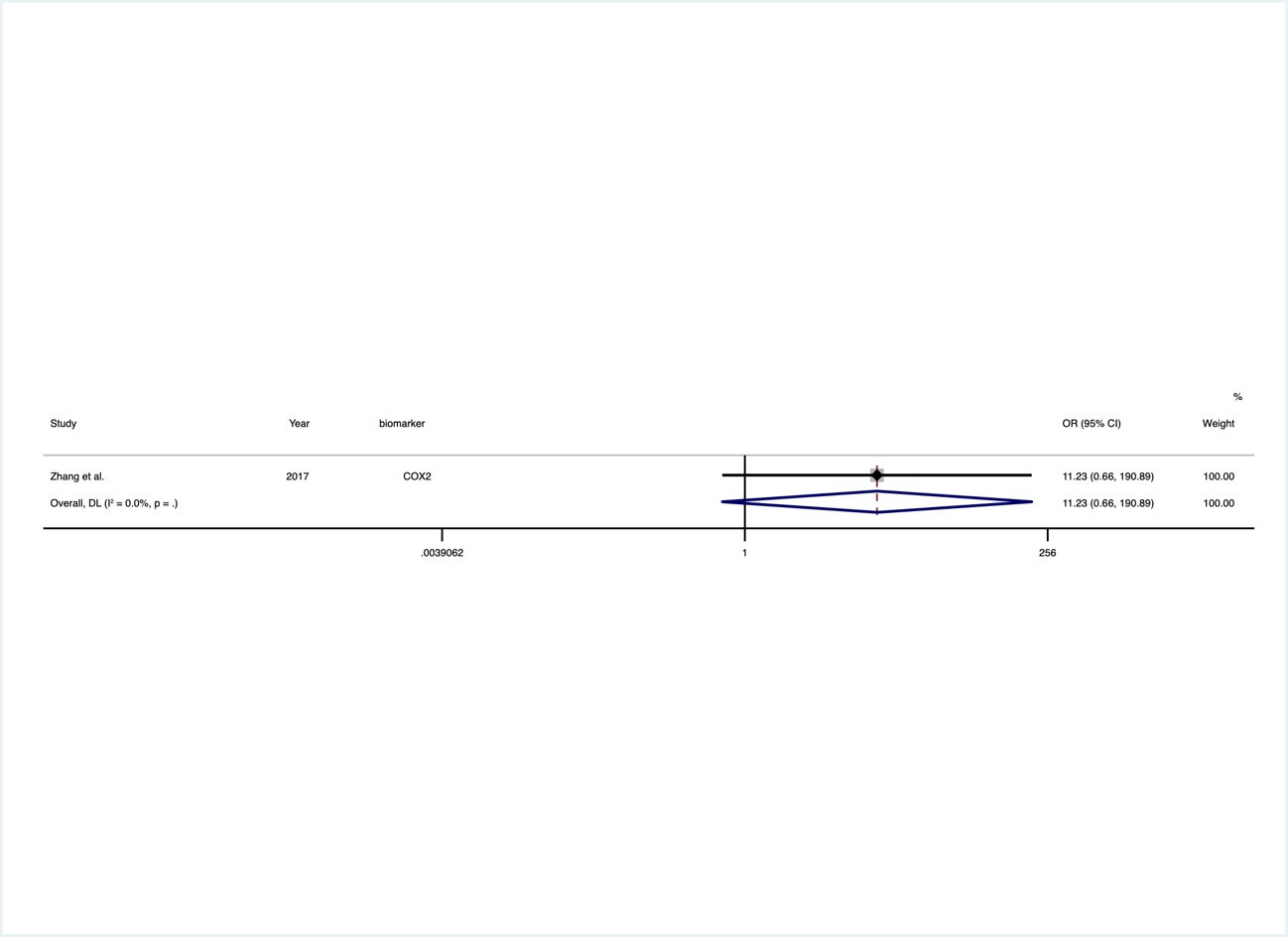


**6. Biomarkers roles in the context of hallmarks of cancer.**

**Table S4. Biomarkers roles in the context of hallmarks of cancer.**

| **Biomarker** | **oncogenic/protector**  **(function)** | **Function** | **Additional source** |
| --- | --- | --- | --- |
| 14-3-3σ | Protector  (growth suppressor) | **Cell cycle control**  14-3-3σ is an isoform of the 14-3-3 protein family. It is expressed mainly in the cytoplasm and nucleus of epithelial cells. Upon DNA damage, p53 regulates the G2/M checkpoint by inducing 14-3-3σ expression, leading to the sequestration of CDK1/cyclin B1 in the cytoplasm and blocking the entry of the cell into mitosis, allowing time for DNA repair. Furthermore, 14-3-3σ may directly increase the transcriptional activity of p53, suggesting a positive feedback loop.  In cancer, mutated p53 results in a decrease in 14-3-3σ expression, not being able to arrest the cancerous cell in the G2 phase, thus allowing the occurrence of mutations and aberrant chromosome structures. | Mhawech P. (2005). 14-3-3 proteins--an update. Cell research, 15(4), 228–236. <https://doi.org/10.1038/sj.cr.7290291>  Kaur, J., Matta, A., Kak, I., Srivastava, G., Assi, J., Leong, I., Witterick, I., Colgan, T. J., Macmillan, C., Siu, K. W., Walfish, P. G., & Ralhan, R. (2014). S100A7 overexpression is a predictive marker for high risk of malignant transformation in oral dysplasia. International journal of cancer, 134(6), 1379–1388. https://doi.org/10.1002/ijc.28473 |
| 14-3-3ζ | oncogenic  (anti-apoptotic) | **Anti-apoptotic**  14-3-3ζ is an isoform of the 14-3-3 protein family.  Overexpression of 14-3-3ζ in human cancers may contribute to transformation by inhibiting apoptosis, activating signaling pathways that promote growth, and/or sequestering tumor suppressor proteins. 14-3-3z can directly bind to Bad, the inhibition of Bcl-2 by the latter. 14-3-3z can also bind to FOXO transcription factors, sequestering them in the cytoplasm and preventing the transcription of the pro-apoptotic proteins Fas and Bax, amongst other survival-related actions. | Neal, C. L., & Yu, D. (2010). 14-3-3ζ as a prognostic marker and therapeutic target for cancer. Expert opinion on therapeutic targets, 14(12), 1343–1354. https://doi.org/10.1517/14728222.2010.531011  Kaur, J., Matta, A., Kak, I., Srivastava, G., Assi, J., Leong, I., Witterick, I., Colgan, T. J., Macmillan, C., Siu, K. W., Walfish, P. G., & Ralhan, R. (2014). S100A7 overexpression is a predictive marker for high risk of malignant transformation in oral dysplasia. International journal of cancer, 134(6), 1379–1388. https://doi.org/10.1002/ijc.28473 |
| ABCG2 | Oncogenic  (pro-invasive) | **Stem cell phenotype and multidrug resistance**  The ATP-binding cassette G2 subfamily (ABCG2), also known as the breast cancer resistance protein (BCRP), is a member of the superfamily of ATP-binding cassette (ABC).It is transporter protein that promotes the efflux of toxins and drugs into the extracellular environment through regulation of ATP hydrolysis related to the increase of migration. It is also involved in the maintenance of the phenotype of cancer stem cell populations and it may represent a mechanism of anticancer drug resistance. | Zhou, S., Schuetz, J. D., Bunting, K. D., Colapietro, A. M., Sampath, J., Morris, J. J., Lagutina, I., Grosveld, G. C., Osawa, M., Nakauchi, H., & Sorrentino, B. P. (2001). The ABC transporter Bcrp1/ABCG2 is expressed in a wide variety of stem cells and is a molecular determinant of the side-population phenotype. Nature medicine, 7(9), 1028–1034. https://doi.org/10.1038/nm0901-1028  Liu, W., Feng, J. Q., Shen, X. M., Wang, H. Y., Liu, Y., & Zhou, Z. T. (2012). Two stem cell markers, ATP-binding cassette, G2 subfamily (ABCG2) and BMI-1, predict the transformation of oral leukoplakia to cancer: a long-term follow-up study. Cancer, 118(6), 1693–1700. https://doi.org/10.1002/cncr.26483 |
| ALDH1 | Oncogenic (pro-invasive) | **Stem cell differentiation**  The ALDH1 is an isoform 1 of the enzyme aldehyde dehydrogenase, which is expressed in humans as a cytosolic detoxifying isoenzyme that oxidises intracellular aldehydes through NAD(P)+-dependent oxidation and contributes to the oxidation of retinol to retinoic acid in early stem cell differentiation. It is involved in the regulation and maintenance of cancer stem cell phenotype. | Marcato P, Dean CA, Giacomantonio CA, Lee PW. Aldehyde dehydrogenase: its role as a cancer stem cell marker comes down to the specific isoform. Cell Cycle. 2011 May 1;10(9):1378-84. doi: 10.4161/cc.10.9.15486. Epub 2011 May 1. PMID: 21552008.  Liu W, Wu L, Shen XM, Shi LJ, Zhang CP, Xu LQ, Zhou ZT. Expression patterns of cancer stem cell markers ALDH1 and CD133 correlate with a high risk of malignant transformation of oral leukoplakia. Int J Cancer. 2013 Feb 15;132(4):868-74. doi: 10.1002/ijc.27720. Epub 2012 Jul 30. PMID: 22782852. |
| ATM | Protector  (pro-stability) | **DNA damage repair**  The ataxia telangiectasia mutated (ATM) gene plays a crucial role in sensing DNA damage and transducing signals. It recognises DNA damage through the BRCA1-associated genome surveillance complex (BASC) and initiates several signal transduction pathways, producing biological consequences such as DNA repair, cell cycle arrest or apoptosis. | Canman CE, Lim DS. The role of ATM in DNA damage responses and cancer. Oncogene. 1998 Dec 24;17(25):3301-8. doi: 10.1038/sj.onc.1202577. PMID: 9916992.  He Y, Chen Q, Li B. ATM in oral carcinogenesis: association with clinicopathological features. J Cancer Res Clin Oncol. 2008 Sep;134(9):1013-20. doi: 10.1007/s00432-008-0365-7. Epub 2008 Feb 21. PMID: 18288488. |
| Axin2 | Oncogenic  (pro-invasive) | **EMT inhibitor**  Axin2 is a negative regulator of the Wnt signalling pathway, which promotes the phosphorylation and degradation of β-catenin by GSK-3 and subsequent EMT progression. When Axin2 is overexpressed, β-catenin consequently accumulates and enters the nucleus, where it interacts with genes involved in cell proliferation and tumor progression. | Jho, E. hoon, Zhang, T., Domon, C., Joo, C. K., Freund, J. N., & Costantini, F. (2002). Wnt/β-Catenin/Tcf Signaling Induces the Transcription of Axin2, a Negative Regulator of the Signaling Pathway. Molecular and Cellular Biology, 22(4), 1172–1183. <https://doi.org/10.1128/MCB.22.4.1172-1183.2002>.  Zhang X, Kim KY, Zheng Z, Kim HS, Cha IH, Yook JI. Snail and Axin2 expression predict the malignant transformation of oral leukoplakia. Oral Oncol. 2017 Oct;73:48-55. doi: 10.1016/j.oraloncology.2017.08.004. Epub 2017 Aug 12. PMID: 28939076. |
| β-catenin | Unespecified | β-Catenin forms a complex with the adhesion molecule E-cadherin, promoting cell–cell adhesion and contributing to the structural formation of the stratified squamous epithelium of the oral mucosa, also preventing the cell dissociation required for cancer invasion and progression. Besides the membrane localization, there is a dynamic pool of cytoplasmic β-catenin that serves as rapid connection between the extracellular microenvironment and nucleus through the plasma membrane. Thus, cytoplasmic β-catenin acts as signal transcription factor to the nucleus in the canonical Wnt pathway, activating the transcription of genes with various cell functions. | MacDonald, B. T., Tamai, K., & He, X. (2009). Wnt/β-catenin signaling: components, mechanisms, and diseases. Developmental cell, 17(1), 9-26.  González-Moles, M. A., Ruiz-Ávila, I., Gil-Montoya, J. A., Plaza-Campillo, J., & Scully, C. (2014). β-catenin in oral cancer: an update on current knowledge. Oral oncology, 50(9), 818-824. |
| Bcl-2 | Oncogenic (anti-apoptotic) | **Anti-apoptotic**  Bcl-2 is a well-known apoptosis inhibitor that suppresses apoptosis through the intrinsic apoptosis pathway by preventing the release of cytochrome C from mitochondria by inhibiting Bax insertion or by directly or indirectly inhibiting Bax channel activity. | Haldar S, Beatty C, Tsujimoto Y, Croce CM. The bcl-2 gene encodes a novel G protein. Nature. 1989 Nov 9;342(6246):195-8. doi: 10.1038/342195a0. PMID: 2478890.  Sulkowska M, Famulski W, Chyczewski L, Sulkowski S. Evaluation of p53 and bcl-2 oncoprotein expression in precancerous lesions of the oral cavity. Neoplasma. 2001;48(2):94-8. PMID: 11478700. |
| BMI-1 | Oncogenic (prosurvival/immortalization) | **Cell immortality**  Member of the complex PCR1 (Polycomb repressive complex 1). It is implied in the gene silencing by regulating chromatin structure. It can suppress tumor suppressor proteins and pathways (e.g. p16, pRb, p53, MDM2), thereby allowing progression through the cell cycle. It is essential for the maintenance and self-renewal implicated in mediating cellular senescence. | Jacobs, J. J., Kieboom, K., Marino, S., DePinho, R. A., & van Lohuizen, M. (1999). The oncogene and Polycomb-group gene bmi-1 regulates cell proliferation and senescence through the ink4a locus. Nature, 397(6715), 164–168. https://doi.org/10.1038/16476  Liu, W., Feng, J. Q., Shen, X. M., Wang, H. Y., Liu, Y., & Zhou, Z. T. (2012). Two stem cell markers, ATP-binding cassette, G2 subfamily (ABCG2) and BMI-1, predict the transformation of oral leukoplakia to cancer: a long-term follow-up study. Cancer, 118(6), 1693–1700. https://doi.org/10.1002/cncr.26483 |
| BUB3 | Protector  (pro-stability) | **DNA stability**  BUB3 is a key player in the spindle assembly checkpoint (SAC) mechanism. The SAC ensures the fidelity of chromosome segregation by delaying anaphase until all sister chromatid pairs are bipolarly attached. In normally cycling cells, unattached or improperly attached kinetochores and spindle damage activate the SAC, which in turn arrests cells in mitosis. The inhibitory activity of the SAC is mediated by a diffusible mitotic checkpoint complex (MCC) formed by the assembly of BubR1, Mad2 and Bub3 proteins with Cdc20, a cofactor and activator of the anaphase-promoting complex or cyclosome (APC/C). | Taylor SS, Ha E, McKeon F. The human homologue of Bub3 is required for kinetochore localization of Bub1 and a Mad3/Bub1-related protein kinase. J Cell Biol. 1998 Jul 13;142(1):1-11. doi: 10.1083/jcb.142.1.1. PMID: 9660858; PMCID: PMC2133037.  Monteiro L, Silva P, Delgado L, Amaral B, Garcês F, Salazar F, Pacheco JJ, Lopes C, Bousbaa H, Warnakulasuriya S. Expression of spindle assembly checkpoint proteins BubR1 and Mad2 expression as potential biomarkers of malignant transformation of oral leukoplakia: an observational cohort study. Med Oral Patol Oral Cir Bucal. 2021 Nov 1;26(6):e719-e728. doi: 10.4317/medoral.24511. PMID: 34704983; PMCID: PMC8601638. |
| BUBR1 | Protector (Pro-Stability) | **Protection Against Genomic Instability**  BubR1 is an essential component of the spindle assembly checkpoint, which ensures correct chromosome segregation by preventing the transition to anaphase if the kinetochores are not properly attached. In addition, it facilitates the formation of stable kinetochore-microtubule junctions during prometaphase. BubR1 also plays important roles in meiosis, DNA damage response and centrosome regulation. | Karess RE, Wassmann K, Rahmani Z. New insights into the role of BubR1 in mitosis and beyond. Int Rev Cell Mol Biol. 2013;306:223-73. doi: 10.1016/B978-0-12-407694-5.00006-7. PMID: 24016527.  Monteiro L, Silva P, Delgado L, Amaral B, Garcês F, Salazar F, Pacheco JJ, Lopes C, Bousbaa H, Warnakulasuriya S. Expression of spindle assembly checkpoint proteins BubR1 and Mad2 expression as potential biomarkers of malignant transformation of oral leukoplakia: an observational cohort study. Med Oral Patol Oral Cir Bucal. 2021 Nov 1;26(6):e719-e728. doi: 10.4317/medoral.24511. PMID: 34704983; PMCID: PMC8601638. |
| c-jun | Oncogenic (pro-proliferative) | **Cell cycle progression**  c-Jun plays a crucial role in regulating the cell cycle, acting as a positive regulator of the cell cycle downstream of the MAP kinase and Akt pathways. C-Jun forms the major component of the activator protein 1 (AP-1) transcription factor, which contributes to cell proliferation through its ability to regulate the expression and function of cell cycle regulators such as cyclin D1, p53 and the inhibitors p21waf1, p19ARF, p27 and p16INK. | Wisdom R, Johnson RS, Moore C. c-Jun regulates cell cycle progression and apoptosis by distinct mechanisms. EMBO J. 1999 Jan 4;18(1):188-97. doi: 10.1093/emboj/18.1.188. PMID: 9878062; PMCID: PMC1171114.  Lima JS, Correa L, Klingbeil MF, de Sousa SC. c-Jun, pc-Jun, and p27 are differently expressed in oral leukoplakias in smokers and never-smokers. Oral Surg Oral Med Oral Pathol Oral Radiol. 2016 Jan;121(1):73-80. doi: 10.1016/j.oooo.2015.09.003. Epub 2015 Sep 16. PMID: 26679360. |
| c-met | Oncogenic  (pro-angiogenic) | **Formation of new blood vessels**  It is a surface receptor tyrosine kinase which triggers the formation of new blood vessels that supply nutrients to the tumor and allows it to grow and to spread throughout multiple signaling pathways directly, by motogenic or morphogenic effects, or indirectly by the regulation of other angiogenic factors, turning into the stimulation of endothelial cells. | Bottaro, D. P., Rubin, J. S., Faletto, D. L., Chan, A. M. L., Kmiecik, T. E., Vande Woude, G. F., & Aaronson, S. A. (1991). Identification of the hepatocyte growth factor receptor as the c-met proto-oncogene product. science, 251(4995), 802-804.  Zhang, X., Kim, K. Y., Zheng, Z., Bazarsad, S., & Kim, J. (2017). Nomogram for risk prediction of malignant transformation in oral leukoplakia patients using combined biomarkers. Oral oncology, 72, 132–139. https://doi.org/10.1016/j.oraloncology.2017.07.015 |
| CA9 | Oncogenic  (enhancing tumor  acidosis) | **Maintenance of intracellular acidosis.**  Carbonic anhydrase 9 is cell-surface glycoprotein that is induced by hypoxia, involved in adaptation to acidosis and implicated in cancer progression via its catalytic activity and/or non-catalytic functions.. It that can induce reversible hydration of carbon dioxide to carbonic acid, thereby creating an acidic microenvironment, allowing the cell to adapt to aerobic glycolysis and simultaneously facilitate CO2 diffusion and proton mobility in the tumor tissue.It enables the tumors to rapidly outgrow blood supply correlating with cell survival. | Pastorekova, S., & Gillies, R. J. (2019). The role of carbonic anhydrase IX in cancer development: links to hypoxia, acidosis, and beyond. Cancer metastasis reviews, 38(1-2), 65–77. https://doi.org/10.1007/s10555-019-09799-0  Zhang, X., Han, S., Han, H. Y., Ryu, M. H., Kim, K. Y., Choi, E. J., ... & Kim, J. (2013). Risk prediction for malignant conversion of oral epithelial dysplasia by hypoxia related protein expression. Pathology-Journal of the RCPA, 45(5), 478-483. |
| CD11C | Oncogenic  (pro-inflammatory) | **Inflammatory response**  Cluster of differentiation 11c is a transmembrane glycoprotein that is established as a marker for M1 polarized tissue macrophages that promote inflammatory reactions. | Skytthe, M. K., Graversen, J. H., & Moestrup, S. K. (2020). Targeting of CD163+ macrophages in inflammatory and malignant diseases. International journal of molecular sciences, 21(15), 5497.  Weber, M., Wehrhan, F., Baran, C., Agaimy, A., Büttner-Herold, M., Öztürk, H., Neubauer, K., Wickenhauser, C., Kesting, M., & Ries, J. (2020). Malignant transformation of oral leukoplakia is associated with macrophage polarization. Journal of translational medicine, 18(1), 11. https://doi.org/10.1186/s12967-019-02191-0 |
| PROM1 | Oncogenic  (pro-invasive) | **Cell migration and invasion activation**  One of the prototypical cancer stem cell biomarkers, which among its multiple roles activates the canonical Wnt oncogenic signalling pathway. It has been associated with increased cell migration and metastasis. | Singh, A., & Settleman, J. E. M. T. (2010). EMT, cancer stem cells and drug resistance: an emerging axis of evil in the war on cancer. Oncogene, 29(34), 4741-4751.  Monteiro, L., do Amaral, B., Delgado, L., Garcês, F., Salazar, F., Pacheco, J. J., Lopes, C., & Warnakulasuriya, S. (2022). Podoplanin Expression Independently and Jointly with Oral Epithelial Dysplasia Grade Acts as a Potential Biomarker of Malignant Transformation in Oral Leukoplakia. Biomolecules, 12(5), 606. https://doi.org/10.3390/biom12050606 |
| BSG | Oncogenic  (pro-invasive) | **Cell migration and invasion activation**  BSG (basigin) , also known as CD147 or EMMPRIN, is a glycoprotein of the immunoglobulin superfamily, that can be present as a transmembrane molecule (acting via cell-cell interactions) or released in a soluble form by tumor cells. It stimulates the production of MMPs by fibroblasts, participating in invasion and metastasis by facilitating tumor cell motility through the surrounding matrix. | Biswas, C., Zhang, Y., DeCastro, R., Guo, H., Nakamura, T., Kataoka, H., & Nabeshima, K. (1995). The human tumor cell-derived collagenase stimulatory factor (renamed EMMPRIN) is a member of the immunoglobulin superfamily. Cancer research, 55(2), 434-439.  Monteiro, L., do Amaral, B., Delgado, L., Garcês, F., Salazar, F., Pacheco, J. J., Lopes, C., & Warnakulasuriya, S. (2022). Podoplanin Expression Independently and Jointly with Oral Epithelial Dysplasia Grade Acts as a Potential Biomarker of Malignant Transformation in Oral Leukoplakia. Biomolecules, 12(5), 606. https://doi.org/10.3390/biom12050606 |
| CD163 | Oncogenic  (anti-tumor arrest) | **Inflammatory response**  Cluster of differentiation 11c is a transmembrane glycoprotein that is established as a marker for polarized M2 macrophages. These have immunoregulatory properties during immunosuppression and tumor progression. | Skytthe, M. K., Graversen, J. H., & Moestrup, S. K. (2020). Targeting of CD163+ macrophages in inflammatory and malignant diseases. International journal of molecular sciences, 21(15), 5497.  Weber, M., Wehrhan, F., Baran, C., Agaimy, A., Büttner-Herold, M., Öztürk, H., Neubauer, K., Wickenhauser, C., Kesting, M., & Ries, J. (2020). Malignant transformation of oral leukoplakia is associated with macrophage polarization. Journal of translational medicine, 18(1), 11. https://doi.org/10.1186/s12967-019-02191-0 |
| CD3 | Oncogenic  (pro-inflammatory) | **Inflammatory response**  Cluster of differentiation 3 is a protein complex that is expressed in the surface of T cells, serving in their differentiation, survival, and function. T cells play a crucial role in the adaptive immune response. | Call, M. E., Pyrdol, J., Wiedmann, M., & Wucherpfennig, K. W. (2002). The organizing principle in the formation of the T cell receptor-CD3 complex. Cell, 111(7), 967-979.  Xu, S. B., Wang, M. Y., Shi, X. Z., Wang, Q., Yu, M., Zhang, W., Xu, X. H., & Liu, L. K. (2023). Influence of PD-1/PD-L1 on immune microenvironment in oral leukoplakia and oral squamous cell carcinoma. Oral diseases, 29(8), 3268–3277. https://doi.org/10.1111/odi.14332 |
| CD44 (v6) | Unespecified | CDDv6 is an adhesion molecule from the CD44 family,  a transmembrane glycoprotein involved in cell-cell and cell-matrix interactions, adhesion and migration, cell growth, EMT, and even tumor progression . It is a receptor for many ligands such as hyaluronic acid, osteopontine, collagens and matrix metalloproteinases. | Todaro, M., Gaggianesi, M., Catalano, V., Benfante, A., Iovino, F., Biffoni, M., Apuzzo, T., Sperduti, I., Volpe, S., Cocorullo, G., Gulotta, G., Dieli, F., De Maria, R., & Stassi, G. (2014). CD44v6 is a marker of constitutive and reprogrammed cancer stem cells driving colon cancer metastasis. Cell stem cell, 14(3), 342–356. <https://doi.org/10.1016/j.stem.2014.01.009>  Monteiro, L., do Amaral, B., Delgado, L., Garcês, F., Salazar, F., Pacheco, J. J., Lopes, C., & Warnakulasuriya, S. (2022). Podoplanin Expression Independently and Jointly with Oral Epithelial Dysplasia Grade Acts as a Potential Biomarker of Malignant Transformation in Oral Leukoplakia. Biomolecules, 12(5), 606. https://doi.org/10.3390/biom12050606 |
| CD68 | Oncogenic  (pro-inflammatory) | **Inflammatory response**  Cluster of differentiation 68 is a transmembrane glycoprotein that is established as pan-macrophage marker to detect monocytes and macrophages. CD68 plays a key role in phagocytosis and inflammation, making it useful in inflammatory diseases and tumor-associated macrophage studies. | Skytthe, M. K., Graversen, J. H., & Moestrup, S. K. (2020). Targeting of CD163+ macrophages in inflammatory and malignant diseases. International journal of molecular sciences, 21(15), 5497.  Weber, M., Wehrhan, F., Baran, C., Agaimy, A., Büttner-Herold, M., Öztürk, H., Neubauer, K., Wickenhauser, C., Kesting, M., & Ries, J. (2020). Malignant transformation of oral leukoplakia is associated with macrophage polarization. Journal of translational medicine, 18(1), 11. https://doi.org/10.1186/s12967-019-02191-0 |
| CD8 | Oncogenic  (pro-inflammatory) | **Inflammatory response**  Cluster of differentiation 8 is a transmembrane protein that serves as a co-receptor of cytotoxic T cells, contributing to antigen recognition, immune cell maturation, and immune cell signaling. CD8+ are able to directly kill infected and cancerous cells, as well as to recruit other cells via cytokine secretion. | Srinivasan, S., Zhu, C., & McShan, A. C. (2024). Structure, function, and immunomodulation of the CD8 co-receptor. Frontiers in immunology, 15, 1412513. https://doi.org/10.3389/fimmu.2024.1412513  Xu, S. B., Wang, M. Y., Shi, X. Z., Wang, Q., Yu, M., Zhang, W., Xu, X. H., & Liu, L. K. (2023). Influence of PD-1/PD-L1 on immune microenvironment in oral leukoplakia and oral squamous cell carcinoma. Oral diseases, 29(8), 3268–3277. https://doi.org/10.1111/odi.14332 |
| CK8 | Unespecified | CK8 (cytokeratin 8) belongs to the basic (type II) keratin subfamily. It is predominantly co-expressed with CK18 (cytokeratin 18) as the primary keratin pair in simple epithelial cells. CK8 and CK18 play crucial roles in maintaining epithelial cellular structural integrity and also in signal transduction and cellular differentiation. | Moll R, Franke WW, Schiller DL, Geiger B, Krepler R. The catalog of human cytokeratins: patterns of expression in normal epithelia, tumors and cultured cells. Cell. 1982 Nov;31(1):11-24. doi: 10.1016/0092-8674(82)90400-7. PMID: 6186379.  Matthias C, Mack B, Berghaus A, Gires O. Keratin 8 expression in head and neck epithelia. BMC Cancer. 2008 Sep 22;8:267. doi: 10.1186/1471-2407-8-267. PMID: 18803884; PMCID: PMC2556347. |
| CK13 | Unespecified | Cytokeratins (CKs) are intermediate filaments that protect cells against mechanical stress, contributing to the maintenance of tissue integrity and regulating cell shape, adhesion, and migration processes. It is also implicated in epithelial differentiation and wound healing mechanisms.  Cytokeratin 13 (CK13), is a low molecular weight cytokeratin that is mucosa specific and expressed in the suprabasal layers of non-keratinized stratified epithelia. Loss of staining is translated as loss of stratification in a squamous epithelium. | Kitamura, R., Toyoshima, T., Tanaka, H., Kawano, S., Kiyosue, T., Matsubara, R., ... & Nakamura, S. (2012). Association of cytokeratin 17 expression with differentiation in oral squamous cell carcinoma. Journal of cancer research and clinical oncology, 138, 1299-1310.  Shigeoka, M., Koma, Y. I., Kodama, T., Nishio, M., Akashi, M., & Yokozaki, H. (2020). Intraepithelial CD163+ macrophages in tongue leukoplakia biopsy: A promising tool for cancer screening. Oral diseases, 26(3), 527–536. https://doi.org/10.1111/odi.13269 |
| CK17 | Unespecified | Cytokeratins (CKs) are intermediate filaments that protect cells against mechanical stress, contributing to the maintenance of tissue integrity and regulating cell shape, adhesion, and migration processes. It is also implicated in epithelial differentiation and wound healing mechanisms. Cytokeratin 17 (CK17) is a protein whose expression is restricted to the basal layer in normal tissues. | Troyanovsky, S. M., Guelstein, V. I., Tchipysheva, T. A., Krutovskikh, V. A., & Bannikov, G. A. (1989). Patterns of expression of keratin 17 in human epithelia: dependency on cell position. Journal of cell science, 93(3), 419-426.  Shigeoka, M., Koma, Y. I., Kodama, T., Nishio, M., Akashi, M., & Yokozaki, H. (2020). Intraepithelial CD163+ macrophages in tongue leukoplakia biopsy: A promising tool for cancer screening. Oral diseases, 26(3), 527–536. https://doi.org/10.1111/odi.13269 |
| COX-2 | Oncogenic  (pro-inflammatory) | **Inflammation and tumour growth promotion**  COX2 is the enzyme responsible for regulating the metabolism of arachidonic acid and its conversion to prostaglandin E2, a potent inducer of the inflammatory response, implicated in numerous related processes (pain, fever, vasodilatation, increased tissue permeability allowing the entry of leukocytes, among others). Numerous specific oncogenic actions have been attributed to COX2 and its product, such as activation of cell proliferation and growth, inhibition of apoptosis, cell migration and neoangiogenesis. | Hla, T., & Neilson, K. (1992). Human cyclooxygenase-2 cDNA. Proceedings of the national academy of sciences, 89(16), 7384-7388.  Zhang, X., Kim, K. Y., Zheng, Z., Bazarsad, S., & Kim, J. (2017). Nomogram for risk prediction of malignant transformation in oral leukoplakia patients using combined biomarkers. Oral oncology, 72, 132–139. https://doi.org/10.1016/j.oraloncology.2017.07.015 |
| Cyclin D1 | Oncogenic  (pro-proliferative) | **Cell cycle progression**  Cyclin D1 plays a vital role in the progression from G1 to S phase of the cell cycle by forming complexes with CDK4 and CDK6. The formation of Cyclin D1-CDK4/6 complexes initiates phosphorylation-dependent inactivation of the retinoblastoma tumour suppressor protein (pRb), leading to dissociation of the pRb-E2F complex and allowing progression from G1 to S phase. | Musgrove EA, Caldon CE, Barraclough J, Stone A, Sutherland RL. Cyclin D as a therapeutic target in cancer. Nat Rev Cancer. 2011 Jul 7;11(8):558-72. doi: 10.1038/nrc3090. PMID: 21734724.  Soni S, Kaur J, Kumar A, Chakravarti N, Mathur M, Bahadur S, Shukla NK, Deo SV, Ralhan R. Alterations of rb pathway components are frequent events in patients with oral epithelial dysplasia and predict clinical outcome in patients with squamous cell carcinoma. Oncology. 2005;68(4-6):314-25. doi: 10.1159/000086970. Epub 2005 Jul 12. PMID: 16020958. |
| DcR2 | Oncogenic (anti-apoptotic) | **Anti-Apoptotic**  DcR2, also known as Decoy receptor 2 or TRAIL-R4, acts as a dominant negative receptor for TRAIL. It confers resistance to TRAIL-mediated apoptosis and attenuates cell response to DNA-damaging agents. | van Noesel MM, van Bezouw S, Salomons GS, Voûte PA, Pieters R, Baylin SB, Herman JG, Versteeg R. Tumor-specific down-regulation of the tumor necrosis factor-related apoptosis-inducing ligand decoy receptors DcR1 and DcR2 is associated with dense promoter hypermethylation. Cancer Res. 2002 Apr 1;62(7):2157-61. PMID: 11929838.  de Villalaín L, Álvarez-Teijeiro S, Rodríguez-Santamarta T, Fernández Del Valle Á, Allonca E, Rodrigo JP, de Vicente JC, García-Pedrero JM. Emerging Role of Decoy Receptor-2 as a Cancer Risk Predictor in Oral Potentially Malignant Disorders. Int J Mol Sci. 2023 Sep 21;24(18):14382. doi: 10.3390/ijms241814382. PMID: 37762685; PMCID: PMC10531848. |
| Dec1 | Unespecified | DEC1 (differentiated embryo-chondrocyte expressed gene 1) is a transcription factor that belongs to a subfamily of basic helix-loop-helix transcription factors. DEC1 is a pleiotropic transcription factor with multiple functions. It acts as a transcriptional repressor that regulates cell cycle, differentiation and apoptosis in response to various stimuli. Depending on the tissue and cellular context, DEC1 can have pro-apoptotic or pro-survival activities. DEC1 is also a marker of senescence and is overexpressed in premalignant lesions. | Qian Y, Zhang J, Yan B, Chen X. DEC1, a basic helix-loop-helix transcription factor and a novel target gene of the p53 family, mediates p53-dependent premature senescence. J Biol Chem. 2008 Feb 1;283(5):2896-905. doi: 10.1074/jbc.M708624200. Epub 2007 Nov 19. PMID: 18025081; PMCID: PMC4118587.  de Villalaín L, Álvarez-Teijeiro S, Rodríguez-Santamarta T, Fernández Del Valle Á, Allonca E, Rodrigo JP, de Vicente JC, García-Pedrero JM. Emerging Role of Decoy Receptor-2 as a Cancer Risk Predictor in Oral Potentially Malignant Disorders. Int J Mol Sci. 2023 Sep 21;24(18):14382. doi: 10.3390/ijms241814382. PMID: 37762685; PMCID: PMC10531848. |
| EGFR | Oncogenic  (pro-proliferative) | **Cell cycle progression**  The epidermal growth factor receptor (EGFR) is transmembrane receptor with tyrosine kinase activity. The binding of ligands to its extracellular domain, activates a number of molecular signalling pathways that stimulate cell proliferation (e.g, the MAPK pathway). | Yarden, Y., & Sliwkowski, M. X. (2001). Untangling the ErbB signalling network. Nature reviews. Molecular cell biology, 2(2), 127–137. <https://doi.org/10.1038/35052073>.  Monteiro L, do Amaral B, Delgado L, Garcês F, Salazar F, Pacheco JJ, Lopes C, Warnakulasuriya S. Podoplanin Expression Independently and Jointly with Oral Epithelial Dysplasia Grade Acts as a Potential Biomarker of Malignant Transformation in Oral Leukoplakia. Biomolecules. 2022 Apr 19;12(5):606. doi: 10.3390/biom12050606. PMID: 35625534; PMCID: PMC9138639. |
| EZH2 | Oncogenic  (pro-proliferative) | **Growth promoter**  Enhancer of Zeste Homolog 2 (EZH2) is the catalytic subunit of Polycomb repressive complex 2 (PRC2), a highly conserved histone methyltransferase that methylates lysine-27 of histone H3 (H3-K27). H3-K27 methylation is often associated with DNA methylation and silencing of a subset of genes involved in cell differentiation including many tumour growth suppressors. | Kim KH, Roberts CW. Targeting EZH2 in cancer. Nat Med. 2016 Feb;22(2):128-34. doi: 10.1038/nm.4036. PMID: 26845405; PMCID: PMC4918227.  Cao W, Younis RH, Li J, Chen H, Xia R, Mao L, Chen W, Ren H. EZH2 promotes malignant phenotypes and is a predictor of oral cancer development in patients with oral leukoplakia. Cancer Prev Res (Phila). 2011 Nov;4(11):1816-24. doi: 10.1158/1940-6207.CAPR-11-0130. Epub 2011 Jun 22. PMID: 21697275; PMCID: PMC3677701. |
| FGFR1 | Oncogenic  (pro-proliferative) | **Cell proliferation**  The Fibroblast Growth Factor Receptor (FGFR) family of transmembrane tyrosine kinase receptors that regulate several physiological and pathological processes, including embryogenic events, cell proliferation, wound healing, amongst others. FGFR activates the upregulation of MAPK and PI3K/Akt, the most relevant pro-proliferative molecular signaling pathways in human cancer. | Turner, N., & Grose, R. (2010). Fibroblast growth factor signalling: from development to cancer. Nature Reviews Cancer, 10(2), 116-129.  Mariz, B. A. L. A., Sales de Sá, R., Araújo, A. L. D., Fernandes, C. I. R., Mariano, F. V., Santos-Silva, A. R., Lopes, M. A., Vargas, P. A., de Almeida, O. P., Kowalski, L. P., & Jorge, J. (2023). FGFR1 is an important prognostic factor in oral leukoplakia and tongue squamous cell carcinoma. Journal of oral pathology & medicine : official publication of the International Association of Oral Pathologists and the American Academy of Oral Pathology, 52(2), 119–126. <https://doi.org/10.1111/jop.13398> |
| FGF2 | Oncogenic  (pro-proliferative) | **Cell proliferation**  Fibroblast growth factor-2 (FGF-2) is a secreted glycoprotein, that, stabilized by heparan sulphate proteoglycans (HPSGs), leading to the  activation of multiple signal transduction pathways of FGFR-2. | Turner, N., & Grose, R. (2010). Fibroblast growth factor signalling: from development to cancer. Nature Reviews Cancer, 10(2), 116-129.  Nayak, S., Goel, M. M., Makker, A., Bhatia, V., Chandra, S., Kumar, S., & Agarwal, S. P. (2015). Fibroblast Growth Factor (FGF-2) and Its Receptors FGFR-2 and FGFR-3 May Be Putative Biomarkers of Malignant Transformation of Potentially Malignant Oral Lesions into Oral Squamous Cell Carcinoma. PloS one, 10(10), e0138801. https://doi.org/10.1371/journal.pone.0138801 |
| FGFR2 | Oncogenic  (pro-proliferative) | **Cell proliferation**  The Fibroblast Growth Factor Receptor (FGFR) family of transmembrane tyrosine kinase receptors that regulate several physiological and pathological processes, including embryogenic events, cell proliferation, wound healing, amongst others. FGFR activates the upregulation of MAPK and PI3K/Akt, the most relevant pro-proliferative molecular signaling pathways in human cancer. | Turner, N., & Grose, R. (2010). Fibroblast growth factor signalling: from development to cancer. Nature Reviews Cancer, 10(2), 116-129.  Nayak, S., Goel, M. M., Makker, A., Bhatia, V., Chandra, S., Kumar, S., & Agarwal, S. P. (2015). Fibroblast Growth Factor (FGF-2) and Its Receptors FGFR-2 and FGFR-3 May Be Putative Biomarkers of Malignant Transformation of Potentially Malignant Oral Lesions into Oral Squamous Cell Carcinoma. PloS one, 10(10), e0138801. https://doi.org/10.1371/journal.pone.0138801 |
| FGFR3 | Oncogenic  (pro-proliferative) | **Cell proliferation**  The Fibroblast Growth Factor Receptor (FGFR) family of transmembrane tyrosine kinase receptors that regulate several physiological and pathological processes, including embryogenic events, cell proliferation, wound healing, amongst others. FGFR activates the upregulation of MAPK and PI3K/Akt, the most relevant pro-proliferative molecular signaling pathways in human cancer. | Turner, N., & Grose, R. (2010). Fibroblast growth factor signalling: from development to cancer. Nature Reviews Cancer, 10(2), 116-129.  Nayak, S., Goel, M. M., Makker, A., Bhatia, V., Chandra, S., Kumar, S., & Agarwal, S. P. (2015). Fibroblast Growth Factor (FGF-2) and Its Receptors FGFR-2 and FGFR-3 May Be Putative Biomarkers of Malignant Transformation of Potentially Malignant Oral Lesions into Oral Squamous Cell Carcinoma. PloS one, 10(10), e0138801. https://doi.org/10.1371/journal.pone.0138801 |
| FOXP3 | Oncogenic  (anti-tumor arrest) | **Tumour microenvironment regulator**  The transcription factor forkhead box P3 constitutes an important marker, which participates in the differentiation and functioning of regulatory T cells (Tregs, CD4+, CD25+), which exert regulatory roles by being capable of suppressing immune responses . | Ziegler, S. F. (2006). FOXP3: of mice and men. Annu. Rev. Immunol., 24(1), 209-226.  Xu, S. B., Wang, M. Y., Shi, X. Z., Wang, Q., Yu, M., Zhang, W., Xu, X. H., & Liu, L. K. (2023). Influence of PD-1/PD-L1 on immune microenvironment in oral leukoplakia and oral squamous cell carcinoma. Oral diseases, 29(8), 3268–3277. https://doi.org/10.1111/odi.14332 |
| KCNH2 | Oncogenic  (pro-proliferative) | **Cell cycle progression**  Also known as KCNH2 or the human EAG-related gene (HERG1, Kv11.1) , is transmembrane protein , part of the voltage-gated potassium channels (Kv) subfamily. It acts to conduct potassium out of the cell, thereby depolarizing the cell membrane and playing a role in maintaining the resting membrane potential. They are involved in mitogenesis and volume regulation in lymphocytes, among other functions. By governing potassium ion flow and intracellular osmolarity that drive water flow across the membrane, potassium channels are central regulators for cell volume dynamics important for cell cycle progression. | Urrego, D., Tomczak, A. P., Zahed, F., Stühmer, W., & Pardo, L. A. (2014). Potassium channels in cell cycle and cell proliferation. Philosophical transactions of the Royal Society of London. Series B, Biological sciences, 369(1638), 20130094. https://doi.org/10.1098/rstb.2013.0094  Fernández-Valle, Á., Rodrigo, J. P., Rodríguez-Santamarta, T., Villaronga, M. Á., Álvarez-Teijeiro, S., García-Pedrero, J. M., Suárez-Fernández, L., Lequerica-Fernández, P., & de Vicente, J. C. (2016). HERG1 potassium channel expression in potentially malignant disorders of the oral mucosa and prognostic relevance in oral squamous cell carcinoma. Head & neck, 38(11), 1672–1678. https://doi.org/10.1002/hed.24493 |
| hnRNP K | Protector  (pro-stability) | **DNA damage repair**  Heterogeneous ribonucleoprotein K (hnRNP K) is a member of the hnRNP family. It is found to be associated with actively proliferating cells and it is involved in mechanisms implicated in gene regulation via interactions with chromatin remodeling factors, it acts as a transcriptional regulator and it is involved in mRNA stability, splicing and translation. | Barboro, P., Ferrari, N., & Balbi, C. (2014). Emerging roles of heterogeneous nuclear ribonucleoprotein K (hnRNP K) in cancer progression. Cancer letters, 352(2), 152–159. <https://doi.org/10.1016/j.canlet.2014.06.019>  Kaur, J., Matta, A., Kak, I., Srivastava, G., Assi, J., Leong, I., Witterick, I., Colgan, T. J., Macmillan, C., Siu, K. W., Walfish, P. G., & Ralhan, R. (2014). S100A7 overexpression is a predictive marker for high risk of malignant transformation in oral dysplasia. International journal of cancer, 134(6), 1379–1388. https://doi.org/10.1002/ijc.28473 |
| Ki-67 | Oncogenic  (pro-proliferative) | **Proliferation Indicator**  The Ki-67 protein is a proliferation marker in epithelial cells. It is expressed during all active phases of the cell cycle and plays a role in the regulation of cell proliferation. | Scholzen T, Gerdes J. The Ki-67 protein: from the known and the unknown. J Cell Physiol. 2000 Mar;182(3):311-22. doi: 10.1002/(SICI)1097-4652(200003)182:3<311::AID-JCP1>3.0.CO;2-9. PMID: 10653597.  de Villalaín L, Álvarez-Teijeiro S, Rodríguez-Santamarta T, Fernández Del Valle Á, Allonca E, Rodrigo JP, de Vicente JC, García-Pedrero JM. Emerging Role of Decoy Receptor-2 as a Cancer Risk Predictor in Oral Potentially Malignant Disorders. Int J Mol Sci. 2023 Sep 21;24(18):14382. doi: 10.3390/ijms241814382. PMID: 37762685; PMCID: PMC10531848. |
| KCNC4 | Oncogenic  (pro-proliferative) | **Cell cycle progression**  Also known as KCNC4, is transmembrane protein , part of the voltage-gated potassium channels (Kv) subfamily. It acts to conduct potassium out of the cell, thereby depolarizing the cell membrane and playing a role in maintaining the resting membrane potential. They are involved in mitogenesis and volume regulation in lymphocytes, among other functions. By governing potassium ion flow and intracellular osmolarity that drive water flow across the membrane, potassium channels are central regulators for cell volume dynamics important for cell cycle progression. | Chang, K. W., Yuan, T. C., Fang, K. P., Yang, F. S., Liu, C. J., Chang, C. S., & Lin, S. C. (2003). The increase of voltage‐gated potassium channel Kv3. 4 mRNA expression in oral squamous cell carcinoma. Journal of oral pathology & medicine, 32(10), 606-611.  Fernández-Valle, Á., Rodrigo, J. P., García-Pedrero, J. M., Rodríguez-Santamarta, T., Allonca, E., Lequerica-Fernández, P., & de Vicente, J. C. (2016). Expression of the voltage-gated potassium channel Kv3.4 in oral leucoplakias and oral squamous cell carcinomas. Histopathology, 69(1), 91–98. https://doi.org/10.1111/his.12917 |
| LAMC2 | Unspecified | **Cell cycle progression**  Laminin gamma chain 2 (LAMC2) is a basal lamina glycoprotein that plays dual roles in health and disease, including cancer progression. In health conditions regulate tissue homeostasis in oral mucosa, serving as a support structure and union between the epithelium and the subjacent connective tissue. On the other hand, elevated LAMC2 expression drives tumorigenesis through interactions with several cell surface receptors, including α6β4 and α3β1 integrins and EGFR. | Colognato H, Yurchenco PD. Form and function: the laminin family of heterotrimers. Dev Dyn. 2000 Jun;218(2):213-34. doi: 10.1002/(SICI)1097-0177(200006)218:2<213::AID-DVDY1>3.0.CO;2-R. PMID: 10842354.  Nguyen CT, Okamura T, Morita KI, Yamaguchi S, Harada H, Miki Y, Izumo T, Kayamori K, Yamaguchi A, Sakamoto K. LAMC2 is a predictive marker for the malignant progression of leukoplakia. J Oral Pathol Med. 2017 Mar;46(3):223-231. doi: 10.1111/jop.12485. Epub 2016 Aug 16. PMID: 27529842. |
| Mad2 | Protector  (pro-stability) | **DNA stability**  Mad2 is a key player in the spindle assembly checkpoint (SAC) mechanism that ensures the fidelity of chromosome segregation by delaying anaphase until all sister chromatid pairs are bipolarly attached. In normally cycling cells, unattached or improperly attached kinetochores and spindle damage activate the SAC, which in turn arrests cells in mitosis. This inhibitory activity of the SAC is mediated by a diffusible mitotic checkpoint complex (MCC) formed by the assembly of BubR1, Mad2 and Bub3 proteins with Cdc20, a cofactor and activator of the anaphase-promoting complex or cyclosome (APC/C). | De Antoni A, Pearson CG, Cimini D, Canman JC, Sala V, Nezi L, Mapelli M, Sironi L, Faretta M, Salmon ED, Musacchio A. The Mad1/Mad2 complex as a template for Mad2 activation in the spindle assembly checkpoint. Curr Biol. 2005 Feb 8;15(3):214-25. doi: 10.1016/j.cub.2005.01.038. PMID: 15694304.  Monteiro L, Silva P, Delgado L, Amaral B, Garcês F, Salazar F, Pacheco JJ, Lopes C, Bousbaa H, Warnakulasuriya S. Expression of spindle assembly checkpoint proteins BubR1 and Mad2 expression as potential biomarkers of malignant transformation of oral leukoplakia: an observational cohort study. Med Oral Patol Oral Cir Bucal. 2021 Nov 1;26(6):e719-e728. doi: 10.4317/medoral.24511. PMID: 34704983; PMCID: PMC8601638. |
| MAGE-A | Oncogenic  (DNA-Instability) | **DNA instability**  MAGE-A is part of the melanoma-associated antigens (MAGE) family. It has specific functions in particular somatic tissues (such as prostate and thyroid) and it intervenes during embryogenesis. Its expression is reactivated in developing cancer cells and is often associated with malignant transformation. MAGE-A facilitates the acquisition of gene mutations via p53-mediated transcription repression | Meek, D. W., & Marcar, L. (2012). MAGE-A antigens as targets in tumour therapy. Cancer letters, 324(2), 126-132.  Ries, J., Schultze-Mosgau, S., Neukam, F., Diebel, E., & Wiltfang, J. (2005). Investigation of the expression of melanoma antigen-encoding genes (MAGE-A1 to-A6) in oral squamous cell carcinomas to determine potential targets for gene-based cancer immunotherapy. International journal of oncology, 26(3), 817-824. |
| MDM2 | Oncogenic (anti-apoptotic) | **Anti-apoptotic**  The MDM2 oncoprotein plays a pivotal role in regulating the p53 protein. MDM2 binds to p53, thereby blocking its function as a tumour suppressor and promoting its own degradation. Consequently, the p53 protein is unable to initiate its cell cycle arrest or apoptotic (programmed cell death) functions. | Freedman, D. A., Wu, L., & Levine, A. J. (1999). Functions of the MDM2 oncoprotein. Cellular and Molecular Life Sciences CMLS, 55, 96-107.  Tanda N, Mori S, Saito K, Ikawa K, Sakamoto S. Expression of apoptotic signaling proteins in leukoplakia and oral lichen planus: quantitative and topographical studies. J Oral Pathol Med. 2000 Sep;29(8):385-93. doi: 10.1034/j.1600-0714.2000.290804.x. PMID: 10972347. |
| NANOG | Oncogenic  (pro-invasive) | **Cancer stem cell and EMT regulation**  NANOG plays a pivotal role in the maintenance of pluripotency and self-renewal capability in both embryonic and adult stem cells. Together with other transcription factors such as OCT4 and SOX2, which mediate self-renewal in embryonic stem cells, NANOG is downregulated by hypermethylation during differentiation in embryonic cells. These downstream regulatory pathways mediated by NANOG suggest that it regulates several biological processes involved in cancer development, including self-renewal, tumour cell proliferation, epithelial-mesenchymal transition and others. | Chambers I, Silva J, Colby D, Nichols J, Nijmeijer B, Robertson M, Vrana J, Jones K, Grotewold L, Smith A. Nanog safeguards pluripotency and mediates germline development. Nature. 2007 Dec 20;450(7173):1230-4. doi: 10.1038/nature06403. PMID: 18097409.  de Vicente JC, Rodríguez-Santamarta T, Rodrigo JP, Allonca E, Vallina A, Singhania A, Donate-Pérez Del Molino P, García-Pedrero JM. The Emerging Role of NANOG as an Early Cancer Risk Biomarker in Patients with Oral Potentially Malignant Disorders. J Clin Med. 2019 Sep 3;8(9):1376. doi: 10.3390/jcm8091376. PMID: 31484317; PMCID: PMC6780631. |
| Notch1 | Oncogenic  (pro-proliferative) | **Growth promoter**  The Notch pathway is an essential molecular signalling pathway that plays a critical role in the regulation of human carcinogenesis. It is predominantly implicated in cell proliferation, through p53-dependent transactivation repression and PI3K/Akt/PKB pathway activation, which in turn promotes tumor progression. | Nicolas M, Wolfer A, Raj K, Kummer JA, Mill P, van Noort M, Hui CC, Clevers H, Dotto GP, Radtke F. Notch1 functions as a tumor suppressor in mouse skin. Nat Genet. 2003 Mar;33(3):416-21. doi: 10.1038/ng1099. Epub 2003 Feb 18. PMID: 12590261.  Ding X, Zheng Y, Wang Z, Zhang W, Dong Y, Chen W, Li J, Chu W, Zhang W, Zhong Y, Mao L, Song X, Wu Y. Expression and oncogenic properties of membranous Notch1 in oral leukoplakia and oral squamous cell carcinoma. Oncol Rep. 2018 Jun;39(6):2584-2594. doi: 10.3892/or.2018.6335. Epub 2018 Mar 27. PMID: 29620248; PMCID: PMC5983926. |
| p16 | Protector  (growth suppressor) | **Cell cycle control**  It is a negative regulator of cell proliferation that inhibits progression through the cell cycle by binding to cyclin-dependent kinases (CDK) 4 or 6 and blocking the action of cyclin D. | Nobori, T., Miura, K., Wu, D. J., Lois, A., Takabayashi, K., & Carson, D. A. (1994). Deletions of the cyclin-dependent kinase-4 inhibitor gene in multiple human cancers. Nature, 368(6473), 753–756.  de Villalaín L, Álvarez-Teijeiro S, Rodríguez-Santamarta T, Fernández Del Valle Á, Allonca E, Rodrigo JP, de Vicente JC, García-Pedrero JM. Emerging Role of Decoy Receptor-2 as a Cancer Risk Predictor in Oral Potentially Malignant Disorders. Int J Mol Sci. 2023 Sep 21;24(18):14382. doi: 10.3390/ijms241814382. PMID: 37762685; PMCID: PMC10531848. |
| p21 | Protector  (growth suppressor) | **Cell cycle control**  p21 is also known as p21WAF1/Cip1. It is a cyclin-dependent kinase inhibitor that promotes cell cycle arrest in response to many stimuli. It is a negative regulator of cell proliferation by inhibiting the formation of cyclin E-CDK2 and cyclin D1-CDK4/6 complexes, or by inhibiting the activity of proteins directly involved in DNA synthesis. | Abbas T, Dutta A. p21 in cancer: intricate networks and multiple activities. Nat Rev Cancer. 2009 Jun;9(6):400-14. doi: 10.1038/nrc2657. PMID: 19440234; PMCID: PMC2722839.  Tanda N, Mori S, Saito K, Ikawa K, Sakamoto S. Expression of apoptotic signaling proteins in leukoplakia and oral lichen planus: quantitative and topographical studies. J Oral Pathol Med. 2000 Sep;29(8):385-93. doi: 10.1034/j.1600-0714.2000.290804.x. PMID: 10972347. |
| p27 | Protector  (growth suppressor) | **Cell cycle control**  p27, also known as p27kip1, is a member of the universal cyclin-dependent kinase inhibitor (CDKI) family. It is a cyclin-dependent kinase inhibitor involved in cell cycle regulation. p27 expression is regulated by cell contact inhibition and by specific growth factors such as transforming growth factor (TGF)-beta. | Liang J, Zubovitz J, Petrocelli T, Kotchetkov R, Connor MK, Han K, Lee JH, Ciarallo S, Catzavelos C, Beniston R, Franssen E, Slingerland JM. PKB/Akt phosphorylates p27, impairs nuclear import of p27 and opposes p27-mediated G1 arrest. Nat Med. 2002 Oct;8(10):1153-60. doi: 10.1038/nm761. Epub 2002 Sep 16. PMID: 12244302.  Lima JS, Correa L, Klingbeil MF, de Sousa SC. c-Jun, pc-Jun, and p27 are differently expressed in oral leukoplakias in smokers and never-smokers. Oral Surg Oral Med Oral Pathol Oral Radiol. 2016 Jan;121(1):73-80. doi: 10.1016/j.oooo.2015.09.003. Epub 2015 Sep 16. PMID: 26679360. |
| P53 | Protector  (growth suppressor) | **Cell cycle control, DNA repair and apoptosis**  It is a key tumour suppressor, known as the "guardian of the genome", stops the cell cycle and inhibits the proliferation of cells with damaged DNA, DNA repair and promotes apoptosis in cases of irreversible damage. | Lane, D. P. (1992). p53, guardian of the genome. Nature, 358(6381).  Ramos-García, P., González-Moles, M. Á., & Warnakulasuriya, S. (2022). Significance of p53 overexpression in the prediction of the malignant transformation risk of oral potentially malignant disorders: A systematic review and meta-analysis. Oral oncology, 126, 105734. https://doi.org/10.1016/j.oraloncology.2022.105734 |
| P63 | Protector  (growth suppressor) | **Cell cycle control, DNA repair and apoptosis**  Transmembranemember of the p53 gene family. It can mimic p53 activities by binding DNA, activating transcription, and inducing apoptosis. | Yang, A., & McKeon, F. (2000). P63 and P73: P53 mimics, menaces and more. Nature reviews. Molecular cell biology, 1(3), 199–207. https://doi.org/10.1038/35043127  Monteiro, L., do Amaral, B., Delgado, L., Garcês, F., Salazar, F., Pacheco, J. J., Lopes, C., & Warnakulasuriya, S. (2022). Podoplanin Expression Independently and Jointly with Oral Epithelial Dysplasia Grade Acts as a Potential Biomarker of Malignant Transformation in Oral Leukoplakia. Biomolecules, 12(5), 606. https://doi.org/10.3390/biom12050606 |
| deltaΔNp63 | Oncogenic  (anti-apoptotic) | **Anti-apoptotic**  Transmembrane member of the p53 gene family. Its ΔNp63 isoform is capable of blocking p53 or its canonical form, p63, activity by directly binding to them or competing for DNA sites. Alternatively, by binding to specific promoter elements, could block the transcription of critical genes, such as those involved in the apoptotic response. | Yang, A., & McKeon, F. (2000). P63 and P73: P53 mimics, menaces and more. Nature reviews. Molecular cell biology, 1(3), 199–207. <https://doi.org/10.1038/35043127>  Saintigny, P., El-Naggar, A. K., Papadimitrakopoulou, V., Ren, H., Fan, Y. H., Feng, L., Lee, J. J., Kim, E. S., Hong, W. K., Lippman, S. M., & Mao, L. (2009). DeltaNp63 overexpression, alone and in combination with other biomarkers, predicts the development of oral cancer in patients with leukoplakia. Clinical cancer research : an official journal of the American Association for Cancer Research, 15(19), 6284–6291. https://doi.org/10.1158/1078-0432.CCR-09-0498 |
| P73 | Protector  (growth suppressor) | **Cell cycle control, DNA repair and apoptosis**  Tumor suppressor from the P53 protein family, that plays a role in the transactivation of reporter genes that contain consensus p53-binding sites, as well as traditional p53 target genes such as p21 and its binding protein GADD45, Bax and MDM2, a negative regulator of p53. It is also capable of inducing apoptosis. | ost, C. A., Marin, M. C., & Jr, W. G. K. (1997). p73 is a human p53-related protein that can induce apoptosis.  Nature, 389(6647), 191-194.  Yang, A., & McKeon, F. (2000). P63 and P73: P53 mimics, menaces and more. Nature reviews. Molecular cell biology, 1(3), 199–207. https://doi.org/10.1038/35043127  Monteiro, L., do Amaral, B., Delgado, L., Garcês, F., Salazar, F., Pacheco, J. J., Lopes, C., & Warnakulasuriya, S. (2022). Podoplanin Expression Independently and Jointly with Oral Epithelial Dysplasia Grade Acts as a Potential Biomarker of Malignant Transformation in Oral Leukoplakia. Biomolecules, 12(5), 606. https://doi.org/10.3390/biom12050606 |
| PCNA | Oncogenic  (pro-proliferative) | **Indicator of cell cycle progression**  The PCNA (proliferating cell nuclear antigen) protein is used as a proliferation marker. PCNA plays an essential role in nucleic acid metabolism as a component of the replication and repair machinery. It acts as a processivity factor for DNA polymerase δ and ε, binding the polymerase catalytic unit to the DNA template for rapid and processive DNA synthesis. As a co-factor of DNA polymerase delta, it is required for DNA synthesis and cell cycle progression. | Kelman Z. PCNA: structure, functions and interactions. Oncogene. 1997 Feb 13;14(6):629-40. doi: 10.1038/sj.onc.1200886. PMID: 9038370.  Kanekawa A, Tsuji T, Mimura Y, Murakami T, Li X, Wen S, Shinozaki F. The estimation of proliferative activity by pcna and AgNORs in leukoplakia and squamous-cell carcinoma of the oral cavity. Oncol Rep. 1995 Sep;2(5):711-5. doi: 10.3892/or.2.5.711. PMID: 21597802. |
| PD-1 | Oncogenic  (anti-tumor arrest) | **Immunity response arrest**  PD-1 is a transmembrane receptor protein that can be found on natural killer T cells, B cells, activated monocytes and dendritic cells and can be induced in CD4 + and CD8 + T cells. It is involved in the development of immune tolerance as it prevents excessive immune cell activity that can lead to tissue destruction and autoimmunity. It can be used as an immune evasion mechanism by cancer cells. | Lenouvel, D., González-Moles, M. Á., Talbaoui, A., Ramos-García, P., González-Ruiz, L., Ruiz-Ávila, I., & Gil-Montoya, J. A. (2020). An update of knowledge on PD-L1 in head and neck cancers: Physiologic, prognostic and therapeutic perspectives. Oral diseases, 26(3), 511–526. <https://doi.org/10.1111/odi.13088>  Xu, S. B., Wang, M. Y., Shi, X. Z., Wang, Q., Yu, M., Zhang, W., Xu, X. H., & Liu, L. K. (2023). Influence of PD-1/PD-L1 on immune microenvironment in oral leukoplakia and oral squamous cell carcinoma. Oral diseases, 29(8), 3268–3277. https://doi.org/10.1111/odi.14332 |
| PDL-1 | Oncogenic  (anti-tumor arrest) | **Immunity response arrest**  Programmed death ligand 1 is a transmembrane protein of the B7 protein family that can be constitutively expressed or induced in the cell surface as ligands that bind to receptors on lymphocytes in order to regulate immune responses. It is involved in the development of immune tolerance as it prevents excessive immune cell activity that can lead to tissue destruction and autoimmunity. It can be used as an immune evasion mechanism by cancer cells. | Lenouvel, D., González-Moles, M. Á., Talbaoui, A., Ramos-García, P., González-Ruiz, L., Ruiz-Ávila, I., & Gil-Montoya, J. A. (2020). An update of knowledge on PD-L1 in head and neck cancers: Physiologic, prognostic and therapeutic perspectives. Oral diseases, 26(3), 511–526. <https://doi.org/10.1111/odi.13088>  Xu, S. B., Wang, M. Y., Shi, X. Z., Wang, Q., Yu, M., Zhang, W., Xu, X. H., & Liu, L. K. (2023). Influence of PD-1/PD-L1 on immune microenvironment in oral leukoplakia and oral squamous cell carcinoma. Oral diseases, 29(8), 3268–3277. https://doi.org/10.1111/odi.14332 |
| Podoplanin | Oncogenic  (pro-invasive) | **EMT induction**  It is a transmembrane receptor glycoprotein whose expression is induced by tumor promoters such as TPA, RAS and Src, activating cell motility through remodelling of actin in the cytoskeleton of tumor cells. | Astarita, J. L., Acton, S. E., & Turley, S. J. (2012). Podoplanin: emerging functions in development, the immune system, and cancer. Frontiers in immunology, 3, 283.  Monteiro, L., do Amaral, B., Delgado, L., Garcês, F., Salazar, F., Pacheco, J. J., Lopes, C., & Warnakulasuriya, S. (2022). Podoplanin Expression Independently and Jointly with Oral Epithelial Dysplasia Grade Acts as a Potential Biomarker of Malignant Transformation in Oral Leukoplakia. Biomolecules, 12(5), 606. https://doi.org/10.3390/biom12050606 |
| pRb | Protector  (growth suppressor) | **Growth suppressor**  pRb is a tumor suppressor protein that inhibits cell proliferation by controlling the cell cycle transition from G1 to S, inducing G1 arrest through the sequestration of E2F transcription factors. This prevents the activation of target genes involved in proliferation. | Dyson N. The regulation of E2F by pRB-family proteins. Genes Dev. 1998 Aug 1;12(15):2245-62. doi: 10.1101/gad.12.15.2245. PMID: 9694791.  Girod SC, Pfeiffer P, Ries J, Pape HD. Proliferative activity and loss of function of tumour suppressor genes as 'biomarkers' in diagnosis and prognosis of benign and preneoplastic oral lesions and oral squamous cell carcinoma. Br J Oral Maxillofac Surg. 1998 Aug;36(4):252-60. doi: 10.1016/s0266-4356(98)90708-2. PMID: 9762452. |
| PTHRP | Unspecified | Thyroid hormone-related protein (PTHrP), encoded by Parathyroid hormone-like hormone (PTHLH) gene, promotes osteolysis with potential oncogenic implications. This protein is secreted  by few normal tissues in an autocrine or paracrine manner, and is found to be upregulated in many tumors, including OSCC. | Kornberg, L. J., Villaret, D., Popp, M., Lui, L., McLaren, R., Brown, H., ... & McFadden, M. (2005). Gene expression profiling in squamous cell carcinoma of the oral cavity shows abnormalities in several signaling pathways. The Laryngoscope, 115(4), 690-698.  Lv, Z., Cong, R., Li, J., Cao, K., Bao, Q., Li, L., Yang, F., & Yuan, J. (2020). PTHLH Predicts the Prognosis of Patients with Oral Leukoplakia. OncoTargets and therapy, 13, 10013–10023. https://doi.org/10.2147/OTT.S261124 |
| PTMA  prothymosin alpha | Oncogenic  (pro-proliferative) | **Cell cycle progression**  Human prothymosin alpha (PTMA) is a protein that can be found in both the nucleus and the cytoplasm. In normal cells, the majority of PTMA is in the nucleus, where it regulates gene transcription, shapes DNA remodeling and promotes cell proliferation. | Samara, P., Ioannou, K., & Tsitsilonis, O. E. (2016). Prothymosin alpha and immune responses: are we close to potential clinical applications?. Vitamins and hormones, 102, 179-207.  Kaur, J., Matta, A., Kak, I., Srivastava, G., Assi, J., Leong, I., Witterick, I., Colgan, T. J., Macmillan, C., Siu, K. W., Walfish, P. G., & Ralhan, R. (2014). S100A7 overexpression is a predictive marker for high risk of malignant transformation in oral dysplasia. International journal of cancer, 134(6), 1379–1388. https://doi.org/10.1002/ijc.28473 |
| S100A7 | Unspecified | Also known as Psoriasin 1, is a protein of the family S100, characterized by its remarkable pleiotropism.  S100A7 secreted by tumor cells transforms surrounding stromal cells, making them secrete numerous paracrine and autocrine growth factors which, in turn, support the establishment of positive activation loops between all cell compartments. The most induced factors (VEGF, TNF-α, angiopoietin, IL6, granulocyte–macrophage colony-stimulating factor, GRO and MMPs) act as central mediators of tumor invasion, matrix remodeling, angiogenesis, inflammation and immune cell differentiation leading to a more aggressive milieu which, together with S100A7, will support tumor progression and facilitate metastatic spread. | Padilla, L., Dakhel, S., Adan, J. et al. S100A7: from mechanism to cancer therapy. Oncogene 36, 6749–6761 (2017). https://doi.org/10.1038/onc.2017.283  Kaur, J., Matta, A., Kak, I., Srivastava, G., Assi, J., Leong, I., Witterick, I., Colgan, T. J., Macmillan, C., Siu, K. W., Walfish, P. G., & Ralhan, R. (2014). S100A7 overexpression is a predictive marker for high risk of malignant transformation in oral dysplasia. International journal of cancer, 134(6), 1379–1388. https://doi.org/10.1002/ijc.28473 |
| DPC4 | Protector  (growth suppressor) | **Growth suppressor**  DPC4 plays a key role in switching TGF-β function in tumourigenesis. TGF-β subfamily signalling is initiated by TGF-β ligand activation and binding to the type II receptor (T-βR II). T-βR II then recruits and phosphorylates the type I receptor (T-βR I), which in turn phosphorylates its downstream targets. The phosphorylated targets then form a heterodimeric complex with DPC4 and translocates to the nucleus in order to recruit co-transcriptional factors to transactivate or repress target genes to lead the regulation of cellular proliferation. | Zhang Y, Feng X, We R, Derynck R. Receptor-associated Mad homologues synergize as effectors of the TGF-beta response. Nature. 1996 Sep 12;383(6596):168-72. doi: 10.1038/383168a0. PMID: 8774881.  Sakata J, Yoshida R, Matsuoka Y, Nagata M, Hirosue A, Kawahara K, Nakamura T, Nakamoto M, Hirayama M, Takahashi N, Nakashima H, Arita H, Ogi H, Hiraki A, Shinohara M, Nakayama H. Predictive value of the combination of SMAD4 expression and lymphocyte infiltration in malignant transformation of oral leukoplakia. Cancer Med. 2017 Apr;6(4):730-738. doi: 10.1002/cam4.1005. Epub 2017 Mar 3. PMID: 28256094; PMCID: PMC5387127. |
| Snail | Oncogenic  (pro-invasive) | **EMT induction**  Snail is a transcriptional repressor that plays a central role in epithelial-mesenchymal transition. It promotes the repression of the adhesion molecule E-cadherin to regulate (EMT), leading to the loss of cell-cell adhesion. As a result, epithelial cells lose their polarity and convert to a mesenchymal phenotype, acquiring tumorigenic and invasive/migratory properties. | Cano A, Pérez-Moreno MA, Rodrigo I, Locascio A, Blanco MJ, del Barrio MG, Portillo F, Nieto MA. The transcription factor snail controls epithelial-mesenchymal transitions by repressing E-cadherin expression. Nat Cell Biol. 2000 Feb;2(2):76-83. doi: 10.1038/35000025. PMID: 10655586.  Zhang X, Kim KY, Zheng Z, Kim HS, Cha IH, Yook JI. Snail and Axin2 expression predict the malignant transformation of oral leukoplakia. Oral Oncol. 2017 Oct;73:48-55. doi: 10.1016/j.oraloncology.2017.08.004. Epub 2017 Aug 12. PMID: 28939076. |
| SOX2 | Oncogenic  (pro-invasive) | **Cancer stem cell and EMT regulation**  The transcription factor SOX2 is essential for embryonic development and plays a key role in maintaining the pluripotency of embryonic stem cells (ESCs). It is also involved in the reprogramming of somatic cells into induced pluripotent stem cells (iPSCs). In particular, SOX2 and OCT4 work synergistically by binding to OCT4/SOX2 to regulate transcription. | Graham V, Khudyakov J, Ellis P, Pevny L. SOX2 functions to maintain neural progenitor identity. Neuron. 2003 Aug 28;39(5):749-65. doi: 10.1016/s0896-6273(03)00497-5. PMID: 12948443.  de Vicente JC, Donate-Pérez Del Molino P, Rodrigo JP, Allonca E, Hermida-Prado F, Granda-Díaz R, Rodríguez Santamarta T, García-Pedrero JM. SOX2 Expression Is an Independent Predictor of Oral Cancer Progression. J Clin Med. 2019 Oct 21;8(10):1744. doi: 10.3390/jcm8101744. PMID: 31640140; PMCID: PMC6832966. |
| Spindly | Protector  (pro-stability) | **DNA stability**  Spindly is a mitotic protein that plays a crucial role in regulating the cell cycle. It acts as a regulator of dynein in kinetochores and is involved in chromosome alignment and the spindle assembly checkpoint (SAC) signalling pathway. The SAC inhibits metaphase-to-anaphase transition until all chromosomes become bipolarly attached to the mitotic spindle and properly aligned at the metaphase plate. | Griffis ER, Stuurman N, Vale RD. Spindly, a novel protein essential for silencing the spindle assembly checkpoint, recruits dynein to the kinetochore. J Cell Biol. 2007 Jun 18;177(6):1005-15. doi: 10.1083/jcb.200702062. PMID: 17576797; PMCID: PMC2064361.  Silva PMA, Delgado ML, Ribeiro N, Florindo C, Tavares ÁA, Ribeiro D, Lopes C, do Amaral B, Bousbaa H, Monteiro LS. Spindly and Bub3 expression in oral cancer: Prognostic and therapeutic implications. Oral Dis. 2019 Jul;25(5):1291-1301. doi: 10.1111/odi.13089. Epub 2019 Apr 4. PMID: 30866167. |
| TIPE2 | Oncogenic  (anti-tumor arrest) | **Immunity response arrest**  TIPE2 (TNF-α–induced protein 8–like 2) is a crucial negative regulator of both innate and adaptive immune responses. It interacts with TGF-β-activated kinase 1 (TAK1), a key molecule in inflammatory and immune signaling pathways, thereby acting as a potent inhibitor of TAK1. TIPE2 is selectively expressed in the immune system, where it regulates TLR (Toll-like receptor) and TCR (T-cell receptor) functions, ensuring that the immune system does not become hyper-responsive. This regulation is essential for maintaining immune homeostasis and preventing excessive immune responses. | Sun H, Gong S, Carmody RJ, Hilliard A, Li L, Sun J, Kong L, Xu L, Hilliard B, Hu S, Shen H, Yang X, Chen YH. TIPE2, a negative regulator of innate and adaptive immunity that maintains immune homeostasis. Cell. 2008 May 2;133(3):415-26. doi: 10.1016/j.cell.2008.03.026. PMID: 18455983; PMCID: PMC2398615.  Chen H, Pei M, Shin SW, Moon S, Nam W, Zheng Z. Implication of TIPE2 Expression on the Malignant Transformation of Oral Leukoplakia. Anticancer Res. 2023 Nov;43(11):4937-4946. doi: 10.21873/anticanres.16691. PMID: 37909982. |
| yH2AFX | Protector  (pro-stability) | **DNA damage repair**  H2A histone family member X (H2AFX) is a key DNA double-strand repair (DDR) component.Within minutes of DNA damage, H2AFX is phosphorylated at its carboxyl terminus to form γH2AFX at double-strand breaks (DSBs) sites. Over the following 30 minutes, a significant number of γH2AX molecules are formed in the surrounding chromatin, creating a concentration point for proteins involved in DNA repair and chromatin remodelling.The formation of these proteins indicates that H2AFX plays a vital role in the initial stages of DDR. H2AFX protein is phosphorylated by ATM, and the level of γH2AFX is positively associated with the degree of DNA damage. | Rogakou EP, Boon C, Redon C, Bonner WM. Megabase chromatin domains involved in DNA double-strand breaks in vivo. J Cell Biol. 1999 Sep 6;146(5):905-16. doi: 10.1083/jcb.146.5.905. PMID: 10477747; PMCID: PMC2169482.  Zhu M, Liu W, Shi L, Xiao X, Wu W, Wu L, Zhou Z. Expression of DNA doublestrand repair proteins in oral leukoplakia and the risk of malignant transformation. Oncol Lett. 2018 Jun;15(6):9827-9835. doi: 10.3892/ol.2018.8574. Epub 2018 Apr 25. PMID: 29928356; PMCID: PMC6004653. |
| AgNOR | Oncogenic (pro-proliferative) | **Biogenesis of ribosomal RNA**  AgNORs are closely related to nucleolar organiser regions (NORs), which form loops of ribosomal DNA (rDNA) that transcribe ribosomal RNA (rRNA). It acts as the structure of central importance in the transcription of nucleic acid protein. During interphase, NORs are located in the fibrillar portion of the nucleus, which is associated with acidic proteins that stain selectively with silver methods. An increase in AgNOR proteins may be due to an increased demand for ribosomal biogenesis, reflecting high metabolic activity. | Derenzini M. The AgNORs. Micron. 2000 Apr;31(2):117-20. doi: 10.1016/s0968-4328(99)00067-0. PMID: 10588056.  Kanekawa A, Tsuji T, Mimura Y, Murakami T, Li X, Wen S, Shinozaki F. The estimation of proliferative activity by pcna and AgNORs in leukoplakia and squamous-cell carcinoma of the oral cavity. Oncol Rep. 1995 Sep;2(5):711-5. doi: 10.3892/or.2.5.711. PMID: 21597802. |

**7. List S1. List of included studies (n = 60)**

Benchekroun, M. T., Saintigny, P., Thomas, S. M., El-Naggar, A. K., Papadimitrakopoulou, V., Ren, H., … Mao, L. (2010). Epidermal growth factor receptor expression and gene copy number in the risk of oral cancer. *Cancer Prevention Research*, *3*(7), 800–809. https://doi.org/10.1158/1940-6207.CAPR-09-0163

Cao, W., Younis, R. H., Li, J., Chen, H., Xia, R., Mao, L., … Ren, H. (2011). EZH2 promotes malignant phenotypes and is a predictor of oral cancer development in patients with oral leukoplakia. *Cancer Prevention Research*, *4*(11), 1816–1824. https://doi.org/10.1158/1940-6207.CAPR-11-0130

Chen, H., Meiling, P. E. I., Shin, S. W. O. O., Moon, S., Woong, N. A. M., & Zheng, Z. (2023). Implication of TIPE2 Expression on the Malignant Transformation of Oral Leukoplakia. *Anticancer Research*, *43*(11), 4937–4946. https://doi.org/10.21873/anticanres.16691

Cruz, I. B., Snijders, P. J. F., Meijer, C. J., Braakhuis, B. J., Snow, G. B., Walboomers, J. M., & Van Der Waal, I. (1998). P53 Expression Above the Basal Cell Layer in Oral Mucosa Is an Early Event of Malignant Transformation and Has Predictive Value for Developing Oral Squamous Cell Carcinoma. *Journal of Pathology*, *184*(4), 360–368. https://doi.org/10.1002/(SICI)1096-9896(199804)184:4<360::AID-PATH1263>3.0.CO;2-H

D’Souza, B., Nayak, R., & Kotrashetti, V. S. (2018). Immunohistochemical Expression of Podoplanin in Clinical Variants of Oral Leukoplakia and Its Correlation with Epithelial Dysplasia. *Applied Immunohistochemistry and Molecular Morphology*, *26*(2), 132–139. https://doi.org/10.1097/PAI.0000000000000383

de Vicente, Juan C., Del Molino, P. D. P., Rodrigo, J. P., Allonca, E., Hermida-Prado, F., Granda-Díaz, R., … García-Pedrero, J. M. (2019). SOX2 expression is an independent predictor of oral cancer progression. *Journal of Clinical Medicine*, *8*(10). https://doi.org/10.3390/jcm8101744

de Vicente, Juan C., Rodríguez-Santamarta, T., Rodrigo, J. P., Allonca, E., Vallina, A., Singhania, A., … García-Pedrero, J. M. (2019). The emerging role of NANOG as an early cancer risk biomarker in patients with oral potentially malignant disorders. *Journal of Clinical Medicine*, *8*(9). https://doi.org/10.3390/jcm8091376

de Vicente, Juan Carlos, Rodrigo, J. P., Rodriguez-Santamarta, T., Lequerica-Fernández, P., Allonca, E., & García-Pedrero, J. M. (2013). Podoplanin expression in oral leukoplakia: Tumorigenic role. *Oral Oncology*, *49*(6), 598–603. https://doi.org/10.1016/j.oraloncology.2013.02.008

de Villalaín, L., Álvarez-Teijeiro, S., Rodríguez-Santamarta, T., Fernández del Valle, Á., Allonca, E., Rodrigo, J. P., … García-Pedrero, J. M. (2023). Emerging Role of Decoy Receptor-2 as a Cancer Risk Predictor in Oral Potentially Malignant Disorders. *International Journal of Molecular Sciences*, *24*(18). https://doi.org/10.3390/ijms241814382

Ding, X., Zheng, Y., Wang, Z., Zhang, W., Dong, Y., Chen, W., … Wu, Y. (2018). Expression and oncogenic properties of membranous Notch1 in oral leukoplakia and oral squamous cell carcinoma. *Oncology Reports*, *39*(6), 2584–2594. https://doi.org/10.3892/or.2018.6335

Fernández-Valle, Á., Rodrigo, J. P., García-Pedrero, J. M., Rodríguez-Santamarta, T., Allonca, E., Lequerica-Fernández, P., & de Vicente, J. C. (2016). Expression of the voltage-gated potassium channel Kv3.4 in oral leucoplakias and oral squamous cell carcinomas. *Histopathology*, *69*(1), 91–98. https://doi.org/10.1111/his.12917

Fernández–Valle, Á., Rodrigo, J. P., Rodríguez–Santamarta, T., Villaronga, M. Á., Álvarez–Teijeiro, S., García–Pedrero, J. M., … de Vicente, J. C. (2016). HERG1 potassium channel expression in potentially malignant disorders of the oral mucosa and prognostic relevance in oral squamous cell carcinoma. *Head and Neck*, *38*(11), 1672–1678. https://doi.org/10.1002/hed.24493

Gissi, D. B., Gabusi, A., Servidio, D., Cervellati, F., & Montebugnoli, L. (2015). Predictive Role of p53 Protein as a Single Marker or Associated with ki67 Antigen in Oral Leukoplakia: A Retrospective Longitudinal Study. *The Open Dentistry Journal*, *9*(1), 41–45. https://doi.org/10.2174/1874210601509010041

Graveland, A. P., Bremmer, J. F., De Maaker, M., Brink, A., Cobussen, P., Zwart, M., … Brakenhoff, R. H. (2013). Molecular screening of oral precancer. *Oral Oncology*, *49*(12), 1129–1135. https://doi.org/10.1016/j.oraloncology.2013.09.005

Habiba, U., Hida, K., Kitamura, T., Matsuda, A. Y., Higashino, F., Ito, Y. M., … Shindoh, M. (2017). ALDH1 and podoplanin expression patterns predict the risk of malignant transformation in oral leukoplakia. *Oncology Letters*, *13*(1), 321–328. https://doi.org/10.3892/ol.2016.5379

Kanekawa, A., Tsuji, T., Mimura, Y., Murakami, T., Li, X. W., Wen, S., & Shinozaki, F. (1995). The estimation of proliferative activity by PCNA and AgNORs in leukoplakia and squamous cell carcinoma of the oral cavity. *Oncology Reports*, *2*(5), 711–715. https://doi.org/10.3892/or.2.5.711

Kaur, J., Matta, A., Kak, I., Srivastava, G., Assi, J., Leong, I., … Ralhan, R. (2014). S100A7 overexpression is a predictive marker for high risk of malignant transformation in oral dysplasia. *International Journal of Cancer*, *134*(6), 1379–1388. https://doi.org/10.1002/ijc.28473

Kawaguchi, H., El-Naggar, A. K., Papadimitrakopoulou, V., Ren, H., Fan, Y. H., Feng, L., … Mao, L. (2008). Podoplanin: A novel marker for oral cancer risk in patients with oral premalignancy. *Journal of Clinical Oncology*, *26*(3), 354–360. https://doi.org/10.1200/JCO.2007.13.4072

Kikegawa, A. (2001). Immunohistochemical analysis of the p53 tumor suppressor gene product in oral leukoplakia. *Kōkūbyō Gakkai Zasshi. The Journal of the Stomatological Society, Japan*, *68*(1), 51–59. https://doi.org/10.5357/koubyou.68.51

Kreppel, M., Kreppel, B., Drebber, U., Wedemayer, I., Rothamel, D., Zöller, J. E., & Scheer, M. (2012). Podoplanin expression in oral leukoplakia: Prognostic value and clinicopathological implications. *Oral Diseases*, *18*(7), 692–699. https://doi.org/10.1111/j.1601-0825.2012.01927.x

Lima, J. S., Correa, L., Guarizo Klingbeil, M. F., & Orsini Machado De Sousa, S. C. (2016). C-Jun, pc-Jun, and p27 are differently expressed in oral leukoplakias in smokers and never-smokers. *Oral Surgery, Oral Medicine, Oral Pathology and Oral Radiology*, *121*(1), 73–80. https://doi.org/10.1016/j.oooo.2015.09.003

Liu, W., Feng, J. Q., Shen, X. M., Wang, H. Y., Liu, Y., & Zhou, Z. T. (2012). Two stem cell markers, ATP-binding cassette, G2 subfamily (ABCG2) and BMI-1, predict the transformation of oral leukoplakia to cancer: A long-term follow-up study. *Cancer*, *118*(6), 1693–1700. https://doi.org/10.1002/cncr.26483

Liu, W., Wu, L., Shen, X. M., Shi, L. J., Zhang, C. P., Xu, L. Q., & Zhou, Z. T. (2012). Expression patterns of cancer stem cell markers ALDH1 and CD133 correlate with a high risk of malignant transformation of oral leukoplakia. *International Journal of Cancer*, *132*(4), 868–874. https://doi.org/10.1002/ijc.27720

Lv, Z., Cong, R., Li, J., Cao, K., Bao, Q., Li, L., … Yuan, J. (2020). Pthlh predicts the prognosis of patients with oral leukoplakia. *OncoTargets and Therapy*, *13*, 10013–10023. https://doi.org/10.2147/OTT.S261124

Mao, T., Xiong, H., Hu, X., Hu, Y., Wang, C., Yang, L., … Su, T. (2020). DEC1: A potential biomarker of malignant transformation in oral leukoplakia. *Brazilian Oral Research*, *34*, e052. https://doi.org/10.1590/1807-3107BOR-2020.VOL34.0052

Mariz, B. A. L. A., Sales de Sá, R., Araújo, A. L. D., Fernandes, C. I. R., Mariano, F. V., Santos-Silva, A. R., … Jorge, J. (2023). FGFR1 is an important prognostic factor in oral leukoplakia and tongue squamous cell carcinoma. *Journal of Oral Pathology and Medicine*, *52*(2), 119–126. https://doi.org/10.1111/jop.13398

Matsubara, R., Kawano, S., Kiyosue, T., Goto, Y., Hirano, M., Jinno, T., … Nakamura, S. (2011). Increased ΔNp63 expression is predictive of malignant transformation in oral epithelial dysplasia and poor prognosis in oral squamous cell carcinoma. *International Journal of Oncology*, *39*(6), 1391–1399. https://doi.org/10.3892/ijo.2011.1151

Matthias, C., Mack, B., Berghaus, A., & Gires, O. (2008). Keratin 8 expression in head and neck epithelia. *BMC Cancer*, *8*, 267. https://doi.org/10.1186/1471-2407-8-267

Mondal, K., Mandal, R., & Sarkar, B. C. (2020). Importance of Ki-67 Labeling in Oral Leukoplakia with Features of Dysplasia and Carcinomatous Transformation: An Observational Study over 4 Years. *South Asian Journal of Cancer*, *9*(2), 99–104. https://doi.org/10.1055/s-0040-1721212

Monteiro, L., Do Amaral, B., Delgado, L., Garcês, F., Salazar, F., Pacheco, J. J., … Warnakulasuriya, S. (2022). Podoplanin Expression Independently and Jointly with Oral Epithelial Dysplasia Grade Acts as a Potential Biomarker of Malignant Transformation in Oral Leukoplakia. *Biomolecules*, *12*(5). https://doi.org/10.3390/biom12050606

Monteiro, L., Silva, P., Delgado, L., Amaral, B., Garcês, F., Salazar, F., … Warnakulasuriya, S. (2021). Expression of spindle assembly checkpoint proteins bubr1 and mad2 expression as potential biomarkers of malignant transformation of oral leukoplakia: An observational cohort study. *Medicina Oral Patologia Oral y Cirugia Bucal*, *26*(6), e719-E728. https://doi.org/10.4317/medoral.24511

Nayak, S., Goel, M. M., Makker, A., Bhatia, V., Chandra, S., Kumar, S., & Agarwal, S. P. (2015). Fibroblast growth factor (FGF-2) and its receptors FGFR-2 and FGFR-3 may be putative biomarkers of malignant transformation of potentially malignant oral lesions into oral squamous cell carcinoma. *PLoS ONE*, *10*(10), e0138801. https://doi.org/10.1371/journal.pone.0138801

Nguyen, C. T. K., Okamura, T., Morita, K. I., Yamaguchi, S., Harada, H., Miki, Y., … Sakamoto, K. (2017). LAMC2 is a predictive marker for the malignant progression of leukoplakia. *Journal of Oral Pathology and Medicine*, *46*(3), 223–231. https://doi.org/10.1111/jop.12485

Ögmundsdóttir, H. M., Björnsson, J., & Holbrook, W. P. (2009). Role of TP53 in the progression of pre-malignant and malignant oral mucosal lesions. A follow-up study of 144 patients. *Journal of Oral Pathology and Medicine*, *38*(7), 565–571. https://doi.org/10.1111/j.1600-0714.2009.00766.x

Oliver, R. J., MacDonald, D. G., & Felix, D. H. (2000). Aspects of cell proliferation in oral epithelial dysplastic lesions. *Journal of Oral Pathology and Medicine*, *29*(2), 49–55. https://doi.org/10.1034/j.1600-0714.2000.290201.x

Papadimitrakopoulou, V., Izzo, J., Lippman, S. M., Lee, J. S., Fan, Y. H., Clayman, G., … Mao, L. (1997). Frequent inactivation of p16(INK4α) in oral premalignant lesions. *Oncogene*, *14*(15), 1799–1803. https://doi.org/10.1038/sj.onc.1201010

Rich, A. M., Kerdpon, D., & Reade, P. C. (1999). P53 Expression in Oral Precancer and Cancer. *Australian Dental Journal*, *44*(2), 103–105. https://doi.org/10.1111/j.1834-7819.1999.tb00209.x

Ries, J., Agaimy, A., Vairaktaris, E., Gorecki, P., Neukam, F. W., Straßburg, L. H., & Nkenke, E. (2012). Detection of MAGE-A expression predicts malignant transformation of oral leukoplakia. *Cancer Investigation*, *30*(7), 495–502. https://doi.org/10.3109/07357907.2012.691191

Ries, J., Vairaktaris, E., Agaimy, A., Bechtold, M., Gorecki, P., Neukam, F. W., & Nkenke, E. (2013). The relevance of EGFR overexpression for the prediction of the malignant transformation of oral leukoplakia. *Oncology Reports*, *30*(3), 1149–1156. https://doi.org/10.3892/or.2013.2545

Saintigny, P., El-Naggar, A. K., Papadimitrakopoulou, V., Ren, H., Fan, Y. H., Feng, L., … Mao, L. (2009). ΔNp63 overexpression, alone and in combination with other biomarkers, predicts the development of oral cancer in patients with leukoplakia. *Clinical Cancer Research*, *15*(19), 6284–6291. https://doi.org/10.1158/1078-0432.CCR-09-0498

Saintigny, P., William, W. N., Foy, J. P., Papadimitrakopoulou, V., Lang, W., Zhang, L., … Lippman, S. M. (2018). Met Receptor Tyrosine Kinase and Chemoprevention of Oral Cancer. *Journal of the National Cancer Institute*, *110*(3), 250–257. https://doi.org/10.1093/jnci/djx186

Sakata, J., Yoshida, R., Matsuoka, Y., Kawahara, K., Arita, H., Nakashima, H., … Nakayama, H. (2020). FOXP3 lymphocyte status may predict the risk of malignant transformation in oral leukoplakia. *Journal of Oral and Maxillofacial Surgery, Medicine, and Pathology*, *32*(1), 33–39. https://doi.org/10.1016/j.ajoms.2019.06.005

Sakata, J., Yoshida, R., Matsuoka, Y., Nagata, M., Hirosue, A., Kawahara, K., … Nakayama, H. (2017). Predictive value of the combination of SMAD4 expression and lymphocyte infiltration in malignant transformation of oral leukoplakia. *Cancer Medicine*, *6*(4), 730–738. https://doi.org/10.1002/cam4.1005

Santos García, A., Abad Hernández, M. M., Fonseca Sánchez, E., Cruz Hernández, J. J., & Bullón Sopelana, A. (2005). Proteic expressión of p53 and cellular proliferation in oral leukoplakias. *Medicina Oral, Patologia Oral y Cirugia Bucal*, *10*(1), 1–8. Retrieved from https://pubmed.ncbi.nlm.nih.gov/15627901/

Shigeoka, M., Koma, Y. ichiro, Kodama, T., Nishio, M., Akashi, M., & Yokozaki, H. (2020). Intraepithelial CD163+ macrophages in tongue leukoplakia biopsy: A promising tool for cancer screening. *Oral Diseases*, *26*(3), 527–536. https://doi.org/10.1111/odi.13269

Soni, S., Kaur, J., Kumar, A., Chakravarti, N., Mathur, M., Bahadur, S., … Ralhan, R. (2005). Alterations of Rb pathway components are frequent events in patients with oral epithelial dysplasia and predict clinical outcome in patients with squamous cell carcinoma. *Oncology*, *68*(4–6), 314–325. https://doi.org/10.1159/000086970

Sulkowska, M., Famulski, W., Chyczewski, L., & Sulkowski, S. (2001). Evaluation of p53 and Bcl-2 oncoprotein expression in precancerous lesions of the oral cavity. *Neoplasma*, *48*(2), 94–98. Retrieved from https://pubmed.ncbi.nlm.nih.gov/11478700/

Sundberg, J., Korytowska, M., Miranda Burgos, P., Blomgren, J., Blomstrand, L., De Lara, S., … Hasséus, B. (2019). Combined testing of p16 tumour-suppressor protein and human papillomavirus in patients with oral leukoplakia and oral squamous cell carcinoma. *Anticancer Research*, *39*(3), 1293–1300. https://doi.org/10.21873/anticanres.13241

Tanda, N., Mori, S., Saito, K., Ikawa, K., & Sakamoto, S. (2000). Expression of apoptotic signaling proteins in leukoplakia and oral lichen planus: Quantitative and topographical studies. *Journal of Oral Pathology and Medicine*, *29*(8), 385–393. https://doi.org/10.1034/j.1600-0714.2000.290804.x

Tarle, M., Müller, D., Raguž, M., & Lukšić, I. (2022). Significance of nuclear EGFR and ABCG2 expression in malignant transformation of oral potentially malignant disorders. *Head and Neck*, *44*(12), 2668–2677. https://doi.org/10.1002/hed.27174

Weber, M., Wehrhan, F., Baran, C., Agaimy, A., Büttner-Herold, M., Öztürk, H., … Ries, J. (2020). Malignant transformation of oral leukoplakia is associated with macrophage polarization. *Journal of Translational Medicine*, *18*(1), 11. https://doi.org/10.1186/s12967-019-02191-0

Wils, L. J., Poell, J. B., Peferoen, L. A. N., Evren, I., Brouns, E. R., de Visscher, J. G. A. M., … Bloemena, E. (2023). The role of differentiated dysplasia in the prediction of malignant transformation of oral leukoplakia. *Journal of Oral Pathology and Medicine*, *52*(10), 930–938. https://doi.org/10.1111/jop.13483

Wu, W., Wang, Z., & Zhou, Z. (2019). Role of the human papillomavirus in malignant transformation of oral leukoplakia distinct from oropharyngeal squamous cell carcinoma: A study of 76 patients with internal-control specimens. *Oral Surgery, Oral Medicine, Oral Pathology and Oral Radiology*, *128*(3), 273–279. https://doi.org/10.1016/j.oooo.2019.01.004

Wu, X., Wang, R., Gong, Z., Zhu, J., & Zhu, S. (2022). The expression and clinical significance of Bmi-1 gene in oral leukoplakia with different prognosis. *National Medical Journal of China*, *102*(11), 787–794. https://doi.org/10.3760/cma.j.cn112137-20211014-02277

Xia, R. H., Song, X. M., Wang, X. J., Li, J., & Mao, L. (2013). The Combination of SMAD4 Expression and Histological Grade of Dysplasia Is a Better Predictor for the Malignant Transformation of Oral Leukoplakia. *PLoS ONE*, *8*(6), e66794. https://doi.org/10.1371/journal.pone.0066794

Xu, S. B., Wang, M. Y., Shi, X. Z., Wang, Q., Yu, M., Zhang, W., … Liu, L. K. (2023). Influence of PD-1/PD-L1 on immune microenvironment in oral leukoplakia and oral squamous cell carcinoma. *Oral Diseases*, *29*(8), 3268–3277. https://doi.org/10.1111/odi.14332

Yagyuu, T., Funayama, N., Imada, M., & Kirita, T. (2021). Effect of smoking status and programmed death-ligand 1 expression on the microenvironment and malignant transformation of oral leukoplakia: A retrospective cohort study. *PLoS ONE*, *16*(4 April), e0250359. https://doi.org/10.1371/journal.pone.0250359

Zhang, X., Kim, K. Y., Zheng, Z., Bazarsad, S., & Kim, J. (2017). Nomogram for risk prediction of malignant transformation in oral leukoplakia patients using combined biomarkers. *Oral Oncology*, *72*, 132–139. https://doi.org/10.1016/j.oraloncology.2017.07.015

Zhang, X., Kim, K. Y., Zheng, Z., Kim, H. S., Cha, I. H., & Yook, J. I. (2017). Snail and Axin2 expression predict the malignant transformation of oral leukoplakia. *Oral Oncology*, *73*, 48–55. https://doi.org/10.1016/j.oraloncology.2017.08.004

Zhu, M., Liu, W., Shi, L., Xiao, X., Wu, W., Wu, L., & Zhou, Z. (2018). Expression of DNA doublestrand repair proteins in oral leukoplakia and the risk of malignant transformation. *Oncology Letters*, *15*(6), 9827–9835. https://doi.org/10.3892/ol.2018.8574

**8. List S2. List of excluded studies (with reasons)**

**Not immunohistochemistry (n = 599):**

Abdulrahim, M. H., McManus, B. A., Flint, S. R., & Coleman, D. C. (2013). Genotyping Candida albicans from Candida Leukoplakia and Non-Candida Leukoplakia Shows No Enrichment of Multilocus Sequence Typing Clades but Enrichment of ABC Genotype C in Candida Leukoplakia. *PLoS ONE*, *8*(9), e73738. https://doi.org/10.1371/journal.pone.0073738

Abe, M., Yamashita, S., Mori, Y., Abe, T., Saijo, H., Hoshi, K., … Takato, T. (2016). High-risk oral leukoplakia is associated with aberrant promoter methylation of multiple genes. *BMC Cancer*, *16*(1), 350. https://doi.org/10.1186/s12885-016-2371-5

Adeoye, J., Koohi-Moghadam, M., Lo, A. W. I., Tsang, R. K. Y., Chow, V. L. Y., Zheng, L. W., … Su, Y. X. (2021). Deep learning predicts the malignant-transformation-free survival of oral potentially malignant disorders. *Cancers*, *13*(23). https://doi.org/10.3390/cancers13236054

Adeoye, J., & Su, Y. X. (2024). Validity of nomograms for predicting cancer risk in oral leukoplakia and oral lichen planus. *Oral Diseases*, *30*(5), 3039–3051. https://doi.org/10.1111/odi.14811

Airoldi, M., Piantino, P., Pacchioni, D., Mastromatteo, V., Pedani, F., & Gandolfo, S. (1986). Gastrointestinal cancer-associated antigen (GICA) in oral carcinoma. *Oral Surgery, Oral Medicine, Oral Pathology*, *61*(3), 263–267. https://doi.org/10.1016/0030-4220(86)90372-5

Ajona, D., Pajares, M. J., Chiara, M. D., Rodrigo, J. P., Jantus-Lewintre, E., Camps, C., … Pio, R. (2015). Complement activation product C4d in oral and oropharyngeal squamous cell carcinoma. *Oral Diseases*, *21*(7), 899–904. https://doi.org/10.1111/odi.12363

Al-Tayar, B. A., Tin-Oo, M. M., Sinor, M. Z., & Alakhali, M. S. (2016). Association between Shammah use and oral leukoplakia-like lesions among adult males in Dawan Valley, Yemen. *Asian Pacific Journal of Cancer Prevention*, *16*(18), 8365–8370. https://doi.org/10.7314/APJCP.2015.16.18.8365

Al Jabab, M. A., Aljbab, A. A., & Patil, S. R. (2015). Evaluation of Oral Changes Among Tobacco Users of Aljouf Province, Saudi Arabia. *Journal of Clinical and Diagnostic Research*, *9*(5), ZC58–ZC61. https://doi.org/10.7860/JCDR/2015/13683.5950

Anantharaman, D., Samant, T. A., Sen, S., & Mahimkar, M. B. (2011). Polymorphisms in tobacco metabolism and DNA repair genes modulate oral precancer and cancer risk. *Oral Oncology*, *47*(9), 866–872. https://doi.org/10.1016/j.oraloncology.2011.06.015

Anastasov, K., & Shpatov, K. (1969). Z etiologichnata vruzka mezhdu levkoplakiiata, luesa i raka na ustnata kukhina [Etiological relationship between leukoplakia, lues and cancer of the oral cavity]. Nauchni trudove. Sofia. Nauchno-izsledovatelski stomatologichen institut, 12, 55–60.

Anil, S., Beena, V. T., Nair, R. G., & Vijayakumar, T. (1995). Evaluation of serum β2-microglobulin in premalignant and malignant lesions of the oral cavity. *Oral Surgery, Oral Medicine, Oral Pathology, Oral Radiology And*, *79*(6), 750–752. https://doi.org/10.1016/S1079-2104(05)80311-7

Ankita, K., Shwetha, V., Vanitha, S., Reddy Sujatha, S., Nagaraju, R., & Tupakula Pavan, K. (2019). Assessment of salivary endothelin-1 in patients with leukoplakia, submucous fibrosis, oral cancer and healthy individuals – a comparative study. *Journal of Stomatology, Oral and Maxillofacial Surgery*, *120*(4), 326–331. https://doi.org/10.1016/j.jormas.2019.02.024

Asokan, G. S., Jeelani, S., & Gnanasundaram, N. (2014). Promoter hypermethylation profile of tumour suppressor genes in oral leukoplakia and oral squamous cell carcinoma. *Journal of Clinical and Diagnostic Research*, *8*(10), ZC09-ZC12. https://doi.org/10.7860/JCDR/2014/9251.4949

Awan, K. H., Morgan, P. R., & Warnakulasuriya, S. (2015). Assessing the accuracy of autofluorescence, chemiluminescence and toluidine blue as diagnostic tools for oral potentially malignant disorders—a clinicopathological evaluation. *Clinical Oral Investigations*, *19*(9), 2267–2272. https://doi.org/10.1007/s00784-015-1457-9

Azab, N. A., Zahran, F. M., Amin, A. A. W., & Rady, N. H. (2021). Dna integrity in diagnosis of premalignant lesions. *Medicina Oral Patologia Oral y Cirugia Bucal*, *26*(4), e445–e450. https://doi.org/10.4317/medoral.24287

Aziz, N. Z., Arathi, K., Ganesh Prasad, B., Desai, D., Shetty, S. J., & Shahid, M. (2018). Evaluation of magnesium levels in blood and saliva of oral squamous cell carcinoma and potentially malignant disorders by xylidyl blue method. *Journal of Oral and Maxillofacial Pathology*, *22*(1), 147–148. https://doi.org/10.4103/jomfp.JOMFP_34_17

Babiuch, K., Bednarczyk, A., Gawlik, K., Pawlica-Gosiewska, D., Kęsek, B., Darczuk, D., … Kaczmarzyk, T. (2019). Evaluation of enzymatic and non-enzymatic antioxidant status and biomarkers of oxidative stress in saliva of patients with oral squamous cell carcinoma and oral leukoplakia: a pilot study. *Acta Odontologica Scandinavica*, *77*(6), 408–418. https://doi.org/10.1080/00016357.2019.1578409

Bagan, J. V., Mata-Roig, M., Cortio-Gimeno, J., Murillo-Cortes, J., Hens-Aumente, E., Poveda-Roda, R., & Bagan, L. (2012). Epidermal growth factor receptor copy number in potentially malignant oral disorders and oral squamous cell carcinoma: A short communication and preliminary study. *Journal of Oral Pathology and Medicine*, *41*(9), 662–666. https://doi.org/10.1111/j.1600-0714.2012.01137.x

Balaram, P., Pillai, M. R., & Abraham, T. (1987). Immunology of premalignant and malignant conditions of the oral cavity. II. Circulating immune complexes. *Journal of Oral Pathology & Medicine*, *16*(8), 389–391. https://doi.org/10.1111/j.1600-0714.1987.tb02070.x

Banerjee, S., Mukherjee, S., Mitra, S., & Singhal, P. (2019). Comparative evaluation of mitochondrial antioxidants in oral potentially malignant disorders. *Kurume Medical Journal*, *66*(1), 15–27. https://doi.org/10.2739/kurumemedj.MS661009

Banoczy, J. (1977). Follow-up studies in oral leukoplakia. *Journal of Maxillofacial Surgery*, *5*(1), 69–75. Retrieved from https://pubmed.ncbi.nlm.nih.gov/5308702/

Bánóczy, J., & Sugár, L. (1972). Longitudinal studies in oral leukoplakias. *Journal of Oral Pathology & Medicine*, *1*(6), 265–272. https://doi.org/10.1111/j.1600-0714.1972.tb01665.x

Bánóczy, J., & Sugár, L. (1975). Progressive and regressive changes in Hungarian oral leukoplakias in the course of longitudinal studies. *Community Dentistry and Oral Epidemiology*, *3*(4), 194–197. https://doi.org/10.1111/j.1600-0528.1975.tb00307.x

Bánóczy, Jolán. (1977). Follow-up studies in oral leukoplakia. *Journal of Maxillofacial Surgery*, *5*(C), 69–75. https://doi.org/10.1016/S0301-0503(77)80079-9

Bánóczy, Jolán, & Csiba, Á. (1972). Comparative study of the clinical picture and histopathologic structure of oral leukoplakia. *Cancer*, *29*(5), 1230–1234. https://doi.org/10.1002/1097-0142(197205)29:5<1230::AID-CNCR2820290515>3.0.CO;2-3

Bánóczy, Jolán, & Csiba, Á. (1976). Occurrence of epithelial dysplasia in oral leukoplakia. Analysis and follow-up study of 12 cases. *Oral Surgery, Oral Medicine, Oral Pathology*, *42*(6), 766–774. https://doi.org/10.1016/0030-4220(76)90099-2

Bánóczy, Jolán, Juhász, J., & Albrecht, M. (1980). Ultrastructure of different clinical forms of oral leukoplakia. *Journal of Oral Pathology & Medicine*, *9*(1), 41–53. https://doi.org/10.1111/j.1600-0714.1980.tb01386.x

Barfi Qasrdashti, A., Habashi, M. S., Arasteh, P., Torabi Ardakani, M., Abdoli, Z., & Eghbali, S. S. (2017). Malignant transformation in leukoplakia and its associated factors in southern Iran: A hospital based experience. *Iranian Journal of Public Health*, *46*(8), 1110–1117. Retrieved from https://pubmed.ncbi.nlm.nih.gov/28894713/

Bastos, D. B., Sarafim-Silva, B. A. M., Sundefeld, M. L. M. M., Ribeiro, A. A., Brandão, J. D. P., Biasoli, É. R., … Bernabé, D. G. (2018). Circulating catecholamines are associated with biobehavioral factors and anxiety symptoms in head and neck cancer patients. *PLoS ONE*, *13*(8), e0202515. https://doi.org/10.1371/journal.pone.0202515

Bathi, R. J., Rao, R., & Mutalik, S. (2009). GST null genotype and antioxidants: Risk indicators for oral pre-cancer and cancer. *Indian Journal of Dental Research*, *20*(3), 298–303. https://doi.org/10.4103/0970-9290.57365

Bedrick, A. E. (1970). Differential nucleolar staining of malignant and benign tissues with pontacyl dark green b1. *Biotechnic and Histochemistry*, *45*(6), 273–276. https://doi.org/10.3109/10520297009067800

Bezamat, M., Harrison, B., Zhou, Y., Glickman, K. M., Telles, V., Guirguis, C., … Vieira, A. R. (2020). Phenome-Wide Scan Finds Potential Orofacial Risk Markers for Cancer. *Scientific Reports*, *10*(1), 4869. https://doi.org/10.1038/s41598-020-61654-3

Bhat, S., Babu, S., Bhat, S., Castelino, R., Rao, K., & Madi, M. (2017). Status of serum and salivary ascorbic acid in oral potentially malignant disorders and oral cancer. *Indian Journal of Medical and Paediatric Oncology*, *38*(3), 306–310. https://doi.org/10.4103/ijmpo.ijmpo_67_16

Bhateja, S., & Arora, G. (2014). ABO blood groups and oral premalignancies: A clinical study in selected Indian population. *Indian Journal of Cancer*, *51*(3), 219–221. https://doi.org/10.4103/0019-509X.146722

Bhavana, Vs., Madhura, M., Kumar, Bv., Suma, S., & Sarita, Y. (2018). Detection of salivary heat shock protein 27 by enzyme-linked immunosorbent assay and its correlation with histopathology of oral leukoplakia. *Journal of Oral and Maxillofacial Pathology*, *22*(3), 307. https://doi.org/10.4103/jomfp.jomfp_86_18

Bhuvaneswari, M., Prasad, H., Rajmohan, M., Sri Chinthu, K., Prema, P., Mahalakshmi, L., & Kumar, G. (2022). Estimation of salivary lactate dehydrogenase in oral squamous cell carcinoma, oral leukoplakia, and smokers. *Journal of Cancer Research and Therapeutics*, *18*(9), 215–218. https://doi.org/10.4103/jcrt.JCRT_969_20

Bloching, M., Hofmann, A., Berghaus, A., Lautenschläger, C., & Grummt, T. (2000). Mikrokerne als biomarker zum nachweis der feldkanzerisierung im oberen aerodigestivtrakt. *Hno*, *48*(6), 444–450. https://doi.org/10.1007/s001060050595

Boccellino, M., De Rosa, A., & Di Domenico, M. (2023). An ELISA Test Able to Predict the Development of Oral Cancer: The Significance of the Interplay between Steroid Receptors and the EGF Receptor for Early Diagnosis. *Diagnostics*, *13*(12). https://doi.org/10.3390/diagnostics13122001

Bornstein, M. M., Lüönd-Valeskeviciute, I., Altermatt, H. J., Stauffer, E., & Buser, D. (2006). [Oral mucosal lesions diagnosed in a stomatology service. An examination of clinico-pathological findings from the year 2003]. *Schweizer Monatsschrift Fur Zahnmedizin = Revue Mensuelle Suisse d’odonto-Stomatologie = Rivista Mensile Svizzera Di Odontologia e Stomatologia*, *116*(5), 468–475. Retrieved from http://www.ncbi.nlm.nih.gov/pubmed/16792051

Bouquot, J. E., Weiland, L. H., & Kurland, L. T. (1988). Leukoplakia and carcinoma in situ synchronously associated with invasive oral/oropharyngeal carcinoma in Rochester, Minn., 1935-1984. *Oral Surgery, Oral Medicine, Oral Pathology*, *65*(2), 199–207. https://doi.org/10.1016/0030-4220(88)90166-1

Bovopoulou, O., Sklavounou, A., & Laskaris, G. (1985). Loss of intercellular substance antigens in oral hyperkeratosis, epithelial dysplasia, and squamous cell carcinoma. *Oral Surgery, Oral Medicine, Oral Pathology*, *60*(6), 648–654. https://doi.org/10.1016/0030-4220(85)90369-X

Brailo, V., Vučićević-Boras, V., Cekić-Arambašin, A., Alajbeg, I. Ž., Milenović, A., & Lukač, J. (2006). The significance of salivary interleukin 6 and tumor necrosis factor alpha in patients with oral leukoplakia. *Oral Oncology*, *42*(4), 370–373. https://doi.org/10.1016/j.oraloncology.2005.09.001

Brailo, Vlaho, Vucicevic-Boras, V., Lukac, J., Biocina-Lukenda, D., Zilic-Alajbeg, I., Milenovic, A., & Balija, M. (2012). Salivary and serum interleukin 1 beta, interleukin 6 and tumor necrosis factor alpha in patients with leukoplakia and oral cancer. *Medicina Oral, Patologia Oral y Cirugia Bucal*, *17*(1), e10-5. https://doi.org/10.4317/medoral.17323

Brands, R. C., Köhler, O., Rauthe, S., Hartmann, S., Ebhardt, H., Seher, A., … Müller-Richter, U. D. A. (2017). The prognostic value of GLUT-1 staining in the detection of malignant transformation in oral mucosa. *Clinical Oral Investigations*, *21*(5), 1631–1637. https://doi.org/10.1007/s00784-016-1954-5

Bremmer, J. F., Brakenhoff, R. H., Broeckaert, M. A. M., Beliën, J. A. M., Leemans, C. R., Bloemena, E., … Braakhuis, B. J. M. (2011). Prognostic value of DNA ploidy status in patients with oral leukoplakia. *Oral Oncology*, *47*(10), 956–960. https://doi.org/10.1016/j.oraloncology.2011.07.025

Bremmer, J. F., Graveland, A. P., Brink, A., Braakhuis, B. J. M., Kuik, D. J., Leemans, C. R., … Brakenhoff, R. H. (2009). Screening for oral precancer with noninvasive genetic cytology. *Cancer Prevention Research*, *2*(2), 128–133. https://doi.org/10.1158/1940-6207.CAPR-08-0128

Brito, J. A. R., Gomes, C. C., Guimarães, A. L. S., Campos, K., & Gomez, R. S. (2014). Relationship between microRNA expression levels and histopathological features of dysplasia in oral leukoplakia. *Journal of Oral Pathology and Medicine*, *43*(3), 211–216. https://doi.org/10.1111/jop.12112

Brouns, E., Baart, J. A., Karagozoglu, K. H., Aartman, I. H. A., Bloemena, E., & Van der Waal, I. (2014). Malignant transformation of oral leukoplakia in a well-defined cohort of 144 patients. *Oral Diseases*, *20*(3), e19-24. https://doi.org/10.1111/odi.12095

Brouns, E. R. E. A., Bloemena, E., Belien, J. A. M., Broeckaert, M. A. M., Aartman, I. H. A., & Van Der Waal, I. (2012). DNA ploidy measurement in oral leukoplakia: Different results between flow and image cytometry. *Oral Oncology*, *48*(7), 636–640. https://doi.org/10.1016/j.oraloncology.2012.01.013

Brouns, E. R., Evren, I., Wils, L. J., Poell, J. B., Brakenhoff, R. H., Bloemena, E., & de Visscher, J. G. A. M. (2023). Oral leukoplakia classification and staging system with incorporation of differentiated dysplasia. *Oral Diseases*, *29*(7), 2667–2676. https://doi.org/10.1111/odi.14295

Browne, R. M., & Potts, A. J. C. (1986). Dysplasia in salivary gland ducts in sublingual leukoplakia and erythroplakia. *Oral Surgery, Oral Medicine, Oral Pathology*, *62*(1), 44–49. https://doi.org/10.1016/0030-4220(86)90068-X

Brzak, B. L., Mravak-Stipetić, M., Canjuga, I., Baricević, M., Balicević, D., Sikora, M., & Filipović-Zore, I. (2012). The frequency and malignant transformation rate of oral lichen planus and leukoplakia--a retrospective study. *Collegium Antropologicum*, *36*(3), 773–777. Retrieved from http://www.ncbi.nlm.nih.gov/pubmed/23213931

Bukovszky, B., Fodor, J., Tóth, E., Kocsis, Z. S., Oberna, F., Ferenczi, Ö., & Polgár, C. (2023). Malignant Transformation and Long-Term Outcome of Oral and Laryngeal Leukoplakia. *Journal of Clinical Medicine*, *12*(13). https://doi.org/10.3390/jcm12134255

Bundgaard, T., Gaihede, M., Smensen, F. B., Spgaard, H., & Overgaard, J. (1992). Stereologic, histopathologic, flow cytometric, and clinical parameters in the prognostic evaluation of 74 patients with intraoral squamous cell carcinomas. *Cancer*, *70*(1), 1–13. https://doi.org/10.1002/1097-0142(19920701)70:1<1::AID-CNCR2820700102>3.0.CO;2-S

Cai, X., Zhang, J., Li, L., Liu, L., Tang, M., Zhou, X., … Li, T. (2024). Copy Number Alterations Predict Development of OSCC from Oral Leukoplakia. *Journal of Dental Research*, *103*(2), 138–146. https://doi.org/10.1177/00220345231217160

Cai, Xinjia, Zhang, J., Han, Y., Tang, Q., Zhang, H., & Li, T. (2021). Development and validation of a nomogram prediction model for malignant transformation of oral potentially malignant disorders. *Oral Oncology*, *123*, 105619. https://doi.org/10.1016/j.oraloncology.2021.105619

Cai, Xinjia, Zhang, J., Zhang, H., Zhou, X., Zhou, Z., Jing, F., … Li, T. (2024). Architectural and cytological features of epithelial dysplasia associated with transformation risk. *Oral Diseases*, *30*(5), 3028–3038. https://doi.org/10.1111/odi.14809

Campisi, G., Giovannelli, L., Ammatuna, P., Capra, G., Colella, G., Di Liberto, C., … D’Angelo, M. (2004). Proliferative verrucous vs conventional leukoplakia: No significantly increased risk of HPV infection. *Oral Oncology*, *40*(8), 835–840. https://doi.org/10.1016/j.oraloncology.2004.02.007

Campisi, Giuseppina, Giovannelli, L., Aricò, P., Lama, A., Di Liberto, C., Ammatuna, P., & D’Angelo, M. (2004). HPV DNA in clinically different variants of oral leukoplakia and lichen planus. *Oral Surgery, Oral Medicine, Oral Pathology, Oral Radiology and Endodontology*, *98*(6), 705–711. https://doi.org/10.1016/j.tripleo.2004.04.012

Cao, J., Jin, J. qiu, Deng, D. jun, & Liu, H. wei. (2016). Determination of human papillomavirus in oral leukoplakia,oral lichen planus and oral squamous cell carcinoma. *Beijing Da Xue Xue Bao. Yi Xue Ban = Journal of Peking University. Health Sciences*, *48*(1), 84–88. Retrieved from https://pubmed.ncbi.nlm.nih.gov/26885914/

Cao, J., Liu, H. W., & Jin, J. Q. (2007). The effect of oral candida to development of oral leukoplakia into cancer. *Zhonghua Yu Fang Yi Xue Za Zhi [Chinese Journal of Preventive Medicine]*, *41 Suppl*, 90–93. Retrieved from https://pubmed.ncbi.nlm.nih.gov/17767868/

Cao, J., Liu, H. wei, Liu, X. song, Jin, J. qiu, & Zhang, P. (2011). [Correlation between the quantity of oral mucosal micronucleus cells and cancerization]. *Beijing Da Xue Xue Bao. Yi Xue Ban = Journal of Peking University. Health Sciences*, *43*(4), 600–602. Retrieved from https://pubmed.ncbi.nlm.nih.gov/21844976/

Cerqueira, J. M. M., Pontes, F. S. C., Santos-Silva, A. R., de Almeida, O. P., e Costa, R. F., Fonseca, F. P., … Pontes, H. A. R. (2021). Malignant transformation of oral leukoplakia: A multicentric retrospective study in brazilian population. *Medicina Oral Patologia Oral y Cirugia Bucal*, *26*(3), e292–e299. https://doi.org/10.4317/medoral.24175

Cervigne, N. K., Machado, J., Goswami, R. S., Sadikovic, B., Bradley, G., Perez-Ordonez, B., … Kamel-Reid, S. (2014). Recurrent genomic alterations in sequential progressive leukoplakia and oral cancer: Drivers of oral tumorigenesis? *Human Molecular Genetics*, *23*(10), 2618–2628. https://doi.org/10.1093/hmg/ddt657

Cervigne, N. K., Reis, P. P., Machado, J., Sadikovic, B., Bradley, G., Galloni, N. N., … Kamel-Reid, S. (2009). Identification of a microRNA signature associated with progression of leukoplakia to oral carcinoma. *Human Molecular Genetics*, *18*(24), 4818–4829. https://doi.org/10.1093/hmg/ddp446

Chan, W. H., Chang, K. P., Yang, S. W., Yao, T. C., Ko, T. Y., Lee, Y. S., … Tsai, C. N. (2010). Transcriptional repression of DLEC1 associates with the depth of tumor invasion in oral squamous cell carcinoma. *Oral Oncology*, *46*(12), 874–879. https://doi.org/10.1016/j.oraloncology.2010.09.007

Chang, C. C., Lin, M. S., Chen, Y. T., Tu, L. T., Jane, S. W., & Chen, M. Y. (2015). Metabolic syndrome and health-related behaviours associated with pre-oral cancerous lesions among adults aged 20a-80 years in Yunlin County, Taiwan: A cross-sectional study. *BMJ Open*, *5*(12), e008788. https://doi.org/10.1136/bmjopen-2015-008788

Chang, L. Y., Lin, S. C., Chang, C. S., Wong, Y. K., Hu, Y. C., & Chang, K. W. (1999). Telomerase activity and in situ telomerase RNA expression in oral carcinogenesis. *Journal of Oral Pathology and Medicine*, *28*(9), 389–396. https://doi.org/10.1111/j.1600-0714.1999.tb02109.x

Chang, P. Y., Kuo, Y. Bin, Wu, T. L., Liao, C. T., Sun, Y. C., Yen, T. C., & Chan, E. C. (2013). Association and prognostic value of serum inflammation markers in patients with leukoplakia and oral cavity cancer. *Clinical Chemistry and Laboratory Medicine*, *51*(6), 1291–1300. https://doi.org/10.1515/cclm-2012-0504

Chang, S. E., Bhatia, P., Johnson, N. W., Morgan, P. R., McCormick, F., Young, B., & Hiorns, L. (1991). Ras mutations in united kingdom examples of oral malignancies are infrequent. *International Journal of Cancer*, *48*(3), 409–412. https://doi.org/10.1002/ijc.2910480318

Chang, Y. A., Weng, S. L., Yang, S. F., Chou, C. H., Huang, W. C., Tu, S. J., … Huang, H. Da. (2018). A three–MicroRNA signature as a potential biomarker for the early detection of oral cancer. *International Journal of Molecular Sciences*, *19*(3). https://doi.org/10.3390/ijms19030758

Chatterjee, S., Dhar, S., Sengupta, B., Ghosh, A., De, M., Roy, S., … Chakrabarti, S. (2009). Cytogenetic monitoring in human oral cancers and other oral pathology: The micronucleus test in exfoliated buccal cells Micronucleus test S. Chatterjee et al. *Toxicology Mechanisms and Methods*, *19*(6–7), 427–433. https://doi.org/10.1080/15376510903127530

Chattopadhyay, E., Singh, R., Ray, A., Roy, R., De Sarkar, N., Paul, R. R., … Roy, B. (2016). Expression deregulation of mir31 and CXCL12 in two types of oral precancers and cancer: Importance in progression of precancer and cancer. *Scientific Reports*, *6*, 32735. https://doi.org/10.1038/srep32735

Chaturvedi, P., Majumder, S. K., Krishna, H., Muttagi, S., & Gupta, P. K. (2010). Fluorescence spectroscopy for noninvasive early diagnosis of oral mucosal malignant and potentially malignant lesions. *Journal of Cancer Research and Therapeutics*, *6*(4), 497–502. https://doi.org/10.4103/0973-1482.77097

Chaudhari, V., Pradeep, G., Prakash, N., & Mahajan, A. (2016). Estimation of salivary sialic acid in oral premalignancy and oral squamous cell carcinoma. *Contemporary Clinical Dentistry*, *7*(4), 451–456. https://doi.org/10.4103/0976-237X.194108

Chaudhry, A., Manjunath, M., Ashwatappa, D., Krishna, S., & Krishna, A. G. (2016). Comparison of chemiluminescence and toluidine blue in the diagnosis of dysplasia in leukoplakia: a cross-sectional study. *Journal of Investigative and Clinical Dentistry*, *7*(2), 132–140. https://doi.org/10.1111/jicd.12141

Chen, H. C., Tseng, Y. K., Chi, C. C., Chen, Y. H., Yang, C. M., Huang, S. J., … Ger, L. P. (2016). Genetic variants in microRNA-146a (C > G) and microRNA-1269b (G > C) are associated with the decreased risk of oral premalignant lesions, oral cancer, and pharyngeal cancer. *Archives of Oral Biology*, *72*, 21–32. https://doi.org/10.1016/j.archoralbio.2016.08.010

Chen, H. H., Chen, I. H., Liao, C. T., Wei, F. C., Lee, L. Y., & Huang, S. F. (2011). Preoperative circulating C-reactive protein levels predict pathological aggressiveness in oral squamous cell carcinoma: A retrospective clinical study. *Clinical Otolaryngology*, *36*(2), 147–153. https://doi.org/10.1111/j.1749-4486.2011.02274.x

Chen, H., Liu, X., Jin, Z., Gou, C., Liang, M., Cui, L., & Zhao, X. (2018). A three miRNAs signature for predicting the transformation of oral leukoplakia to oral squamous cell carcinoma. *American Journal of Cancer Research*, *8*(8), 1403–1413. Retrieved from http://www.ncbi.nlm.nih.gov/pubmed/30210912%0Ahttp://www.pubmedcentral.nih.gov/articlerender.fcgi?artid=PMC6129488

Chen, H. Z. (1992). Rose bengal staining for clinical detection of oral premalignant lesions and carcinomas. *Zhonghua Kou Qiang Yi Xue Za Zhi = Zhonghua Kouqiang Yixue Zazhi = Chinese Journal of Stomatology*, *27*(1), 44–47. Retrieved from https://pubmed.ncbi.nlm.nih.gov/1379903/

Chen, I. A., Chiu, S. F., Hung, K. C., Yu, C. H., Chen, Y. C., Ho, C. H., … Chang, Y. J. (2022). Malignant transformation of oral potentially malignant disorders in Taiwanese indigenous peoples: A nationwide retrospective cohort study. *Medicine (United States)*, *101*(46), E31910. https://doi.org/10.1097/MD.0000000000031910

Cheng, B., Rhodus, N. L., Williams, B., & Griffin, R. J. (2004). Detection of apoptotic cells in whole saliva of patients with oral premalignant and malignant lesions: A preliminary study. *Oral Surgery, Oral Medicine, Oral Pathology, Oral Radiology, and Endodontics*, *97*(4), 465–470. https://doi.org/10.1016/j.tripleo.2003.12.020

Cheng, P., Li, H., Yang, P., & Tan, Y. (1996). C-Ha-ras oncogene in oral leukoplakia tissues. *Journal of Tongji Medical University = Tong Ji Yi Ke Da Xue Xue Bao*, *16*(1), 58–60. https://doi.org/10.1007/bf02889048

Cheng, R., Li, D., Shi, X., Gao, Q., Wei, C., Li, X., … Zhou, H. (2016). Reduced CX3CL1 secretion contributes to the susceptibility of oral leukoplakia-associated fibroblasts to Candida albicans. *Frontiers in Cellular and Infection Microbiology*, *6*(NOV), 150. https://doi.org/10.3389/fcimb.2016.00150

Cheng, S. J., Chang, C. F., Ko, H. H., Lee, J. J., Chen, H. M., Wang, H. J., … Chiang, C. P. (2018). Hypermethylated ZNF582 and PAX1 genes in mouth rinse samples as biomarkers for oral dysplasia and oral cancer detection. *Head and Neck*, *40*(2), 355–368. https://doi.org/10.1002/hed.24958

Chiang, W. F., Liu, S. Y., Lin, J. F., Chiu, S. F., Gou, S. Bin, Chiou, C. T., & Chang, C. H. (2020). Malignant development in patients with oral potentially malignant disorders detected through nationwide screening: Outcomes of 5-year follow-up at a single hospital. *Head and Neck*, *42*(1), 67–76. https://doi.org/10.1002/hed.25973

Christopher, V., Murthy, S., Ashwinirani, S. R., Singh, S., Athira, C. P., Shivaram, S. K., & Neethupriya. (2015). Morphometry as a diagnostic tool for potentially maligna nt lesions. *Journal of Clinical and Diagnostic Research*, *9*(12), ZC22–ZC25. https://doi.org/10.7860/JCDR/2015/15838.6959

Chuang, S. L., Wang, C. P., Chen, M. K., Su, W. W. Y., Su, C. W., Chen, S. L. S., … Yen, A. M. F. (2018). Malignant transformation to oral cancer by subtype of oral potentially malignant disorder: A prospective cohort study of Taiwanese nationwide oral cancer screening program. *Oral Oncology*, *87*, 58–63. https://doi.org/10.1016/j.oraloncology.2018.10.021

Chung, C. M., Hung, C. C., Lee, C. H., Lee, C. P., Lee, K. W., Chen, M. K., … Ko, Y. C. (2019). Variants in FAT1 and COL9A1 genes in male population with or without substance use to assess the risk factors for oral malignancy. *PLoS ONE*, *14*(1), e0210901. https://doi.org/10.1371/journal.pone.0210901

Cowan, C. G., Gregg, T. A., Napier, S. S., McKenna, S. M., & Kee, F. (2001). Potentially malignant oral lesions in Northern Ireland: A 20-year population-based perspective of malignant transformation. *Oral Diseases*, *7*(1), 18–24. https://doi.org/10.1034/j.1601-0825.2001.0070104.x

Cox, M., Maitlan, N., & Scully, C. (1993). Human herpes simplex-1 and papillomavirus type 16 homologous DNA sequences in normal, potentially malignant and malignant oral mucosa. *European Journal of Cancer. Part B: Oral Oncology*, *29*(3), 215–219. https://doi.org/10.1016/0964-1955(93)90025-A

Cui, J. J., Han, X. L., & Wang, W. M. (2013). Expression and significance of p53 and mdm2 in patients with leukoplakia cancer. *Asian Pacific Journal of Tropical Medicine*, *6*(10), 831–834. https://doi.org/10.1016/S1995-7645(13)60147-9

da Silva, A. M., Freitas, V. S., & Vieira, A. R. (2024). Polymorphisms associated with oral clefts as potential markers for oral pre and malignant disorders. *Oral Diseases*, *30*(5), 2985–2990. https://doi.org/10.1111/odi.14779

Daftary, D. K., Pitkar, V. K., Gupta, P. C., Pindborg, J. J., & Metha, F. S. (1978). A study of the natural history of oral preleukoplakia. *Acta Odontologica Scandinavica*, *36*(5–6), 327–331. https://doi.org/10.3109/00016357809029083

Dalla Torre, D., Burtscher, D., Edlinger, M., Sölder, E., Widschwendter, A., Rasse, M., & Puelacher, W. (2015). Comparison of the prevalence of human papilloma virus infection in histopathologically confirmed premalignant oral lesions and healthy oral mucosa by brush smear detection. *Oral Surgery, Oral Medicine, Oral Pathology and Oral Radiology*, *119*(3), 333–339. https://doi.org/10.1016/j.oooo.2014.11.013

Das, D., Maitra, A., Panda, C. K., Ghose, S., Roy, B., Sarin, R., & Majumder, P. P. (2021). Genes and pathways monotonically dysregulated during progression from normal through leukoplakia to gingivo-buccal oral cancer. *Npj Genomic Medicine*, *6*(1), 32. https://doi.org/10.1038/s41525-021-00195-8

Datta, S., Chattopadhyay, E., Ray, J. G., Majumder, M., Roy, P. Das, & Roy, B. (2015). D-loop somatic mutations and ∼5 kb “common” deletion in mitochondrial DNA: important molecular markers to distinguish oral precancer and cancer. *Tumor Biology*, *36*(4), 3025–3033. https://doi.org/10.1007/s13277-014-2937-2

Datta, S., Majumder, M., Biswas, N. K., Sikdar, N., & Roy, B. (2007). Increased risk of oral cancer in relation to common Indian mitochondrial polymorphisms and autosomal GSTP1 locus. *Cancer*, *110*(9), 1991–1999. https://doi.org/10.1002/cncr.23016

Datta, S., Ray, A., Roy, R., & Roy, B. (2016). Association of DNA sequence variation in mitochondrial DNA polymerase with mitochondrial DNA synthesis and risk of oral cancer. *Gene*, *575*(2), 650–654. https://doi.org/10.1016/j.gene.2015.09.039

Datta, S., Ray, A., Singh, R., Mondal, P., Basu, A., De Sarkar, N., … Roy, B. (2015). Sequence and expression variations in 23 genes involved in mitochondrial and non-mitochondrial apoptotic pathways and risk of oral leukoplakia and cancer. *Mitochondrion*, *25*, 28–33. https://doi.org/10.1016/j.mito.2015.09.001

Davidova, L. A., Fitzpatrick, S. G., Bhattacharyya, I., Cohen, D. M., & Islam, M. N. (2019). Lichenoid Characteristics in Premalignant Verrucous Lesions and Verrucous Carcinoma of the Oral Cavity. *Head and Neck Pathology*, *13*(4), 573–579. https://doi.org/10.1007/s12105-019-01006-4

Davidson, S., Kumar, S., Somasundaram, E., & Jaishankar, S. (2023). Assessment of brush biopsy findings and salivary LDH levels in oral mucosal lesions of tobacco users. *Indian Journal of Dental Research*, *34*(2), 191–195. https://doi.org/10.4103/ijdr.ijdr_228_21

de Azevedo, A. B., dos Santos, T. C. R. B., Lopes, M. A., & Pires, F. R. (2021). Oral leukoplakia, leukoerythroplakia, erythroplakia and actinic cheilitis: Analysis of 953 patients focusing on oral epithelial dysplasia. *Journal of Oral Pathology and Medicine*, *50*(8), 829–840. https://doi.org/10.1111/jop.13183

De Sarkar, N., Roy, R., Mitra, J. K., Ghose, S., Chakraborty, A., Paul, R. R., … Roy, B. (2014). A quest for miRNA bio-marker: A track back approach from gingivo buccal cancer to two different types of precancers. *PLoS ONE*, *9*(8), e104839. https://doi.org/10.1371/journal.pone.0104839

Decsi, G., Soki, J., Pap, B., Dobra, G., Harmati, M., Kormondi, S., … Buzas, K. (2019). Chicken or the Egg: Microbial Alterations in Biopsy Samples of Patients with Oral Potentially Malignant Disorders. *Pathology and Oncology Research*, *25*(3), 1023–1033. https://doi.org/10.1007/s12253-018-0457-x

Deepthi, G., Kulkarni, P. G., & Nandan, S. R. K. (2019). Eosinophils: An imperative histopathological prognostic indicator for oral squamous cell carcinoma. *Journal of Oral and Maxillofacial Pathology*, *23*(2), 307. https://doi.org/10.4103/jomfp.JOMFP_111_19

Deepthi, G., Nandan, S. R. K., & Kulkarni, P. G. (2019). Salivary tumour necrosis factor-α as a biomarker in oral leukoplakia and oral squamous cell carcinoma. *Asian Pacific Journal of Cancer Prevention*, *20*(7), 2087–2093. https://doi.org/10.31557/APJCP.2019.20.7.2087

Della Vella, F., Pannone, G., Patano, A., Ninivaggi, R., Del Prete, R., Lauritano, D., & Petruzzi, M. (2020). Detection of HPV in oral leukoplakia by brushing and biopsy: prospective study in an Italian cohort. *Clinical Oral Investigations*, *24*(5), 1845–1851. https://doi.org/10.1007/s00784-019-03048-y

Deneuve, S., Guerlain, J., Dupret-Bories, A., Majoufre, C., Philouze, P., Ceruse, P., … Fervers, B. (2022). Oral tongue squamous cell carcinomas in young patients according to their smoking status: a GETTEC study. *European Archives of Oto-Rhino-Laryngology*, *279*(1), 415–424. https://doi.org/10.1007/s00405-021-06793-7

Dikova, V., Jantus-Lewintre, E., & Bagan, J. (2021). Potential non-invasive biomarkers for early diagnosis of oral squamous cell carcinoma. *Journal of Clinical Medicine*, *10*(8). https://doi.org/10.3390/jcm10081658

Dineshkumar, T., Srikanth, P., Nagarathinam, A. E., Rajkumar, K., Priyadharini, S., & Shruthiquestionable, T. A. (2019). Diagnostic utility of cytology in assessment of ploidy status in potentially malignant oral disorders. *Asian Pacific Journal of Cancer Prevention*, *20*(10), 3145–3151. https://doi.org/10.31557/APJCP.2019.20.10.3145

Ding, L., Hu, E. L., Xu, Y. J., Huang, X. F., Zhang, D. Y., Li, B., … Hou, Y. Y. (2015). Serum IL-17F combined with VEGF as potential diagnostic biomarkers for oral squamous cell carcinoma. *Tumor Biology*, *36*(4), 2523–2529. https://doi.org/10.1007/s13277-014-2867-z

Ding, L., Li, B., Zhao, Y., Yi-Fu, F., Er-Ling, H., Qin-Gang, H., … Hou, Y. Y. (2014). Serum CCL2 and CCL3 as potential biomarkers for the diagnosis of oral squamous cell carcinoma. *Tumor Biology*, *35*(10), 10539–10546. https://doi.org/10.1007/s13277-014-2306-1

Dodia, V. S., Odedra, S. P., Himanshu Shah, K., Monpara, P. C., Vyas, P. M., & Pillai, J. P. (2022). The association of fingerprint patterns with oral potentially malignant disorders and oral cancer: A dermatoglyphic study. *Journal of Oral and Maxillofacial Pathology*, *26*(3), 420. https://doi.org/10.4103/jomfp.jomfp_261_21

Dong, Y. ying, Wang, J., Dong, F. sheng, Wang, X., Li, H. xiang, & Gu, H. tao. (2006). The relationship between methylation of p16 INK 4/CDKN2 gene in promoter region and progress of squamous cell carcinoma of buccal mucosa. *Zhonghua Kou Qiang Yi Xue Za Zhi = Zhonghua Kouqiang Yixue Zazhi = Chinese Journal of Stomatology*, *41*(5), 297–298. Retrieved from https://pubmed.ncbi.nlm.nih.gov/16784614/

Doseva, D., Christov, K., & Kristeva, K. (1984). DNA content in reactive hyperplasia, precancerosis, and carcinomas of the oral cavity A cytophotometric study. *Acta Histochemica*, *75*(2), 113–119. https://doi.org/10.1016/S0065-1281(84)80045-8

Dosi, T., Gupta, D., Hazari, A., Rajput, R., Chauhan, P., & Rajapuri, A. (2016). Assessment of micronuclei frequency in individuals with a habit of tobacco by means of exfoliated oral buccal cells. *Journal of International Society of Preventive and Community Dentistry*, *6*(8), S143–S147. https://doi.org/10.4103/2231-0762.189745

Doyle, J. L., & Manhold, J. H. (1975). Feulgen Microspectrophotometry of Oral Cancer and Leukoplakia. *Journal of Dental Research*, *54*(6), 1196–1199. https://doi.org/10.1177/00220345750540061601

Duarte, E. C. B., Da Silva, M. S. L., Gomez, M. V., & Gomez, R. S. (2006a). GSTM1 polymorphism and oral leukoplakia. *Journal of Oral Pathology and Medicine*, *35*(4), 202–205. https://doi.org/10.1111/j.1600-0714.2006.00405.x

Duarte, E. C. B., Da Silva, M. S. L., Gomez, M. V., & Gomez, R. S. (2006b). GSTM1 polymorphism and oral leukoplakia. *Journal of Oral Pathology and Medicine*, *35*(4), 202–205. https://doi.org/10.1111/j.1600-0714.2006.00405.x

Einhorn, J., & Wersäll, J. (1967). Incidence of oral carcinoma in patients with leukoplakia of the oral mucosa. *Cancer*, *20*(12), 2189–2193. https://doi.org/10.1002/1097-0142(196712)20:12<2189::AID-CNCR2820201218>3.0.CO;2-M

Elamir, A., ElRefai, S. M., & Ghazy, S. E. (2019). Molecular alterations of mitochondrial D-loop in oral leukoplakia. *Journal of Cellular Biochemistry*, *120*(8), 13944–13951. https://doi.org/10.1002/jcb.28668

Emilion, G., Langdon, J. D., Speight, P., & Partridge, M. (1996). Frequent gene deletions in potentially malignant oral lesions. *British Journal of Cancer*, *73*(6), 809–813. https://doi.org/10.1038/bjc.1996.142

Espinoza, I., Rojas, R., Aranda, W., & Gamonal, J. (2003). Prevalence of oral mucosal lesions in elderly people in Santiago, Chile. *Journal of Oral Pathology and Medicine*, *32*(10), 571–575. https://doi.org/10.1034/j.1600-0714.2003.00031.x

Evren, I., Brouns, E. R., Poell, J. B., Wils, L. J., Brakenhoff, R. H., Bloemena, E., & de Visscher, J. G. A. M. (2023). Associations between clinical and histopathological characteristics in oral leukoplakia. *Oral Diseases*, *29*(2), 696–706. https://doi.org/10.1111/odi.14038

Evren, I., Brouns, E. R., Wils, L. J., Poell, J. B., Peeters, C. F. W., Brakenhoff, R. H., … de Visscher, J. G. A. M. (2020). Annual malignant transformation rate of oral leukoplakia remains consistent: A long-term follow-up study. *Oral Oncology*, *110*, 105014. https://doi.org/10.1016/j.oraloncology.2020.105014

Evren, I., Najim, A. M., Poell, J. B., Brouns, E. R., Wils, L. J., Peferoen, L. A. N., … de Visscher, J. G. A. M. (2024). The value of regular follow-up of oral leukoplakia for early detection of malignant transformation. *Oral Diseases*, *30*(5), 2991–3003. https://doi.org/10.1111/odi.14797

Evstifeeva, T. V., & Zaridze, D. G. (1992). Nass use, cigarette smoking, alcohol consumption and risk of oral and oesophageal precancer. *European Journal of Cancer. Part B: Oral Oncology*, *28*(1), 29–35. https://doi.org/10.1016/0964-1955(92)90008-O

Fan, Y., Zhan, Z., Peng, T., Song, X. ling, & Feng, Z. qing. (2004). The expression of apoptosis-associated proteins Bcl-2, Bax in oral leukoplakia and lichen planus. *Shanghai Kou Qiang Yi Xue = Shanghai Journal of Stomatology*, *13*(6), 497–501. Retrieved from https://pubmed.ncbi.nlm.nih.gov/15619691/

Fang, Y., Chen, M., Li, G., Yang, Y., He, P., Chen, J., … Wu, H. (2022). Cancer-associated fibroblast-like fibroblasts in vocal fold leukoplakia suppress CD8+T cell functions by inducing IL-6 autocrine loop and interacting with Th17 cells. *Cancer Letters*, *546*, 215839. https://doi.org/10.1016/j.canlet.2022.215839

Fantozzi, P. J., Bavarian, R., Tamayo, I., Bind, M. A., Woo, S. Bin, & Villa, A. (2021). The role of family history of Cancer in Oral Cavity Cancer. *Head and Face Medicine*, *17*(1), 48. https://doi.org/10.1186/s13005-021-00298-8

Farah, C. S., & Fox, S. A. (2019). Dysplastic oral leukoplakia is molecularly distinct from leukoplakia without dysplasia. *Oral Diseases*, *25*(7), 1715–1723. https://doi.org/10.1111/odi.13156

Fathima, R., Ramamoorthi, R., Gopalakrishnan, S., Jayaseelan, V. P., & Muniapillai, S. (2024). Expression of salivary levels of S100A7 in oral submucous fibrosis and oral leukoplakia. *Journal of Oral and Maxillofacial Pathology*, *28*(1), 84–89. https://doi.org/10.4103/jomfp.jomfp_113_23

Feng, J., Zhou, Z., Shen, X., Wang, Y., Shi, L., Wang, Y., … Liu, W. (2015). Prevalence and distribution of oral mucosal lesions: A cross-sectional study in Shanghai, China. *Journal of Oral Pathology and Medicine*, *44*(7), 490–494. https://doi.org/10.1111/jop.12264

Ferreira, L. L., Biasoli, É. R., Bernabé, D. G., Nunes, C. M., & Miyahara, G. I. (2017). Plasma HPV DNA is detectable in oral leukoplakia patients. *Pathology Research and Practice*, *213*(7), 759–765. https://doi.org/10.1016/j.prp.2017.04.005

Ferrer-Sánchez, A., Bagan, J., Vila-Francés, J., Magdalena-Benedito, R., & Bagan-Debon, L. (2022). Prediction of the risk of cancer and the grade of dysplasia in leukoplakia lesions using deep learning. *Oral Oncology*, *132*, 105967. https://doi.org/10.1016/j.oraloncology.2022.105967

Fonseca-Silva, T., Diniz, M. G., de Sousa, S. F., Gomez, R. S., & Gomes, C. C. (2016). Association between histopathological features of dysplasia in oral leukoplakia and loss of heterozygosity. *Histopathology*, *68*(3), 456–460. https://doi.org/10.1111/his.12746

Foy, J. P., Bertolus, C., Ortiz-Cuaran, S., Albaret, M. A., Williams, W. N., Lang, W., … Saintigny, P. (2018). Immunological and classical subtypes of oral premalignant lesions. *OncoImmunology*, *7*(12), e1496880. https://doi.org/10.1080/2162402X.2018.1496880

Furuta, H., Kondo, Y., Nakahata, S., Hamasaki, M., Sakoda, S., & Morishita, K. (2010). NDRG2 is a candidate tumor-suppressor for oral squamous-cell carcinoma. *Biochemical and Biophysical Research Communications*, *391*(4), 1785–1791. https://doi.org/10.1016/j.bbrc.2009.12.156

Ganavi, B. S., Patil, S., & Rao, R. S. (2015). Evaluation of serum lipids and lipoproteins as prognosticators in leukoplakia. *Journal of Contemporary Dental Practice*, *15*(3), 294–299. https://doi.org/10.5005/jp-journals-10024-1531

Gandara-Vila, P., Pérez-Sayans, M., Suárez-Peñaranda, J. M., Gallas-Torreira, M., Somoza-Martín, J., Reboiras-López, M. D., … García-García, A. (2018). Survival study of leukoplakia malignant transformation in a region of northern Spain. *Medicina Oral Patologia Oral y Cirugia Bucal*, *23*(4), e413–e420. https://doi.org/10.4317/MEDORAL.22326

Ganesan, A., & Gautham Kumar, N. (2014). Assessment of lipid peroxides in multiple biofluids of leukoplakia and oral squamous cell carcinoma patients - A clinico- biochemical study. *Journal of Clinical and Diagnostic Research*, *8*(8), ZC55-8. https://doi.org/10.7860/JCDR/2014/10200.4768

Ganesh, D., Dafar, A., Niklasson, J., Sandberg, I., Braz-Silva, P., Sapkota, D., … Hasséus, B. (2023). EZH2 Expression Correlates With T-Cell Infiltration in Oral Leukoplakia and Predicts Cancer Transformation. *Anticancer Research*, *43*(4), 1533–1542. https://doi.org/10.21873/anticanres.16302

Gangadharan, P., & Paymaster, J. C. (1971). Leukoplakia-an epidemiologic study of 1504 cases observed at the Tata memorial hospital, Bombay, India. *British Journal of Cancer*, *25*(4), 657–668. https://doi.org/10.1038/bjc.1971.81

Gao, L., Chen, R., Sugimoto, M., Mizuta, M., Zhou, L., Kishimoto, Y., … Omori, K. (2021). The RNA Methylation Modification 5-Methylcytosine Impacts Immunity Characteristics, Prognosis and Progression of Oral Squamous Cell Carcinoma by Bioinformatics Analysis. *Frontiers in Bioengineering and Biotechnology*, *9*, 760724. https://doi.org/10.3389/fbioe.2021.760724

Gao, S. (1992). Cell morphometric analysis in oral submucous fibrosis, leukoplakia and squamous cell carcinoma. *Zhonghua Kou Qiang Yi Xue Za Zhi = Zhonghua Kouqiang Yixue Zazhi = Chinese Journal of Stomatology*, *27*(3), 145-147,189. Retrieved from https://pubmed.ncbi.nlm.nih.gov/1292898/

García-Pola Vallejo, M. J., García Martín, J. M., González García, M., & Telenti Arnáiz, P. (1997). Precancerous lesions (oral leukoplakia and lichen planus) in the geriatric patient. *Atencion Primaria / Sociedad Española de Medicina de Familia y Comunitaria*, *20*(1), 41–44. Retrieved from https://pubmed.ncbi.nlm.nih.gov/9303661/

Garg, D., Sunil, M. K., Singh, P. P., Singla, N., Rani, S. R. A., & Kaur, B. (2014). Serum lipid profile in oral precancer and cancer: a diagnostic or prognostic marker? *Journal of International Oral Health : JIOH*, *6*(2), 33–39. Retrieved from http://www.ncbi.nlm.nih.gov/pubmed/24876700%0Ahttp://www.pubmedcentral.nih.gov/articlerender.fcgi?artid=PMC4037787

Gau, S., Liu, S. F., & Shen, Z. H. (1994). Stereological analysis in oral precancerous lesions and squamous cell carcinoma. *Zhonghua Kou Qiang Yi Xue Za Zhi = Zhonghua Kouqiang Yixue Zazhi = Chinese Journal of Stomatology*, *29*(6), 360–363. Retrieved from https://pubmed.ncbi.nlm.nih.gov/7743883/

Gharat, L., Rathod, G. P., & Kandalgaonkar, S. (2013). Quantitative estimation of Serum Fibrinogen Degradation Product levels in Oral Premalignant and Malignant lesions. *Journal of International Oral Health : JIOH*, *5*(5), 65–72. Retrieved from http://www.ncbi.nlm.nih.gov/pubmed/24324307%0Ahttp://www.pubmedcentral.nih.gov/articlerender.fcgi?artid=PMC3845287

Ghosh, A., Das, C., Ghose, S., Maitra, A., Roy, B., Majumder, P. P., & Biswas, N. K. (2022). Integrative analysis of genomic and transcriptomic data of normal, tumour, and co-occurring leukoplakia tissue triads drawn from patients with gingivobuccal oral cancer identifies signatures of tumour initiation and progression. *Journal of Pathology*, *257*(5), 593–606. https://doi.org/10.1002/path.5900

Ghosh, R., Sharma, J. K., & Ghosh, P. K. (1988). Sister chromatid exchanges in the lymphocytes of patients with oral leukoplakia. *Cancer Genetics and Cytogenetics*, *36*(2), 177–182. https://doi.org/10.1016/0165-4608(88)90142-2

Gielkens, P. F., de Visscher, J. G., & van der Waal, I. (2003). [A white lesion of the oral mucosa: leukoplakia?]. *Een Orale Witte Slijmvliesafwijking: Leukoplakie?*, *147*(45), 2197–2201. Retrieved from http://ovidsp.ovid.com/ovidweb.cgi?T=JS&PAGE=reference&D=med4&NEWS=N&AN=14640054

Gilligan, G., Panico, R., Lazos, J., Morelatto, R., Belardinelli, P., Criscuolo, M. I., … Piemonte, E. (2024). Oral squamous cell carcinomas and oral potentially malignant disorders: A Latin American study. *Oral Diseases*, *30*(5), 2965–2984. https://doi.org/10.1111/odi.14778

Girja, K. P., Sundharam, B. S., Krishnan, P. A., & Devi, C. S. (2002). Biochemical changes of saliva in tobacco chewers tobacco smokers, alcohol consumers, leukoplakia and oral cancer patients. *Indian Journal of Dental Research : Official Publication of Indian Society for Dental Research*, *13*(2), 102–107. Retrieved from https://pubmed.ncbi.nlm.nih.gov/12420576/

Gissi, D. B., Gabusi, A., Tarsitano, A., Asioli, S., Rossi, R., Marchetti, C., … Morandi, L. (2020). Application of a non-invasive oral brushing procedure based on bisulfite sequencing of a 13-gene panel to study high-risk OSCC patients. *Cancer Biomarkers*, *28*(4), 499–510. https://doi.org/10.3233/CBM-190422

Gom es, C. C., Fonseca-Silva, T., Galvão, C. F., Friedman, E., De Marco, L., & Gomez, R. S. (2015). Inter- and intra-lesional molecular heterogeneity of oral leukoplakia. *Oral Oncology*, *51*(2), 178–181. https://doi.org/10.1016/j.oraloncology.2014.11.003

Gómez-Armayones, S., Chimenos-Küstner, E., Arranz, C., Tous, S., Marquez, S., Penín, R. M., … Mena, M. (2022). Risk factors for oral epithelial dysplasias to become malignant: clinical implications. *International Journal of Oral and Maxillofacial Surgery*, *51*(4), 473–480. https://doi.org/10.1016/j.ijom.2021.08.012

Goodson, M. L., Sloan, P., Robinson, C. M., Cocks, K., & Thomson, P. J. (2015). Oral precursor lesions and malignant transformation - Who, where, what, and when? *British Journal of Oral and Maxillofacial Surgery*, *53*(9), 831–835. https://doi.org/10.1016/j.bjoms.2015.08.268

Goodson, M. L., Smith, D. R., & Thomson, P. J. (2017). Efficacy of oral brush biopsy in potentially malignant disorder management. *Journal of Oral Pathology and Medicine*, *46*(10), 896–901. https://doi.org/10.1111/jop.12627

Gopinath, D., Thannikunnath, B. V., & Neermunda, S. F. (2016). Prevalence of carcinomatous foci in oral leukoplakia: A clinicopathologic study of 546 Indian samples. *Journal of Clinical and Diagnostic Research*, *10*(8), ZC78–ZC83. https://doi.org/10.7860/JCDR/2016/16815.8305

Govindarajan, G. V. V., Bhanumurthy, L., Balasubramanian, A., & Ramanathan, A. (2016). A novel mutation in the DNA binding domain of NFKB is associated with speckled leukoplakia. *Asian Pacific Journal of Cancer Prevention*, *17*(7), 3627–3629. Retrieved from https://pubmed.ncbi.nlm.nih.gov/27510021/

Goyal, G. (2020). Comparison of salivary and serum alkaline phosphates level and lactate dehydrogenase levels in patients with tobacco related oral lesions with healthy subjects-A step towards early diagnosis. *Asian Pacific Journal of Cancer Prevention*, *21*(4), 983–991. https://doi.org/10.31557/APJCP.2020.21.4.983

Granero Fernandez, M., & Lopez-Jornet, P. (2017). Association between smoking, glycaemia, blood lipoproteins and risk of oral leukoplakia. *Australian Dental Journal*, *62*(1), 47–51. https://doi.org/10.1111/adj.12431

Guerrero-Sánchez, Y., García, F. G., Chamorro-Petronacci, C. M., Suárez-Peñaranda, J. M., & Pérez-Sayáns, M. (2022). Use of the Fractal Dimension to Differentiate Epithelium and Connective Tissue in Oral Leukoplakias. *Cancers*, *14*(11). https://doi.org/10.3390/cancers14112697

Gupta, A., Gupta, S., & Mahdi, A. A. (2015). 1H NMR-derived serum metabolomics of leukoplakia and squamous cell carcinoma. *Clinica Chimica Acta*, *441*, 47–55. https://doi.org/10.1016/j.cca.2014.12.003

Gupta, J., Gupta, K., & Agarwal, R. (2019). Comparison of different stains in exfoliated oral mucosal cell micronucleus of potentially malignant disorders of oral cavity. *Journal of Cancer Research and Therapeutics*, *15*(3), 615–619. https://doi.org/10.4103/jcrt.JCRT_1326_16

Gupta, K., Gupta, J., & Miglani, R. (2016). Computer aided morphometric analysis of oral leukoplakia and oral squamous cell carcinoma. *Biotechnic and Histochemistry*, *91*(4), 251–254. https://doi.org/10.3109/10520295.2016.1139181

Gupta, N., Rakshit, A., Srivastava, S., Suryawanshi, H., Kumar, P., & Naik, R. (2019). Comparative evaluation of micronuclei in exfoliated oral epithelial cells in potentially malignant disorders and malignant lesions using special stains. *Journal of Oral and Maxillofacial Pathology*, *23*(1), 157. https://doi.org/10.4103/jomfp.JOMFP_164_17

Gupta, P. C., Bhonsle, R. B., Murti, P. R., Daftary, D. K., Mehta, F. S., & Pindborg, J. J. (1989). An epidemiologic assessment of cancer risk in oral precancerous lesions in India with special reference to nodular leukoplakia. *Cancer*, *63*(11), 2247–2252. https://doi.org/10.1002/1097-0142(19890601)63:11<2247::AID-CNCR2820631132>3.0.CO;2-D

Gupta, Prakash C. (1989). Leukoplakia and incidence of oral cancer. *Journal of Oral Pathology & Medicine*, *18*(1), 17–17. https://doi.org/10.1111/j.1600-0714.1989.tb00725.x

Gupta, Prakash C., Pindborg, J. J., Bhonsle, R. B., Murti, P. R., Mehta, F. S., Aghi, M. B., … Sinor, P. N. (1986). Intervention Study for Primary Prevention of Oral Cancer Among 36 000 Indian Tobacco Users. *The Lancet*, *327*(8492), 1235–1239. https://doi.org/10.1016/S0140-6736(86)91386-3

Gupta, S., Gupta, O. P., & Srivastava, S. (2014). Role of CYP2E1genetic polymorphism in the development of oral leukoplakia among tobacco users in North Indian population. *Indian Journal of Cancer*, *51*(2), 154–158. https://doi.org/10.4103/0019-509X.138266

Gupta, Swati, Shah, J., Parikh, S., Limbdiwala, P., & Goel, S. (2014). Clinical correlative study on early detection of oral cancer and precancerous lesions by modified oral brush biopsy and cytology followed by histopathology. *Journal of Cancer Research and Therapeutics*, *10*(2), 232–238. https://doi.org/10.4103/0973-1482.136539

Gurudath, S., Ganapathy, K. S., Sujatha, D., Pai, A., Ballal, S., & Ml, A. (2012). Estimation of superoxide dismutase and glutathione peroxidase in oral submucous fibrosis, oral leukoplakia and oral cancer - a comparative study. *Asian Pacific Journal of Cancer Prevention*, *13*(9), 4409–4412. https://doi.org/10.7314/APJCP.2012.13.9.4409

Guttenplan, J. B., Chen, K. M., Sun, Y. W., Shalaby, N. A. E., Kosinska, W., Desai, D., … El-Bayoumy, K. (2019). Effects of the Tobacco Carcinogens N′-Nitrosonornicotine and Dibenzo[ a, l]pyrene Individually and in Combination on DNA Damage in Human Oral Leukoplakia and on Mutagenicity and Mutation Profiles in lacI Mouse Tongue. *Chemical Research in Toxicology*, *32*(9), 1893–1899. https://doi.org/10.1021/acs.chemrestox.9b00257

Hadzic, S., GojkovVukelic, M., Pasic, E., & Dervisevic, A. (2017). Importance of Early Detection of Potentially Malignant Lesions in the Prevention of Oral Cancer. *Materia Socio Medica*, *29*(2), 129. https://doi.org/10.5455/msm.2017.29.129-133

Hamidi, S., Salo, T., Kainulainen, T., Epstein, J., Lerner, K., & Larjava, H. (2000). Expression of α(v)β6 integrin in oral leukeplakia. *British Journal of Cancer*, *82*(8), 1433–1440. https://doi.org/10.1054/bjoc.1999.1130

Hankinson, P. M., Mohammed-Ali, R. I., Smith, A. T., & Khurram, S. A. (2021). Malignant transformation in a cohort of patients with oral epithelial dysplasia. *British Journal of Oral and Maxillofacial Surgery*, *59*(9), 1099–1101. https://doi.org/10.1016/j.bjoms.2021.02.019

Hanna, G. J., Villa, A., Mistry, N., Jia, Y., Quinn, C. T., Turner, M. M., … Stephen Hodi, F. (2021). Comprehensive Immunoprofiling of High-Risk Oral Proliferative and Localized Leukoplakia. *Cancer Research Communications*, *1*(1), 30–40. https://doi.org/10.1158/2767-9764.CRC-21-0060

Hanna, G. J., Villa, A., Mistry, N., Jia, Y., Quinn, C. T., Turner, M. M., … Stephen Hodi, F. (2022). Correction: Comprehensive Immunoprofiling of High-risk Oral Proliferative and Localized Leukoplakia. Cancer research communications, 2(5), 390. https://doi.org/10.1158/2767-9764.CRC-22-0193

Hashimoto, K., Shimizu, D., Hirabayashi, S., Ueda, S., Miyabe, S., Oh-iwa, I., … Nomoto, S. (2019). Changes in oral microbial profiles associated with oral squamous cell carcinoma vs leukoplakia. *Journal of Investigative and Clinical Dentistry*, *10*(4), e12445. https://doi.org/10.1111/jicd.12445

Hashimoto, K., Shimizu, D., Ueda, S., Miyabe, S., Oh-Iwa, I., Nagao, T., … Nomoto, S. (2022). Feasibility of oral microbiome profiles associated with oral squamous cell carcinoma. *Journal of Oral Microbiology*, *14*(1), 2105574. https://doi.org/10.1080/20002297.2022.2105574

Hassan, N. M. M., Hamada, J. I., Kameyama, T., Tada, M., Nakagawa, K., Yoshida, S., … Moriuchi, T. (2011). Increased expression of the PRL-3 gene in human oral squamous cell carcinoma and dysplasia tissues. *Asian Pacific Journal of Cancer Prevention*, *12*(4), 947–951. Retrieved from https://pubmed.ncbi.nlm.nih.gov/21790231/

Haya-Fernández, M. C., Bagán, J. V., Murillo-Cortés, J., Poveda-Roda, R., & Calabuig, C. (2004). The prevalence of oral leukoplakia in 138 patients with oral squamous cell carcinoma. *Oral Diseases*, *10*(6), 346–348. https://doi.org/10.1111/j.1601-0825.2004.01031.x

He, H., Sun, G., Ping, F., & Cong, Y. (2011). A new and preliminary three-dimensional perspective: Proteomes of optimization between OSCC and OLK. *Artificial Cells, Blood Substitutes, and Biotechnology*, *39*(1), 26–30. https://doi.org/10.3109/10731199.2010.516258

Hernández-Arenas, Y. Y., Támara-De Ávila, J. J., Isaza-Guzmán, D. M., González-Pérez, L. V., & Tobón-Arroyave, S. I. (2021). Relationship of the XRCC1 rs25487 polymorphism with demographic, behavioral, clinical, and histological parameters in oral potentially malignant disorders and oral squamous cell carcinoma in a Colombian population. *Journal of Oral Biosciences*, *63*(2), 217–223. https://doi.org/10.1016/j.job.2021.02.006

Hernandez, B. Y., Zhu, X., Goodman, M. T., Gatewood, R., Mendiola, P., Quinata, K., & Paulino, Y. C. (2017). Betel nut chewing, oral premalignant lesions, and the oral microbiome. *PLoS ONE*, *12*(2), e0172196. https://doi.org/10.1371/journal.pone.0172196

Herreros-Pomares, A., Hervás, D., Bagán, L., Proaño, A., & Bagan, J. (2024). Proliferative verrucous and homogeneous Leukoplakias exhibit differential methylation patterns. *Oral Diseases*. https://doi.org/10.1111/odi.15028

Ho, M. W., Risk, J. M., Woolgar, J. A., Field, E. A., Field, J. K., Steele, J. C., … Shaw, R. J. (2012). The clinical determinants of malignant transformation in oral epithelial dysplasia. *Oral Oncology*, *48*(10), 969–976. https://doi.org/10.1016/j.oraloncology.2012.04.002

Hoffmann, R. R., Yurgel, L. S., & Campos, M. M. (2011). Evaluation of salivary endothelin-1 levels in oral squamous cell carcinoma and oral leukoplakia. *Regulatory Peptides*, *166*(1–3), 55–58. https://doi.org/10.1016/j.regpep.2010.08.006

Hogewind, W. F. C., & Van Der Waal, I. (1988). Leukoplakia of the labial commissure. *British Journal of Oral and Maxillofacial Surgery*, *26*(2), 133–140. https://doi.org/10.1016/0266-4356(88)90008-3

Hogewind, W. F. C., van der Waal, I., van der Kwast, W. A. M., & Snow, G. B. (1989). The association of white lesions with oral squamous cell carcinoma. A retrospective study of 212 patients. *International Journal of Oral and Maxillofacial Surgery*, *18*(3), 163–164. https://doi.org/10.1016/S0901-5027(89)80117-1

Hohberger, L., Wuertz, B. R. K., Xie, H., Griffin, T., & Ondrey, F. (2008). TNF-α drives matrix metalloproteinase-9 in squamous oral carcinogenesis. *Laryngoscope*, *118*(8), 1395–1399. https://doi.org/10.1097/MLG.0b013e318174e09b

Holmstrup, P., Vedtofte, P., Reibel, J., & Stoltze, K. (2006). Long-term treatment outcome of oral premalignant lesions. *Oral Oncology*, *42*(5), 461–474. https://doi.org/10.1016/j.oraloncology.2005.08.011

Hornstein, O. P., Schirner, E., & Schell, H. (1981). Sites of predilection of leukoplakia and carcinoma of the oral mucosa. *Deutsche Medizinische Wochenschrift*, *106*(37), 1168–1173. https://doi.org/10.1055/s-2008-1070475

Hosni, E. S., Salum, F. G., Cherubini, K., Yurgel, L. S., & Figueiredo, M. A. Z. (2009). Oral erythroplakia and speckled leukoplakia: Retrospective analysis of 13 cases. *Brazilian Journal of Otorhinolaryngology*, *75*(2), 295–299. https://doi.org/10.1016/S1808-8694(15)30793-X

Hou, Y. Y., Lee, J. H., Chen, H. C., Yang, C. M., Huang, S. J., Liou, H. H., … Ger, L. P. (2015). The association between miR-499a polymorphism and oral squamous cell carcinoma progression. *Oral Diseases*, *21*(2), 195–206. https://doi.org/10.1111/odi.12241

Hsu, H. J., Yang, Y. H., Shieh, T. Y., Chen, C. H., Kao, Y. H., Yang, C. F., & Ko, E. C. C. (2014). Role of cytokine gene (interferon-γ, transforming growth factor-β1, tumor necrosis factor-α, interleukin-6, and interleukin-10) polymorphisms in the risk of oral precancerous lesions in Taiwanese. *Kaohsiung Journal of Medical Sciences*, *30*(11), 551–558. https://doi.org/10.1016/j.kjms.2014.09.003

Hsue, S. S., Wang, W. C., Chen, C. H., Lin, C. C., Chen, Y. K., & Lin, L. M. (2007). Malignant transformation in 1458 patients with potentially malignant oral mucosal disorders: A follow-up study based in a Taiwanese hospital. *Journal of Oral Pathology and Medicine*, *36*(1), 25–29. https://doi.org/10.1111/j.1600-0714.2006.00491.x

Huang, H. I., Chen, C. H., Wang, S. H., Wang, L. H., & Lin, Y. C. (2019). Effects of APE1 Asp148Glu polymorphisms on OPMD malignant transformation, and on susceptibility to and overall survival of oral cancer in Taiwan. *Head and Neck*, *41*(6), 1557–1564. https://doi.org/10.1002/hed.25576

Idris, A. M., Warnakulasuriya, K. A. A. S., Ibrahim, Y. E., Nielsen, R., Cooper, D., & Johnson, N. W. (1996). Toombak-associated oral mucosal lesions in Sudanese show a low prevalence of epithelial dysplasia. *Journal of Oral Pathology and Medicine*, *25*(5), 239–244. https://doi.org/10.1111/j.1600-0714.1996.tb01378.x

Inchanalkar, M., Srivatsa, S., Ambatipudi, S., Bhosale, P. G., Patil, A., Schäffer, A. A., … Mahimkar, M. B. (2023). Genome-wide DNA methylation profiling of HPV-negative leukoplakia and gingivobuccal complex cancers. *Clinical Epigenetics*, *15*(1), 93. https://doi.org/10.1186/s13148-023-01510-z

Iqbal, J., Patil, R., Khanna, V., Tripathi, A., Singh, V., Munshi, M. I., & Tiwari, R. (2020). Role of fractal analysis in detection of dysplasia in potentially malignant disorders. *Journal of Family Medicine and Primary Care*, *9*(5), 2448. https://doi.org/10.4103/jfmpc.jfmpc_159_20

Irfan, S., Zaidi, N., Tiwari, K., Lal, N., Srivastava, A. N., & Singh, S. (2023). Evaluation of salivary endothelin-1 as a biomarker for oral cancer and precancer. *Journal of Cancer Research and Therapeutics*. https://doi.org/10.4103/jcrt.jcrt_2664_22

Islam, M. N., Kornberg, L., Veenker, E., Cohen, D. M., & Bhattacharyya, I. (2010). Anatomic site based ploidy analysis of oral premalignant lesions. *Head and Neck Pathology*, *4*(1), 10–14. https://doi.org/10.1007/s12105-009-0151-0

Izumchenko, E., Sun, K., Jones, S., Brait, M., Agrawal, N., Koch, W., … Sidransky, D. (2015). Notch1 mutations are drivers of oral tumorigenesis. *Cancer Prevention Research*, *8*(4), 277–286. https://doi.org/10.1158/1940-6207.CAPR-14-0257

Jacob, B. J., Straif, K., Thomas, G., Ramadas, K., Mathew, B., Zhang, Z. F., … Hashibe, M. (2004). Betel quid without tobacco as a risk factor for oral precancers. *Oral Oncology*, *40*(7), 697–704. https://doi.org/10.1016/j.oraloncology.2004.01.005

Jain, M., Kasetty, S., Sudheendra, U. S., Tijare, M., Khan, S., & Desai, A. (2014). Assessment of tissue eosinophilia as a prognosticator in oral epithelial dysplasia and oral squamous cell carcinoma - An image analysis study. *Pathology Research International*, *2014*, 507512. https://doi.org/10.1155/2014/507512

Jain, V., Mahajan, R., Rathi, S., Biyani, V., Ninama, K., & Marfatia, Y. (2023). Oral mucosal lesions-A study of 369 cases. *Indian Dermatology Online Journal*, *14*(2), 213–220. https://doi.org/10.4103/idoj.idoj_203_22

Jalouli, M. M., Jalouli, J., Hasséus, B., Öhman, J., Hirsch, J. M., & Sand, L. (2015). Nested PCR for detection of HSV-1 in oral mucosa. *Medicina Oral, Patologia Oral y Cirugia Bucal*, *20*(6), e664–e669. https://doi.org/10.4317/medoral.20630

Jane, C., Nerurkar, A. V., & Karjodkar, F. R. (2007). Circulating Immune Complexes (CIC) as marker for disease progress in oral cancer. *Indian Journal of Clinical Biochemistry*, *22*(2), 114–117. https://doi.org/10.1007/BF02913327

Jäwert, F., Pettersson, H., Jagefeldt, E., Holmberg, E., Kjeller, G., & Öhman, J. (2021). Clinicopathologic factors associated with malignant transformation of oral leukoplakias: a retrospective cohort study. *International Journal of Oral and Maxillofacial Surgery*, *50*(11), 1422–1428. https://doi.org/10.1016/j.ijom.2021.01.012

Jäwert, Fredrik, Fehr, A., Öhman, J., Stenman, G., & Kjeller, G. (2022). Recurrent copy number alterations involving EGFR, CDKN2A, and CCND1 in oral premalignant lesions. *Journal of Oral Pathology and Medicine*, *51*(6), 546–552. https://doi.org/10.1111/jop.13303

Jäwert, Fredrik, Nyman, J., Olsson, E., Adok, C., Helmersson, M., & Öhman, J. (2021). Regular clinical follow-up of oral potentially malignant disorders results in improved survival for patients who develop oral cancer. *Oral Oncology*, *121*, 105469. https://doi.org/10.1016/j.oraloncology.2021.105469

Jayachnadran, S., Prakasarao, A., Ramamoorthy, S., & Manoharan, Y. (2023). Significance of Fluorescent Spectroscopy in Screening Oral Potentially Malignant Disorders and Oral Cancer by Characterization of Salivary DNA Using Ethidium Bromide-A Comparative Study. *South Asian Journal of Cancer*, *12*(2), 159–165. https://doi.org/10.1055/s-0042-1750186

Jayadeep, A., Raveendran Pillai, K., Kannan, S., Nalinakumari, K. R., Mathew, B., Nair, M. K., & Menon, V. P. (1997). Serum levels of copper, zinc, iron and ceruloplasmin in oral leukoplakia and squamous cell carcinoma. *Journal of Experimental and Clinical Cancer Research*, *16*(3), 295–300. Retrieved from https://pubmed.ncbi.nlm.nih.gov/9387904/

Jayasooriya, P. R., Dayaratne, K., Dissanayake, U. B., & Warnakulasuriya, S. (2020). Malignant transformation of oral leukoplakia: a follow-up study. *Clinical Oral Investigations*, *24*(12), 4563–4569. https://doi.org/10.1007/s00784-020-03322-4

Jiakuan, P., Hongxia, D., Hao, X., Xin, Z., & Qianming, C. (2022). Agreement evaluation of the severity of oral epithelial dysplasia in oral leukoplakia. *Chinese Journal of Stomatology*, *57*(9), 921–926. https://doi.org/10.3760/cma.j.cn112144-20211206-00537

Jiang, W. W., Fujii, H., Shirai, T., Mega, H., & Takagi, M. (2001). Accumulative increase of loss heterozygosity from leukoplakia to foci of early cancerization in leukoplakia of the oral cavity. *Cancer*, *92*(9), 2349–2356. https://doi.org/10.1002/1097-0142(20011101)92:9<2349::AID-CNCR1582>3.0.CO;2-I

Jing, F., Zhang, J., Cai, X., Zhou, X., Bai, J., Zhang, H., & Li, T. (2022). Screening for Biomarkers for Progression from Oral Leukoplakia to Oral Squamous Cell Carcinoma and Evaluation of Diagnostic Efficacy by Multiple Machine Learning Algorithms. *Cancers*, *14*(23). https://doi.org/10.3390/cancers14235808

Joshi, P. S., & Golgire, S. (2014). A study of salivary lactate dehydrogenase isoenzyme levels in patients with oral leukoplakia and squamous cell carcinoma by gel electrophoresis method. *Journal of Oral and Maxillofacial Pathology*, *18*(5), 39–44. https://doi.org/10.4103/0973-029X.141342

Jurczyszyn, K., Gedrange, T., & Kozakiewicz, M. (2020). Theoretical Background to Automated Diagnosing of Oral Leukoplakia: A Preliminary Report. *Journal of Healthcare Engineering*, *2020*, 8831161. https://doi.org/10.1155/2020/8831161

Kämmerer, P. W., Koch, F. P., Santoro, M., Babaryka, G., Biesterfeld, S., Brieger, J., & Kunkel, M. (2013). Prospective, blinded comparison of cytology and DNA-image cytometry of brush biopsies for early detection of oral malignancy. *Oral Oncology*, *49*(5), 420–426. https://doi.org/10.1016/j.oraloncology.2012.12.006

Kannan, S., Balaram, P., Radhakrishna Pillai, M., Jagadeesh Chandran, G., Krishnan Nair, M., Kartha, C. C., … Mangalam, M. K. (1993). Ultrastructural Variations and Assessment of Malignant Transformation Risk in Oral Leukoplakia. *Pathology Research and Practice*, *189*(10), 1169–1180. https://doi.org/10.1016/S0344-0338(11)80840-X

Kanwar, S., Lingaraju, N., Mahesh, M. S., Basappa, S., & Rani, P. (2017). Genetic changes in oral premalignant lesion, condition, and oral squamous cell carcinoma-A study based on inhibition of G2M phase by colchicines. *Indian Journal of Dental Research*, *28*(1), 55–58. https://doi.org/10.4103/ijdr.IJDR_373_15

Katarkar, A., Mukherjee, S., Khan, M. H., Ray, J. G., & Chaudhuri, K. (2014). Comparative evaluation of genotoxicity by micronucleus assay in the buccal mucosa over comet assay in peripheral blood in oral precancer and cancer patients. *Mutagenesis*, *29*(5), 325–334. https://doi.org/10.1093/mutage/geu023

Kaur, J., Srivastava, A., & Ralhan, R. (1996). p53-HSP70 complexes in oral dysplasia and cancer: Potential prognostic implications. *European Journal of Cancer Part B: Oral Oncology*, *32*(1), 45–49. https://doi.org/10.1016/0964-1955(95)00054-2

Kaur, Jasdeep, & Jacobs, R. (2015a). Combination of autofluorescence imaging and salivary protoporphyrin in oral precancerous and cancerous lesions: Non-invasive tools. *Journal of Clinical and Experimental Dentistry*, *7*(2), e187–e191. https://doi.org/10.4317/jced.52100

Kaur, Jasdeep, & Jacobs, R. (2015b). Proinflammatory cytokine levels in oral lichen planus, oral leukoplakia, and oral submucous fibrosis. *Journal of the Korean Association of Oral and Maxillofacial Surgeons*, *41*(4), 171. https://doi.org/10.5125/jkaoms.2015.41.4.171

Kaur, Jasdeep, Politis, C., & Jacobs, R. (2015). Salivary apoptotic cells in oral (pre-) cancer as a potential diagnostic means. *Journal of Clinical and Experimental Dentistry*, *7*(3), e400–e404. https://doi.org/10.4317/jced.52212

Kaur, Jasdeep, Politis, C., & Jacobs, R. (2016). Salivary 8-hydroxy-2-deoxyguanosine, malondialdehyde, vitamin C, and vitamin E in oral pre-cancer and cancer: diagnostic value and free radical mechanism of action. *Clinical Oral Investigations*, *20*(2), 315–319. https://doi.org/10.1007/s00784-015-1506-4

Kaur, N., Zaheer, S., Sangwan, S., & Ranga, S. (2023). To evaluate eosinophilia (tissue eosinophilia, blood eosinophilia and tissue eosinophilia/blood eosinophilia ratio) in increasing grades/ severity of oral neoplastic lesions. *Journal of Cancer Research and Therapeutics*, *19*(8), S206–S211. https://doi.org/10.4103/jcrt.JCRT_1628_20

Khan, A. S., Khan, Z. A., Nisar, M., Saeed, S., Maryam, H., Haq, M., … Issrani, R. (2023). Description of clinicopathological characteristics of oral potentially malignant disorders with special focus on two histopathologic grading systems and subepithelial inflammatory infiltrate. *Journal of Cancer Research and Therapeutics*, *19*, S724–S730. https://doi.org/10.4103/jcrt.jcrt_969_22

Khan, M. M., Frustino, J., Villa, A., Nguyen, B. C., Woo, S. Bin, Johnson, W. E., … Monti, S. (2023). Total RNA sequencing reveals gene expression and microbial alterations shared by oral pre-malignant lesions and cancer. *Human Genomics*, *17*(1). https://doi.org/10.1186/s40246-023-00519-y

Khan, S. U., Connor, T. E. O., & Keogh, I. J. (2015). A series of oral lesions presenting to an otolaryngology department. *Irish Medical Journal*, *108*(6), 1–2. Retrieved from https://pubmed.ncbi.nlm.nih.gov/26182801/

Khanna, R., Thapa, P. B., Khanna, H. D., Khanna, S., Khanna, A. K., & Shukla, H. S. (2005). Lipid peroxidation and antioxidant enzyme status in oral carcinoma patients. *Kathmandu University Medical Journal (KUMJ)*, *3*(4), 334–339. Retrieved from https://pubmed.ncbi.nlm.nih.gov/16449831/

Khanna, Rahul, Agarwal, A., Khanna, S., Basu, S., & Khanna, A. K. (2010). S-phase fraction and DNA ploidy in oral leukoplakia. *ANZ Journal of Surgery*, *80*(7–8), 548–551. https://doi.org/10.1111/j.1445-2197.2009.05196.x

Kierce, J., Shi, Y., Klieb, H., Blanas, N., Xu, W., & Magalhaes, M. (2021). Identification of specific clinical risk factors associated with the malignant transformation of oral epithelial dysplasia. *Head and Neck*, *43*(11), 3552–3561. https://doi.org/10.1002/hed.26851

Kil, T. J., Kim, H. S., Kim, H. J., Nam, W., & Cha, I. H. (2016). Genetic abnormalities in oral leukoplakia and oral cancer progression. *Asian Pacific Journal of Cancer Prevention*, *17*(6), 3001–3006. Retrieved from https://pubmed.ncbi.nlm.nih.gov/27356725/

Kim, J., Shin, D. M., El-Naggar, A., Lee, J. S., Corrales, C., Lippman, S. M., … Hittelman, W. N. (2001). Chromosome polysomy and histological characteristics in oral premalignant lesions. *Cancer Epidemiology Biomarkers and Prevention*, *10*(4), 319–325. Retrieved from https://pubmed.ncbi.nlm.nih.gov/11319171/

Kinoshita, Y., Inoue, S., Honma, Y., & Shimura, K. (1992). Diagnostic significance of nuclear DNA content and nuclear area in oral hyperplasia, dysplasia, and carcinoma. *Journal of Oral and Maxillofacial Surgery*, *50*(7), 728–733. https://doi.org/10.1016/0278-2391(92)90108-C

Kitabatake, K., Ishikawa, S., Sugimoto, M., Enomoto, A., Kaneko, M., Ota, S., … Iino, M. (2023). Salivary metabolomics for oral leukoplakia with and without dysplasia. *Journal of Stomatology, Oral and Maxillofacial Surgery*, *124*(6), 101618. https://doi.org/10.1016/j.jormas.2023.101618

Kizhakkoottu, S., & Ramani, P. (2024). Knowledge About the Importance of Early Diagnosis and Treatment of Oral Potentially Malignant Disorders Among the South Indian Population: An Institutional Retrospective Study. *Cureus*, *16*(4), e57740. https://doi.org/10.7759/cureus.57740

Kohli, M., Ahuja, P., Mehendiratta, M., Sharma, M., & Dutta, J. (2017). Micronucleus assay: An early diagnostic tool to assess genotoxic changes in patients with tobacco use, oral leukoplakia and oral submucous fibrosis. *Journal of Clinical and Diagnostic Research*, *11*(9), ZC28–ZC32. https://doi.org/10.7860/JCDR/2017/27711.10567

Kolenko, Y. G., Volovyk, I. A., Bidenko, N. V., Mialkivskyi, K. O., & Tkachenko, I. M. (2022). Buccal Cell Micronuclei Among Patients With Oral Leukoplakia. *Wiadomosci Lekarskie (Warsaw, Poland : 1960)*, *75*(6), 1713–1717. https://doi.org/10.36740/WLek202207119

Kouketsu, A., Doi, C., Tanaka, H., Araki, T., Nakayama, R., Toyooka, T., … Takahashi, T. (2024). Detection of oral cancer and oral potentially malignant disorders using artificial intelligence-based image analysis. *Head and Neck*, *46*(9), 2253–2260. https://doi.org/10.1002/hed.27843

Kramer, I. R. H., El-Labban, N. G., & Sonkodi, S. (1974). Further studies on lesions of the oral mucosa using computer-aided analyses of histological features. *British Journal of Cancer*, *29*(3), 223–231. https://doi.org/10.1038/bjc.1974.61

Kramer, I. R. H., Lucas, R. B., El-Labban, N., & Lister, L. (1970a). A computer-aided study on the tissue changes in oral keratoses and lichen planus, and an analysis of case groupings by subjective and objective criteria. *British Journal of Cancer*, *24*(3), 407–426. https://doi.org/10.1038/bjc.1970.49

Kramer, I. R. H., Lucas, R. B., El-Labban, N., & Lister, L. (1970b). The use of discriminant analysis foe examining the histological features of oral keratoses and lichen planus. *British Journal of Cancer*, *24*(4), 673–683. https://doi.org/10.1038/bjc.1970.80

Krishnan, K., & Balasundaram, S. (2017). Estimation of total and lipid bound sialic acid in serum in oral leukoplakia. *Journal of Clinical and Diagnostic Research*, *11*(3), ZC25–ZC27. https://doi.org/10.7860/JCDR/2017/16483.9497

Krogh, P., Holmstrup, P., Vedtofte, P., & Pindborg, J. J. (1986). Yeast organisms associated with human oral leukoplakia. *Acta Dermato-Venereologica*, *66*(SUPPL. 121), 51–55. Retrieved from https://pubmed.ncbi.nlm.nih.gov/3459346/

Kudva, A., Kumar, M., John, E. R., & Dhara, V. (2023). Occurrence of Second Oral Potentially Malignant Disorder following Excision of Primary Lesion: A Prospective Study of Cases from a Tertiary Care Centre. *Journal of Maxillofacial and Oral Surgery*, *22*(1), 252–257. https://doi.org/10.1007/s12663-022-01764-9

Kujan, O., Huang, G., Ravindran, A., Vijayan, M., & Farah, C. S. (2019). CDK4, CDK6, cyclin D1 and Notch1 immunocytochemical expression of oral brush liquid-based cytology for the diagnosis of oral leukoplakia and oral cancer. *Journal of Oral Pathology and Medicine*, *48*(7), 566–573. https://doi.org/10.1111/jop.12902

Kujan, O., Idrees, M., Anand, N., Soh, B., Wong, E., & Farah, C. S. (2021). Efficacy of oral brush cytology cell block immunocytochemistry in the diagnosis of oral leukoplakia and oral squamous cell carcinoma. *Journal of Oral Pathology and Medicine*, *50*(5), 451–458. https://doi.org/10.1111/jop.13153

Kumar, A., Asiedu, E., Hefni, E., Armstrong, C., Menon, D., Ma, T., … Montaner, S. (2024). Angiopoietin-like 4 is upregulated by amphiregulin and activates cell proliferation and migration through p38 kinase in head and neck squamous cell carcinoma. *Journal of Oral Pathology and Medicine*, *53*(6), 366–375. https://doi.org/10.1111/jop.13545

Kumar, G. K., Abidullah, M., Elbadawi, L., Dakhil, S., & Mawardi, H. (2019). Epidemiological profile and clinical characteristics of oral potentially malignant disorders and oral squamous cell carcinoma: A pilot study in Bidar and Gulbarga Districts, Karnataka, India. *Journal of Oral and Maxillofacial Pathology*, *23*(1), 90–96. https://doi.org/10.4103/jomfp.JOMFP_116_18

Kumar, S., Suhag, A., Kolay, S., Kumar, P., Narwal, A., Srinivas, K., & Haideri, S. (2019). Serum fucose level in oral cancer, leukoplakia, and oral sub mucous fibrosis: A biochemical study. *Journal of Family Medicine and Primary Care*, *8*(7), 2414. https://doi.org/10.4103/jfmpc.jfmpc_301_19

Kuribayashi, Y., Morita, K. I., Tomioka, H., Uekusa, M., Ito, D., & Omura, K. (2009). Gene expression analysis by oligonucleotide microarray in oral leukoplakia. *Journal of Oral Pathology and Medicine*, *38*(4), 356–361. https://doi.org/10.1111/j.1600-0714.2008.00731.x

Kuribayashi, Y., Tsushima, F., Morita, K. I., Matsumoto, K., Sakurai, J., Uesugi, A., … Harada, H. (2015). Long-term outcome of non-surgical treatment in patients with oral leukoplakia. *Oral Oncology*, *51*(11), 1020–1025. https://doi.org/10.1016/j.oraloncology.2015.09.004

Kusiak, A., Maj, A., Cichońska, D., Kochańska, B., Cydejko, A., & Świetlik, D. (2020). The analysis of the frequency of leukoplakia in reference of tobacco smoking among northern polish population. *International Journal of Environmental Research and Public Health*, *17*(18), 1–9. https://doi.org/10.3390/ijerph17186919

Kuthoor, J., Sunil, E., Raghavan, S., & Purushothaman, B. (2022). A comparative investigation of the activity of superoxide dismutase in patients with leukoplakia and healthy controls. *Indian Journal of Dental Research*, *33*(3), 287–291. https://doi.org/10.4103/ijdr.ijdr_1009_21

Lamey, P. J., & Douglas, P. S. (1994). Secretor status and oral cancer. *British Journal of Oral and Maxillofacial Surgery*, *32*(4), 214–217. https://doi.org/10.1016/0266-4356(94)90205-4

Langvad, E., & Roed‐Petersen, B. (1970). Lactate Dehydrogenase Isoenzyme Patterns in Oral Leukoplakias and in Clinically Uninvolved Oral Mucosa of the Same Persons. *Acta Pathologica Microbiologica Scandinavica Section A Pathology*, *78 A*(5), 505–508. https://doi.org/10.1111/j.1699-0463.1970.tb02532.x

Lazarus, P., Garewal, H. S., Sciuuba, J., Zwiebel, N., Calcagnotto, A., Fair, A., … Richie, J. P. (1995). A low incidence of p53 mutations in pre‐malignant lesions of the oral cavity from non‐tobacco users. *International Journal of Cancer*, *60*(4), 458–463. https://doi.org/10.1002/ijc.2910600406

Lee, J. J., Lin, C. L., Chen, T. H. H., Kok, S. H., Chang, M. C., & Jeng, J. H. (2010). Changes in peripheral blood lymphocyte phenotypes distribution in patients with oral cancer/oral leukoplakia in Taiwan. *International Journal of Oral and Maxillofacial Surgery*, *39*(8), 806–814. https://doi.org/10.1016/j.ijom.2010.04.045

Lee, J. S., Kim, S. Y., Hong, W. K., Lippman, S. M., Ro, J. Y., Gay, M. L., & Hittelman, W. N. (1993). Detection of chromosomal polysomy in oral leukoplakia, a premalignant lesion. *Journal of the National Cancer Institute*, *85*(23), 1951–1954. https://doi.org/10.1093/jnci/85.23.1951

Lee, Jang Jaer, Hung, H. C., Cheng, S. J., Chen, Y. J., Chiang, C. P., Liu, B. Y., … Kok, S. H. (2006). Carcinoma and dysplasia in oral leukoplakias in Taiwan: Prevalence and risk factors. *Oral Surgery, Oral Medicine, Oral Pathology, Oral Radiology and Endodontology*, *101*(4), 472–480. https://doi.org/10.1016/j.tripleo.2005.07.024

Lee, K. W., & Chin, C. T. (1970). The effects of betel-nut chewing on the buccal mucosa: A histological study. *British Journal of Cancer*, *24*(3), 433–441. https://doi.org/10.1038/bjc.1970.51

Lee, S. Y., Cho, N. H., Choi, E. C., Baek, S. J., Kim, W. S., Shin, D. H., & Kim, S. H. (2010). Relevance of human papilloma virus (HPV) infection to carcinogenesis of oral tongue cancer. *International Journal of Oral and Maxillofacial Surgery*, *39*(7), 678–683. https://doi.org/10.1016/j.ijom.2010.03.014

Lehner, T. (1970). Immunopathology of oral leukoplakia. *British Journal of Cancer*, *24*(3), 442–446. https://doi.org/10.1038/bjc.1970.52

Lenz, C., Dietz, A., Pfuhl, A., Finckh, M., Conradt, C., Weidauer, H., & Bosch, F. X. (2000). Detection of numerical chromosomal aberrations in oral leukoplakias and HNSCC by fluorescence in situ hybridization. *Hno*, *48*(5), 367–371. https://doi.org/10.1007/s001060050582

Lenz, C. F., Pfuhl, A., Finckh, M., Weidauer, H., & Bosch, F. X. (1998). Oral leukoplakias show numerical chromosomal aberrations detected by fluorescence in situ hybridization. *Laryngoscope*, *108*(6), 917–922. https://doi.org/10.1097/00005537-199806000-00023

Li, C. C., Almazrooa, S., Carvo, I., Salcines, A., & Woo, S. Bin. (2021). Architectural Alterations in Oral Epithelial Dysplasia are Similar in Unifocal and Proliferative Leukoplakia. *Head and Neck Pathology*, *15*(2), 443–460. https://doi.org/10.1007/s12105-020-01216-1

Li, Chenxi, Wu, L., Deng, Y., Shen, X., Liu, W., & Shi, L. (2020). DNA aneuploidy with image cytometry for detecting dysplasia and carcinoma in oral potentially malignant disorders: A prospective diagnostic study. *Cancer Medicine*, *9*(17), 6411–6420. https://doi.org/10.1002/cam4.3293

Li, Chenxi, Zhang, Q., Sun, K., Jia, H., Shen, X., Tang, G., … Shi, L. (2022). Autofluorescence imaging as a noninvasive tool of risk stratification for malignant transformation of oral leukoplakia: A follow-up cohort study. *Oral Oncology*, *130*, 105941. https://doi.org/10.1016/j.oraloncology.2022.105941

Li, Chun lei, Ren, X. meng, Fang, X., Luo, H. yan, & Hua, H. (2023). Clinical, histological and direct immunofluorescence features in oral mucosal patches striae diseases with malignant potential. *Journal of Dental Sciences*, *18*(3), 1008–1015. https://doi.org/10.1016/j.jds.2022.11.028

Li, H., Li, W., Gao, Y., Li, J., Zeng, X., Lin, J., … Ling, T. (2024). DNA Image Cytometry for Screening the Carcinogenetic Risk of Oral Potential Malignant Disorders. *Journal of Cancer*, *15*(5), 1182–1190. https://doi.org/10.7150/jca.91048

Li, J., Liu, Y., Zhang, H., & Hua, H. (2020). Association between hyperglycemia and the malignant transformation of oral leukoplakia in China. *Oral Diseases*, *26*(7), 1402–1413. https://doi.org/10.1111/odi.13372

Li, X., Liu, L., Zhang, J., Ma, M., Sun, L., Li, X., … Li, T. (2021). Improvement in the risk assessment of oral leukoplakia through morphology-related copy number analysis. *Science China Life Sciences*, *64*(9), 1379–1391. https://doi.org/10.1007/s11427-021-1965-x

Lin, S. C., Liu, C. J., Ko, S. Y., Chang, H. C., Liu, T. Y., & Chang, K. W. (2005). Copy number amplification of 3q26-27 oncogenes in microdissected oral squamous cell carcinoma and oral brushed samples from areca chewers. *Journal of Pathology*, *206*(4), 417–422. https://doi.org/10.1002/path.1790

Lin, Y. C., Huang, H. I., Wang, L. H., Tsai, C. C., Lung, O., Dai, C. Y., … Chen, C. H. (2008). Polymorphisms of COX-2 -765G > C and p53 codon 72 and risks of oral squamous cell carcinoma in a Taiwan population. *Oral Oncology*, *44*(8), 798–804. https://doi.org/10.1016/j.oraloncology.2007.10.006

LIND, P. O. (1987). Malignant transformation in oral leukoplakia. *European Journal of Oral Sciences*, *95*(6), 449–455. https://doi.org/10.1111/j.1600-0722.1987.tb01959.x

Liu, C. J., Lin, S. C., Yang, C. C., Cheng, H. W., & Chang, K. W. (2012). Exploiting salivary miR-31 as a clinical biomarker of oral squamous cell carcinoma. *Head and Neck*, *34*(2), 219–224. https://doi.org/10.1002/hed.21713

Liu, Wei, Shi, L. J., Wu, L., Feng, J. Q., Yang, X., Li, J., … Zhang, C. P. (2012). Oral cancer development in patients with leukoplakia - clinicopathological factors affecting outcome. *PLoS ONE*, *7*(4), e34773. https://doi.org/10.1371/journal.pone.0034773

Liu, Wei, Wang, Y. F., Zhou, H. W., Shi, P., Zhou, Z. T., & Tang, G. Y. (2010). Malignant transformation of oral leukoplakia: A retrospective cohort study of 218 Chinese patients. *BMC Cancer*, *10*, 685. https://doi.org/10.1186/1471-2407-10-685

Liu, Wei, Yao, Y., Shi, L., Tang, G., & Wu, L. (2021). A novel lncRNA LOLA1 may predict malignant progression and promote migration, invasion, and EMT of oral leukoplakia via the AKT/GSK-3β pathway. *Journal of Cellular Biochemistry*, *122*(10), 1302–1312. https://doi.org/10.1002/jcb.29951

Liu, Weiwei, Zheng, W., Xie, J., Zhang, B., Ma, W., & Chen, X. (2011). Identification of genes related to carcinogenesis of oral leukoplakia by oligo cancer microarray analysis. *Oncology Reports*, *26*(1), 265–274. https://doi.org/10.3892/or.2011.1281

Liu, Y., Gao, Y., Chen, X. J., & Hua, H. (2019). DNA cytometry of exfoliated cells in the diagnosis of oral potential malignant disorders. *Beijing Da Xue Xue Bao. Yi Xue Ban = Journal of Peking University. Health Sciences*, *51*(1), 16–20. https://doi.org/10.19723/j.issn.1671-167X.2019.01.004

Liu, Yao, Li, J., Liu, X., Liu, X., Khawar, W., Zhang, X., … Sun, Z. (2015). Quantitative risk stratification of oral leukoplakia with exfoliative cytology. *PLoS ONE*, *10*(5), e0126760. https://doi.org/10.1371/journal.pone.0126760

Liu, Yao, Li, Y., Fu, Y., Liu, T., Liu, X., Zhang, X., … Sun, Z. (2017). Quantitative prediction of oral cancer risk in patients with oral leukoplakia. *Oncotarget*, *8*(28), 46057–46064. https://doi.org/10.18632/oncotarget.17550

Lodi, G., Franchini, R., Bez, C., Sardella, A., Moneghini, L., Pellegrini, C., … Carrassi, A. (2010). Detection of survivin mRNA in healthy oral mucosa, oral leucoplakia and oral cancer. *Oral Diseases*, *16*(1), 61–67. https://doi.org/10.1111/j.1601-0825.2009.01614.x

Lohe, V. K., Degwekar, S. S., Bhowate, R. R., Kadu, R. P., & Dangore, S. B. (2010). Evaluation of correlation of serum lipid profile in patients with oral cancer and precancer and its association with tobacco abuse. *Journal of Oral Pathology and Medicine*, *39*(2), 141–148. https://doi.org/10.1111/j.1600-0714.2009.00828.x

Lombardi, N., Arduino, P. G., Lampiano, M., Gambino, A., Broccoletti, R., Varoni, E. M., & Lodi, G. (2024). Surgical treatment compared with “wait and see” in patients affected by oral leukoplakia to prevent oral cancer: Preliminary data from a multicenter randomized controlled trial. *Oral Diseases*. https://doi.org/10.1111/odi.15058

López-Jornet, P., Olmo-Monedero, A., Peres-Rubio, C., Pons-Fuster, E., & Tvarijonaviciute, A. (2023). Preliminary Evaluation Salivary Biomarkers in Patients with Oral Potentially Malignant Disorders (OPMD): A Case–Control Study. *Cancers*, *15*(21). https://doi.org/10.3390/cancers15215256

López, M., Aguirre, J. M., Cuevas, N., Anzola, M., Videgain, J., Aguirregaviria, J., … Martínez de Pancorbo, M. (2004). Use of cytological specimens for p53 gene alteration detection in oral squamous cell carcinoma risk patients. *Clinical Oncology*, *16*(5), 366–370. https://doi.org/10.1016/j.clon.2004.03.011

Magdum, D. B., Kulkarni, N. A., Kavle, P. G., Paraye, S., Pohankar, P. S., & Giram, A. V. (2024). Salivary Neutrophil-to-Lymphocyte Ratio as a Prognostic Predictor of Oral Premalignant and Malignant Disorders: A Prospective Study. *Cureus*, *16*(3), e56273. https://doi.org/10.7759/cureus.56273

Maheswari, H., Eswaran, M. A., Srividhya, S., Malavika, R., Prabhu, R., & Geetha, K. R. (2014). Estimation of circulating immune complexes in patients with oral leukoplakia and oral submucous fibrosis: A case control study. *Journal of Clinical and Diagnostic Research*, *8*(1), 224–227. https://doi.org/10.7860/JCDR/2014/7095.3967

Mahimkar, M. B., Samant, T. A., Kannan, S., & Patil, T. (2010). Influence of genetic polymorphisms on frequency of micronucleated buccal epithelial cells in leukoplakia patients. *Oral Oncology*, *46*(10), 761–766. https://doi.org/10.1016/j.oraloncology.2010.08.009

Maia, H. C. de M., Pinto, N. A. S., Pereira, J. D. S., de Medeiros, A. M. C., da Silveira, É. J. D., & Miguel, M. C. da C. (2016). Potentially malignant oral lesions: clinicopathological correlations. *Einstein (Sao Paulo, Brazil)*, *14*(1), 35–40. https://doi.org/10.1590/S1679-45082016AO3578

Majumder, M., Ghosh, S., & Roy, B. (2012). Association between polymorphisms at N-acetyltransferase 1 (NAT1) & risk of oral leukoplakia & cancer. *Indian Journal of Medical Research*, *136*(4), 605–613. Retrieved from https://pubmed.ncbi.nlm.nih.gov/23168701/

Majumder, M., Sikdar, N., Paul, R. R., & Roy, B. (2005). Increased risk of oral leukoplakia and cancer among mixed tobacco users carrying XRCC1 variant haplotypes and cancer among smokers carrying two risk genotypes: One on each of two loci, GSTM3 and XRCC1 (codon 280). *Cancer Epidemiology Biomarkers and Prevention*, *14*(9), 2106–2112. https://doi.org/10.1158/1055-9965.EPI-05-0108

Mampilly, M., Ravindran, N., Parambil, M., Nilesh, K., Jayagopalan, P., & Dhamali, D. (2021). Assessment of serum selenium and ceruloplasmin in potentially malignant disorders and oral cancer. *Journal of Pharmacy and Bioallied Sciences*, *13*(6), S989–S992. https://doi.org/10.4103/jpbs.jpbs_380_21

Manzone, H., Billings, P. C., Odell, C. S., Horan, A. M., Kennedy, A. R., Cummings, W. N., … Meyskens, F. L. (1995). Levels of Proteolytic Activities as Intermediate Marker Endpoints in Oral Carcinogenesis. *Cancer Epidemiology Biomarkers and Prevention*, *4*(5), 521–527. Retrieved from https://pubmed.ncbi.nlm.nih.gov/7549809/

Mao, L., El-Naggar, A. K., Fan, Y. H., Lee, J. S., Lippman, S. M., Kayser, S., … Hong, W. K. (1996). Telomerase activity in head and neck squamous cell carcinoma and adjacent tissues. *Cancer Research*, *56*(24), 5600–5604. Retrieved from https://pubmed.ncbi.nlm.nih.gov/8758922/

Mao, L., Lee, J. S., Fan, Y. H., Ro, J. Y., Batsakis, J. G., Lippman, S., … Hong, W. K. (1996). Frequent microsatellite alterations at chromosomes 9p21 and 3p14 in oral premalignant lesions and their value in cancer risk assessment. *Nature Medicine*, *2*(6), 682–685. https://doi.org/10.1038/nm0696-682

Maraki, D., Becker, J., & Boecking, A. (2004). Cytologic and DNA-cytometric very early diagnosis of oral cancer. *Journal of Oral Pathology and Medicine*, *33*(7), 398–404. https://doi.org/10.1111/j.1600-0714.2004.0235.x

Martin, P. (2006). DNA cytometry of oral leukoplakia and oral lichen planus. *Medicina Oral, Patología Oral y Cirugía Bucal.*, *11*(2), E9-14. Retrieved from https://pubmed.ncbi.nlm.nih.gov/15800471/

Martínez, C., Hernández, M., Martínez, B., & Adorno, D. (2016). Frecuencia de displasia epitelial y carcinoma escamoso en mucosa oral y orofaríngea en Chile, entre los años 1990 y 2009. *Revista Medica de Chile*, *144*(2), 169–174. https://doi.org/10.4067/S0034-98872016000200004

Mashberg, A., Merletti, F., Boffetta, P., Gandolfo, S., Ozzello, F., Fracchia, F., & Terracini, B. (1989). Appearance, site of occurrence, and physical and clinical characteristics of oral carcinoma in Torino, Italy. *Cancer*, *63*(12), 2522–2527. https://doi.org/10.1002/1097-0142(19890615)63:12<2522::AID-CNCR2820631227>3.0.CO;2-X

Mashhadiabbas, F., & Fayazi-Boroujeni, M. (2017). Correlation of vascularization and inflammation with severity of oral Leukoplakia. *Iranian Journal of Pathology*, *12*(3), 225–230. https://doi.org/10.30699/ijp.2017.25044

Mathew, A., Mody, R. N., Patait, M. R., Razooki, A. A., Varghese, N. T., & Saraf, K. (2011). Prevalence and relationship of human papilloma virus type 16 and type 18 with oral squamous cell carcinoma and oral leukoplakia in fresh scrappings: A PCR study. *Indian Journal of Medical Sciences*, *65*(5), 212–221. https://doi.org/10.4103/0019-5359.106612

Mazumder, S., Basu, B., Ray, J. G., & Chatterjee, R. (2023). MiRNAs as non-invasive biomarkers in the serum of Oral Squamous Cell Carcinoma (OSCC) and Oral Potentially Malignant Disorder (OPMD) patients. *Archives of Oral Biology*, *147*, 105627. https://doi.org/10.1016/j.archoralbio.2023.105627

Mcguirt, W. F. (1983). Snuff Dipper’s Carcinoma. *Archives of Otolaryngology*, *109*(11), 757–760. https://doi.org/10.1001/archotol.1983.00800250051012

Mehta, F. S., Sahiar, B. E., Daftary, D. K., Gupta, P. C., & Pindborg, J. J. (1972). A correlative histocytological study of carcinoma and epithelial atypia of the palate among indian reverse smokers. *British Journal of Cancer*, *26*(3), 230–233. https://doi.org/10.1038/bjc.1972.31

Mehta, R., Gurudath, S., Dayansoor, S., Pai, A., & Ganapathy, K. S. (2014). Serum lipid profile in patients with oral cancer and oral precancerous conditions. *Dental Research Journal*, *11*(3), 345–350. https://doi.org/10.4103/1735-3327.135889

Mellerup, E., Moeller, G. L., Mondal, P., & Roychoudhury, S. (2015). Combinations of genetic data in a study of oral cancer. *Genes and Cancer*, *6*(9–10), 422–427. https://doi.org/10.18632/genesandcancer.79

Mello, F. W., Melo, G., Meurer, M. I., & Rivero, E. R. C. (2018). Intraoral Potentially Malignant Disorders in a Brazilian Oral Pathology Service: Epidemiological, Clinical, and Histopathological Findings. *Journal of Oncology*, *2018*, 2325808. https://doi.org/10.1155/2018/2325808

Metgud, R., Gupta, K., Prasad, U., & Gupta, J. (2015). Cytomorphometric analysis of oral submucous fibrosis and leukoplakia using methyl green-pyronin Y, Feulgen staining and exfoliative brush cytology. *Biotechnic and Histochemistry*, *90*(1), 8–13. https://doi.org/10.3109/10520295.2014.919025

Metgud, R., & Patel, S. (2014). Serum and salivary levels of albumin as diagnostic tools for oral pre-malignancy and oral malignancy. *Biotechnic and Histochemistry*, *89*(1), 8–13. https://doi.org/10.3109/10520295.2013.793394

Metgud, Rashmi, & Bajaj, S. (2014). Evaluation of salivary and serum lipid peroxidation, and glutathione in oral leukoplakia and oral squamous cell carcinoma. *Journal of Oral Science*, *56*(2), 135–142. https://doi.org/10.2334/josnusd.56.135

Michailidou, E., Tzimagiorgis, G., Chatzopoulou, F., Vahtsevanos, K., Antoniadis, K., Kouidou, S., … Antoniades, D. (2016). Salivary mRNA markers having the potential to detect oral squamous cell carcinoma segregated from oral leukoplakia with dysplasia. *Cancer Epidemiology*, *43*, 112–118. https://doi.org/10.1016/j.canep.2016.04.011

Mighell, A. J., Thompson, J., Hume, W. J., Markham, A. F., & Robinson, P. A. (1997). RT-PCR investigation of fibronectin mRA isoforms in malignant, normal and reactive oral mucosa. *European Journal of Cancer Part B: Oral Oncology*, *33*(3), 155–162. https://doi.org/10.1016/S0964-1955(96)00074-7

Miller, W. A., Wuertz, B. R., & Ondrey, F. G. (2018). PPARγ-Mediated p21 Induction in Aerodigestive Preneoplastic Cell Lines. *Annals of Otology, Rhinology and Laryngology*, *127*(10), 677–686. https://doi.org/10.1177/0003489418787833

Mishra, M., Mohanty, J., Sengupta, S., & Tripathy, S. (2005). Epidemiological and clinicopathological study of oral leukoplakia. *Indian Journal of Dermatology, Venereology and Leprology*, *71*(3), 161–165. https://doi.org/10.4103/0378-6323.16229

Misra, C., Majumder, M., Bajaj, S., Ghosh, S., Roy, B., & Roychoudhury, S. (2009). Polymorphisms at p53, p73, and MDM2 loci modulate the risk of tobacco associated leukoplakia and oral cancer. *Molecular Carcinogenesis*, *48*(9), 790–800. https://doi.org/10.1002/mc.20523

Mitra, S., Banerjee, S., Misra, C., Singh, R. K., Roy, A., Sengupta, A., … Roychoudhury, S. (2007). Interplay between human papilloma virus infection and p53 gene alterations in head and neck squamous cell carcinoma of an Indian patient population. *Journal of Clinical Pathology*, *60*(9), 1040–1047. https://doi.org/10.1136/jcp.2005.034835

Mitra, Sayan, Sikdar, N., Misra, C., Gupta, S., Paul, R. R., Roy, B., … Roychoudhury, S. (2005). Risk assessment of p53 genotypes and haplotypes in tobacco-associated leukoplakia and oral cancer patients from eastern India. *International Journal of Cancer*, *117*(5), 786–793. https://doi.org/10.1002/ijc.21263

Miyoshi, Y., Tsukinoki, K., Imaizumi, T., Yamada, Y., Ishizaki, T., Watanabe, Y., … Kubota, Y. (1999). Telomerase activity in oral cancer. *Oral Oncology*, *35*(3), 283–289. https://doi.org/10.1016/S1368-8375(98)00117-1

Mohamed Anser, S., & Aswath, N. (2014). Comparison of the carcinogenic potential of smokeless tobacco and smoked tobacco by quantifying the excretion of nicotine metabolite NNAL in patients with oral leukoplakia. *Indian Journal of Clinical Biochemistry*, *29*(2), 246–249. https://doi.org/10.1007/s12291-013-0363-7

Morandi, L., Gissi, D., Tarsitano, A., Asioli, S., Gabusi, A., Marchetti, C., … Foschini, M. P. (2017). CpG location and methylation level are crucial factors for the early detection of oral squamous cell carcinoma in brushing samples using bisulfite sequencing of a 13-gene panel. *Clinical Epigenetics*, *9*(1), 85. https://doi.org/10.1186/s13148-017-0386-7

More, C. B., Shah, P. H., & Venkatesh, R. (2017). Estimation of serum protein in oral potentially malignant disorders and oral malignancy – A cross-sectional study. *Journal of Clinical and Diagnostic Research*, *11*(2), ZC17–ZC19. https://doi.org/10.7860/JCDR/2017/20815.9254

Moro, A., Di Nardo, F., Boniello, R., Marianetti, T. M., Cervelli, D., Gasparini, G., & Pelo, S. (2010). Autofluorescence and early detection of mucosal lesions in patients at risk for oral cancer. *Journal of Craniofacial Surgery*, *21*(6), 1899–1903. https://doi.org/10.1097/SCS.0b013e3181f4afb4

Mukherjee, S., Ray, J., & Chaudhuri, K. (2011). Evaluation of DNA damage in oral precancerous and squamous cell carcinoma patients by single cell gel electrophoresis. *Indian Journal of Dental Research*, *22*(5), 735–736. https://doi.org/10.4103/0970-9290.93475

Murali, P. S., Somasundaram, R., Chiplunkar, S. V., Fakih, A. R., Rao, R. S., & Gangal, S. G. (1989). Monocyte/macrophage functions in patients with squamous cell carcinoma of the oral cavity. *Journal of Oral Pathology & Medicine*, *18*(10), 539–543. https://doi.org/10.1111/j.1600-0714.1989.tb01550.x

Murali, P. S., Somasundaram, R., Rao, R. S., Fakih, A. R., & Gangal, S. G. (1989). lnterleukin‐2 mediated regulation of mitogen‐activated T cell reactivity from different lymphoid sources in patients with squamous cell carcinoma of the oral cavity. *Journal of Oral Pathology & Medicine*, *18*(6), 327–332. https://doi.org/10.1111/j.1600-0714.1989.tb01561.x

Mustafa, M. B., Hassan, M. O., Alhussein, A., Mamoun, E., El Sheikh, M., & Suleiman, A. M. (2019). Oral leukoplakia in the Sudan: clinicopathological features and risk factors. *International Dental Journal*, *69*(6), 428–435. https://doi.org/10.1111/idj.12509

Nanayakkara, P. G. C. L., Dissanayaka, W. L., Nanayakkara, B. G., Amaratunga, E. A. P. D., & Tilakaratne, W. M. (2016). Comparison of spatula and cytobrush cytological techniques in early detection of oral malignant and premalignant lesions: A prospective and blinded study. *Journal of Oral Pathology and Medicine*, *45*(4), 268–274. https://doi.org/10.1111/jop.12357

Nayyar, A. S. (2012). Novel biochemical markers: Early detection and prevention of malignant transformation a pilot study. *Acta Medica Iranica*, *50*(9), 597–602. Retrieved from https://pubmed.ncbi.nlm.nih.gov/23165808/

Nayyar, A. S., & Khan, M. (2012). In search of malignant transformation: A pilot study. *Journal of Cancer Research and Therapeutics*, *8*(2), 277–281. https://doi.org/10.4103/0973-1482.98987

Nayyar, A. S., Khan, M., Vijayalakshmi, K. R., Suman, B., Gayitri, H. C., & Anitha, M. (2012). Serum total protein, albumin and advanced oxidation protein products (AOPP) - implications in oral squamous cell carcinoma. *Malaysian Journal of Pathology*, *34*(1), 47–52. Retrieved from https://pubmed.ncbi.nlm.nih.gov/22870598/

Neerupakam, M., Alaparthi, R. K., Sathish, S., Katta, S. A., Polisetty, N., & Damera, S. (2014). Alterations in plasma lipid profile patterns in oral cancer. *Journal of Indian Academy of Oral Medicine and Radiology*, *26*(3), 274–278. https://doi.org/10.4103/0972-1363.145004

Nigam, K., Gupta, S., Gupta, O. P., Srivastav, R. K., Singh, S. P., & Sanyal, S. (2021). Alteration of the risk of pre-oral cancer and cancer in North Indian population by NAT1 and NAT2 polymorphisms genotypes and haplotypes. *European Archives of Oto-Rhino-Laryngology*, *278*(10), 4081–4089. https://doi.org/10.1007/s00405-021-06774-w

Nigam, K., Singh, N., Yadav, S. K., & Sanyal, S. (2023). The Taq 1 polymorphism of Vitamin D receptor gene is associated with oral cancer and preoral cancer in North Indian population. *Journal of Cancer Research and Therapeutics*, *19*(2), 403–407. https://doi.org/10.4103/jcrt.jcrt_109_22

Nigam, K., Yadav, S. K., Samadi, F. M., Bhatt, M. L. B., Gupta, S., & Sanyal, S. (2019). Risk modulation of oral pre cancer and cancer with polymorphisms in XPD and XPG genes in north Indian population. *Asian Pacific Journal of Cancer Prevention*, *20*(8), 2397–2403. https://doi.org/10.31557/APJCP.2019.20.8.2397

Nitsche, M., Koy, S., Mörz, M., Koch, R., & Eckelt, U. (2007). Untersuchung der Tumorsuppressoren p16INK4a und p14ARF in Mundschleimhautleukoplakien. *Mund - Kiefer - Und Gesichtschirurgie*, *11*(6), 317–326. https://doi.org/10.1007/s10006-007-0086-0

Nivia, M., Sunil, S. N., Rathy, R., & Anilkumar, T. V. (2015). Comparative cytomorphometric analysis of oral mucosal cells in normal, tobacco users, oral leukoplakia and oral squamous cell carcinoma. *Journal of Cytology*, *32*(4), 253–260. https://doi.org/10.4103/0970-9371.171241

Odani, T., Ito, D., Li, M. H., Kawamata, A. I., Isobe, T., Iwase, M., & Nagumo, M. (2006). Gene expression profiles of oral leukoplakia and carcinoma: Genome-wide comparison analysis using oligonucleotide microarray technology. *International Journal of Oncology*, *28*(3), 619–624. https://doi.org/10.3892/ijo.28.3.619

Olinici, D., Cotrutz, C. E., Mihali, C. V., Grecu, V. B., Botez, E. A., Stoica, L., … Dimitriu, D. C. (2018). The ultrastructural features of the premalignant oral lesions. *Romanian Journal of Morphology and Embryology*, *59*(1), 243–248. Retrieved from https://pubmed.ncbi.nlm.nih.gov/29940634/

Onofre, M. A., Sposto, M. R., Navarro, C. M., Motta, M. E. S. F. M., Turatti, E., & Almeida, R. T. (1997). Potentially malignant epithelial oral lesions: Discrepancies between clinical and histological diagnosis. *Oral Diseases*, *3*(3), 148–152. https://doi.org/10.1111/j.1601-0825.1997.tb00026.x

Oshin, M., Kulkarni, P. G., D, S. P. R., Deepthi, G., S, K. S., & S, J. K. (2024). Salivary and Serum Interleukin-6: A Credible Marker for Predicting Oral Leukoplakia and Oral Squamous Cell Carcinoma by Enzyme-Linked Immunosorbent Assay (ELISA). *Cureus*, *16*(4), e59113. https://doi.org/10.7759/cureus.59113

Pal, J., Rajput, Y., Shrivastava, S., Gahine, R., Mungutwar, V., Barardiya, T., … Shammas, M. A. (2022). A standalone approach to utilize telomere length measurement as a surveillance tool in oral leukoplakia. *Molecular Oncology*, *16*(8), 1650–1660. https://doi.org/10.1002/1878-0261.13133

Pandey, M., Prakash, O., Santhi, W. S., Soumithran, C. S., & Pillai, R. M. (2008). Overexpression of COX-2 gene in oral cancer is independent of stage of disease and degree of differentiation. *International Journal of Oral and Maxillofacial Surgery*, *37*(4), 379–383. https://doi.org/10.1016/j.ijom.2008.01.004

Pandey, R., Mehrotra, D., Mahdi, A. A., Sarin, R., & Kowtal, P. (2014). Additional cytosine inside mitochondrial C-tract D-loop as a progression risk factor in oral precancer cases. *Journal of Oral Biology and Craniofacial Research*, *4*(1), 3–7. https://doi.org/10.1016/j.jobcr.2014.02.003

Panneer Selvam, N., & Sadaksharam, J. (2015). Salivary interleukin-6 in the detection of oral cancer and precancer. *Asia-Pacific Journal of Clinical Oncology*, *11*(3), 236–241. https://doi.org/10.1111/ajco.12330

Patel, S., & Metgud, R. (2015). Estimation of salivary lactate dehydrogenase in oral leukoplakia and oral squamous cell carcinoma: A biochemical study. *Journal of Cancer Research and Therapeutics*, *11*(1), 119–123. https://doi.org/10.4103/0973-1482.138193

Patel, U., Shah, R., Patel, A., Shah, S., Patel, D., & Patel, A. (2020). Effect of tobacco in human oral leukoplakia: a cytomorphometric analysis. *Medicine and Pharmacy Reports*, *93*(3), 273–279. https://doi.org/10.15386/mpr-1439

Patil, M. B., Lavanya, T., Meena Kumari, C., Shetty, S. R., Gufran, K., Viswanath, V., … Nayyar, A. S. (2021). Serum ceruloplasmin as cancer marker in oral pre-cancers and cancers. *Journal of Carcinogenesis*, *20*(1), 15. https://doi.org/10.4103/jcar.jcar_10_21

Patil, S., Rao, R. S., Sanketh, D. S., & Warnakulasuriya, S. (2015). Lichenoid dysplasia revisited - evidence from a review of Indian archives. *Journal of Oral Pathology and Medicine*, *44*(7), 507–514. https://doi.org/10.1111/jop.12258

Pazhani, J., Chanthu, K., Jayaraman, S., & Varun, B. R. (2023). Evaluation of salivary MMP‑9 in oral squamous cell carcinoma and oral leukoplakia using ELISA. *Journal of Oral and Maxillofacial Pathology*, *27*(4), 649–654. https://doi.org/10.4103/jomfp.jomfp_426_23

Peng, Y. S., Peng, Y. M., McGee, D. L., & Alberts, D. S. (1994). Carotenoids, tocopherols, and retinoids in human buccal mucosal cells: Intra- and interindividual variability and storage stability. *American Journal of Clinical Nutrition*, *59*(3), 636–643. https://doi.org/10.1093/ajcn/59.3.636

Pentenero, M., Castagnola, P., Castillo, F. V., Isaevska, E., Sutera, S., & Gandolfo, S. (2023). Predictors of malignant transformation in oral leukoplakia and proliferative verrucous leukoplakia: An observational prospective study including the DNA ploidy status. *Head and Neck*, *45*(10), 2589–2604. https://doi.org/10.1002/hed.27483

Pentenero, M., Donadini, A., di Nallo, E., Maffei, M., Marino, R., Familiari, U., … Giaretti, W. (2012). Field effect in oral precancer as assessed by DNA flow cytometry and array-CGH. *Journal of Oral Pathology and Medicine*, *41*(2), 119–123. https://doi.org/10.1111/j.1600-0714.2011.01085.x

Pereira, J. D. S., Carvalho, M. D. V., Henriques, Á. C. G., De Queiroz Camara, T. H., Miguel, M. C. D. C., & Freitas, R. D. A. (2011). Epidemiology and correlation of the clinicopathological features in oral epithelial dysplasia: Analysis of 173 cases. *Annals of Diagnostic Pathology*, *15*(2), 98–102. https://doi.org/10.1016/j.anndiagpath.2010.08.008

Pereira, N. D. S., & Pinheiro, T. N. (2019). Histomorphometric Comparative Analysis between Oral Dysplastic Potentially Malignant Disorders and Oral Squamous Cell Carcinoma. *European Journal of Dentistry*, *13*(1), 1–4. https://doi.org/10.1055/s-0039-1688734

Philipone, E., Yoon, A. J., Wang, S., Shen, J., Ko, Y. C. K., Sink, J. M., … Santella, R. M. (2016). MicroRNAs-208b-3p, 204-5p, 129-2-3p and 3065-5p as predictive markers of oral leukoplakia that progress to cancer. *American Journal of Cancer Research*, *6*(7), 1537–1546. Retrieved from https://pubmed.ncbi.nlm.nih.gov/27508095/

Phookan, J., & Saikia, K. P. (1998). A clinicopathological study of the pre-malignant conditions of oral cavity. *Indian Journal of Otolaryngology and Head and Neck Surgery*, *50*(3), 246–249. https://doi.org/10.1007/BF03007000

Pindborg, J. J., Kiaer, J., Gupta, P. C., & Chawla, T. N. (1967). Studies in oral leukoplakias. Prevalence of leukoplakia among 10,000 persons in Lucknow, India, with special reference to use of tobacco and betel nut. *Bulletin of the World Health Organization*, *37*(1), 109–116. Retrieved from https://pubmed.ncbi.nlm.nih.gov/5300044/

Pindborg, J. J., Odont D. Barmes, D., & Roed‐Petersen, B. (1968). Epidemiology and histology of oral leukoplakia and leukoedema among papuans and New Guineans. *Cancer*, *22*(2), 379–384. https://doi.org/10.1002/1097-0142(196808)22:2<379::AID-CNCR2820220215>3.0.CO;2-A

Pindborg, J. J., Reibel, J., & Holmstrup, P. (1985). Subjectivity in evaluating oral epithelial dysplasia, carcinoma in situ and initial carcinoma. *Journal of Oral Pathology & Medicine*, *14*(9), 698–708. https://doi.org/10.1111/j.1600-0714.1985.tb00549.x

Pindborg, J. J., Reibel, J., Roed‐Petersen, B., & Mehta, F. S. (1980). Tobacco‐induced changes in oral leukoplakic epithelium. *Cancer*, *45*(9), 2330–2336. https://doi.org/10.1002/1097-0142(19800501)45:9<2330::AID-CNCR2820450916>3.0.CO;2-R

Pindborg, J. J., Renstrup, G., Poulsen, H. E., & Silverman, S. (1963). Studies in oral leukoplakias. V. Clinical and histologic signs of malignancy. *Acta Odontologica Scandinavica*, *21*(5), 407–414. https://doi.org/10.3109/00016356309028203

Pindborg, Jens J., Daftary, D. K., & Mehta, F. S. (1977). A follow-up study of sixty-one oral dysplastic precancerous lesions in Indian villagers. *Oral Surgery, Oral Medicine, Oral Pathology*, *43*(3), 383–390. https://doi.org/10.1016/0030-4220(77)90325-5

Pires, F. R., Barreto, M. E. Z., Nunes, J. G. R., Car-Neiro, N. S., de Azevedo, A. B., & Dos Santos, T. C. R. B. (2020). Oral potentially malignant disorders: Clinical-pathological study of 684 cases diagnosed in a brazilian population. *Medicina Oral Patologia Oral y Cirugia Bucal*, *25*(1), e84–e88. https://doi.org/10.4317/medoral.23197

Piscopo, M., Campisi, G., Colella, G., Bilancione, M., Caccamo, S., Di Liberto, C., … Fucci, L. (2006). H3 and H3.3 histone mRNA amounts and ratio in oral squamous cell carcinoma and leukoplakia. *Oral Diseases*, *12*(2), 130–136. https://doi.org/10.1111/j.1601-0825.2005.01169.x

Plndborg, J. J., Roed‐petersen, B., & Renstrup, G. (1972). Role of smoking in floor of the mouth leukoplakias. *Journal of Oral Pathology & Medicine*, *1*(1), 22–29. https://doi.org/10.1111/j.1600-0714.1972.tb02119.x

Poate, T. W. J., & Warnakulasuriya, S. (2006). Effective management of smoking in an oral dysplasia clinic in London. *Oral Diseases*, *12*(1), 22–26. https://doi.org/10.1111/j.1601-0825.2005.01146.x

Pontes, C. C., Chikte, U., Kimmie-dhansay, F., Erasmus, R. T., Kengne, A. P., & Matsha, T. E. (2020). Prevalence of oral mucosal lesions and relation to serum cotinine levels—findings from a cross- sectional study in South Africa. *International Journal of Environmental Research and Public Health*, *17*(3). https://doi.org/10.3390/ijerph17031065

Porto, U. N., Laureano, N. K., Dos Santos, N. S., Rodrigues, A. Z., Ferri, C. A., de Lima, T. B., … Visioli, F. (2024). Leukoplakia and erythroplakia in youngers versus older individuals: a clinicopathological retrospective study. *Medicina Oral Patologia Oral y Cirugia Bucal*, *29*(5), 665–672. https://doi.org/10.4317/medoral.26659

Prabhu Venkatesh, D., Ramalingam, K., Ramani, P., Krishnan, M., & Kumar Vadivel, J. (2024). Epidemiological Trends and Clinicopathological Characteristics of Oral Leukoplakia: A Retrospective Analysis From a Single Institution in Chennai, Tamil Nadu, India. *Cureus*, *16*(6), e61590. https://doi.org/10.7759/cureus.61590

Prime, S. S., Rosser, T. J., Malamos, D., Shepherd, J. P., & Scully, C. (1985). The use of the lectin Ulex europeus to study epithelial cell differentiation in neoplastic and non‐neoplastic oral white lesions. *The Journal of Pathology*, *147*(3), 173–179. https://doi.org/10.1002/path.1711470305

Punyani, S. R., & Sathawane, R. S. (2013). Salivary level of interleukin-8 in oral precancer and oral squamous cell carcinoma. *Clinical Oral Investigations*, *17*(2), 517–524. https://doi.org/10.1007/s00784-012-0723-3

Qian, L., Qian, B., Xu, J., Yang, J., Wu, G., Zhao, Y., … Li, H. (2023). Clinical relevance of serum lipids in the carcinogenesis of oral squamous cell carcinoma. *BMC Oral Health*, *23*(1), 200. https://doi.org/10.1186/s12903-023-02859-6

Qiao, B. S. (1993). Quantitative studies on DNA content of normal mucosa, leukoplakia and carcinoma of the oral cavity. *Zhonghua Kou Qiang Yi Xue Za Zhi = Zhonghua Kouqiang Yixue Zazhi = Chinese Journal of Stomatology*, *28*(6), 372–375. Retrieved from https://pubmed.ncbi.nlm.nih.gov/8033652/

Ragavendra, T. R., Rammanohar, M., & Sowmya, K. (2010). Morphometric computer-assisted image analysis of oral epithelial cells in normal epithelium and leukoplakia. *Journal of Oral Pathology and Medicine*, *39*(2), 149–154. https://doi.org/10.1111/j.1600-0714.2009.00860.x

Rai, N. P., Anekar, J., Shivaraja Shankara, Y. M., Divakar, D. D., Al Kheraif, A. A., Ramakrishnaiah, R., … Mustafa, S. M. (2015). Comparison of serum fucose levels in leukoplakia and oral cancer patients. *Asian Pacific Journal of Cancer Prevention*, *16*(17), 7497–7500. https://doi.org/10.7314/APJCP.2015.16.17.7497

Rajendran, R., Sugathan, C. K., Remani, P., Ankathil, R., & Vijayakumar, T. (1986). Cell mediated and humoral immune responses in oral submucous fibrosis. *Cancer*, *58*(12), 2628–2631. https://doi.org/10.1002/1097-0142(19861215)58:12<2628::AID-CNCR2820581214>3.0.CO;2-Z

Rajesh, D., Azeem Mohiyuddin, S. M., Moideen Kutty, A. V., & Balakrishna, S. (2017). Prevalence of human papillomavirus in oral squamous cell carcinoma: A rural teaching hospital-based cross-sectional study. *Indian Journal of Cancer*, *54*(3), 498–501. https://doi.org/10.4103/ijc.IJC_272_17

Ramachandran, S., Ramadas, K., Hariharan, R., Rejnish Kumar, R., & Radhakrishna Pillai, M. (2006). Single nucleotide polymorphisms of DNA repair genes XRCC1 and XPD and its molecular mapping in Indian oral cancer. *Oral Oncology*, *42*(4), 35

Symeonides, A. (1969). The histopathological aspect of leukoplakia and its relation to oral carcinoma. *Stomatologia*, *26*(1), 5–12. Retrieved from http://www.embase.com/search/results?subaction=viewrecord&from=export&id=L89128807

**Cross-sectional (n = 262):**

Abiko, Y., Suraweera, A. K., Nishimura, M., Arakawa, T., Takuma, T., Mizoguchi, I., & Kaku, T. (2001). Differential expression of human beta-defensin 2 in keratinized and non-keratinized oral epithelial lesions; immunohistochemistry and in situ hybridization. Virchows Archiv, 438(3), 248–253. https://doi.org/10.1007/s004280000303

Acay, R. R., Santos, E. dos, & Machado de Sousa, S. O. (2008). Correlation between c-Jun and human papillomavirus in oral premalignant and malignant lesions. Oral Oncology, 44(7), 698–702. https://doi.org/10.1016/j.oraloncology.2007.09.007

Acay, R., Rezende, N., Fontes, A., Aburad, A., Nunes, F., & Sousa, S. (2008). Human papillomavirus as a risk factor in oral carcinogenesis: A study using in situ hybridization with signal amplification. Oral Microbiology and Immunology, 23(4), 271–274. https://doi.org/10.1111/j.1399-302X.2007.00422.x

Agarwal, A., Kamboj, M., & Shreedhar, B. (2019). “Expression of p16 in oral leukoplakia and oral squamous cell carcinoma and correlation of its expression with individual atypical features.” Journal of Oral Biology and Craniofacial Research, 9(2), 156–160. https://doi.org/10.1016/j.jobcr.2019.03.002

Agarwal, S., Mathur, M., Shukla, N. K., & Ralhan, R. (1998). Expression of cyclin dependent kinase inhibitor p21(waf1/cip1) in premalignant and malignant oral lesions: Relationship with p53 status. Oral Oncology, 34(5), 353–360. https://doi.org/10.1016/S1368-8375(98)00021-9

Agarwal, Sandhya, Mathur, M., Srivastava, A., & Ralhan, R. (1999). MDM2/p53 co-expression in oral premalignant and malignant lesions: Potential prognostic implications. Oral Oncology, 35(2), 209–216. https://doi.org/10.1016/S1368-8375(98)00092-X

Ahire, M. S., D′souza, Z. I., Chettiankandy, T. J., Nagar, S. R., Sinha, A., & Tupkari, J. V. (2021). Demographic study of 366 cases of oral leukoplakia and immunohistochemical analysis-An institutional study. Journal of Oral and Maxillofacial Pathology, 25(3), 478–484. https://doi.org/10.4103/jomfp.jomfp_228_21

Airoldi, M., Negri, L., Pedani, F., Gerbino, G., Gandolfo, S., & Patriarca, E. (1984). The presence of CEA in spinocellular carcinoma of the oral cavity. Bollettino Della Societa Italiana Di Biologia Sperimentale, 60(4), 871–875. Retrieved from https://pubmed.ncbi.nlm.nih.gov/6375692/

Aiswarya, A., Suresh, R., Janardhanan, M., Savithri, V., Aravind, T., & Mathew, L. (2019). An immunohistochemical evaluation of podoplanin expression in oral leukoplakia and oral squamous cell carcinoma to explore its potential to be used as a predictor for malignant transformation. Journal of Oral and Maxillofacial Pathology, 23(1), 159–160. https://doi.org/10.4103/jomfp.JOMFP_272_17

Akhtar, K., Ara, A., Siddiqui, S. A., & Sherwani, R. K. (2016). Transition of immunohistochemical expression of E-cadherin and vimentin from premalignant to malignant lesions of oral cavity and oropharynx. Oman Medical Journal, 31(3), 165–169. https://doi.org/10.5001/omj.2016.33

Akkaloori, A., Saikia, J., Kuppusamy, A., Rana, K., Dashatwar, P. D., & Behura, S. S. (2023). Comparison of the IHC markers CD138 and CD43 in oral leukoplakia: An original research. Journal of Pharmacy and Bioallied Sciences, 15(5), 209–212. https://doi.org/10.4103/jpbs.jpbs_454_22

Al-Dhohrah, T., Mashrah, M., Yao, Z., & Huang, J. (2016). Aberrant DKK3 expression in the oral leukoplakia and oral submucous fibrosis: A comparative immunohistochemical study. European Journal of Histochemistry, 60(2), 155–159. https://doi.org/10.4081/ejh.2016.2629

Ali, A., Langdon, J., Stern, P., & Partridge, M. (2001). The pattern of expression of the 5T4 oncofoetal antigen on normal, dysplastic and malignant oral mucosa. Oral Oncology, 37(1), 57–64. https://doi.org/10.1016/S1368-8375(00)00057-9

Amirchaghmaghi, M., Mohtasham, N., & Mozaffari, P. M. (2012). Comparison of COX2 expression between oral squamous cell carcinoma, leukoplakia and normal mucosa. Journal of Contemporary Dental Practice, 13(2), 205–209. https://doi.org/10.5005/jp-journals-10024-1122

Angelin, D., & Nair, B. (2020). Comparative evaluation of survivin expression in leukoplakia, lichen planus, and oral squamous cell carcinoma: An immunohistochemical study. Journal of Cancer Research and Therapeutics, 16(3), 569–574. https://doi.org/10.4103/jcrt.JCRT_421_19

Arora, K. S., Nayyar, A., Kaur, P., Arora, K. S., Goel, A., & Singh, S. (2018). Evaluation of collagen in leukoplakia, oral submucous fibrosis and oral squamous cell carcinomas using polarizing microscopy and immunohistochemistry. Asian Pacific Journal of Cancer Prevention, 19(4), 1075–1080. https://doi.org/10.22034/APJCP.2018.19.4.1075

Babiuch, K., Kuśnierz-Cabala, B., Kęsek, B., Okoń, K., Darczuk, D., & Chomyszyn-Gajewska, M. (2020). Evaluation of proinflammatory, nf-kappab dependent cytokines: Il-1α, Il-6, Il-8, and TNF-α in tissue specimens and saliva of patients with oral squamous cell carcinoma and oral potentially malignant disorders. Journal of Clinical Medicine, 9(3). https://doi.org/10.3390/jcm9030867

Baddevithana, A. K., Jayasinghe, R. D., Tilakaratne, W. M., Illeperuma, R. P., & Siriwardena, B. S. M. S. (2023). Expression of Human Papillomavirus and the p16 Gene in Oral Potentially Malignant Disorders (OPMD): a Comparative Study With Oral Squamous Cell Carcinoma. Applied Immunohistochemistry and Molecular Morphology, 31(5), 331–338. https://doi.org/10.1097/PAI.0000000000001124

Bajracharya, D., Shrestha, B., Kamath, A., Menon, A., & Radhakrishnan, R. (2014). Immunohistochemical correlation of matrix metalloproteinase-2 and tissue inhibitors of metalloproteinase-2 in tobacco associated epithelial dysplasia. Disease Markers, 2014, 197813. https://doi.org/10.1155/2014/197813

Bascones-Martínez, A., López-Durán, M., Cano-Sánchez, J., Sánchez-Verde, L., Díez-Rodríguez, A., Aguirre-Echebarría, P., … Campo-Trapero, J. (2012). Differences in the expression of five senescence markers in oral cancer, oral leukoplakia and control samples in humans. Oncology Letters, 3(6), 1319–1325. https://doi.org/10.3892/ol.2012.649

Bernardes, V. F., Correa, G. T. B., Loyola, A. M., Cardoso, S. V., De Paula, A. M. B., Cabral, M. M. D. Á., … Gomes, C. C. (2014). STAG2 expression in oral cancer and potentially malignant lesions. Tumor Biology, 35(4), 3641–3645. https://doi.org/10.1007/s13277-013-1482-8

Bhosale, P. G., Cristea, S., Ambatipudi, S., Desai, R. S., Kumar, R., Patil, A., … Mahimkar, M. B. (2017). Chromosomal Alterations and Gene Expression Changes Associated with the Progression of Leukoplakia to Advanced Gingivobuccal Cancer. Translational Oncology, 10(3), 396–409. https://doi.org/10.1016/j.tranon.2017.03.008

Bhosale, P. G., Pandey, M., Desai, R. S., Patil, A., Kane, S., Prabhash, K., & Mahimkar, M. B. (2016). Low prevalence of transcriptionally active human papilloma virus in Indian patients with HNSCC and leukoplakia. Oral Surgery, Oral Medicine, Oral Pathology and Oral Radiology, 122(5), 609-618.e7. https://doi.org/10.1016/j.oooo.2016.06.006

Bienk Dias, K., Pereira Costa Flores, A., Gaiger Oliveira, M., Varvaki Rados, P., & Sant’ana Filho, M. (2017). Predictive value of p63, ki-67, and survivin expression in oral leukoplakia: A tissue microarray study. Microscopy Research and Technique, 80(8), 845–850. https://doi.org/10.1002/jemt.22872

Bortoluzzi, M. C., Yurgel, L. S., Dekker, N. P., Jordan, R. C. K., & Regezi, J. A. (2004). Assessment of p63 expression in oral squamous cell carcinomas and dysplasias. Oral Surgery, Oral Medicine, Oral Pathology, Oral Radiology and Endodontology, 98(6), 698–704. https://doi.org/10.1016/j.tripleo.2004.04.001

Buajeeb, W., Poomsawat, S., Punyasingh, J., & Sanguansin, S. (2009). Expression of p16 in oral cancer and premalignant lesions. Journal of Oral Pathology and Medicine, 38(1), 104–108. https://doi.org/10.1111/j.1600-0714.2008.00710.x

Buryska, S., Patel, K., Wuertz, B., Gaffney, P. M., & Ondrey, F. (2023). Potential Roles of Activin in Head and Neck Squamous Cell Carcinoma Progression and Mortality. Anticancer Research, 43(12), 5299–5310. https://doi.org/10.21873/anticanres.16733

Chakravarti, N., Mathur, M., Bahadur, S., Shukla, N. K., Rochette-Egly, C., & Ralhan, R. (2001). Expression of RARα and RARβ in human oral potentially malignant and neoplastic lesions. International Journal of Cancer, 91(1), 27–31. https://doi.org/10.1002/1097-0215(20010101)91:1<27::AID-IJC1003>3.0.CO;2-K

Chamorro-Petronacci, C. M., Lafuente-Ibanez De Mendoza, I., Suarez-Peñaranda, J. M., Padin-Iruegas, E., Blanco-Carrion, A., Lorenzo-Pouso, A. I., … Pérez-Sayáns, M. (2021). Immunohistochemical Characterization of Bcl-2 in Oral Potentially Malignant Disorders. Applied Immunohistochemistry and Molecular Morphology, 29(9), 706–712. https://doi.org/10.1097/PAI.0000000000000945

Chandak, A. R., Gadbail, A. R. amchandr., Chaudhary, M. S., Chandak, S. A., & Wadhwani, R. (2011). Actual proliferating index in oral squamous cell carcinoma and leukoplakia. Journal of Investigative and Clinical Dentistry, 2(3), 176–183. https://doi.org/10.1111/j.2041-1626.2011.00057.x

Chandran, G. J., Balaram, P., Kannan, S., Pillai, M. R., Nalinakumari, K. R., & Nair, M. K. (1994). Immunohistochemical localization of epidermal growth factor and its receptor in normal, premalignant and malignant oral mucosa. International Journal of Oncology, 4(2), 503–508. https://doi.org/10.3892/ijo.4.2.503

Chattopadhyay, A., Ray, J. G., & Caplan, D. J. (2002). AgNOR count as objective marker for dysplastic features in oral leukoplakia. Journal of Oral Pathology and Medicine, 31(9), 512–517. https://doi.org/10.1034/j.1600-0714.2002.00153.x

Chen, J. Y. F., Chang, Y. L., Yu, Y. C., Chao, C. C., Kao, H. W., Wu, C. T., … Jou, Y. S. (2004). Specific induction of the high-molecular-weight microtubule-associated protein 2 (hmw-MAP2) by betel quid extract in cultural oral keratinocytes: Clinical implications in betel quid-associated oral squamous cell carcinoma (OSCC). Carcinogenesis, 25(2), 269–276. https://doi.org/10.1093/carcin/bgh006

Chen, Q., Luo, G., Li, B., & Samaranayake, L. P. (1999). Expression of p16 and CDk4 in oral premalignant lesions and oral squamous cell carcinomas: A semi-quantitative immunohistochemical study. Journal of Oral Pathology and Medicine, 28(4), 158–164. https://doi.org/10.1111/j.1600-0714.1999.tb02016.x

Chen, X. J., Tan, Y. Q., Zhang, N., He, M. J., & Zhou, G. (2019). Expression of programmed cell death-ligand 1 in oral squamous cell carcinoma and oral leukoplakia is associated with disease progress and CD8+ tumor-infiltrating lymphocytes. Pathology Research and Practice, 215(6), 152418. https://doi.org/10.1016/j.prp.2019.04.010

Chen, Y. K., & Lin, L. M. (1995). Immunohistochemical demonstration of epithelial glutathione S‐transferase isoenzymes in normal, benign, premalignant and malignant human oral mucosa. Journal of Oral Pathology & Medicine, 24(7), 316–321. https://doi.org/10.1111/j.1600-0714.1995.tb01192.x

Chen, Yuk Kwan, Huang, A. H. C., Cheng, P. H., Yang, S. H., & Lin, L. M. (2013). Overexpression of Smad proteins, especially Smad7, in oral epithelial dysplasias. Clinical Oral Investigations, 17(3), 921–932. https://doi.org/10.1007/s00784-012-0756-7

Chowdhury, P., Nagamalini, B., Singh, J., Ashwini, B., & Sharada. (2021). Expression of β-catenin in oral leukoplakia and oral submucous fibrosis: An immunohistochemical study. Journal of Oral and Maxillofacial Pathology, 25(1), 124–130. https://doi.org/10.4103/jomfp.JOMFP_41_20

Cintorino, M., Petracca, R., Vindigni, C., Tripodi, S. A., & Leoncini, P. (1990). Topography-related expression of individual cytokeratins in normal and pathological (non-neoplastic and neoplastic) human oral mucosa. Virchows Archiv A Pathological Anatomy and Histopathology, 417(5), 419–426. https://doi.org/10.1007/BF01606030

da Silva, A. D., Maraschin, B. J., Laureano, N. K., Daroit, N., Brochier, F., Bündrich, L., … Rados, P. V. (2017). Expression of E-cadherin and involucrin in leukoplakia and oral cancer: an immunocytochemical and immunohistochemical study. Brazilian Oral Research, 31, 1–8. https://doi.org/10.1590/1807-3107BOR-2017.vol31.0019

Daniel, F. I., Rivero, E. R. C., Modolo, F., Lopes, T. G., & Salum, F. G. (2010). Immunohistochemical expression of DNA methyltransferases 1, 3a and 3b in oral leukoplakias and squamous cell carcinomas. Archives of Oral Biology, 55(12), 1024–1030. https://doi.org/10.1016/j.archoralbio.2010.08.009

Das, P., & Deshmukh, R. (2022). Expression of S100A7 in oral potentially malignant disorders: An immunohistochemical study. Journal of Oral and Maxillofacial Pathology, 26(3), 419. https://doi.org/10.4103/jomfp.jomfp_151_22

de-Assis, E. M., Pimenta, L. G. G. S., Costa-e-Silva, E., Souza, P. E. A., & Horta, M. C. R. (2012). Stromal myofibroblasts in oral leukoplakia and oral squamous cell carcinoma. Medicina Oral, Patologia Oral y Cirugia Bucal, 17(5), e733-8. https://doi.org/10.4317/medoral.17834

de Freitas Silva, B. S., Yamamoto-Silva, F. P., Pontes, H. A. R., & Pinto Júnior, D. dos S. (2014). E-cadherin downregulation and Twist overexpression since early stages of oral carcinogenesis. Journal of Oral Pathology and Medicine, 43(2), 125–131. https://doi.org/10.1111/jop.12096

De Lawall, M. A., & Crivelini, M. M. (2006). PCNA and P53 expression in oral leukoplakia with different degrees of keratinization. Journal of Applied Oral Science, 14(4), 276–280. https://doi.org/10.1590/s1678-77572006000400012

Deepa, A. G., Janardanan-Nair, B., & Varun, B. R. (2017). Podoplanin expression in oral potentially malignant disorders and oral squamous cell carcinoma. Journal of Clinical and Experimental Dentistry, 9(12), e1418–e1424. https://doi.org/10.4317/jced.54213

Dimitrios, A., Zisis, V., Anastasiadou, P., Anagnostou, L., Paraskevopoulos, K., & Poulopoulos, A. (2023). Aldehyde Dehydrogenase: An Off-Label Marker of Endothelial Activation in Oral Squamous Cell Carcinoma. Cureus, 15(7), e41596. https://doi.org/10.7759/cureus.41596

Ding, Y. M., Dong, J. H., Chen, L. L., & Zhang, H. D. (2009). Increased expression of galectin-1 is associated with human oral squamous cell carcinoma development. Oncology Reports, 21(4), 983–987. https://doi.org/10.3892/or_00000312

Dmello, C., Sawant, S., Chaudhari, P. R., Dongre, H., Ahire, C., D’Souza, Z. C., … Vaidya, M. (2018). Aberrant expression of vimentin predisposes oral premalignant lesion derived cells towards transformation. Experimental and Molecular Pathology, 105(3), 243–251. https://doi.org/10.1016/j.yexmp.2018.08.010

Dong, X. L., & Liu, Z. W. (2018). Clinical importance of microtubule-associated protein 1 light chain 3 and mammalian target of rapamycin expression in oral leukoplakia and oral squamous cell carcinoma. Hua Xi Kou Qiang Yi Xue Za Zhi = Huaxi Kouqiang Yixue Zazhi = West China Journal of Stomatology, 36(6), 613–618. https://doi.org/10.7518/hxkq.2018.06.006

Dong, Y., Wang, Z., Mao, F., Cai, L., Dan, H., Jiang, L., … Chen, Q. (2021). PD-1 blockade prevents the progression of oral carcinogenesis. Carcinogenesis, 42(6), 891–902. https://doi.org/10.1093/carcin/bgab035

Doss, D., Nirmal, M., Veeravarmal, Saravanan, R., & Venkatesh, A. (2020). Evaluating the expression of GLUT-1 in oral leukoplakia. Journal of Oral and Maxillofacial Pathology, 24(2), 308. https://doi.org/10.4103/jomfp.jomfp_220_17

Duarte, E. C. B., Ribeiro, D. C., Gomez, M. V., Ramos-Jorge, M. L., & Gomez, R. S. (2008). Genetic polymorphisms of carcinogen metabolizing enzymes are associated with oral leukoplakia development and p53 overexpression. Anticancer Research, 28(2 A), 1101–1106. Retrieved from https://pubmed.ncbi.nlm.nih.gov/18507060/

Duś-Ilnicka, I., Hałoń, A., Perra, A., & Radwan-Oczko, M. (2024). HPV related p16INK4A and HSV in benign and potentially malignant oral mucosa pathologies. BMC Oral Health, 24(1), 347. https://doi.org/10.1186/s12903-024-04105-z

Duś-Ilnicka, I., Radwan-Oczko, M., Gerber, H., & Hałoń, A. (2020). Histopathological assessment of oral leukoplakia. Osteonectin as possible biomarker for further diagnostics. Polish Journal of Pathology, 71(2), 138–145. https://doi.org/10.5114/pjp.2020.97021

Feng, C. J., Li, H. J., Li, J. N., Lu, Y. J., & Liao, G. Q. (2008). Expression of Mcm7 and Cdc6 in oral squamous cell carcinoma and precancerous lesions. Anticancer Research, 28(6 A), 3763–3769. Retrieved from https://pubmed.ncbi.nlm.nih.gov/19189662/

Fillies, T., Jogschies, M., Kleinheinz, J., Brandt, B., Joos, U., & Buerger, H. (2007). Cytokeratin alteration in oral leukoplakia and oral squamous cell carcinoma. Oncology Reports, 18(3), 639–643. https://doi.org/10.3892/or.18.3.639

Foki, E., Gangl, K., Kranebitter, V., Niederberger-Leppin, V., Eckl-Dorna, J., Wiebringhaus, R., … Heiduschka, G. (2020). Early effects of cigarette smoke extract on human oral keratinocytes and carcinogenesis in head and neck squamous cell carcinoma. Head and Neck, 42(9), 2348–2354. https://doi.org/10.1002/hed.26247

Gandolfo, M., Keszler, A., Lanfranchi, H., & Itoiz, M. E. (2011). Increased subepithelial vascularization and VEGF expression reveal potentially malignant changes in human oral mucosa lesions. Oral Surgery, Oral Medicine, Oral Pathology, Oral Radiology and Endodontology, 111(4), 486–493. https://doi.org/10.1016/j.tripleo.2010.11.018

Ghazi, N., Saghravanian, N., Taghi Shakeri, M., & Jamali, M. (2021). Evaluation of CD44 and TGF-B Expression in Oral Carcinogenesis. Journal of Dentistry (Shiraz, Iran), 22(1), 33–40. https://doi.org/10.30476/DENTJODS.2020.84393.1079

Girod, S. C., Krueger, G., & Pape, H. D. (1993). p53 and Ki 67 expression in preneoplastic and neoplastic lesions of the oral mucosa. International Journal of Oral and Maxillofacial Surgery, 22(5), 285–288. https://doi.org/10.1016/S0901-5027(05)80517-X

Girod, Sabine C., Pape, H. D., & Krueger, G. R. F. (1994). p53 and PCNA expression in carcinogenesis of the oropharyngeal mucosa. European Journal of Cancer. Part B: Oral Oncology, 30(6), 419–423. https://doi.org/10.1016/0964-1955(94)90023-X

Gissi, D. B., Gabusi, A., Tarsitano, A., Luccarini, L., Morandi, L., & Montebugnoli, L. (2018). Podoplanin expression as a predictive marker of dysplasia in oral leukoplakia. Journal of Cranio-Maxillofacial Surgery, 46(5), 759–764. https://doi.org/10.1016/j.jcms.2018.02.016

Goto, Y., Sueoka, E., Chiba, H., & Fujiki, H. (1999). Significance of heterogeneous nuclear ribonucleoprotein B1 as a new early detection marker for oral squamous cell carcinoma. Japanese Journal of Cancer Research, 90(12), 1358–1363. https://doi.org/10.1111/j.1349-7006.1999.tb00720.x

Greer, R. O., Meyers, A., Said, S. M., & Shroyer, K. R. (2008). Is p16INK4a protein expression in oral ST lesions a reliable precancerous marker? International Journal of Oral and Maxillofacial Surgery, 37(9), 840–846. https://doi.org/10.1016/j.ijom.2008.05.015

Greeshma, L. R., Joseph, A. P., Sivakumar, T. T., Raghavan Pillai, V., & Vijayakumar, G. (2023). Correlation of PD-1 and PD-L1 expression in oral leukoplakia and oral squamous cell carcinoma: an immunohistochemical study. Scientific Reports, 13(1), 21698. https://doi.org/10.1038/s41598-023-48572-w

Grochau, K. J., Safi, A. F., Drebber, U., Grandoch, A., Zöller, J. E., & Kreppel, M. (2019). Podoplanin expression in oral leukoplakia─a prospective study. Journal of Cranio-Maxillofacial Surgery, 47(3), 505–509. https://doi.org/10.1016/j.jcms.2018.12.005

Guan, W. Q., Li, Q., & Ouyang, Q. M. (2019). Expression and significance of periostin in tissues and serum in oral leukoplakia and squamous cell carcinoma. Cancer Biotherapy and Radiopharmaceuticals, 34(7), 444–450. https://doi.org/10.1089/cbr.2018.2764

Gupta, K., Metgud, R., & Gupta, J. (2015). Evaluation of stromal myofibroblasts in oral leukoplakia, oral submucous fibrosis, and oral squamous cell carcinoma - An immunohistochemical study. Journal of Cancer Research and Therapeutics, 11(4), 893–898. https://doi.org/10.4103/0973-1482.147700

Häkkinen, L., Kainulainen, T., Salo, T., Grenman, R., & Larjava, H. (1999). Expression of integrin α9 subunit and tenascin in oral leukoplakia, lichen planus, and squamous cell carcinoma. Oral Diseases, 5(3), 210–217. https://doi.org/10.1111/j.1601-0825.1999.tb00303.x

Hanbuli, H. M. E., & Sarie, M. A. A. (2022). KRAS Protein Expression in Oral Squamous Cell Carcinoma: A Potential Marker for Progression and Prognosis. Iranian Journal of Pathology, 17(4), 469–479. https://doi.org/10.30699/ijp.2022.550727.2856

He, W., Xiao, Y., & Chen, W. min. (2015). Expression of Ki-67 and P53 protein in oral squamous cell carcinoma and its clinical significance. Shanghai Kou Qiang Yi Xue = Shanghai Journal of Stomatology, 24(2), 228–231. Retrieved from https://pubmed.ncbi.nlm.nih.gov/25938156/

He, Y., Chen, Q., & Li, B. (2008). ATM in oral carcinogenesis: Association with clinicopathological features. Journal of Cancer Research and Clinical Oncology, 134(9), 1013–1020. https://doi.org/10.1007/s00432-008-0365-7

He, Y., Chen, Q. ming, Wu, L. yan, & Li, B. qi. (2008). Role of ataxia telangiectasis mutated in the oncogenesis of oral squamous cell carcinoma. Hua Xi Kou Qiang Yi Xue Za Zhi = Huaxi Kouqiang Yixue Zazhi = West China Journal of Stomatology, 26(1), 90–93. Retrieved from https://pubmed.ncbi.nlm.nih.gov/18357894/

Hefni, E., Menon, D., Ma, T., Asiedu, E. B., Sultan, A., Meiller, T., … Montaner, S. (2023). Angiopoietin-like 4 induces head and neck squamous cell carcinoma cell migration through the NRP1/ABL1/PXN pathway. Cellular Signalling, 108, 110697. https://doi.org/10.1016/j.cellsig.2023.110697

Herrera Costa, F., Narana Ribeiro El Achkar, V., Costa, V., Paladini, I., Kowalski, L. P., Rodarte Carvalho, Y., & Kaminagakura, E. (2019). Different Expression of Aldehyde Dehydrogenases 1A1 and 2 in Oral Leukoplakia with Epithelial Dysplasia and in Oral Squamous Cell Carcinoma. Applied Immunohistochemistry and Molecular Morphology, 27(7), 537–542. https://doi.org/10.1097/PAI.0000000000000612

Hiromoto, T., Noguchi, K., Yamamura, M., Zushi, Y., Segawa, E., Takaoka, K., … Urade, M. (2011). Up-regulation of neutrophil gelatinase-associated lipocalin in oral squamous cell carcinoma: Relation to cell differentiation. Oncology Reports, 26(6), 1415–1421. https://doi.org/10.3892/or.2011.1429

Hu, F., Chen, X., Liu, X., Wang, C., Lv, L., Xie, N., … Huang, H. (2015). Clinicopathological features and prognostic implications of Raf kinase inhibitor protein downregulation in tongue squamous cell carcinoma. Oncology Letters, 10(3), 1303–1308. https://doi.org/10.3892/ol.2015.3496

Humayun, S., & Prasad, Vr. (2011). Expression of p53 protein and ki-67 antigen in oral premalignant lesions and oral squamous cell carcinomas: An immunohistochemical study. National Journal of Maxillofacial Surgery, 2(1), 38. https://doi.org/10.4103/0975-5950.85852

Ishida, K., Ito, S., Wada, N., Deguchi, H., Hata, T., Hosoda, M., & Nohno, T. (2007). Nuclear localization of beta-catenin involved in precancerous change in oral leukoplakia. Molecular Cancer, 6, 62. https://doi.org/10.1186/1476-4598-6-62

Iwasa, M., Imamura, Y., Noriki, S., Nishi, Y., Kato, H., & Fukuda, M. (2001). Immunohistochemical detection of early-stage carcinogenesis of oral leukoplakia by increased DNA-instability and various malignancy markers. European Journal of Histochemistry, 45(4), 333–346. https://doi.org/10.4081/1642

Jane, C., Nerurkar, A. V., Shirsat, N. V., Deshpande, R. B., Amrapurkar, A. D., & Karjodkar, F. R. (2006). Increased survivin expression in high-grade oral squamous cell carcinoma: A study in Indian tobacco chewers. Journal of Oral Pathology and Medicine, 35(10), 595–601. https://doi.org/10.1111/j.1600-0714.2006.00473.x

Jayaraj, G., Sherlin, H. J., Ramani, P., Premkumar, P., & Natesan, A. (2015). Stromal myofibroblasts in oral squamous cell carcinoma and potentially malignant disorders. Indian Journal of Cancer, 52(1), 87–92. https://doi.org/10.4103/0019-509X.175580

Jin, Y., Tipoe, G. L., Liong, E. C., Lau, T. Y. H., Fung, P. C. W., & Leung, K. M. (2001). Overexpression of BMP-2/4, -5 and BMPR-IA associated with malignancy of oral epithelium. Oral Oncology, 37(3), 225–233. https://doi.org/10.1016/S1368-8375(00)00087-7

Johann, A. C. B. R., Da Silveira, J. B., Souto, G. R., Horta, M. C. R., Aguiar, M. C. F., & Mesquita, R. A. (2008). Metallothionein immunoexpression in oral leukoplakia. Medicina Oral, Patologia Oral y Cirugia Bucal, 13(3), 156–160. Retrieved from https://pubmed.ncbi.nlm.nih.gov/18305434/

Juneja, S., Chaitanya, N., & Agarwal, M. (2015). Immunohistochemical expression of Bcl-2 in oral epithelial dysplasia and oral squamous cell carcinoma. Indian Journal of Cancer, 52(4), 505–510. https://doi.org/10.4103/0019-509X.178411

Kamat, S. S., Kumar, G. S., & Koshy, A. V. (2013). Immunohistochemical analysis of syndecan-1 in leukoplakia and oral submucous fibrosis. Dental Research Journal, 10(3), 321–327. Retrieved from http://www.ncbi.nlm.nih.gov/pubmed/24019799%0Ahttp://www.pubmedcentral.nih.gov/articlerender.fcgi?artid=PMC3760354

Kamperos, G., Nikitakis, N., Sfakianou, A., Avgoustidis, D., & Sklavounou-Andrikopoulou, A. (2016). Expression of NF-ĸB and IL-6 in oral precancerous and cancerous lesions: An immunohistochemical study. Medicina Oral, Patologia Oral y Cirugia Bucal, 21(1), e6–e13. https://doi.org/10.4317/medoral.20570

Kang, Y., Chen, J., Li, X., Luo, M., Chen, H., Cui, B., … Zhang, P. (2021). Salivary KLK5 and uPA are potential biomarkers for malignant transformation of OLK and OLP. Cancer Biomarkers, 31(4), 317–328. https://doi.org/10.3233/CBM-203105

Kannan, S., Balaram, P., Chandran, G. J., Pillai, M. R., Mathew, B., & Nair, M. K. (1993). Expression of lectin-specific cellular glycoconjugates during oral carcinogenesis. Journal of Cancer Research and Clinical Oncology, 119(11), 689–694. https://doi.org/10.1007/BF01215989

Kannan, S., Balaram, P., Chandran, G. J., Pillai, M. R., Mathew, B., Nalinakumari, K. R., & Nair, M. K. (1994). Alterations in expression of basement membrane proteins during tumour progression in oral mucosa. Histopathology, 24(6), 531–537. https://doi.org/10.1111/j.1365-2559.1994.tb00571.x

Kannan, S., Jagadeesh Chandran, G., Raveendran Pillai, K., Mathew, B., Sujathan, K., Nalinakumary, K. R., & Krishnan Nair, M. (1996). Expression of p53 in leukoplakia and squamous cell carcinoma of the oral mucosa: Correlation with expression of Ki67. Journal of Clinical Pathology - Clinical Molecular Pathology, 49(3), M170-5. https://doi.org/10.1136/mp.49.3.m170

Kaur, Jasbir, Srivastava, A., & Ralhan, R. (1994). Overexpression of p53 protein in betel‐ and tobacco‐related human oral dysplasia and squamous‐cell carcinoma in India. International Journal of Cancer, 58(3), 340–345. https://doi.org/10.1002/ijc.2910580305

Kaur, Jatinder, Sawhney, M., DattaGupta, S., Shukla, N. K., Srivastava, A., & Ralhan, R. (2010). Clinical significance of Phosphatidyl Inositol Synthase overexpression in oral cancer. BMC Cancer, 10, 168. https://doi.org/10.1186/1471-2407-10-168

Khan, Z., Tiwari, R. P., Mulherkar, R., Sah, N. K., Prasad, G. B. K. S., Shrivastava, B. R., & Bisen, P. S. (2009). Detection of survivin and p53 in human oral cancer: Correlation with clinicopathologic findings. Head and Neck, 31(8), 1039–1048. https://doi.org/10.1002/hed.21071

Kitamura, R., Toyoshima, T., Tanaka, H., Kawano, S., Kiyosue, T., Matsubara, R., … Nakamura, S. (2012). Association of cytokeratin 17 expression with differentiation in oral squamous cell carcinoma. Journal of Cancer Research and Clinical Oncology, 138(8), 1299–1310. https://doi.org/10.1007/s00432-012-1202-6

Kobayashi, I., Matsuo, K., Ozeki, S., Ohishi, M., Ishibashi, Y., & Sakai, H. (1995). The proliferative activity in oral epithelial dysplasia analyzed by proliferating cell nuclear antigen immunostaining and argyrophilic nucleolar organizer region staining. Human Pathology, 26(8), 907–913. https://doi.org/10.1016/0046-8177(95)90015-2

Kouketsu, A., Sato, I., Oikawa, M., Shimizu, Y., Saito, H., Takahashi, T., & Kumamoto, H. (2019). Expression of immunoregulatory molecules PD-L1 and PD-1 in oral cancer and precancerous lesions: A cohort study of Japanese patients. Journal of Cranio-Maxillofacial Surgery, 47(1), 33–40. https://doi.org/10.1016/j.jcms.2017.04.013

Kövesi, G., & Szende, B. (2003). Changes in apoptosis and mitotic index, p53 and Ki67 expression in various types of oral leukoplakia. Oncology, 65(4), 331–336. https://doi.org/10.1159/000074646

Kövesi, G., & Szende, B. (2006). Prognostic value of cyclin D1, p27, and p63 in oral leukoplakia. Journal of Oral Pathology and Medicine, 35(5), 274–277. https://doi.org/10.1111/j.1600-0714.2006.00396.x

Kujan, O., Agag, M., Smaga, M., Vaishnaw, Y., Idrees, M., Shearston, K., & Farah, C. S. (2022). PD-1/PD-L1, Treg-related proteins, and tumour-infiltrating lymphocytes are associated with the development of oral squamous cell carcinoma. Pathology, 54(4), 409–416. https://doi.org/10.1016/j.pathol.2021.09.013

Kuo, M. Y. P., Chang, H. H., Hahn, L. J., Wang, J. T., & Chiang, C. P. (1995). Elevated ras p21 expression in oral premalignant lesions and squamous cell carcinomas in Taiwan. Journal of Oral Pathology & Medicine, 24(6), 255–260. https://doi.org/10.1111/j.1600-0714.1995.tb01178.x

Kurokawa, H., Matsumoto, S., Murata, T., Yamashita, Y., Tomoyose, T., Zhang, M., … Takahashi, T. (2003). Immunohistochemical study of syndecan-1 down-regulation and the expression of p53 protein or Ki-67 antigen in oral leukoplakia with or without epithelial dysplasia. Journal of Oral Pathology and Medicine, 32(9), 513–521. https://doi.org/10.1034/j.1600-0714.2003.00117.x

Kyrodimou, M., Andreadis, D., Drougou, A., Amanatiadou, E. P., Angelis, L., Barbatis, C., … Vizirianakis, I. S. (2014). Desmoglein-3/γ-catenin and E-cadherin/ß-catenin differential expression in oral leukoplakia and squamous cell carcinoma. Clinical Oral Investigations, 18(1), 199–210. https://doi.org/10.1007/s00784-013-0937-z

Laishram, D., Rao, K., Devi, H. S. U., Priya, N. S., Smitha, T., & Sheethal, H. S. (2017). Mast cells and angiogenesis in malignant and premalignant oral lesions: An immunohistochemical study. Journal of Oral and Maxillofacial Pathology, 21(2), 229–238. https://doi.org/10.4103/jomfp.JOMFP_111_15

Lameira, A. G., Pontes, F. S. C., Guimarães, D. M., Alves, A. C. G., de Jesus, A. S., Pontes, H. A. R., & Pinto, D. dos S. (2014). MCM3 could be a better marker than Ki-67 for evaluation of dysplastic oral lesions: An immunohistochemical study. Journal of Oral Pathology and Medicine, 43(6), 427–434. https://doi.org/10.1111/jop.12153

Lee, J., Lee, S. K., Lee, B. U., Lee, H. J., Cho, N. P., Yoon, J. H., … Kim, E. C. (2008). Upregulation of heme oxygenase-1 in oral epithelial dysplasias. International Journal of Oral and Maxillofacial Surgery, 37(3), 287–292. https://doi.org/10.1016/j.ijom.2007.07.028

Leite, A. F. S. de A., Bernardo, V. G., Buexm, L. A., da Fonseca, E. C., da Silva, L. E., Barroso, D. R. C., & Lourenço, S. de Q. C. (2016). Immunoexpression of cleaved caspase-3 shows lower apoptotic area indices in lip carcinomas than in intraoral cancer. Journal of Applied Oral Science, 24(4), 359–365. https://doi.org/10.1590/1678-775720160156

Li, H. G., Han, J. J., Huang, Z. Q., Wang, L., Chen, W. L., & Shen, X. M. (2011). IMP3 is a novel biomarker to predict metastasis and prognosis of tongue squamous cell carcinoma. Journal of Craniofacial Surgery, 22(6), 2022–2025. https://doi.org/10.1097/SCS.0b013e3182319750

Liao, J., Mitsuyasu, T., Yamane, K., & Ohishi, M. (2000). Telomerase activity in oral and maxillofacial tumors. Oral Oncology, 36(4), 347–352. https://doi.org/10.1016/S1368-8375(00)00013-0

Lin, L., Wang, J., Liu, D., Liu, S., Xu, H., Ji, N., … Chen, Q. (2016). Interleukin-37 expression and its potential role in oral leukoplakia and oral squamous cell carcinoma. Scientific Reports, 6, 26757. https://doi.org/10.1038/srep26757

Liu, Y. M., Huang, J. H., Feng, D. Y., & Guo, X. C. (2005). Expression of survivin and its correlation to angiogenesis in oral squamous cell carcinoma. Ai Zheng = Aizheng = Chinese Journal of Cancer, 24(11), 1354–1357. Retrieved from https://pubmed.ncbi.nlm.nih.gov/16552962/

Lo Muzio, L., Campisi, G., Giovannelli, L., Ammatuna, P., Greco, I., Staibano, S., … D’Angelo, M. (2004). HPV DNA and survivin expression in epithelial oral carcinogenesis: A relationship? Oral Oncology, 40(7), 736–741. https://doi.org/10.1016/j.oraloncology.2003.11.011

Logeswari, J., Malathi, N., Thamizhchelvan, H., Sangeetha, N., & Nirmala, S. (2014). Expression of podoplanin in oral premalignant and malignant lesions and its potential as a biomarker. Indian Journal of Dental Research, 25(3), 305–310. https://doi.org/10.4103/0970-9290.138321

Lopes, N. M., Xavier, F. C. A., Ortiz, R. C., Amôr, N. G., Garlet, G. P., Lara, V. S., … Rodini, C. O. (2018). Subcellular localization and expression of E-cadherin and SNAIL are relevant since early stages of oral carcinogenesis. Pathology Research and Practice, 214(8), 1185–1191. https://doi.org/10.1016/j.prp.2018.06.004

López de Cicco, R., Bassi, D. E., Page, R., & Klein-Szanto, A. J. (2002). Furin expression in squamous cell carcinomas of the oral cavity and other sites evaluated by tissue microarray technology. Acta Odontológica Latinoamericana : AOL, 15(1–2), 29–37. Retrieved from https://pubmed.ncbi.nlm.nih.gov/15208940/

Lu, H. (1989). The diagnostic significance of isoantigens ABH in oral leukoplakia. Zhonghua Kou Qiang Yi Xue Za Zhi = Zhonghua Kouqiang Yixue Zazhi = Chinese Journal of Stomatology, 24(4), 201–203, 254. Retrieved from https://pubmed.ncbi.nlm.nih.gov/2517092/

Luiz, S. T., Modolo, F., Mozzer, I., dos Santos, E. C., Nagashima, S., Camargo Martins, A. P., … Johann, A. C. B. R. (2018). Immunoexpression of SOX-2 in oral leukoplakia. Oral Diseases, 24(8), 1449–1457. https://doi.org/10.1111/odi.12922

Luomanen, M., Tiitta, O., Heikinheimo, K., Heinaro, I., & Happonen, R. P. (1997). Effect of snuff on cytokeratin expression in oral vestibular sulcus epithelium. Journal of Oral Pathology and Medicine, 26(3), 110–116. https://doi.org/10.1111/j.1600-0714.1997.tb00032.x

MacHa, M. A., Matta, A., Kaur, J., Chauhan, S. S., Thakar, A., Shukla, N. K., … Ralhan, R. (2011). Prognostic significance of nuclear pSTAT3 in oral cancer. Head and Neck, 33(4), 482–489. https://doi.org/10.1002/hed.21468

MacHa, M. A., Matta, A., Sriram, U., Thakkar, A., Shukla, N. K., Datta Gupta, S., & Ralhan, R. (2010). Clinical significance of TC21 overexpression in oral cancer. Journal of Oral Pathology and Medicine, 39(6), 477–485. https://doi.org/10.1111/j.1600-0714.2009.00854.x

Mack, B., & Gires, O. (2008). CD44s and CD44v6 expression in head and neck epithelia. PLoS ONE, 3(10), e3360. https://doi.org/10.1371/journal.pone.0003360

Mandel, U., Dabelsteen, E., Gaggero, B., Reibel, J., Therkildsen, M. H., & Clausen, H. (1994). Oncofetal fibronectins in oral carcinomas: Correlation of two different types. Apmis, 102(7–12), 695–702. https://doi.org/10.1111/j.1699-0463.1994.tb05222.x

Mane, D., Bhat, K., Kale, A., & Hallikerimath, S. (2015). Immunoexpression of tenascin as a predictor of the malignancy potential of oral leukoplakia associated with a tobacco habit. Biotechnic and Histochemistry, 90(7), 544–551. https://doi.org/10.3109/10520295.2015.1015055

Martín-Ezquerra, G., Salgado, R., Toll, A., Baró, T., Mojal, S., Yébenes, M., … Pujol, R. M. (2011). CDC28 protein kinase regulatory subunit 1B (CKS1B) expression and genetic status analysis in oral squamous cell carcinoma. Histology and Histopathology, 26(1), 71–77. https://doi.org/10.14670/HH-26.71

Matias, M. D. P., Meirelles, D. P., Horta, M. C. R., da Silva, K. D., Caldeira, P. C., & de Aguiar, M. C. F. (2023). ALDH1 immunoexpression in epithelial and stromal cells of oral lichen planus and lesions with lichenoid inflammatory infiltrate. Medicina Oral Patologia Oral y Cirugia Bucal, 28(6), e512–e518. https://doi.org/10.4317/medoral.25861

Mazumdar, S., Sengupta, S. K., Param, R., & Sinha, S. N. (1993). Binding pattern of eight different lectins in healthy subjects and patients with dysplastic and malignant lesions of the oral cavity. International Journal of Oral and Maxillofacial Surgery, 22(5), 301–305. https://doi.org/10.1016/S0901-5027(05)80521-1

Meka, N. J., Ugrappa, S., Velpula, N., Kumar, S., Maloth, K. N., Kodangal, S., … Goyal, S. (2015). Quantitative immunoexpression of egfr in oral potentially malignant disorders: Oral leukoplakia and oral submucous fibrosis. Journal of Dental Research, Dental Clinics, Dental Prospects, 9(3), 166–174. https://doi.org/10.15171/JODDD.2015.031

Meng, W., Xia, Q., Wu, L., Chen, S., He, X., Zhang, L., … Zhou, H. (2011). Downregulation of TGF-beta receptor types II and III in oral squamous cell carcinoma and oral carcinoma-associated fibroblasts. BMC Cancer, 11, 88. https://doi.org/10.1186/1471-2407-11-88

Migliorati, C. A., Migliorati, E. K. J., Silverman, S., Greenspan, D., & Greenspan, J. S. (1986). Phenotypic identification of mononuclear cells in oral premalignant lesions and cancer by monoclonal antibodies. Journal of Oral Pathology & Medicine, 15(6), 352–358. https://doi.org/10.1111/j.1600-0714.1986.tb00639.x

Miguel, A. F. P., Poletto, D. A. G., Embaló, B., & Rivero, E. R. C. (2023). Association between epithelial-mesenchymal transition markers, proliferative index, and oral epithelial dysplasia: an immunohistochemical study. Oral Surgery, Oral Medicine, Oral Pathology and Oral Radiology, 135(6), 904–913. https://doi.org/10.1016/j.oooo.2023.03.005

Miyahara, L. A. N., Pontes, F. S. C., Burbano, R. M. R., Conte Neto, N., Guimarães, D. M., Fonseca, F. P., & Pontes, H. A. R. (2018). PTEN allelic loss is an important mechanism in the late stage of development of oral leucoplakia into oral squamous cell carcinoma. Histopathology, 72(2), 330–338. https://doi.org/10.1111/his.13381

Mondal, K., Mandal, R., & Sarkar, B. (2016). A study of Ki-67 expression and its clinicopathological determinants in nondysplastic oral leukoplakia. Contemporary Clinical Dentistry, 7(4), 493–499. https://doi.org/10.4103/0976-237X.194106

Montebugnoli, L., Venturi, M., Gissi, D. B., Leonardi, E., Farnedi, A., & Foschini, M. P. (2011). Immunohistochemical expression of p16 INK4A protein in oral lichen planus. Oral Surgery, Oral Medicine, Oral Pathology, Oral Radiology and Endodontology, 112(2), 222–227. https://doi.org/10.1016/j.tripleo.2011.02.029

Muraki, Y., Yoshioka, C., Fukuda, J., Haneji, T., & Kobayashi, N. (1997). Immunohistochemical detection of Fas antigen in oral epithelia. Journal of Oral Pathology and Medicine, 26(2), 57–62. https://doi.org/10.1111/j.1600-0714.1997.tb00022.x

Nakahara, Y., Shintani, S., Mihara, M., Kiyota, A., Ueyama, Y., & Matsumura, T. (2000). Alterations of Rb, p16(INK4A) and cyclin D1 in the tumorigenesis of oral squamous cell carcinomas. Cancer Letters, 160(1), 3–8. https://doi.org/10.1016/S0304-3835(00)00546-2

Nanda, K. D. S., Ranganathan, K., Devi, U., & Joshua, E. (2012). Increased expression of CK8 and CK18 in leukoplakia, oral submucous fibrosis, and oral squamous cell carcinoma: An immunohistochemistry study. Oral Surgery, Oral Medicine, Oral Pathology and Oral Radiology, 113(2), 245–253. https://doi.org/10.1016/j.tripleo.2011.05.034

Narashiman, S., Narasimhan, M., & Venkatraman, G. (2014). Expression of Mucin 4 in leukoplakia and oral squamous cell carcinoma: An immunohistochemical study. Journal of Oral and Maxillofacial Pathology, 18(1), 25–31. https://doi.org/10.4103/0973-029X.131887

Nayak, S., Goel, M. M., Bhatia, V., Chandra, S., Makker, A., Kumar, S., … Rath, S. K. (2013). Molecular and phenotypic expression of decorin as modulator of angiogenesis in human potentially malignant oral lesions and oral squamous cell carcinomas. Indian Journal of Pathology and Microbiology, 56(3), 204–210. https://doi.org/10.4103/0377-4929.120366

Negi, A., Puri, A., Gupta, R., Nangia, R., Sachdeva, A., & Mittal, M. (2015). Comparison of immunohistochemical expression of antiapoptotic protein survivin in normal oral mucosa, oral leukoplakia, and oral squamous cell carcinoma. Pathology Research International, 2015, 840739. https://doi.org/10.1155/2015/840739

Ni, Y. H., Ding, L., Zhang, D. Y., Hou, Y. Y., Huang, X., & Hu, Q. (2015). Distinct expression patterns of Toll-like receptor 7 in tumour cells and fibroblast-like cells in oral squamous cell carcinoma. Histopathology, 67(5), 730–739. https://doi.org/10.1111/his.12703

Nikitakis, N. G., Rassidakis, G. Z., Tasoulas, J., Gkouveris, I., Kamperos, G., Daskalopoulos, A., & Sklavounou, A. (2018). Alterations in the expression of DNA damage response-related molecules in potentially preneoplastic oral epithelial lesions. Oral Surgery, Oral Medicine, Oral Pathology and Oral Radiology, 125(6), 637–649. https://doi.org/10.1016/j.oooo.2018.03.006

Nishioka, H., Hiasa, Y., Hayashi, I., Kitahori, Y., Konishi, N., & Sugimura, M. (1993). Immunohistochemical detection of p53 oncoprotein in human oral squamous cell carcinomas and leukoplakias: Comparison with proliferating cell nuclear antigen staining and correlation with clinicopathological findings. Oncology (Switzerland), 50(6), 426–429. https://doi.org/10.1159/000227223

Norhany, S., Kouzu, Y., Uzawa, K., Hayama, M., Higo, M., Koike, H., … Tanzawa, H. (2006). Overexpression of PAX5 in oral carcinogenesis. Oncology Reports, 16(5), 1003–1008. https://doi.org/10.3892/or.16.5.1003

Ohkura, S., Kondoh, N., Hada, A., Arai, M., Yamazaki, Y., Sindoh, M., … Yamamoto, M. (2005). Differential expression of the keratin-4, -13, -14, -17 and transglutaminase 3 genes during the development of oral squamous cell carcinoma from leukoplakia. Oral Oncology, 41(6), 607–613. https://doi.org/10.1016/j.oraloncology.2005.01.011

Oliver, R. J., & Gordon MacDonald, D. (2000). Comparison of BrdU and cyclin A as markers of the S-phase in oral precancerous lesions. Journal of Oral Pathology and Medicine, 29(9), 426–431. https://doi.org/10.1034/j.1600-0714.2000.290902.x

Palani, J., Lakshminarayanan, V., & Kannan, R. (2011). Immunohistochemical detection of human telomerase reverse transcriptase in oral cancer and pre-cancer. Indian Journal of Dental Research, 22(2), 362. https://doi.org/10.4103/0970-9290.84281

Pande, P., Mathur, M., Shukla, N. K., & Ralhan, R. (1998). pRB and p16 protein alterations in human oral tumorigenesis. Oral Oncology, 34(5), 396–403. https://doi.org/10.1016/S1368-8375(98)00024-4

Papadopoulos, P., Zisis, V., Andreadis, D., Vahtsevanos, K., & Poulopoulos, A. (2024). Investigation of the Vascular-Endothelial Pattern of Expression of DAPK-1 in Oral Squamous Cell Carcinoma and Oral Potentially Malignant Disorders Through Immunohistochemistry. Cureus, 16(6), e63519. https://doi.org/10.7759/cureus.63519

Patel, S. B., Manjunatha, B. S., Shah, V., Soni, N., & Sutariya, R. (2017). Immunohistochemical evaluation of p63 and cyclin D1 in oral squamous cell carcinoma and leukoplakia. Journal of the Korean Association of Oral and Maxillofacial Surgeons, 43(5), 324–330. https://doi.org/10.5125/jkaoms.2017.43.5.324

Patil, A., Patil, K., Tupsakhare, S., Gabhane, M., Sonune, S., & Kandalgaonkar, S. (2015). Evaluation of Podoplanin in Oral Leukoplakia and Oral Squamous Cell Carcinoma. Scientifica, 2015, 1–6. https://doi.org/10.1155/2015/135298

Pérez, M. Á., Gandolfo, M. S., Masquijo Bisio, P., Paparella, M. L., & Itoiz, M. E. (2018). Different expression patterns of carbonic anhydrase IX in oral lichen planus and leukoplakia. Acta Odontologica Latinoamericana : AOL, 31(2), 77–81. Retrieved from http://www.ncbi.nlm.nih.gov/pubmed/30383070

Piattelli, A., Rubini, C., Fioroni, M., Iezzi, G., & Santinelli, A. (2002). Prevalence of p53, bcl-2, and Ki-67 imnmnoreactivity and of apoptosis in normal oral epithelium and in premalignant and malignant lesions of the oral cavity. Journal of Oral and Maxillofacial Surgery, 60(5), 532–540. https://doi.org/10.1053/joms.2002.31851

Pigatti, F. M., Taveira, L. A. de A., & Soares, C. T. (2015). Immunohistochemical expression of Bcl-2 and Ki-67 in oral lichen planus and leukoplakia with different degrees of dysplasia. International Journal of Dermatology, 54(2), 150–155. https://doi.org/10.1111/ijd.12279

Pimenta, F. J., Cordeiro, G. T., Pimenta, L. G. G. S., Viana, M. B., Lopes, J., Gomez, M. V., … Gomez, R. S. (2008). Molecular alterations in the tumor suppressor gene WWOX in oral leukoplakias. Oral Oncology, 44(8), 753–758. https://doi.org/10.1016/j.oraloncology.2007.08.019

Pimenta, F. J., Horta, M. C. R., Vidigal, P. V., De Souza, B. R., De Marco, L., Romano-Silva, M. A., & Gomez, R. S. (2007). Decreased expression of DARPP-32 in oral premalignant and malignant lesions. Anticancer Research, 27(4 B), 2339–2343. Retrieved from https://pubmed.ncbi.nlm.nih.gov/17695523/

Pitigala‐Arachchi, A., Crane, I. J., Scully, C., & Prime, S. S. (1989). Epithelial dendritic cells in pathological human oral tissues. Journal of Oral Pathology & Medicine, 18(1), 11–16. https://doi.org/10.1111/j.1600-0714.1989.tb00724.x

Pogoda, K., Cieśluk, M., Deptuła, P., Tokajuk, G., Piktel, E., Król, G., … Bucki, R. (2021). Inhomogeneity of stiffness and density of the extracellular matrix within the leukoplakia of human oral mucosa as potential physicochemical factors leading to carcinogenesis. Translational Oncology, 14(7), 101105. https://doi.org/10.1016/j.tranon.2021.101105

Poomsawat, S., Buajeeb, W., Khovidhunkit, S. on, & Punyasingh, J. (2010). Alteration in the expression of cdk4 and cdk6 proteins in oral cancer and premalignant lesions. Journal of Oral Pathology and Medicine, 39(10), 793–799. https://doi.org/10.1111/j.1600-0714.2010.00909.x

Poomsawat, S., Kariya, A., Nimmanon, T., Kosanwat, T., Juengsomjit, R., & Sanguansin, S. (2023). Diagnostic potential of Type VII Collagen during oral carcinogenesis. Journal of Applied Oral Science, 31, e20220486. https://doi.org/10.1590/1678-7757-2022-0486

Poomsawat, S., Kosanwat, T., Meesakul, O., & Sanguansin, S. (2022). Epithelial and fibroblast SPARC expression patterns in oral leukoplakia and oral squamous cell carcinoma. Oral Surgery, Oral Medicine, Oral Pathology and Oral Radiology, 134(2), e44–e50. https://doi.org/10.1016/j.oooo.2021.10.019

Poomsawat, S., Punyasingh, J., & Vejchapipat, P. (2014). Overexpression of survivin and caspase 3 in oral carcinogenesis. Applied Immunohistochemistry and Molecular Morphology, 22(1), 65–71. https://doi.org/10.1097/PAI.0b013e31828a0d0c

Poomsawat, S., Punyasingh, J., & Vejchapipat, P. (2015). Aberrant expression of p-Smad3 in oral carcinogenesis. Clinical Oral Investigations, 19(3), 613–618. https://doi.org/10.1007/s00784-014-1281-7

Prakash, P., Khandare, M., Kumar, M., Khanna, R., Prakash Singh, G., Nath, G., & Kumar Gulati, A. (2013). Immunohistochemical detection of p16INK4ain leukoplakia and oral squamous cell carcinoma. Journal of Clinical and Diagnostic Research, 7(12), 2793–2795. https://doi.org/10.7860/JCDR/2013/7720.3882

Priyanka, K. P., Majumdar, S., Kotina, S., Uppala, Di., & Balla, H. (2019). Expression of heat shock protein 70 in oral epithelial dysplasia and oral squamous cell carcinoma: An immunohistochemical study. Contemporary Clinical Dentistry, 10(2), 185–190. https://doi.org/10.4103/ccd.ccd_101_18

Rahman, R., Poomsawat, S., Juengsomjit, R., & Buajeeb, W. (2019). Overexpression of Epstein-Barr virus-encoded latent membrane protein-1 (LMP-1) in oral squamous cell carcinoma. BMC Oral Health, 19(1), 142. https://doi.org/10.1186/s12903-019-0832-3

Raja, N., Ganesan, A., Chandrasekar Lakshmi, K., & Aniyan, Y. (2024). Assessing DNA methylation of ATG 5 and MAP1LC3Av1 gene in oral squamous cell carcinoma and oral leukoplakia- a cross sectional study. Journal of Oral Biology and Craniofacial Research, 14(5), 534–539. https://doi.org/10.1016/j.jobcr.2024.07.001

Ralhan, R., Agarwal, S., Nath, N., Mathur, M., Wasylyk, B., & Srivastava, A. (2001). Correlation between p53 gene mutations and circulating antibodies in betel- and tobacco-consuming North Indian population. Oral Oncology, 37(3), 243–250. https://doi.org/10.1016/S1368-8375(00)00092-0

Ralhan, Ranju, Narayan, M., Salotra, P., Shukla, N. K., & Chauhan, S. S. (1997). Evaluation of P-glycoprotein expression in human oral oncogenesis: Correlation with clinicopathological features. International Journal of Cancer, 72(5), 728–734. https://doi.org/10.1002/(SICI)1097-0215(19970904)72:5<728::AID-IJC4>3.0.CO;2-U

Ralhan, Ranju, Nath, N., Agarwal, S., Mathur, M., Wasylyk, B., & Shukla, N. K. (1998). Circulating p53 antibodies as early markers of oral cancer: Correlation with p53 alterations. Clinical Cancer Research, 4(9), 2147–2152. Retrieved from https://pubmed.ncbi.nlm.nih.gov/9748133/

Ramakrishna, A., Shreedhar, B., Narayan, T. V., Mohanty, L., Shenoy, S., & Jamadar, S. (2013). Cyclin D1 an early biomarker in oral carcinogenesis. Journal of Oral and Maxillofacial Pathology, 17(3), 351–357. https://doi.org/10.4103/0973-029X.125189

Ramasubramanian, A., Ramani, P., Sherlin, H. J., Premkumar, P., Natesan, A., & Thiruvengadam, C. (2013). Immunohistochemical evaluation of oral epithelial dysplasia using cyclin-D1, p27 and p63 expression as predictors of malignant transformation. Journal of Natural Science, Biology and Medicine, 4(2), 349–358. https://doi.org/10.4103/0976-9668.117011

Ranieri, G., Labriola, A., Achille, G., Florio, G., Zito, A. F., Grammatica, L., & Paradiso, A. (2002). Microvessel density, mast cell density and thymidine phosphorylase expression in oral squamous carcinoma. International Journal of Oncology, 21(6), 1317–1323. https://doi.org/10.3892/ijo.21.6.1317

Rathee, R., Devi, A., Narwal, A., Kamboj, M., & Singh, S. (2021). Immunohistochemical Coexpression of MUC1 and MUC4 in Oral Leukoplakia and Oral Squamous Cell Carcinoma. Head and Neck Pathology, 15(3), 831–842. https://doi.org/10.1007/s12105-021-01291-y

Ravi, D., Nalinakumari, K. R., Rajaram, R. S., Nair, M. K., & Pillai, M. R. (1996). Expression of programmed cell death regulatory p53 and bcl-2 proteins in oral lesions. Cancer Letters, 105(2), 139–146. https://doi.org/10.1016/0304-3835(96)04258-9

Reddy, V. M., Kamath, A., & Radhakrishnan, R. A. (2012). P53 immunoprofiling of potentially malignant oral disorders: A case series analysis. Indian Journal of Cancer, 49(1), 27–32. https://doi.org/10.4103/0019-509X.98913

Reibel, J., Clausen, H., & Dabelsteen, E. (1985). Staining patterns of human pre-malignant oral epithelium and squamous cell carcinomas by monoclonal anti-keratin antibodies. Acta Pathologica Microbiologica et Immunologica Scandinavica - Section A Pathology, 93(6), 323–330. https://doi.org/10.1111/j.1699-0463.1985.tb03958.x

Ribeiro, D. C., Gleber-Netto, F. O., Sousa, S. F., Bernardes, V. de F., Guimarães-Abreu, M. H. N., & Aguiar, M. C. F. (2012). Immunohistochemical expression of EGFR in oral leukoplakia: Association with clinicopathological features and cellular proliferation. Medicina Oral, Patologia Oral y Cirugia Bucal, 17(5), e739-44. https://doi.org/10.4317/medoral.17950

Rodríguez, M. J., Acha, A., Ruesga, M. T., Rodríguez, C., Rivera, J. M., & Aguirre, J. M. (2007). Loss of expression of DNA repair enzyme MGMT in oral leukoplakia and early oral squamous cell carcinoma. A prognostic tool? Cancer Letters, 245(1–2), 263–268. https://doi.org/10.1016/j.canlet.2006.01.015

Rohatgi, N., Matta, A., Kaur, J., Srivastava, A., & Ralhan, R. (2006). Novel molecular targets of smokeless tobacco (khaini) in cell culture from oral hyperplasia. Toxicology, 224(1–2), 1–13. https://doi.org/10.1016/j.tox.2006.03.014

Routray, S., Kheur, S. M., & Kheur, M. (2013). Osteopontin: A marker for invasive oral squamous cell carcinoma but not for potentially malignant epithelial dysplasias. Annals of Diagnostic Pathology, 17(5), 421–424. https://doi.org/10.1016/j.anndiagpath.2013.03.005

Sakthivel, R., Ramamoorthy, A., Jeddy, N., & Singaram, M. (2020). Evaluation and Expression of Survivin in Potentially Malignant Lesions and Squamous Cell Carcinoma: A Comparative Study. Cureus, 12(4), e7551. https://doi.org/10.7759/cureus.7551

Sanguansin, S., Kosanwat, T., Juengsomjit, R., & Poomsawat, S. (2021). Diagnostic Value of Cytokeratin 17 during Oral Carcinogenesis: An Immunohistochemical Study. International Journal of Dentistry, 2021, 4089549. https://doi.org/10.1155/2021/4089549

Santoshi, C., Kumar, J., Bhagirath, P., Vinay, B., & Prakash, Y. (2020). Morphometric analysis of basal cells of oral epithelium in predicting malignant transformation of oral potentially malignant disorders in patients with tobacco chewing habit. Journal of Oral and Maxillofacial Pathology, 24(3), 579–580. https://doi.org/10.4103/jomfp.JOMFP_55_20

Saranath, D., Tandle, A. T., Teni, T. R., Dedhia, P. M., Borges, A. M., Parikh, D., … Mehta, A. R. (1999). p53 inactivation in chewing tobacco-induced oral cancers and leukoplakias from India. Oral Oncology, 35(3), 242–250. https://doi.org/10.1016/S1368-8375(98)00110-9

Sawant, S., Dongre, H., Ahire, C., Sharma, S., Jamghare, S., Kansara, Y., … Dongre, P. (2018). Alterations in desmosomal adhesion at protein and ultrastructure levels during the sequential progressive grades of human oral tumorigenesis. European Journal of Oral Sciences, 126(4), 251–262. https://doi.org/10.1111/eos.12426

Sawant, S. S., Vaidya, M. M., Chaukar, D. A., Alam, H., Dmello, C., Gangadaran, P., … D’Cruz, A. K. (2014). Clinical significance of aberrant vimentin expression in oral premalignant lesions and carcinomas. Oral Diseases, 20(5), 453–465. https://doi.org/10.1111/odi.12151

Schulz, J., Ermich, T., Kasper, M., Raabe, G., & Schumann, D. (1992). Cytokeratin pattern of clinically intact and pathologically changed oral mucosa. International Journal of Oral and Maxillofacial Surgery, 21(1), 35–39. https://doi.org/10.1016/S0901-5027(05)80450-3

Selvaraj, F., Joseph, A., Varun, B., Mony, V., & Siva Kumar, T. (2022). Immunohistochemical evaluation of galectin-3 expression in oral squamous cell carcinoma, oral leukoplakia and normal mucosa. Indian Journal of Dental Research, 33(3), 282–286. https://doi.org/10.4103/ijdr.ijdr_157_22

Seoane, J. M., Varela-Centelles, P. I., Ramirez, J. R., Cameselle-Teijeiro, J., Romero, M. A., & Aguirre, J. M. (2006). Heat shock proteins (HSP70 and HSP27) as markers of epithelial dysplasia in oral leukoplakia. American Journal of Dermatopathology, 28(5), 417–422. https://doi.org/10.1097/01.dad.0000211509.44865.bb

Sheelam, S., Reddy, S. P., Kulkarni, P. G., Nandan, S., Keerthi, M., & Raj, G. S. (2018). Role of cell proliferation and vascularity in malignant transformation of potentially malignant disorders. Journal of Oral and Maxillofacial Pathology, 22(2), 281. https://doi.org/10.4103/jomfp.JOMFP_182_17

Shigeoka, M., Koma, Y. ichiro, Nishio, M., Komori, T., & Yokozaki, H. (2019). CD163+ macrophages infiltration correlates with the immunosuppressive cytokine interleukin 10 expression in tongue leukoplakia. Clinical and Experimental Dental Research, 5(6), 627–637. https://doi.org/10.1002/cre2.228

Shin, D. M., Xu, X. C., Lippman, S. M., Lee, J. J., Lee, J. S., Batsakis, J. G., … Hong, W. K. (1997). Accumulation of p53 protein and retinoic acid receptor β in retinoid chemoprevention. Clinical Cancer Research, 3(6), 875–880. Retrieved from https://pubmed.ncbi.nlm.nih.gov/9815762/

Shintani, S., Yoshihama, Y., Emilio, A. R., & Matsumura, Y. (1995). Overexpression of p53 is an early event in the tumorigenesis of oral squamous cell carcinomas. Anticancer Research, 15(2), 305–308. Retrieved from https://pubmed.ncbi.nlm.nih.gov/7762998/

Shirasuna, K., Hayashido, Y., Sugiyama, M., Yoshioka, H., & Matsuya, T. (1991). Immunohistochemical localization of epidermal growth factor (EGF) and EGF receptor in human oral mucosa and its malignancy. Virchows Archiv A Pathological Anatomy and Histopathology, 418(4), 349–353. https://doi.org/10.1007/BF01600165

Shyam, N. D. V. N., Rao, N. N., Narang, R. D. S., George, J., Bommu, S. R., & Kiran, G. (2014). Immunohistochemical characterization of cyclin dependent kinase-4 in different histological grades of oral leukoplakia and oral squamous cell carcinoma. Journal of Oral and Maxillofacial Pathology, 18(1), 36–41. https://doi.org/10.4103/0973-029X.131896

Singh, A., Singh, S., Soni, V., & Srivastava, D. k. (2023). A Comparative Study of Morphometric Analysis of Nucleolar Organizer Regions in Oral Leukoplakia and Oral Squamous Cell Carcinoma and Significance of AgNOR as a Diagnostic Tool. Cureus, 15(8), e44228. https://doi.org/10.7759/cureus.44228

Singh, D. N., Srivastava, K. C., Potsangbam, A. D., Shrivastava, D., Nandini, D. B., Singh, W. T., & Singh, K. S. (2020). A case-control study comparing and correlating iNOS expression among various clinicopathological variants of oral leukoplakia and oral squamous cell carcinoma: A Immunohistochemistry study. Journal of Pharmacy and Bioallied Sciences, 12(5), S324–S331. https://doi.org/10.4103/jpbs.JPBS_96_20

Singh, K. N., Ramadas, M. N., Veeran, V., Naidu, M. R., Dhanaraj, T. S., & Chandrasekaran, K. (2019). Expression pattern of the cancer stem cell marker “Nestin” in Leukoplakia and oral squamous cell Carcinoma. Rambam Maimonides Medical Journal, 10(4). https://doi.org/10.5041/RMMJ.10378

Singla, S., Singla, G., Zaheer, S., Rawat, D. S., & Mandal, A. K. (2018). Expression of p53, epidermal growth factor receptor, c-erbB2 in oral leukoplakias and oral squamous cell carcinomas. Journal of Cancer Research and Therapeutics, 14(2), 388–393. https://doi.org/10.4103/0973-1482.191027

Sirur, D., Tamgadge, A., Tamgadge, S., Bhalerao, S., & Gujjar, P. (2020). Correlation of p53 expression with histopathological and immunohistochemical features of human papillomavirus in oral leukoplakia. Journal of Microscopy and Ultrastructure, 8(3), 81–88. https://doi.org/10.4103/JMAU.JMAU_44_19

Sivakumar, N., Narwal, A., Kamboj, M., Devi, A., Kumar, S., & Bhardwaj, R. (2021). Molecular and Immunohistochemical Cognizance of HPV16 in Oral Leukoplakia, Oral Squamous Cell Carcinoma and Oropharyngeal Squamous Cell Carcinoma. Head and Neck Pathology, 15(3), 882–892. https://doi.org/10.1007/s12105-021-01309-5

Smitha, A., Rao, K., Umadevi, H. S., Smitha, T., Sheethal, H. S., & Vidya, M. A. (2019). Immunohistochemical study of α-smooth muscle actin expression in oral leukoplakia and oral squamous cell carcinoma. Journal of Oral and Maxillofacial Pathology, 23(1), 59–64. https://doi.org/10.4103/jomfp.JOMFP_94_18

Spolidorio, L. C., Neves, K. A., Soares, C. P., Spolidorio, D. M. P., Basso, M. F. M., Malavazzi, I., & Almeida, O. P. (2002). Evaluation of argyrophilic nucleolar organizer regions in oral tumor progression. Micron, 33(7–8), 605–608. https://doi.org/10.1016/S0968-4328(02)00031-8

Sravya, T., Sivaranjani, Y., & Rao, G. (2016). Immunohistochemical expression of budding uninhibited by benzimidazole related 1 in leukoplakia and oral squamous cell carcinoma. Journal of Oral and Maxillofacial Pathology, 20(1), 71–77. https://doi.org/10.4103/0973-029X.180938

Sridevi, U., Jain, A., Nagalaxmi, V., Kumar, U. V., & Goyal, S. (2015). Expression of E-cadherin in normal oral mucosa, in oral precancerous lesions and in oral carcinomas. European Journal of Dentistry, 9(3), 364–372. https://doi.org/10.4103/1305-7456.163238

Srinivasan, V., Shyam, N., Kumar, G. K., Narayen, V., Konda, P., & Swetha Rani, K. (2023). A Comparison of Podoplanin Expression in Oral Leukoplakia and Oral Squamous Cell Carcinoma: An Immunohistochemical Study. Cureus, 15(5), e38467. https://doi.org/10.7759/cureus.38467

Stasikowska-Kanicka, O., Wągrowska-Danilewicz, M., & Danilewicz, M. (2018). CD8+ and CD163+ infiltrating cells and PD-L1 immunoexpression in oral leukoplakia and oral carcinoma. Apmis, 126(9), 732–738. https://doi.org/10.1111/apm.12881

Sun, Y., Liu, N., Guan, X., Wu, H., Sun, Z., & Zeng, H. (2016). Immunosuppression Induced by Chronic Inflammation and the Progression to Oral Squamous Cell Carcinoma. Mediators of Inflammation, 2016, 5715719. https://doi.org/10.1155/2016/5715719

Sutariya, R. V., & Manjunatha, B. S. (2016). Immunohistochemical study of p21 and Bcl-2 in leukoplakia, oral submucous fibrosis and oral squamous cell carcinoma. Journal of Experimental Therapeutics and Oncology, 11(4), 285–292. Retrieved from https://pubmed.ncbi.nlm.nih.gov/27849339/

Suwasini, S., Chatterjee, K., Purkait, S. K., Samaddar, D., Chatterjee, A., & Kumar, M. (2018). Expression of p53 protein and Ki-67 antigen in oral leukoplakia with different histopathological grades of epithelial dysplasia. Journal of International Society of Preventive and Community Dentistry, 8(6), 513–522. https://doi.org/10.4103/jispcd.JISPCD_241_18

Świątkowski, W., Rahnama, M., Tomaszewski, T., Bigas, M., Świątkowska, A., Łobacz, M., & Wallner, J. (2017). Association between clinical stage of oral cancer and expression of immunohistochemical markers. Polish Journal of Surgery, 89(6), 17–22. https://doi.org/10.5604/01.3001.0010.6736

Takeshima, M., Saitoh, M., Kusano, K., Nagayasu, H., Kurashige, Y., Malsantha, M., … Abiko, Y. (2008). High frequency of hypermethylation of p14, p15 and p16 in oral pre-cancerous lesions associated with betel-quid chewing in Sri Lanka. Journal of Oral Pathology and Medicine, 37(8), 475–479. https://doi.org/10.1111/j.1600-0714.2008.00644.x

Tandon, N., Srivastava, A., Fatima, N., Raza, S., & Kumar, V. (2017). P53 Codon 72 Gene Polymorphism Studies and P53 Expression By Immunohistochemistry in Oral Lesions As Risk Factor for Malignancy. International Journal of Applied and Basic Medical Research, 7(4), 243. https://doi.org/10.4103/ijabmr.ijabmr_205_16

Tarle, M., Raguž, M., Muller, D., & Lukšić, I. (2023). Nuclear Epidermal Growth Factor Receptor Overexpression as a Survival Predictor in Oral Squamous Cell Carcinoma. International Journal of Molecular Sciences, 24(6). https://doi.org/10.3390/ijms24065816

Tashiro, K., Oikawa, M., Miki, Y., Takahashi, T., & Kumamoto, H. (2020). Immunohistochemical assessment of growth factor signaling molecules: MAPK, Akt, and STAT3 pathways in oral epithelial precursor lesions and squamous cell carcinoma. Odontology, 108(1), 91–101. https://doi.org/10.1007/s10266-019-00428-4

Tegginamani, A. S., Shivakumar, V. H., Ismail, S. M. B., Abraham, M. T., Fernandes, B. A., & Zamzuri, A. T. B. (2022). C-kit Expression in Oral Leukoplakia. Journal of the College of Physicians and Surgeons Pakistan, 32(2), 256–258. https://doi.org/10.29271/jcpsp.2022.02.256

Tegginamani, A., Shivakumar, V., Kallarakkal, T., Ismail, S., Abraham, M., & Bin Zamzuri, A. (2020). Analysis of octamer-binding transcription factor-4 expression in oral leukoplakia. Journal of Oral and Maxillofacial Pathology, 24(2), 400. https://doi.org/10.4103/jomfp.jomfp_272_19

Teresa, D. B., Neves, K. A., Neto, C. B., Fregonezi, P. A. G., de Oliveira, M. R. B., Zuanon, J. A. S., … Soares, C. P. (2007). Computer-assisted analysis of cell proliferation markers in oral lesions. Acta Histochemica, 109(5), 377–387. https://doi.org/10.1016/j.acthis.2007.03.007

Thorup, A. K., Reibel, J., Schiødt, M., Stenersen, T. C., Therkildsen, M. H., Carter, W. G., & Dabelsteen, E. (1998). Can alterations in integrin and laminin-5 expression be used as markers of malignancy. Apmis, 106(12), 1170–1180. https://doi.org/10.1111/j.1699-0463.1998.tb00274.x

Tomo, S., Biss, S. P., Crivelini, M. M., de Oliveira, S. H. P., Biasoli, É. R., Tjioe, K. C., … Miyahara, G. I. (2020). High p16INK4a immunoexpression is not HPV dependent in oral leukoplakia. Archives of Oral Biology, 115, 104738. https://doi.org/10.1016/j.archoralbio.2020.104738

Tosios, K. I., Kapranos, N., & Papanicolaou, S. I. (1998). Loss of basement membrane components laminin and type IV collagen parallels the progression of oral epithelial neoplasia. Histopathology, 33(3), 261–268. https://doi.org/10.1046/j.1365-2559.1998.00452.x

Tripathi, S. C., Matta, A., Kaur, J., Grigull, J., Chauhan, S. S., Thakar, A., … Michael Siu, K. W. (2010). Nuclear S100A7 is associated with poor prognosis in head and neck cancer. PLoS ONE, 5(8), e11939. https://doi.org/10.1371/journal.pone.0011939

Tsai, S. ‐T, & Jin, Y. ‐T. (1995). Proliferating cell nuclear antigen (PCNA) expression in oral squamous cell carcinomas. Journal of Oral Pathology & Medicine, 24(7), 313–315. https://doi.org/10.1111/j.1600-0714.1995.tb01191.x

Turatti, E., da Costa Neves, A., de Magalhães, M. H. C. G., & de Sousa, S. O. M. (2005). Assessment of c-Jun, c-Fos and cyclin D1 in premalignant and malignant oral lesions. Journal of Oral Science, 47(2), 71–76. https://doi.org/10.2334/josnusd.47.71

Vadla, P., Deepthi, G., Kumar, C., Bashamalla, R., Syeda, N., & Naramala, S. (2021). Immunohistochemical expression of stathmin in oral dysplasia: An original study with an insight of its action on microtubules. Journal of Oral and Maxillofacial Pathology, 25(2), 247–252. https://doi.org/10.4103/0973-029X.325122

Vadla, P., Yeluri, S., Deepthi, G., Guttikonda, V. R., Taneeru, S., & Naramala, S. (2020). Stathmin! An Immunohistochemical Analysis of the Novel Marker in Oral Squamous Cell Carcinoma and Oral Leukoplakia. Asian Pacific Journal of Cancer Prevention, 21(11), 3317–3323. https://doi.org/10.31557/APJCP.2020.21.11.3317

Valach, J., Foltán, R., Vlk, M., Szabo, P., & Smetana, K. (2017). Phenotypic characterization of oral mucosa: what is normal? Journal of Oral Pathology and Medicine, 46(9), 834–839. https://doi.org/10.1111/jop.12556

Varun, B. R., Ranganathan, K., Rao, U. K., & Joshua, E. (2014). Immunohistochemical detection of p53 and p63 in oral squamous cell carcinoma, oral leukoplakia, and oral submucous fibrosis. Journal of Investigative and Clinical Dentistry, 5(3), 214–219. https://doi.org/10.1111/jicd.12038

Vigneswaran, N., Peters, K. ‐P, Hornstein, O. P., & Diepgen, T. L. (1990). Alteration of cell surface carbohydrates associated with ordered and disordered proliferation of oral epithelia: a lectin histochemical study in oral leukoplakias, papillomas and carcinomas. Cell Proliferation, 23(1), 41–55. https://doi.org/10.1111/j.1365-2184.1990.tb01108.x

Vigneswaran, N., Peters, K. ‐P, Hornstein, O. P., & Haneke, E. (1989). Comparison of cytokeratin, filaggrin and involucrin profiles in oral leukoplakias and squamous carcinomas. Journal of Oral Pathology & Medicine, 18(7), 377–390. https://doi.org/10.1111/j.1600-0714.1989.tb01569.x

Vigneswaran, Nadarajah, Beckers, S., Waigel, S., Mensah, J., Wu, J., Mo, J., … Zacharias, W. (2006). Increased EMMPRIN (CD 147) expression during oral carcinogenesis. Experimental and Molecular Pathology, 80(2), 147–159. https://doi.org/10.1016/j.yexmp.2005.09.011

Vijayakumar, G., Narwal, A., Kamboj, M., & Sen, R. (2020). Association of SOX2, OCT4 and WNT5A Expression in Oral Epithelial Dysplasia and Oral Squamous Cell Carcinoma: An Immunohistochemical Study. Head and Neck Pathology, 14(3), 749–757. https://doi.org/10.1007/s12105-019-01114-1

Visioli, F., Lauxen, I. S., Sant’Ana Filho, M., & Rados, P. V. (2012). Expression of the cell cycle regulation proteins p53 and p21WAF1 in different types of non-dysplastic leukoplakias. Journal of Applied Oral Science, 20(3), 369–375. https://doi.org/10.1590/S1678-77572012000300013

Vora, H. H., Mehta, S. V., Shah, K. N., Brahmbhatt, B. V., Desai, N. S., Shukla, S. N., & Shah, P. M. (2007). Cytoplasmic Localization of BAG-1 in Leukoplakia and Carcinoma of the Tongue: Correlation with p53 and C-Erbb2 in Carcinoma. The International Journal of Biological Markers, 22(2), 100–107. https://doi.org/10.1177/172460080702200203

Vora, H. H., Trivedi, T. I., Shukla, S. N., Shah, N. G., Goswami, J. V., & Shah, P. M. (2006). p53 Expression in Leukoplakia and Carcinoma of the Tongue. The International Journal of Biological Markers, 21(2), 74–80. https://doi.org/10.1177/172460080602100202

Wang, J., Jin, X., Liu, J., Zhao, K., Xu, H., Wen, J., … Chen, Q. (2017). The prognostic value of B7-H6 protein expression in human oral squamous cell carcinoma. Journal of Oral Pathology and Medicine, 46(9), 766–772. https://doi.org/10.1111/jop.12586

Wang, L., Qi, F., Hao, L., & Sun, H. (2021). Evaluation of P53 gene expression by immunohistochemistry to diagnosis oral precancerous lesions. Cellular and Molecular Biology, 67(3), 158–162. https://doi.org/10.14715/cmb/2021.67.3.24

Wang, Q. M., Huang, X. Y., & Guan, W. Q. (2022). Expressions of Interleukin-27 in Oral Lichen Planus, Oral Leukoplakia, and Oral Squamous Cell Carcinoma. Inflammation, 45(3), 1023–1038. https://doi.org/10.1007/s10753-021-01599-5

Wang, Xiang, Jiang, W., Duan, N., Qian, Y., Zhou, Q., Ye, P., … Wang, W. (2014). NOD1, RIP2 and Caspase12 are potentially novel biomarkers for oral squamous cell carcinoma development and progression. International Journal of Clinical and Experimental Pathology, 7(4), 1677–1686. Retrieved from https://pubmed.ncbi.nlm.nih.gov/24817964/

Wang, Xin, Jin, Y., Li, Y. X., & Yang, Y. (2018). Secretory leukocyte peptidase inhibitor expression and apoptosis effect in oral leukoplakia and oral squamous cell carcinoma. Oncology Reports, 39(4), 1793–1804. https://doi.org/10.3892/or.2018.6251

Warnakulasuriya, K. A. A. S., & Johnson, N. W. (1992). Expression of p53 mutant nuclear phosphoprotein in oral carcinoma and potentially malignant oral lesions. Journal of Oral Pathology & Medicine, 21(9), 404–408. https://doi.org/10.1111/j.1600-0714.1992.tb01028.x

Weise, J. B., Rudolph, P., Heiser, A., Kruse, M. L., Hedderich, J., Cordes, C., … Görögh, T. (2008). LOXL4 is a selectively expressed candidate diagnostic antigen in head and neck cancer. European Journal of Cancer, 44(9), 1323–1331. https://doi.org/10.1016/j.ejca.2008.03.026

Wood, M. W., Medina, J. E., Thompson, G. C., Houck, J. R., & Min, K. (1994). Accumulation of the p53 Tumor‐Suppressor Gene Product in Oral Leukoplakia. Otolaryngology–Head and Neck Surgery, 111(6), 758–763. https://doi.org/10.1177/019459989411100610

Wu, F., Shi, X., Zhang, R., Tian, Y., Wang, X., Wei, C., … Zhou, H. (2018). Regulation of proliferation and cell cycle by protein regulator of cytokinesis 1 in oral squamous cell carcinoma. Cell Death and Disease, 9(5), 564. https://doi.org/10.1038/s41419-018-0618-6

Wu, P. F., Han, B. feng, Xia, H., Yan, C. ran, & Li, L. jiang. (2010). [The expression of human major histocompatibility complex-I in oral leukoplakia]. Hua Xi Kou Qiang Yi Xue Za Zhi = Huaxi Kouqiang Yixue Zazhi = West China Journal of Stomatology, 28(4), 439–442. Retrieved from https://pubmed.ncbi.nlm.nih.gov/20848943/

Xia, J., Chen, N., Hong, Y., Chen, X., Tao, X., Cheng, B., & Huang, Y. (2012). Expressions of CXCL12/CXCR4 in oral premalignant and malignant lesions. Mediators of Inflammation, 2012, 516395. https://doi.org/10.1155/2012/516395

Xia, J., Wang, J., Chen, N., Dai, Y., Hong, Y., Chen, X., & Cheng, B. (2011). Expressions of CXCR7/ligands may be involved in oral carcinogenesis. Journal of Molecular Histology, 42(2), 175–180. https://doi.org/10.1007/s10735-011-9322-x

Xu, X. C., Lotan, R., Lee, J. S., Lippman, S. M., Hong, W. K., & Ro, J. Y. (1995). Increased Expression of Cytokeratins CK8 and CK19 is Associated with Head and Neck Carcinogenesis. Cancer Epidemiology Biomarkers and Prevention, 4(8), 871–876. Retrieved from https://pubmed.ncbi.nlm.nih.gov/8634660/

Yang, L.-Q., Xiao, X., Li, C.-X., Wu, W.-Y., Shen, X.-M., Zhou, Z.-T., … Shi, L.-J. (2019). Human papillomavirus genotypes and p16 expression in oral leukoplakia and squamous cell carcinoma. International Journal of Clinical and Experimental Pathology, 12(3), 1022–1028. Retrieved from http://www.ncbi.nlm.nih.gov/pubmed/31933914%0Ahttp://www.pubmedcentral.nih.gov/articlerender.fcgi?artid=PMC6945145

Ye, X., Zhang, J., Lu, R., & Zhou, G. (2016). Signal regulatory protein α associated with the progression of oral leukoplakia and oral squamous cell carcinoma regulates phenotype switch of macrophages. Oncotarget, 7(49), 81305–81321. https://doi.org/10.18632/oncotarget.12874

Yin, F., Chen, Q., Shi, Y., Xu, H., Huang, J., Qing, M., … Zeng, X. (2022). Activation of EGFR-Aurora A induces loss of primary cilia in oral squamous cell carcinoma. Oral Diseases, 28(3), 621–630. https://doi.org/10.1111/odi.13791

Yoshida, T., Terabe, T., Nagai, H., Uchida, F., Hasegawa, S., Nagao, T., … Bukawa, H. (2019). Association between p62 expression and clinicopathological characteristics in oral leukoplakia. Clinical and Experimental Dental Research, 5(4), 389–397. https://doi.org/10.1002/cre2.193

Younes, F., Quartey, E. L., Kiguwa, S., & Partridge, M. (1996). Expression of TNF and the 55-kDa TNF receptor in epidermis, oral mucosa, lichen planus and squamous cell carcinoma. Oral Diseases, 2(1), 25–31. https://doi.org/10.1111/j.1601-0825.1996.tb00199.x

Zhang, J., Zeng, Y., Zheng, J., & Xu, J. (2013). [Expression of Prion protein and its clinical significance in oral squamous cells carcinoma and oral leukoplakia]. Zhonghua Kou Qiang Yi Xue Za Zhi = Zhonghua Kouqiang Yixue Zazhi = Chinese Journal of Stomatology, 48(12), 752–754. Retrieved from https://pubmed.ncbi.nlm.nih.gov/24495728/

Zhang, Z., Guo, W., Zhang, Y., Wang, X., Liu, H., Xu, S., … Chen, D. (2017). Changes in the expression of Col IV, gelatinase and TIMP-1 in oral leukoplakia. International Journal of Clinical and Experimental Pathology, 10(8), 8535–8543. Retrieved from https://pubmed.ncbi.nlm.nih.gov/31966707/

Zhao, X. yi, Liu, H. wei, & Wei, M. jie. (2005). Expressions of PDCD5 and p53 in oral leukoplakia and oral squamous cell carcinoma. Beijing Da Xue Xue Bao. Yi Xue Ban = Journal of Peking University. Health Sciences, 37(4), 429–432. Retrieved from https://pubmed.ncbi.nlm.nih.gov/16086069/

Zisis, V., Anastasiadou, P. A., Poulopoulos, A., Vahtsevanos, K., Paraskevopoulos, K., & Andreadis, D. (2024). A Preliminary Study of the Role of Endothelial-Mesenchymal Transitory Factor SOX 2 and CD147 in the Microvascularization of Oral Squamous Cell Carcinoma. Cureus, 16(1), e52265. https://doi.org/10.7759/cureus.52265

Zisis, V., Andreadis, D., Anastasiadou, P. A., Akrivou, M., Vizirianakis, I. S., Anagnostou, L., … Poulopoulos, A. (2023). Expression of the Embryonic Cancer Stem Cells’ Biomarkers SOX2 and OCT3/4 in Oral Leukoplakias and Squamous Cell Carcinomas: A Preliminary Study. Cureus, 15(9), e45482. https://doi.org/10.7759/cureus.45482

Zisis, V., Andreadis, D., Anastasiadou, P., Vahtsevanos, K., Akrivou, M., Vizirianakis, I. S., & Poulopoulos, A. (2023). Preliminary Study of the Cancer Stem Cells’ Biomarker CD147 in Leukoplakia: Dysplasia and Squamous Cell Carcinoma of Oral Epithelial Origin. Cureus, 15(5), e38807. https://doi.org/10.7759/cureus.38807

Zisis, V., Giannakopoulos, N. N., Schmitter, M., Poulopoulos, A., & Andreadis, D. (2023). Cancer Stem Cells’ Biomarker ALDH1&2 Increased Expression in Erosive Oral Lichen Planus Compared to Oral Leukoplakia. Cureus, 15(8), e44278. https://doi.org/10.7759/cureus.44278

Zisis, V., Paraskeuopoulos, K., Athanasios, P., Panta, P., & Dimitrios, A. (2023). Altered Presence of Cancer Stem Cell ALDH1/2 in Oral Leukoplakias and Squamous Cell Carcinomas. Cureus, 15(6), e40836. https://doi.org/10.7759/cureus.40836

**Lack of essential data (n= 205):**

Abidullah, M., Nahar, P., Ahmed, S., Kothari, H., & Vakeel, S. (2023). MUC4 expression in oral dysplastic epithelium and oral squamous cell carcinoma: An immunohistochemical study. Journal of International Society of Preventive and Community Dentistry, 13(2), 124–132. https://doi.org/10.4103/jispcd.JISPCD_241_22

Abrahao, A. C., Bonelli, B. V., Nunes, F. D., Dias, E. P., & Cabral, M. G. (2011). Immunohistochemical expression of p53, p16 and hTERT in oral squamous cell carcinoma and potentially malignant disorders. Brazilian Oral Research, 25(1), 34–41. https://doi.org/10.1590/S1806-83242011000100007

Aida, J., Kobayashi, T., Saku, T., Yamaguchi, M., Shimomura, N., Nakamura, K. I., … Takubo, K. (2012). Short telomeres in an oral precancerous lesion: Q-FISH analysis of leukoplakia. Journal of Oral Pathology and Medicine, 41(5), 372–378. https://doi.org/10.1111/j.1600-0714.2011.01120.x

Al-Tarawneh, Z. A., Pena-Cristóbal, M., Cernadas, E., Suarez-Peñaranda, J. M., Fernández-Delgado, M., Mbaidin, A., … Gándara-Vila, P. (2024). OralImmunoAnalyser: a software tool for immunohistochemical assessment of oral leukoplakia using image segmentation and classification models. Frontiers in Artificial Intelligence, 7, 1324410. https://doi.org/10.3389/frai.2024.1324410

Ankathil, R. (1997). Interphase nucleolar organizer region distribution in the tissues of oral leukoplakia, oral submucous fibrosis and oral cancer: Evaluation of diagnostic and prognostic implications. Oncology Reports, 4(1), 187–190. https://doi.org/10.3892/or.4.1.187

Barros, C. C. da S., Freitas, R. de A., Miguel, M. C. da C., & Dantas da Silveira, É. J. (2022). DNA damage through oxidative stress is an important event in oral leukoplakia. Archives of Oral Biology, 135, 105359. https://doi.org/10.1016/j.archoralbio.2022.105359

Bavle, R., Paremala, K., Venugopal, R., Rudramuni, A., Khan, N., & Hosthor, S. (2021). Grading of oral leukoplakia: Can it be improvised using immunohistochemical markers p63 and CD31. Contemporary Clinical Dentistry, 12(1), 37–43. https://doi.org/10.4103/ccd.ccd_493_19

Becker, A. S., Holm, M., Liese, J., Engel, N., & Zimpfer, A. H. (2024). Diagnosis of differentiated dysplasia as a variant of oral epithelial dysplasia. Oral Diseases. https://doi.org/10.1111/odi.14846

Beenken, S. W., Sellers, M. T., Huang, P., Peters, G., Krontiras, H., Dixon, P., … Grizzle, W. E. (1999). Transforming growth factor α (TGF-α) expression in dysplastic oral leukoplakia: Modulation by 13-cis retinoic acid. Head and Neck, 21(6), 566–573. https://doi.org/10.1002/(SICI)1097-0347(199909)21:6<566::AID-HED11>3.0.CO;2-H

Beevi, B., Nayak, S., Peter, C., Haridas, A., Jacob, L., & Aboobakker, A. (2019). Analysis of Ki-67 expression in oral premalignant lesions and normal oral mucosa: An immunohistochemical study. Journal of Pharmacy and Bioallied Sciences, 11(6), S232–S235. https://doi.org/10.4103/JPBS.JPBS_305_18

Bharti, A., Qayoom, S., Jaiswal, R., Agarwal, P., Singh, R. K., Agarwal, S. P., … Goel, M. M. (2022). Can dual staining with p16 and Ki67 be biomarkers of epithelial dysplasia in oral lesions? Journal of Cancer Research and Therapeutics, 18(4), 1003–1008. https://doi.org/10.4103/jcrt.JCRT_40_20

Bhattacharyya, S., Ray, S., Saha, D., Mustafi, S. M., Alam, N., Sarkar, A., & Murmu, N. (2021). Chewing tobacco may act as a risk factor for dysplastic transformation of squamous cells in Oral leukoplakia- A cytochemistry based approach. Pathology Research and Practice, 218, 153287. https://doi.org/10.1016/j.prp.2020.153287

Bryne, M., Reibel, J., Mandel, U., & Dabelsteen, E. (1991). Expression of mucin type carbohydrates may supplement histologic diagnosis in oral premalignant lesions. Journal of Oral Pathology & Medicine, 20(3), 120–125. https://doi.org/10.1111/j.1600-0714.1991.tb00904.x

Cai, X., Li, L., Yu, F., Guo, R., Zhou, X., Zhang, F., … Li, T. (2023). Development of a Pathomics-Based Model for the Prediction of Malignant Transformation in Oral Leukoplakia. Laboratory Investigation, 103(8), 100173. https://doi.org/10.1016/j.labinv.2023.100173

Cai, X., Zhang, J., Peng, Y., Yao, Z., Huang, J., Tang, Q., … Li, T. (2023). The preliminary exploration of immune microenvironment in oral leukoplakia concomitant with oral submucosal fibrosis: A comparative immunohistochemical study. Journal of Oral Pathology and Medicine, 52(7), 666–672. https://doi.org/10.1111/jop.13434

Camisasca, D. R., da Rós Gonçalves, L., Soares, M. R., Sandim, V., Nogueira, F. C. S., Garcia, C. H. S., … Lourenço, S. Q. C. (2017). A proteomic approach to compare saliva from individuals with and without oral leukoplakia. Journal of Proteomics, 151, 43–52. https://doi.org/10.1016/j.jprot.2016.07.029

Chandy, B., Abreo, F., Nassar, R., Stucker, F. J., & Nathan, C. A. (2002). Expression of the proto-oncogene eIF4E in inflammation of the oral cavity. Otolaryngology - Head and Neck Surgery, 126(3), 290–295. https://doi.org/10.1067/mhn.2002.123104

Chattopadhyay, A., Chawda, J. G., & Doshi, J. J. (1994). Silver-binding nucleolar organizing regions: A study of oral leukoplakia and squamous cell carcinoma. International Journal of Oral and Maxillofacial Surgery, 23(6 PART 1), 374–377. https://doi.org/10.1016/S0901-5027(05)80060-8

Chaudhari, N., Tupkari, J., Joy, T., & Ahire, M. (2016). Human MutL homolog 1 immunoexpression in oral leukoplakia and oral squamous cell carcinoma: A prospective study in Indian population. Journal of Oral and Maxillofacial Pathology, 20(3), 453–461. https://doi.org/10.4103/0973-029X.190948

Chaves, A. L. F., Silva, A. G., Maia, F. M., Lopes, G. F. M., de Paulo, L. F. B., Muniz, L. V., … de Azambuja Ribeiro, R. I. M. (2019). Reduced CD8 + T cells infiltration can be associated to a malignant transformation in potentially malignant oral epithelial lesions. Clinical Oral Investigations, 23(4), 1913–1919. https://doi.org/10.1007/s00784-018-2622-8

Chen, W., Xiao, M., Zhang, J., & Chen, W. (2018). M1-like tumor-associated macrophages activated by exosome-transferred THBS1 promote malignant migration in oral squamous cell carcinoma. Journal of Experimental and Clinical Cancer Research, 37(1), 143. https://doi.org/10.1186/s13046-018-0815-2

Chen, Y. S., Wang, J. T., Chang, Y. F., Liu, B. Y., Wang, Y. P., Sun, A., & Chiang, C. P. (2004). Expression of hepatocyte growth factor and c-met protein is significantly associated with the progression of oral squamous cell carcinoma in Taiwan. Journal of Oral Pathology and Medicine, 33(4), 209–217. https://doi.org/10.1111/j.0904-2512.2004.00118.x

Cheng, L. H. H. (2001). Ubiquitin and malignant transformation of oral mucosa. Head and Neck, 23(11), 972–978. https://doi.org/10.1002/hed.1141

Costa, N. L., Gonçalves, A. S., Martins, A. F. L., Arantes, D. A. C., Silva, T. A., & Batista, A. C. (2016). Characterization of dendritic cells in lip and oral cavity squamous cell carcinoma. Journal of Oral Pathology and Medicine, 45(6), 418–424. https://doi.org/10.1111/jop.12380

Costa, N. L., Gonçalves, A. S., Souza-Lima, N. C., Jaime-Paiva, L. G., Junqueira-Kipnis, A. P., Silva, T. A., … Batista, A. C. (2011). Distinct expression of perforin and granzyme B in lip and oral cavity squamous cell carcinoma. Journal of Oral Pathology and Medicine, 40(5), 380–384. https://doi.org/10.1111/j.1600-0714.2011.01014.x

Crawford, M., Liu, X., Cheng, Y. S. L., & Tsai, R. Y. (2021). Nucleostemin upregulation and STAT3 activation as early events in oral epithelial dysplasia progression to squamous cell carcinoma. Neoplasia (United States), 23(12), 1289–1299. https://doi.org/10.1016/j.neo.2021.11.001

Cruz, I., Meijer, C. J. L. M., Napier, S. S., Van der Waal, I., Snijders, P. J. F., Walboomers, J. M. M., … Maxwell, P. (2002). Suprabasal p53 immunoexpression is strongly associated with high grade dysplasia and risk for malignant transformation in potentially malignant oral lesions from Northern Ireland. Journal of Clinical Pathology, 55(2), 98–104. https://doi.org/10.1136/jcp.55.2.98

da Silva, A. D., Daroit, N. B., Cardoso, F. B., Laureano, N. K., Maraschin, B. J., Bündrich, L., … Rados, P. V. (2018). Epithelial oral mucosal cells: Do they behave differently when exposed to oral carcinogens? Cytopathology, 29(1), 49–57. https://doi.org/10.1111/cyt.12468

da Silva, L. C., Fonseca, F. P., de Almeida, O. P., de Almeida Mariz, B. A. L., Lopes, M. A., Radhakrishnan, R., … Vargas, P. A. (2020). CD1A+ and CD207+ cells are reduced in oral submucous fibrosis and orasquamous cell carcinoma. Medicina Oral Patologia Oral y Cirugia Bucal, 25(1), e49–e55. https://doi.org/10.4317/medoral.23177

Dabelsteen, E., & Clausen, H. (1987). Tumor‐associated carbohydrate antigens. Journal of Oral Pathology & Medicine, 16(4), 196–198. https://doi.org/10.1111/j.1600-0714.1987.tb02066.x

Dabelsteen, E., Clausen, H., Holmstrup, P., & Reibel, J. (1988). Premalignant and malignant oral lesions are associated with changes in the glycosylation pattern of carbohydrates related to ABH blood group antigens. Apmis, 96(9), 813–819. https://doi.org/10.1111/j.1699-0463.1988.tb00948.x

Dabelsteen, E., Roed‐Petersen, B., & Pindborg, J. J. (1975). Loss of Epithelial Blood Group Antigens a and B in Oral Premalignant Lesions. Acta Pathologica Microbiologica Scandinavica Section A Pathology, 83 A(3), 292–300. https://doi.org/10.1111/j.1699-0463.1975.tb01876.x

Dalley, A. J., Pitty, L. P., Major, A. G., Abdulmajeed, A. A., & Farah, C. S. (2014). Expression of ABCG2 and Bmi-1 in oral potentially malignant lesions and oral squamous cell carcinoma. Cancer Medicine, 3(2), 273–283. https://doi.org/10.1002/cam4.182

de Camargo, J. F., de F. Ribeiro, S., Rovani, G., Piardi, C., de J Freitas, V., Gambin, D. J., … De Carli, J. P. (2020). Histopathological classifications of oral leukoplakia and its relation to cell proliferative activity: A case series. Journal of Contemporary Dental Practice, 21(6), 651–656. https://doi.org/10.5005/jp-journals-10024-2840

de Carvalho Fraga, C. A., Farias, L. C., de Oliveira, M. V. M., Domingos, P. L. B., Pereira, C. S., Silva, T. F., … Guimarães, A. L. S. (2014). Increased VEGFR2 and MMP9 protein levels are associated with epithelial dysplasia grading. Pathology Research and Practice, 210(12), 959–964. https://doi.org/10.1016/j.prp.2014.06.020

de Freitas Silva, B. S., Yamamoto, F. P., Pontes, F. S. C., Cury, S. E. V., Fonseca, F. P., Pontes, H. A. R., & Décio-dos Santos, P. J. (2012). TWIST and p-Akt immunoexpression in normal oral epithelium, oral dysplasia and in oral squamous cell carcinoma. Medicina Oral, Patologia Oral y Cirugia Bucal, 17(1), e29-34. https://doi.org/10.4317/medoral.17344

de Lima, T. B., Paz, A. H. R., Rados, P. V., Leonardi, R., Bufo, P., Pedicillo, M. C., … Visioli, F. (2017). Autophagy analysis in oral carcinogenesis. Pathology Research and Practice, 213(9), 1072–1077. https://doi.org/10.1016/j.prp.2017.07.027

De Oliveira, M. G., Pereira Ramalho, L. M., Gaião, L., Pozza, D. H., & De Mello, R. A. (2012). Retinoblastoma and p53 protein expression in pre-malignant oral lesions and oral squamous cell carcinoma. Molecular Medicine Reports, 6(1), 163–166. https://doi.org/10.3892/mmr.2012.876

Ding, L., Zhao, X., Zhu, N., Zhao, M., Hu, Q., & Ni, Y. (2020). The balance of serum IL-18/IL-37 levels is disrupted during the development of oral squamous cell carcinoma. Surgical Oncology, 32, 99–107. https://doi.org/10.1016/j.suronc.2019.12.001

Ding, X., Zhang, N., Cai, Y., Li, S., Zheng, C., Jin, Y., … Zhou, X. (2012). Down-regulation of tumor suppressor MTUS1/ATIP is associated with enhanced proliferation, poor differentiation and poor prognosis in oral tongue squamous cell carcinoma. Molecular Oncology, 6(1), 73–80. https://doi.org/10.1016/j.molonc.2011.11.002

Dong, Y. Y., Wang, J., Dong, F. S., Wang, X., Zhang, Y. H., & Guo, L. H. (2006). Study of p16INK4/CDKN2 gene homozygous deletions and point mutation in squamous cell carcinoma of buccal mucosa. Hua Xi Kou Qiang Yi Xue Za Zhi = Huaxi Kouqiang Yixue Zazhi = West China Journal of Stomatology, 24(4), 362–365. Retrieved from https://pubmed.ncbi.nlm.nih.gov/16999361/

Donís, S. P., González, A. P., Alves, M. G. O., Do Carmo Carvalho, B. F., Ferreira, C. C. P., Almeida, J. D., … Sayáns, M. P. (2021). MLH1, MSH2, MRE11, and XRCC1 in Oral Leukoplakia and Oral Squamous Cell Carcinoma. Applied Immunohistochemistry and Molecular Morphology, 29(8), 613–618. https://doi.org/10.1097/PAI.0000000000000929

Donofrio, V., Lo Muzio, L., Mignogna, M. D., Troncone, G., Staibano, S., Boscaino, A., & De Rosa, G. (1995). Prognostic evaluation of HPV-associated precancerous and microinvasive Carcinoma of the Oral Cavity: Combined use of Nucleolar Organiser Regions (AgNOR) and Proliferating Cell Nuclear Antigen (PCNA). European Journal of Cancer. Part B: Oral Oncology, 31(3), 174–180. https://doi.org/10.1016/0964-1955(95)00003-Z

Dwivedi, R., Chandra, S., Mehrotra, D., Raj, V., & Pandey, R. (2020). Predicting transition from oral pre-malignancy to malignancy via Bcl-2 immuno-expression: Evidence and lacunae. Journal of Oral Biology and Craniofacial Research, 10(4), 397–403. https://doi.org/10.1016/j.jobcr.2020.07.003

Elangovan, T., Mani, N., & Malathi, N. (2008). Argyrophilic nucleolar organizer regions in inflammatory, premalignant, and malignant oral lesions: A quantitative and qualitative assessment. Indian Journal of Dental Research, 19(2), 141–146. https://doi.org/10.4103/0970-9290.40469

Embaló, B., Miguel, A. F. P., Konrath, A. C., Modolo, F., & Rivero, E. R. C. (2023). Evaluation of two classification systems for oral epithelial dysplasia. Oral Diseases, 29(1), 100–104. https://doi.org/10.1111/odi.13867

Eversole, L. R., & Philip Sapp, J. (1993). c-myc Oncoprotein expression in oral precancerous and early cancerous lesions. European Journal of Cancer. Part B: Oral Oncology, 29(2), 131–135. https://doi.org/10.1016/0964-1955(93)90035-D

Fan, G. K., Chen, J., Ping, F., & Geng, Y. (2006). Immunohistochemical analysis of P57(kip2), p53 and hsp60 expressions in premalignant and malignant oral tissues. Oral Oncology, 42(2), 147–153. https://doi.org/10.1016/j.oraloncology.2005.06.017

Farah, C. S., Jessri, M., Bennett, N. C., Dalley, A. J., Shearston, K. D., & Fox, S. A. (2019). Exome sequencing of oral leukoplakia and oral squamous cell carcinoma implicates DNA damage repair gene defects in malignant transformation. Oral Oncology, 96, 42–50. https://doi.org/10.1016/j.oraloncology.2019.07.005

Fornatora, M., Jones, A. C., Kerpel, S., & Freedman, P. (1996). Human papillomavirus-associated oral epithelial dysplasia (koilocytic dysplasia) An entity of unknown biologic potential. Oral Surgery, Oral Medicine, Oral Pathology, Oral Radiology, and Endodontics, 82(1), 47–56. https://doi.org/10.1016/s1079-2104(96)80377-5

Fregonezi, P. A. G., Silva, T. G. A., Simões, R. T., Moreau, P., Carosella, E. D., Kläy, C. P. M., … Soares, C. P. (2012). Expression of nonclassical molecule human leukocyte antigen-G in oral lesions. American Journal of Otolaryngology - Head and Neck Medicine and Surgery, 33(2), 193–198. https://doi.org/10.1016/j.amjoto.2010.08.001

Garg, K. N., Raj, V., & Chandra, S. (2015). Efficacy of argyrophilic nucleolar organizing region analysis using computer-assisted and manual in oral leukoplakia: A comparative study. Indian Journal of Cancer, 52(1), 75–79. https://doi.org/10.4103/0019-509X.175589

Garzino-Demo, P., Carrozzo, M., Trusolino, L., Savoia, P., Gandolfo, S., & Marchisio, P. C. (1998). Altered expression of α6 integrin subunit in oral squamous cell carcinoma and oral potentially malignant lesions. Oral Oncology, 34(3), 204–210. https://doi.org/10.1016/S1368-8375(97)00059-6

Gassenmaier, A., & Hornstein, O. P. (1988). Presence of human papillomavirus DNA in benign and precancerous oral leukoplakias and squamous cell carcinomas. Dermatologica, 176(5), 224–233. https://doi.org/10.1159/000248710

Georgaki, M., Avgoustidis, D., Theofilou, V. I., Piperi, E., Pettas, E., Kalyvas, D. G., … Nikitakis, N. G. (2021). Recurrence in oral premalignancy: Clinicopathologic and immunohistochemical analysis. Diagnostics, 11(5). https://doi.org/10.3390/diagnostics11050872

Gilchrist, J. M., Thompson, G. C., & Medina, J. E. (1992). Markers of keratinocyte differentiation in snuff-induced leukoplakia. The American Journal of Surgery, 164(6), 563–566. https://doi.org/10.1016/S0002-9610(05)80707-X

Gires, O., MacK, B., Rauch, J., & Matthias, C. (2006). CK8 correlates with malignancy in leukoplakia and carcinomas of the head and neck. Biochemical and Biophysical Research Communications, 343(1), 252–259. https://doi.org/10.1016/j.bbrc.2006.02.139

Girod, S. C., Pfeiffer, P., Ries, J., & Pape, H. D. (1998). Proliferative activity and loss of function of tumour suppressor genes as “biomarkers” in diagnosis and prognosis of benign and preneoplastic oral lesions and oral squamous cell carcinoma. British Journal of Oral and Maxillofacial Surgery, 36(4), 252–260. https://doi.org/10.1016/S0266-4356(98)90708-2

Godge, P. Y., & Poonja, L. S. (2011). Quantitative assessment of expression of cell adhesion molecule (CD44) splice variants: CD44 standard (CD44s) and v5, v6 isoforms in oral leukoplakias: An immunohistochemical study. Indian Journal of Dental Research, 22(3), 506–513. https://doi.org/10.4103/0970-9290.87080

Gonçalves, A. S., Mosconi, C., Jaeger, F., Wastowski, I. J., Aguiar, M. C. F., Silva, T. A., … Batista, A. C. (2017). Overexpression of immunomodulatory mediators in oral precancerous lesions. Human Immunology, 78(11–12), 752–757. https://doi.org/10.1016/j.humimm.2017.09.003

Gonzalez Segura, I., Secchi, D., Carrica, A., Barello, R., Arbelo, D., Burgos, A., … Zarate, A. M. (2015). Exfoliative cytology as a tool for monitoring pre-malignant and malignant lesions based on combined stains and morphometry techniques. Journal of Oral Pathology and Medicine, 44(3), 178–184. https://doi.org/10.1111/jop.12219

Guo, Z. L., & Gao, Y. (2010). [Expression of integrin-linked kinase in oral leukoplakia and early invasive carcinoma]. Zhonghua Kou Qiang Yi Xue Za Zhi = Zhonghua Kouqiang Yixue Zazhi = Chinese Journal of Stomatology, 45(3), 163–167. Retrieved from https://pubmed.ncbi.nlm.nih.gov/20450685/

Gupta, V., Ramalingam, K., Yasothkumar, D., Debnath, D., & Sundar, V. (2023). Ki-67 Expression as a Prognostic Marker: A Comparative Immunohistochemical Analysis of Oral Epithelial Dysplasia and Oral Squamous Cell Carcinoma. Cureus, 15(5), e38941. https://doi.org/10.7759/cureus.38941

Hamadah, O., Goodson, M. L., & Thomson, P. J. (2010). Clinicopathological behaviour of multiple oral dysplastic lesions compared with that of single lesions. British Journal of Oral and Maxillofacial Surgery, 48(7), 503–506. https://doi.org/10.1016/j.bjoms.2009.08.027

Haraguchi, K., Yada, N., Sato, S., Habu, M., Hayakawa, M., Takahashi, O., … Tominaga, K. (2017). The methylation status and expression of human telomerase reverse transcriptase is significantly high in oral carcinogenesis. Apmis, 125(9), 797–807. https://doi.org/10.1111/apm.12723

Hartmann, S., Kipke, R. U. N., Rauthe, S., Mutzbauer, G., Brands, R. C., Ebhardt, H., … Müller-Richter, U. D. A. (2015). Oral brush biopsy and melanoma-associated antigens A (MAGE-A) staining in clinically suspicious lesions. Journal of Cranio-Maxillofacial Surgery, 43(10), 2214–2218. https://doi.org/10.1016/j.jcms.2015.10.018

Heyden, A., Huitfeldt, H. S., Koppang, H. S., Thrane, P. S., Bryne, M., & Brandtzaeg, P. (1992). Cytokeratins as epithelial differentiation markers in premalignant and malignant oral lesions. Journal of Oral Pathology & Medicine, 21(1), 7–11. https://doi.org/10.1111/j.1600-0714.1992.tb00960.x

Hu, S., Lu, H., Xie, W., Wang, D., Shan, Z., Xing, X., … Wang, Z. (2022). TDO2+ myofibroblasts mediate immune suppression in malignant transformation of squamous cell carcinoma. Journal of Clinical Investigation, 132(19). https://doi.org/10.1172/JCI157649

I, A., Raghavan Pillai, V. B., P. Joseph, A., Ramani, P., P, J., & Ramalingam, K. (2024). Identification and Evaluation of Cancer Stem Cells in Oral Squamous Cell Carcinoma and Oral Epithelial Dysplasia Using NANOG: An Immunohistochemical Study. Cureus, 16(2), e55111. https://doi.org/10.7759/cureus.55111

Iamaroon, A., Khemaleelakul, U., Pongsiriwet, S., & Pintong, J. (2004). Co-expression of p53 and Ki67 and lack of EBV expression in oral squamous cell carcinoma. Journal of Oral Pathology and Medicine, 33(1), 30–36. https://doi.org/10.1111/j.1600-0714.2004.00192.x

Jham, B. C., Costa, N. L., Silva, J. M., de Miranda, A. C., Oliveira, J. C., Silva, T. A., & Batista, A. C. (2012). Midkine expression in oral squamous cell carcinoma and leukoplakia. Journal of Oral Pathology and Medicine, 41(1), 21–26. https://doi.org/10.1111/j.1600-0714.2011.01049.x

Jiang, Y., Wu, Y., & Wang, F. (1996). Histochemical study of lectin receptor and cell morphometric analysis on oral leukoplakias, epithelial dysplasia and squamous carcinoma. Zhonghua Kou Qiang Yi Xue Za Zhi = Zhonghua Kouqiang Yixue Zazhi = Chinese Journal of Stomatology, 31(6), 351–353. Retrieved from https://pubmed.ncbi.nlm.nih.gov/9592290/

Jimson, S., Murali, S., Zunt, S., Goldblatt, L., & Srinivasan, M. (2016). Epithelial expression of keratinocytes growth factor in oral precancer lesions. Dental Research Journal, 13(3), 199–205. https://doi.org/10.4103/1735-3327.182148

John, S., Joseph, A. P., Raghavan Pillai, V. B., Ramani, P., P, J., & Ramalingam, K. (2024). Evaluation of Cytotoxic T Lymphocytes and Natural Killer Cell Distribution in Oral Squamous Cell Carcinoma and Oral Epithelial Dysplasia: An Immunohistochemical Study. Cureus, 16(3), e56323. https://doi.org/10.7759/cureus.56323

Kahn, M. A., Mincer, H. H., Dockter, M. E., & Hermann‐Petrin, J. M. (1993). Comparing flow cytometric analysis and nucleolar organizer region enumeration in archival oral premalignant lesions. Journal of Oral Pathology & Medicine, 22(6), 257–262. https://doi.org/10.1111/j.1600-0714.1993.tb01067.x

Kannan, S., Balaram, P., Chandran, G. J., Pillai, M. R., Mathew, B., Nalinakumari, K. R., & Nair, M. K. (1994). Alterations in expression of terminal differentiation markers of keratinocytes during oral carcinogenesis. Pathobiology, 62(3), 127–133. https://doi.org/10.1159/000163889

Kannan, S., Jagadeesh Chandran, G., Balaram, P., Chidambaram, S., & Krishnan Nair, M. (1996). Potential biological markers for the staging of tumor progression in oral mucosa: A multivariate analysis. International Journal of Biological Markers, 11(2), 67–76. https://doi.org/10.1177/172460089601100202

KC, S., RD, A., D, S., & G, P. (2014). Oxidant-antioxidant status in tissue samples of oral leukoplakia. Dental Research Journal, 11(2), 180–186. Retrieved from http://europepmc.org/articles/PMC4052642

Kearsley, J. H., Stenzel, D. J., Sculley, T. B., & Cooke, R. A. (1990). Cellular localisation of tumour antigen (Ta-4) in normal, dysplastic and neoplastic squamous epithelia of the upper aerodigestive tract. British Journal of Cancer, 61(4), 631–635. https://doi.org/10.1038/bjc.1990.140

Khushbu, B., Chalishazar, M., Kale, H., Baranwal, M., & Modi, T. (2017). Quantitative and qualitative assessment of argyrophilic nucleolar organizer regions in normal, premalignant and malignant oral lesions. Journal of Oral and Maxillofacial Pathology, 21(3), 360–366. https://doi.org/10.4103/jomfp.JOMFP_52_15

Kiyosue, T., Kawano, S., Matsubara, R., Goto, Y., Hirano, M., Jinno, T., … Nakamura, S. (2013). Immunohistochemical location of the p75 neurotrophin receptor (p75NTR) in oral leukoplakia and oral squamous cell carcinoma. International Journal of Clinical Oncology, 18(1), 154–163. https://doi.org/10.1007/s10147-011-0358-4

Klein, I. P., Meurer, L., Danilevicz, C. K., Squarize, C. H., Martins, M. D., & Carrard, V. C. (2020). BML-1 expression increases in oral leukoplakias and correlates with cell proliferation. Journal of Applied Oral Science, 28, 1–10. https://doi.org/10.1590/1678-7757-2019-0532

Kokubun, K., Nakajima, K., Akashi, Y., Yamamoto, K., Katakura, A., & Matsuzaka, K. (2024). Clinicopathological evaluation of oral leukoplakia: a single-center study of 676 cases in Japan. Oral Surgery, Oral Medicine, Oral Pathology and Oral Radiology, 137(5), 529–536. https://doi.org/10.1016/j.oooo.2024.02.022

Kövesi, G., & Szende, B. (2003). Changes in apoptosis and mitotic index, p53 and Ki67 expression in various types of oral leukoplakia. Oncology, 65(4), 331–336. https://doi.org/10.1159/000074646

Kövesi, G., & Szende, B. (2006). Prognostic value of cyclin D1, p27, and p63 in oral leukoplakia. Journal of Oral Pathology and Medicine, 35(5), 274–277. https://doi.org/10.1111/j.1600-0714.2006.00396.x

Krauss, E., Rauthe, S., Gattenlöhner, S., Reuther, T., Kochel, M., Kriegebaum, U., … Müller-Richter, U. D. A. (2011). MAGE-A antigens in lesions of the oral mucosa. Clinical Oral Investigations, 15(3), 315–320. https://doi.org/10.1007/s00784-010-0387-9

Kühn, J. P., Speicher, S., Linxweiler, B., Körner, S., Rimbach, H., Wagner, M., … Linxweiler, M. (2024). Dual Sec62/Ki67 immunocytochemistry of liquid-based cytological preparations represents a highly valid biomarker for non-invasive detection of head and neck squamous cell carcinomas. Cytopathology, 35(1), 113–121. https://doi.org/10.1111/cyt.13310

Kujan, O., Siddiqui, I., Lee, C., Idrees, M., Shearston, K., & Farah, C. S. (2023). Automated immunohistochemical quantification of hypoxia biomarkers shows correlation with dysplastic epithelial changes. Journal of Oral Pathology and Medicine, 52(6), 504–513. https://doi.org/10.1111/jop.13427

Kulkarni, S., Solomon, M., Chandrashekar, C., Shetty, N., & Carnelio, S. (2020). Spalt-like transcription factor 4 expression in oral epithelial dysplasia and oral squamous cell carcinoma: An immunohistochemical appraisal. Journal of Carcinogenesis, 19(1), 12. https://doi.org/10.4103/jcar.jcar_13_20

Kumar, P., Kane, S., & Rathod, G. P. (2012). Coexpression of p53 and Ki 67 and lack of c-erbB2 expression in oral leukoplakias in India. Brazilian Oral Research, 26(3), 228–234. https://doi.org/10.1590/S1806-83242012000300008

Kuo, M. Y. P., Cheng, S. J., Chen, H. M., Kok, S. H., Hahn, L. J., & Chiang, C. P. (1998). Expression of CD44s, CD44v5, CD44v6 and CD44v7-8 in betel quid chewing-associated oral premalignant lesions and squamous cell carcinomas in Taiwan. Journal of Oral Pathology and Medicine, 27(9), 428–433. https://doi.org/10.1111/j.1600-0714.1998.tb01980.x

Larsson, L., Jäwert, F., Magnusson, B., Hasséus, B., & Kjeller, G. (2013). Expression of high mobility group A proteins in oral leukoplakia. Anticancer Research, 33(10), 4261–4266. Retrieved from https://pubmed.ncbi.nlm.nih.gov/24122990/

Li, M., Jin, S., Zhang, Z., Ma, H., & Yang, X. (2022). Interleukin-6 facilitates tumor progression by inducing ferroptosis resistance in head and neck squamous cell carcinoma. Cancer Letters, 527, 28–40. https://doi.org/10.1016/j.canlet.2021.12.011

Li, R., Zhang, R., Shi, X., Jiao, X., Li, Y., Zhao, Y., … Zhang, C. (2024). Expression of FAP in Oral Leukoplakia and Oral Squamous Cell Carcinoma. International Dental Journal, 74(3), 581–588. https://doi.org/10.1016/j.identj.2023.12.011

Li, S., Yang, Y., Ding, Y., Tang, X., & Sun, Z. (2017). Impacts of survivin and caspase-3 on apoptosis and angiogenesis in oral cancer. Oncology Letters, 14(3), 3774–3779. https://doi.org/10.3892/ol.2017.6626

Li, W., Han, Y., Zhao, Z., Ji, X., Wang, X., Jin, J., … Liu, H. (2019). Oral mucosal mesenchymal stem cell-derived exosomes: A potential therapeutic target in oral premalignant lesions. International Journal of Oncology, 54(5), 1567–1578. https://doi.org/10.3892/ijo.2019.4756

Lin, C. Y., Chen, W. H., Liao, C. T., Chen, I. H., Chiu, C. C., Wang, H. M., … Cheng, A. J. (2010). Positive association of glucose-regulated protein 78 during oral cancer progression and the prognostic value in oral precancerous lesions. Head and Neck, 32(8), 1028–1039. https://doi.org/10.1002/hed.21287

Liu, S. C., Sauter, E. R., Clapper, M. L., Feldman, R. S., Levin, L., Chen, S. Y., … Klein-Szanto, A. J. P. (1998). Markers of cell proliferation in normal epithelia and dysplastic leukoplakias of the oral cavity. Cancer Epidemiology Biomarkers and Prevention, 7(7), 597–603. Retrieved from https://pubmed.ncbi.nlm.nih.gov/9681528/

Liu, S., Ye, D., Wang, T., Guo, W., Song, H., Liao, Y., … Deng, J. (2017). Repression of GPRC5A is associated with activated STAT3, which contributes to tumor progression of head and neck squamous cell carcinoma. Cancer Cell International, 17(1), 34. https://doi.org/10.1186/s12935-017-0406-x

Lo Muzio, L., Mignogna, M. D., Staibano, S., De Vico, G., Salvatore, G., Damiano, S., … De Rosa, G. (1997). Morphometric study of nucleolar organiser regions (AgNOR) in HPV- associated precancerous lesions and microinvasive carcinoma of the oral cavity. European Journal of Cancer Part B: Oral Oncology, 33(4), 247–259. https://doi.org/10.1016/S0964-1955(97)00003-1

Lopez-Labady, J., Bologna-Molina, R., & Villarroel-Dorrego, M. (2021). Expression of Interleukin-1ß and Interleukin-8 in Oral Potentially Malignant Disorders and Carcinomas. Frontiers in Oral Health, 2, 649406. https://doi.org/10.3389/froh.2021.649406

Lu, Y., Li, L., Li, J., Wang, M., Yang, J., Zhang, M., … Tang, X. (2024). Prx1/PHB2 axis mediates mitophagy in oral leukoplakia cellular senescence. Pathology Research and Practice, 260, 155411. https://doi.org/10.1016/j.prp.2024.155411

Lucio, M., Andrea, G., Bartolomeo, G. D., Fabio, C., & Dora, S. (2013). Between-Lesion Discrepancies in Terms of Dysplasia, Cell Turnover and Diagnosis in Patients with Multiple Potentially Malignant Oral Lesions. The Open Dentistry Journal, 7(1), 169–174. https://doi.org/10.2174/1874210601307010169

Ma, N., Tagawa, T., Hiraku, Y., Murata, M., Ding, X., & Kawanishi, S. (2006). 8-Nitroguanine formation in oral leukoplakia, a premalignant lesion. Nitric Oxide - Biology and Chemistry, 14(2 SPEC. ISS.), 137–143. https://doi.org/10.1016/j.niox.2005.09.012

Madan, M., Chandra, S., Raj, V., & Madan, R. (2015). Evaluation of cell proliferation in malignant and potentially malignant oral lesions. Journal of Oral and Maxillofacial Pathology, 19(3), 297–305. https://doi.org/10.4103/0973-029X.174613

Mansoor Samadi, F., Thattil Sebastian, B., Singh, A., Chandra, S., Mohammad, S., Singh, A., … Samadi, F. (2014).  Silver Binding Nucleolar Organizer Regions Dots in Oral Leukoplakia with Epithelial Dysplasia and Oral Squamous Cell Carcinoma: An In Vivo Study . ISRN Dentistry, 2014, 1–4. https://doi.org/10.1155/2014/479187

Marincsák, R., Tóth, B., Czifra, G., Márton, I., Rédl, P., Tar, I., … Bíró, T. (2009). Increased expression of TRPV1 in squamous cell carcinoma of the human tongue. Oral Diseases, 15(5), 328–335. https://doi.org/10.1111/j.1601-0825.2009.01526.x

Mashhadiabbas, F., Fayazi-Boroujeni, M., Alizadeh, A., Namdari, M., & Mirzaei, S. A. (2021). Immunoexpression of C4 Binding Protein in Oral Leukoplakia and Oral Squamous Cell Carcinoma. Iranian Journal of Immunology, 18(2), 95–102. https://doi.org/10.22034/iji.2021.87031.1782

Matta, A., Tripathi, S. C., DeSouza, L. V., Grigull, J., Kaur, J., Chauhan, S. S., … Siu, K. W. M. (2009). Heterogeneous ribonucleoprotein K is a marker of oral leukoplakia and correlates with poor prognosis of squamous cell carcinoma. International Journal of Cancer, 125(6), 1398–1406. https://doi.org/10.1002/ijc.24517

Meyer, J. R., Silverman, S., Daniels, T. E., Kramer, R. H., & Greenspan, J. S. (1985). Distribution of fibronectin and laminin in oral leukoplakia and carcinoma. Journal of Oral Pathology & Medicine, 14(3), 247–255. https://doi.org/10.1111/j.1600-0714.1985.tb00488.x

Michailidou, E. ., Markopoulos, A. ., & Antoniades, D. . (2008). Mast Cells and Angiogenesis in Oral Malignant and Premalignant Lesions. The Open Dentistry Journal, 2(1), 126–132. https://doi.org/10.2174/1874210600802010126

Michailidou, E. Z., Markopoulos, A. K., & Antoniades, D. Z. (2012). VEGF expression from human dysplastic or malignant oral epithelium may be related to mast cell density and the subsequent angiogenetic phenomena. International Journal of Oral and Maxillofacial Surgery, 41(12), 1467–1473. https://doi.org/10.1016/j.ijom.2011.12.038

Migliorati, C. A., Ranken, R., Kaplan, M. J., & Silverman, S. (1992). Reactivity of monoclonal antibodies 17.13 and 63.12 with 141 oral mucosal lesions. Journal of Oral Pathology & Medicine, 21(9), 412–417. https://doi.org/10.1111/j.1600-0714.1992.tb01030.x

Miguel, A. F. P., Embaló, B., Alves Dias, H. B., & Rivero, E. R. C. (2021). Immunohistochemical expression of MMP-9, TIMP-1, and vimentin and its correlation with inflammatory reaction and clinical parameters in oral epithelial dysplasia. Applied Immunohistochemistry and Molecular Morphology, 29(5), 382–389. https://doi.org/10.1097/PAI.0000000000000910

Mishra, R., & Das, B. R. (2009). Cyclin D1 expression and its possible regulation in chewing tobacco mediated oral squamous cell carcinoma progression. Archives of Oral Biology, 54(10), 917–923. https://doi.org/10.1016/j.archoralbio.2009.07.003

Mohanraj, R., Ramani, P., Premkumar, P., Natesan, A., Sherlin, H., & Sukumaran, G. (2017). Immunohistochemical expression of ezrin in oral potentially malignant disorders-A descriptive study. Journal of Pharmacy and Bioallied Sciences, 9(5), S205–S210. https://doi.org/10.4103/jpbs.JPBS_139_17

Moraes, J. K. de, Wagner, V. P., Fonseca, F. P., Amaral-Silva, G. K. do, de Farias, C. B., Pilar, E. F. S., … Martins, M. D. (2019). Activation of BDNF/TrkB/Akt pathway is associated with aggressiveness and unfavorable survival in oral squamous cell carcinoma. Oral Diseases, 25(8), 1925–1936. https://doi.org/10.1111/odi.13190

Mori, K., Haraguchi, S., Hiori, M., Shimada, J., & Ohmori, Y. (2015). Tumor-associated macrophages in oral premalignant lesions coexpress CD163 and STAT1 in a Th1-dominated microenvironment. BMC Cancer, 15(1), 573. https://doi.org/10.1186/s12885-015-1587-0

Mozaffari, M. S., & Abdelsayed, R. (2021). Expression Profiles of GILZ and SGK-1 in Potentially Malignant and Malignant Human Oral Lesions. Frontiers in Oral Health, 2, 675288. https://doi.org/10.3389/froh.2021.675288

Muniz, J. M., Bibiano Borges, C. R., Beghini, M., de Araújo, M. S., Miranda Alves, P., de Lima, L. M. B., … Rodrigues, D. B. R. (2015). Galectin-9 as an important marker in the differential diagnosis between oral squamous cell carcinoma, oral leukoplakia and oral lichen planus. Immunobiology, 220(8), 1006–1011. https://doi.org/10.1016/j.imbio.2015.04.004

Murti, P. R., Warnakulasuriya, K. A. A. S., Johnson, N. W., Bhonsle, R. B., Gupta, P. C., Daftary, D. K., & Mehta, F. S. (1998). P53 Expression in Oral Precancer As a Marker for Malignant Potential. Journal of Oral Pathology and Medicine, 27(5), 191–196. https://doi.org/10.1111/j.1600-0714.1998.tb01940.x

Naga, S. K. S. V., Shekar, P. C., Kattappagari, K. K., Chandra, K. L. P., Reddy, G. S., & Reddy, B. V. R. (2019). Expression of cluster differentiation-44 stem cell marker in grades of oral epithelial dysplasia: A preliminary study. Journal of Oral and Maxillofacial Pathology, 23(2), 203–207. https://doi.org/10.4103/jomfp.JOMFP_308_18

Nararyanan, R. C., Sebastian, B. T., Sulaikha, S. H., Augustine, C., Thomas, T., & Sudhakaran, A. (2024). Computer-Assisted Morphometric Comparative Analysis of Argyrophilic Nucleolar Organizer Regions (AgNORs) in Leukoplakia With Dysplasia and Oral Squamous Cell Carcinoma. Cureus, 16(2), e54471. https://doi.org/10.7759/cureus.54471

Nogami, T., Kuyama, K., & Yamamoto, H. (2003). Histopathological and immunohistochemical study of malignant transformation of oral leukoplakia, with special reference to apoptosis-related gene products and proliferative activity. Acta Oto-Laryngologica, 123(6), 767–775. https://doi.org/10.1080/00016480310000700b

Novack, R., Zhang, L., Hoang, L. N., Kadhim, M., Ng, T. L., Poh, C. F., & Kevin Ko, Y. C. (2023). Abnormal p53 Immunohistochemical Patterns Shed Light on the Aggressiveness of Oral Epithelial Dysplasia. Modern Pathology, 36(7), 100153. https://doi.org/10.1016/j.modpat.2023.100153

Ogura, I., Amagasa, T., Fujii, E., & Yoshimasu, H. (1998). Quantitative evaluation of consistency of normal mucosa, leukoplakia and squamous cell carcinoma of the tongue. Journal of Cranio-Maxillo-Facial Surgery, 26(2), 107–111. https://doi.org/10.1016/S1010-5182(98)80049-8

Öhman, J., Magnusson, B., Telemo, E., Jontell, M., & Hasséus, B. (2012). Langerhans Cells and T Cells Sense Cell Dysplasia in Oral Leukoplakias and Oral Squamous Cell Carcinomas - Evidence for Immunosurveillance. Scandinavian Journal of Immunology, 76(1), 39–48. https://doi.org/10.1111/j.1365-3083.2012.02701.x

Öhman, Jenny, Mowjood, R., Larsson, L., Kovacs, A., Magnusson, B., Kjeller, G., … Hasseus, B. (2015). Presence of CD3-positive t-cells in oral premalignant leukoplakia indicates prevention of cancer transformation. Anticancer Research, 35(1), 311–318. Retrieved from https://pubmed.ncbi.nlm.nih.gov/25550565/

Palaçon, M. P., de Oliveira Barbeiro, C., Fernandes, D., Biancardi, M. R., Silveira, H. A., Ferrisse, T. M., … Bufalino, A. (2023). Macrophages CD163+ and Factor XIIIa+ Provide a First-Line Defence against Proliferative Verrucous Leukoplakia Antigens. International Journal of Molecular Sciences, 24(6). https://doi.org/10.3390/ijms24065242

Patlolla, P., N Shyam, N., Kumar, G., Narayen, V., Konda, P., & Mudududla, P. (2020). Evaluation of glucose transporter-1 expression in oral epithelial dysplasia and oral squamous cell carcinoma: An immunohistochemical study. Journal of Oral and Maxillofacial Pathology, 24(3), 578–579. https://doi.org/10.4103/jomfp.JOMFP_314_19

Pellicioli, A. C. A., Bingle, L., Farthing, P., Lopes, M. A., Martins, M. D., & Vargas, P. A. (2017). Immunosurveillance profile of oral squamous cell carcinoma and oral epithelial dysplasia through dendritic and T-cell analysis. Journal of Oral Pathology and Medicine, 46(10), 928–933. https://doi.org/10.1111/jop.12597

Pérez-Sayáns, M., Suárez-Peñaranda, J. M., Torres-López, M., Supuran, C. T., Gándara-Vila, P., Gayoso-Diz, P., … García-García, A. (2015). The use of CA-IX as a diagnostic method for oral leukoplakia. Biotechnic and Histochemistry, 90(2), 124–131. https://doi.org/10.3109/10520295.2014.965276

Pillai, K. R., Remani, P., Kannan, S., Sujathan, K., Mathew, B., Vijayakumar, T., … Menon, V. P. (1996). Lectin histochemistry of oral premalignant and malignant lesions: Correlation of JFL and PNA binding pattern with tumour progression. European Journal of Cancer Part B: Oral Oncology, 32(1), 32–37. https://doi.org/10.1016/0964-1955(95)00051-8

Pillai, K. Raveendran, Kannan, S., Sujathan, K., Madhavan, J., & Abraham, E. K. (2005). Significance of silver-stained nucleolar organizer regions in early diagnosis and prognosis of oral squamous cell carcinoma: A multivariate analysis. In Vivo, 19(4), 807–812. Retrieved from https://pubmed.ncbi.nlm.nih.gov/16001464/

Poell, J. B., Wils, L. J., Brink, A., Dietrich, R., Krieg, C., Velleuer, E., … Brakenhoff, R. H. (2023). Oral cancer prediction by noninvasive genetic screening. International Journal of Cancer, 152(2), 227–238. https://doi.org/10.1002/ijc.34277

Pokala, A., Paramkusam, G., Tejasvi, M. L. A., Bangi, B. B., Nadendla, L. K., & Devulapalli, R. V. (2020). Histo-blood group antigens in oral cancer and potentially malignant disorders. Asian Pacific Journal of Cancer Prevention, 21(4), 1163–1166. https://doi.org/10.31557/APJCP.2020.21.4.1163

Pontes, H. A.R., De Aquino Xavier, F. C., Da Silva, T. S. P., Fonseca, F. P., Paiva, H. B., Pontes, F. S. C., & Dos Santos Pinto, D. (2009). Metallothionein and p-Akt proteins in oral dysplasia and in oral squamous cell carcinoma: An immunohistochemical study. Journal of Oral Pathology and Medicine, 38(8), 644–650. https://doi.org/10.1111/j.1600-0714.2009.00787.x

Pontes, Hélder Antônio Rebelo, Pontes, F. S. C., Fonseca, F. P., De Carvalho, P. L., Pereira, É. M., De Abreu, M. C., … Dos Santos Pinto, D. (2013). Nuclear factor κb and cyclooxygenase-2 immunoexpression in oral dysplasia and oral squamous cell carcinoma. Annals of Diagnostic Pathology, 17(1), 45–50. https://doi.org/10.1016/j.anndiagpath.2012.04.008

Prado, S. M. D., Cedrún, J. L. L., Rey, R. L., Villaamil, V. M., García, A. Á., Ayerbes, M. V., & Aparicio, L. A. (2010). Evaluation of COX-2, EGFR, and p53 as biomarkers of non-dysplastic oral leukoplakias. Experimental and Molecular Pathology, 89(2), 197–203. https://doi.org/10.1016/j.yexmp.2010.06.004

PRIME, S. S., PITIGALA‐ARACHCHI, A., CRANE, I. J., ROSSER, T. J., & SCULLY, C. (1987). The expression of cell surface MHC class I heavy and light chain molecules in pre‐malignant and malignant lesions of the oral mucosa. Histopathology, 11(1), 81–91. https://doi.org/10.1111/j.1365-2559.1987.tb02611.x

Qi, M., Li, L., Lu, Y., Chen, H., Zhang, M., Wang, M., … Tang, X. (2019). Proteome profiling to identify peroxiredoxin 1 interacting protein partners in nicotine-associated oral leukoplakia. Archives of Oral Biology, 108, 104537. https://doi.org/10.1016/j.archoralbio.2019.104537

Qi, M., Li, L., Tang, X., Lu, Y., Wang, M., Yang, J., & Zhang, M. (2021). Nicotine promotes the development of oral leukoplakia via regulating peroxiredoxin 1 and its binding proteins. Brazilian Journal of Medical and Biological Research, 54(9), e10931. https://doi.org/10.1590/1414-431X2020E10931

Qiao, B., Huang, J., Mei, Z., Lam, A. K. yin, Zhao, J., & Ying, L. (2020). Analysis of Immune Microenvironment by Multiplex Immunohistochemistry Staining in Different Oral Diseases and Oral Squamous Cell Carcinoma. Frontiers in Oncology, 10, 555757. https://doi.org/10.3389/fonc.2020.555757

Rajanna, V. R., Raveendranath, M. C., Kathiresan, S., Srinivasan, S., & Ilango, J. (2020). Expression of survivin in oral potentially malignant disorders: An immunohistochemical study. Journal of Pharmacy and Bioallied Sciences, 12(5), S382–S393. https://doi.org/10.4103/jpbs.JPBS_114_20

Ramsridhar, S., & Narasimhan, M. (2016). Immunohistochemical evaluation of mast cells in leukoplakia and oral squamous cell carcinoma. Journal of Clinical and Diagnostic Research, 10(8), ZC100–ZC103. https://doi.org/10.7860/JCDR/2016/19297.8334

Rao, D. S., Ali, I. M., & Annigeri, R. G. (2017). Evaluation of diagnostic value of AgNOR and PAP in early detection of dysplastic changes in leukoplakia and lichen planus – a preliminary case–control study. Journal of Oral Pathology and Medicine, 46(1), 56–60. https://doi.org/10.1111/jop.12457

Raveendran Pillai, K., Sujathan, K., Kannan, S., Abraham, E. K., Mathew, B., Sreedevi Amma, N., … Menon, V. P. (1994). Argyrophilic nucleolar organizer regions in the evaluation of tumour progression in the oral mucosa: correlation with tissue pathology. Journal of Cancer Research and Clinical Oncology, 120(12), 723–726. https://doi.org/10.1007/BF01194270

Renkonen, J., Wolff, H., & Paavonen, T. (2002). Expression of cyclo-oxygenase-2 in human tongue carcinoma and its precursor lesions. Virchows Archiv, 440(6), 594–597. https://doi.org/10.1007/s00428-002-0616-y

Ries, J., Agaimy, A., Wehrhan, F., Baran, C., Bolze, S., Danzer, E., … Weber, M. (2021). Importance of the pd-1/pd-l1 axis for malignant transformation and risk assessment of oral leukoplakia. Biomedicines, 9(2), 1–26. https://doi.org/10.3390/biomedicines9020194

Sahoo, P., Kumar, H., Panda, A., Dash, K. C., Bhuyian, L., Raghuvanshi, M., & Panda, B. (2024). Expression of CD44 and SALL4 in Leukoplakia and Oral Squamous Cell Carcinoma. Journal of Pharmacy and Bioallied Sciences, 16(Suppl 2), S1761–S1763. https://doi.org/10.4103/jpbs.jpbs_1178_23

Santhi, W. S., Sebastian, P., Varghese, B. T., Prakash, O., & Pillai, M. R. (2006). NF-κB and COX-2 during oral tumorigenesis and in assessment of minimal residual disease in surgical margins. Experimental and Molecular Pathology, 81(2), 123–130. https://doi.org/10.1016/j.yexmp.2006.05.001

Santos García, A., Abad Hernández, M. M., Fonseca Sánchez, E., Gonzalez, R. J., Galindo Villardón, P., Cruz Hernández, J. J., & Bullón Sopelana, A. (2006). E-cadherin, laminin and collagen IV expression in the evolution from dysplasia to oral squamous cell carcinoma. Medicina Oral, Patologia Oral y Cirugia Bucal, 11(2), 69–74. Retrieved from https://pubmed.ncbi.nlm.nih.gov/16505783/

Sathasivam, H. P., Nayar, D., Sloan, P., Thomson, P. J., Odell, E. W., & Robinson, M. (2021). Dysplasia and DNA ploidy to prognosticate clinical outcome in oral potentially malignant disorders. Journal of Oral Pathology and Medicine, 50(2), 200–209. https://doi.org/10.1111/jop.13121

Sathyakumar, M., Sriram, G., Saraswathi, T. R., & Sivapathasundharam, B. (2012). Immunohistochemical evaluation of mast cells and vascular endothelial proliferation in oral precancerous lesion-leukoplakia. Journal of Oral and Maxillofacial Pathology, 16(3), 343–348. https://doi.org/10.4103/0973-029X.102481

Schaaij-Visser, T. B. M., Bremmer, J. F., Braakhuis, B. J. M., Heck, A. J. R., Slijper, M., van der Waal, I., & Brakenhoff, R. H. (2010). Evaluation of cornulin, keratin 4, keratin 13 expression and grade of dysplasia for predicting malignant progression of oral leukoplakia. Oral Oncology, 46(2), 123–127. https://doi.org/10.1016/j.oraloncology.2009.11.012

Scharenberg, C., Eckardt, A., Tiede, C., Kreipe, H., & Hussein, K. (2013). Expression of Caspase 14 and Filaggrin in Oral Squamous Carcinoma. Head and Neck Pathology, 7(4), 327–333. https://doi.org/10.1007/s12105-013-0445-0

Shi, L., Yang, Y., Li, M., Li, C., Zhou, Z., Tang, G., … Jia, H. (2022). LncRNA IFITM4P promotes immune escape by up-regulating PD-L1 via dual mechanism in oral carcinogenesis. Molecular Therapy, 30(4), 1564–1577. https://doi.org/10.1016/j.ymthe.2022.01.003

Shrestha, P, Kusakabe, M., & Mori, M. (1996). Tenascin in human neoplasia. International Journal of Oncology, 8(4), 741–755. https://doi.org/10.3892/ijo.8.4.741

Shrestha, Prashanta, Sakamoto, F., Takagi, H., Yamada, T., & Mori, M. (1994). Enhanced tenascin immunoreactivity in leukoplakia and squamous cell carcinoma of the oral cavity: An immunohistochemical study. European Journal of Cancer. Part B: Oral Oncology, 30(2), 132–137. https://doi.org/10.1016/0964-1955(94)90065-5

Silva, R. N. F., Dallarmi, L. B., Araujo, A. K. C., Alencar, R. C. G., Mendonça, E. F., Silva, T. A., … Costa, N. L. (2018). Immunohistochemical analysis of neutrophils, interleukin-17, matrix metalloproteinase-9, and neoformed vessels in oral squamous cell carcinoma. Journal of Oral Pathology and Medicine, 47(9), 856–863. https://doi.org/10.1111/jop.12762

Silva Servato, J. P., Ueira Vieira, C., de Faria, P. R., Cardoso, S. V., & Loyola, A. M. (2019). The importance of inducible nitric oxide synthase and nitrotyrosine as prognostic markers for oral squamous cell carcinoma. Journal of Oral Pathology and Medicine, 48(10), 967–975. https://doi.org/10.1111/jop.12942

Sinanoglu, A., Soluk-Tekkesin, M., & Olgac, V. (2015). Cyclooxygenase-2 and Ki67 Expression in Oral Leukoplakia: a Clinicopathological Study. Journal of Oral and Maxillofacial Research, 6(2), e3. https://doi.org/10.5037/jomr.2015.6203

Singh, R., Das, S., Datta, S., Mazumdar, A., Biswas, N. K., Maitra, A., … Roy, B. (2020). Study of Caspase 8 mutation in oral cancer and adjacent precancer tissues and implication in progression. PLoS ONE, 15(6), e0233058. https://doi.org/10.1371/journal.pone.0233058

Souto, G. R., Matias, M. D. P., Nunes, L. F. M., Ferreira, R. C., & Mesquita, R. A. (2018). Mature dendritic cell density is affected by smoking habit, lesion size, and epithelial dysplasia in oral leukoplakia samples. Archives of Oral Biology, 95, 51–57. https://doi.org/10.1016/j.archoralbio.2018.07.008

Srinivasan, M., & Jewell, S. D. (2001). Evaluation of TGF-α and EGFR expression in oral leukoplakia and oral submucous fibrosis by quantitative immunohistochemistry. Oncology, 61(4), 284–292. https://doi.org/10.1159/000055335

Sripodok, P., Saito, H., Kouketsu, A., Takahashi, T., & Kumamoto, H. (2024). Immunoexpression of SIRT1, 6, and 7 in oral leukoplakia and oral squamous cell carcinoma. Odontology, 112(1), 221–229. https://doi.org/10.1007/s10266-023-00816-x

Stasikowska-Kanicka, O., Wągrowska-Danilewicz, M., & Danilewicz, M. (2018). T cells are involved in the induction of macrophage phenotypes in oral leukoplakia and squamous cell carcinoma—a preliminary report. Journal of Oral Pathology and Medicine, 47(2), 136–143. https://doi.org/10.1111/jop.12657

Stasikowska-Kanicka, O., Wągrowska-Danilewicz, M., Kulicka, P., & Danilewicz, M. (2018). Overexpression of ADAM10 in oral squamous cell carcinoma with metastases. Polish Journal of Pathology, 69(1), 67–72. https://doi.org/10.5114/pjp.2018.75339

Su, L., Morgan, P. R., & Lane, E. B. (1996). Keratin 14 and 19 expression in normal, dysplastic and malignant oral epithelia. A study using in situ hybridization and immunohistochemistry. Journal of Oral Pathology and Medicine, 25(6), 293–301. https://doi.org/10.1111/j.1600-0714.1996.tb00265.x

Sundararajan, A., Muthusamy, R., Siva, K. G., Harikrishnan, P., Kumar, S. C. K., & Rathinasamy, S. K. (2021). Correlation of mast cell and angiogenesis in oral lichen planus, dysplasia (leukoplakia), and oral squamous cell carcinoma. Rambam Maimonides Medical Journal, 12(2). https://doi.org/10.5041/RMMJ.10438

Sundberg, J., Pandey, S., Giglio, D., Holmberg, E., Kjeller, G., Kovács, A., … Hasséus, B. (2021). Expression of p53, p63, podoplanin and Ki-67 in recurring versus non-recurring oral leukoplakia. Scientific Reports, 11(1), 20781. https://doi.org/10.1038/s41598-021-99326-5

Tanwar, R., Iyengar, A. R., Nagesh, K. S., Patil, S., & Subhash, B. V. (2016). GSTM1 null polymorphism prevalence in tobacco users, oral leukoplakia and oral squamous cell carcinoma patients in South Indian population: A polymerase chain reaction study. Indian Journal of Dental Research, 27(4), 353–358. https://doi.org/10.4103/0970-9290.191881

Thiem, D. G. E., Schneider, S., Venkatraman, N. T., Kumar, V. V., Brieger, J., Frerich, B., & Kämmerer, P. W. (2017). Semiquantifiable angiogenesis parameters in association with the malignant transformation of oral leukoplakia. Journal of Oral Pathology and Medicine, 46(9), 710–716. https://doi.org/10.1111/jop.12544

Tiitta, O., Happonen, R. ‐P, Virtanen, I., & Luomanen, M. (1994). Distribution of tenascin in oral premalignant lesions and squamous cell carcinoma. Journal of Oral Pathology & Medicine, 23(10), 446–450. https://doi.org/10.1111/j.1600-0714.1994.tb00442.x

Tsai, S. ‐T, & Jin, Y. ‐T. (1995). Proliferating cell nuclear antigen (PCNA) expression in oral squamous cell carcinomas. Journal of Oral Pathology & Medicine, 24(7), 313–315. https://doi.org/10.1111/j.1600-0714.1995.tb01191.x

Tsuji, T., Shrestha, P., Yamada, K., Takagi, H., Shinozaki, F., Sasaki, K., … Mori, M. (1992). Proliferating cell nuclear antigen in malignant and pre-malignant lesions of epithelial origin in the oral cavity and the skin: an immunohistochemical study. Virchows Archiv A Pathological Anatomy and Histopathology, 420(5), 377–383. https://doi.org/10.1007/BF01600508

Tsuji, Tatsuo, Sasaki, K., Kimura, Y., Yamada, K., Mori, M., & Shinozaki, F. (1992). Measurement of proliferating cell nuclear antigen (PCNA) and its clinical application in oral cancers. International Journal of Oral and Maxillofacial Surgery, 21(6), 369–372. https://doi.org/10.1016/S0901-5027(05)80765-9

Van Der Velden, L. A., Manni, J. J., Ramaekers, F. C. S., & Kuijpers, W. (1999). Expression of intermediate filament proteins in benign lesions of the oral mucosa. European Archives of Oto-Rhino-Laryngology, 256(10), 514–519. https://doi.org/10.1007/s004050050202

Vered, M., Allon, I., & Dayan, D. (2009). Maspin, p53, p63, and Ki-67 in epithelial lesions of the tongue: From hyperplasia through dysplasia to carcinoma. Journal of Oral Pathology and Medicine, 38(3), 314–320. https://doi.org/10.1111/j.1600-0714.2008.00698.x

von Zeidler, S. V., de Souza Botelho, T., Mendonça, E. F., & Batista, A. C. (2014). E-cadherin as a potential biomarker of malignant transformation in oral leukoplakia: A retrospective cohort study. BMC Cancer, 14(1), 972. https://doi.org/10.1186/1471-2407-14-972

Vora, H. H., Shah, N. G., Patel, D. D., Trivedi, T. I., & Choksi, T. J. (2003). BRCA1 expression in leukoplakia and carcinoma of the tongue. Journal of Surgical Oncology, 83(4), 232–240. https://doi.org/10.1002/jso.10213

Wagner, V. P., Cardoso, P. R., Dos Santos, J. N., Meurer, L., Vargas, P. A., Fonseca, F. P., … Martins, M. D. (2017). Immunohistochemical Study of TGF-β1 in Oral Leukoplakia and Oral Squamous Cell Carcinoma: Correlations between Clinicopathologic Factors and Overall Survival. Applied Immunohistochemistry and Molecular Morphology, 25(9), 651–659. https://doi.org/10.1097/PAI.0000000000000355

Wan, Z., Zheng, Z., Huang, M., Chen, Y., & Yao, L. (2021). Expression of Ki-67, Cyclin D1, P53, and P16 in patients with oral leukoplakia and leukoplakia cancerization with spicy diet in Chengdu. Hua Xi Kou Qiang Yi Xue Za Zhi / West China Journal of Stomatology, 39(4), 434–440. https://doi.org/10.7518/hxkq.2021.04.009

Wang, K., Shen, Y., Xu, J., Li, Z., Liu, Y., Yu, C., … Zeng, Y. (2020). Evaluation of synuclein-γ levels by novel monoclonal antibody in saliva and cancer tissues from oral squamous cell carcinoma patients. Neoplasma, 67(3), 707–713. https://doi.org/10.4149/neo_2020_190619N523

Wang, S., Li, F., Fan, H., Xu, J., & Hu, Z. (2019). Expression of PIWIL2 in oral cancer and leukoplakia: Prognostic implications and insights from tumors. Cancer Biomarkers, 26(1), 11–20. https://doi.org/10.3233/CBM-182009

Warnakulasuriya, K. A. A. S., & Johnson, N. W. (1993). Nucleolar organiser region (NOR) distribution as a diagnostic marker in oral keratosis, dysplasia and squamous cell carcinoma. Journal of Oral Pathology & Medicine, 22(2), 77–81. https://doi.org/10.1111/j.1600-0714.1993.tb00048.x

Watanabe, S., Sato, K., Okazaki, Y., Tonogi, M., Tanaka, Y., & Yamane, G. yuki. (2009). Activation of PI3K-AKT pathway in oral epithelial dysplasia and early cancer of tongue. The Bulletin of Tokyo Dental College, 50(3), 125–133. https://doi.org/10.2209/tdcpublication.50.125

Webber, L. P., Wagner, V. P., Curra, M., Vargas, P. A., Meurer, L., Carrard, V. C., … Martins, M. D. (2017). Hypoacetylation of acetyl-histone H3 (H3K9ac) as marker of poor prognosis in oral cancer. Histopathology, 71(2), 278–286. https://doi.org/10.1111/his.13218

Wenghoefer, M., Pantelis, A., Najafi, T., Deschner, J., Allam, J. P., Novak, N., … Winter, J. (2010). Gene expression of oncogenes, antimicrobial peptides, and cytokines in the development of oral leukoplakia. Oral Surgery, Oral Medicine, Oral Pathology, Oral Radiology and Endodontology, 110(3), 351–356. https://doi.org/10.1016/j.tripleo.2009.08.013

Wilkman, T. S. E., Hietanen, J. H. P., Malmström, M. J., & Konttinen, Y. T. (1998). Immunohistochemical analysis of the oncoprotein c-erbB-2 expression in oral benign and malignant lesions. International Journal of Oral and Maxillofacial Surgery, 27(3), 209–212. https://doi.org/10.1016/S0901-5027(98)80012-X
[truncated: 6,343 more chars]
